# RSSDI clinical practice recommendations for the management of type 2 diabetes mellitus 2017

**DOI:** 10.1007/s13410-018-0604-7

**Published:** 2018-02-28

**Authors:** Sarita Bajaj

**Affiliations:** Department of Medicine, MLN Medical College, Allahabad, UP India

## Steering committee

**Chairperson:** Dr. Sarita Bajaj

**Members:** Dr. Anuj Maheshwari, Dr. Banshi Saboo, Dr. B. M. Makkar, Dr. C. R. Anand Moses, Dr. Ch. Vasanth Kumar, Dr. J. Jayaprakashsai, Dr. Jayant Panda, Dr. K. R. Narasimha Setty, Dr. P. V. Rao, Dr. Rajeev Chawla, Dr. Rakesh Sahay, Dr. Samar Banerjee, Dr. Sanjay Agarwal, Dr. Sanjay Kalra, Dr. S. R. Aravind, Dr. Sujoy Ghosh, Dr. Sunil Gupta, Dr. S. V. Madhu, Dr. Vijay Panikar, Dr. Vijay Viswanathan

**Table:** 

Members of the expert panel group for each section	
Diagnosis	Dr. S. R. Aravind (Coordinator), Dr. C. Munichoodappa, Dr. V. Mohan, Dr. K. M. Prasanna Kumar, Dr. G. R. Sridhar, Dr. Vageesh Ayyar, Dr. Ranjit Unnikrishnan, Dr. Sanjay Reddy, Dr. Bhavana Sosale, Dr. R. M. Anjana, Dr. Subhankar Chowdhury
Screening and early detection	Dr. S. V. Madhu (Coordinator), Dr. Nikhil Tandon, Dr. A. Ramachandran, Dr. D. Bachani, Dr. Subhankar Chowdhury, Dr. A. Aggarwal
Obesity and diabetes	Dr. B. M. Makkar (Coordinator), Dr. Anoop Misra, Dr. Naval Vikram, Dr. R. M. Anjana, Dr. Sujoy Ghosh, Dr. Neeta Deshpande, Dr. J. K. Sharma
Diet therapy	Dr. P. V. Rao (Coordinator), Dr. Ch. Vasanth Kumar, Dr. S. V. Madhu, Dr. K. M. Prasanna Kumar, Dr. A. K. Das, Dr. Sarita Bajaj, Dr. G. R. Sridhar
Lifestyle management	Dr. Rakesh Sahay (Coordinator), Dr. K. R. Narasimha Setty, Dr. B. K. Sahay, Dr. Anoop Misra, Dr. Ganapathi Bantwal, Dr. A. G. Unnikrishnan, Dr. Nihal Thomas
Education	Dr. Sunil Gupta (Coordinator), Dr. G. C. Reddy, Dr. J. Jayaprakashsai, Dr. B. K. Sahay, Dr. N. Sudhakar Rao, Dr. P. V. Rao
Oral antidiabetic agents	Dr. Vijay Panikar (Coordinator), Dr. Banshi Saboo, Dr. Jayant Panda, Dr. Shashank R. Joshi, Dr. Samar Banerjee, Dr. Vijay Viswanathan, Dr. Anil Bhoraskar, Dr. Vijay Negalur, Dr. V. Chopra, Dr. V. Mohan, Dr. G. R. Sridhar, Dr. Sujoy Ghosh, Dr. Alok Kanungo, Dr. Sambit Das, Dr. A. K. Das, Dr. Ajay Kumar, Dr. Arvind Gupta, Dr. Urman Dhruv, Dr. Sanjeev Phatak, Dr. Mangesh Tiwaskar
Injectables	Dr. Sujoy Ghosh (Coordinator), Dr. Banshi Saboo, Dr. Jayant Panda, Dr. Shashank R. Joshi, Dr. Samar Banerjee, Dr. Vijay Viswanathan, Dr. Anil Bhoraskar, Dr. Vijay Negalur, Dr. V. Chopra, Dr. V. Mohan, Dr. G. R. Sridhar, Dr. Alok Kanungo, Dr. Sambit Das, Dr. A. K. Das, Dr. Ajay Kumar, Dr. Arvind Gupta, Dr. Urman Dhruv, Dr. Sanjeev Phatak, Dr. Mangesh Tiwaskar
Alternate therapies	Dr. K. R. Narasimha Setty, Dr. S. V. Madhu, Dr. K. M. Prasanna Kumar, Dr. A. K. Das, Dr. Sarita Bajaj, Dr. G. R. Sridhar
Individualizing therapy	Dr. Sanjay Agarwal (Coordinator), Dr. Rajeev Chawla, Dr. S. V. Madhu
Postprandial hyperglycemia	Dr. Anuj Maheshwari (Coordinator), Dr. Sarita Bajaj, Dr. B. K. Sahay, Dr. Banshi Saboo, Dr. Manash P. Baruah, Dr. Ameya Joshi, Dr. Sameer Aggarwal
Clinical monitoring	Dr. C. R. Anand Moses (Coordinator), Dr. C Munichoodappa, Dr. Krishna Seshadri, Dr. A. G. Unnikrishnan, Dr. Ganapathi Bantwal, Dr. Mala Dharmalingam, Dr. R. M. Anjana, Dr. Bhavana Sosale, Dr. Sanjay Reddy, Dr. Neeta Deshpande
Self-monitoring	Dr. Ch. Vasanth Kumar (Coordinator), Dr. Samar Banerjee, Dr. Debmalya Sanyal, Dr. Sunil Gupta
Chronic complications	Dr. Rajeev Chawla (Coordinator), Dr. Viay Viswanathan, Dr. Sudha Vidyasagar, Dr. S. K. Singh, Dr. Shalini Jaggi, Dr. Hitesh Punyani, Dr. Vinod Mittal, Dr. R. K. Lalwani
Infection and vaccinations	Dr. Jayant Panda (Coordinator), Dr. Sidhartha Das, Dr. A. K. Das, Dr. Vijay Viswanathan, Dr. Abhaya Kumar Sahu, Dr. Ramesh K. Goenka
Fasting and diabetes	Dr. Sarita Bajaj (Coordinator), Dr. Sanjay Kalra, Dr. Sandeep Julka, Dr. Yashdeep Gupta, Dr. Navneet Agarwal
Diabetes and CV risk	Dr. Sanjay Kalra (Coordinator), Dr. Gagan Priya, Dr. Jubbin Jacob, Dr. Sameer Aggarwal, Dr. Deepak Khandelwal
Hypoglycemia	Dr. Vijay Viswanathan (Coordinator), Dr. Mangesh Tiwaskar, Dr. Girish Mathur
Technologies	Dr. Banshi Saboo (Coordinator), Dr. S. R. Aravind, Dr. Jothydev Kesavadev, Dr. Manoj Chawla, Dr. Rajeev Kovi

### Table of contents

**Table:** 

Preface	8
Methodology	11
Diagnosis of diabetes	13
Screening/early detection of diabetes/prediabetes	17
Obesity and diabetes	35
Diet therapy	47
Lifestyle management	64
Education	77
Oral antidiabetic agents	87
Injectables	97
Alternate therapies	115
Individualizing therapy	118
Postprandial hyperglycemia	132
Clinical monitoring	142
Targets of glucose control	143
Self-monitoring of blood glucose	150
Chronic complications	158
Infections and vaccinations	187
Fasting and diabetes	197
Diabetes and CV risk	208
Hypoglycemia	226
Technologies	233
Annexures	241

### Abbreviations (alphabetical order)

**Table:** 

A1C	Glycated hemoglobin	IDRS	Indian Diabetes Risk Score
ACE	Angiotensin converting enzyme	IFG	Impaired fasting glucose
ACR	Albumin-to-creatinine ratio	IGT	Impaired glucose tolerance
ACS	Acute coronary syndrome	IR	Insulin resistance
ADA	American Diabetes Association	LDL	Low density lipoprotein
AGIs	Alpha-glucosidase inhibitors	MI	Myocardial infarction
ARB	Angiotensin receptor blocker	MNT	Medical Nutrition Therapy
BMI	Body mass index	MS	Metabolic syndrome
CAD	Coronary artery disease	MUFA	Monounsaturated fatty acids
		NDS	Neuropathy Disability Score
CHF	Congestive heart failure	NSS	Neuropathy Symptom Score
CKD	Chronic kidney disease	OADs	Oral antidiabetic agents
CURES	Chennai Urban Rural Epidemiological Study	OGTT	Oral glucose tolerance test
CV	Cardiovascular	PAD	Peripheral arterial disease
CVD	Cardiovascular disease	PPG	Postprandial glucose
DBP	Diastolic blood pressure	PUFA	Polyunsaturated fatty acids
DM	Diabetes mellitus	PVD	Peripheral vascular disease
DN	Diabetic neuropathy	QoL	Quality of life
DPP-4	Dipeptidyl peptidase-4	RCT	Randomized controlled trial
DR	Diabetic retinopathy	SBP	Systolic blood pressure
DSME	Diabetes self-management education	SGLT 2	Sodium-glucose cotransporter 2
ESRD	End stage renal disease	SMBG	Self-monitoring of blood glucose
GFR	Glomerular filtration rate	SU	Sulfonylurea
GLP-1	Glucagon like peptide-1	T2DM	Type 2 diabetes mellitus
HDL	High density lipoprotein	UTI	Urinary tract infection
HYQ	Hydroxychloroquine	WC	Waist circumference
IDA	Iron deficiency anemia	WHO	World Health Organization
IDF	International Diabetes Federation	WHR	Waist-to-hip ratio

#### **Preface**

Management of diabetes, a disease which is assuming epidemic proportions, remains a challenge despite the availability of numerous guidelines. According to International Diabetes Federation (IDF) 2015 estimates, globally 415 million people are suffering from diabetes and this figure may reach up to 642 million in 2040 [1]. Currently, 78.3 million people with diabetes are in Southeast Asia (SEA) region and this may rise up to 140.2 million in 2040 if proper measures are not taken [1]. India has the second largest population (69.2 million) with diabetes in the world after China (109.7 million) [2]. In addition, approximately 52% adults with diabetes remain undiagnosed in India. Large-scale surveys, such as District Level Household and Facility Survey (DLHS) 2012–2013 and Annual Health Survey (AHS) 2014, have reported that around 7% Indian adults are suffering from diabetes and the prevalence is higher in urban (9.8%) compared to rural areas (5.7%) [3].

Type 2 diabetes mellitus (T2DM) is a progressive metabolic disorder characterized by abnormal insulin secretion and utilization. Indian patients with T2DM have distinctive clinical and biochemical characteristics, which makes them the so-called Asian Indian Phenotype [4–7]. These abnormalities include more insulin resistance (IR), elevated abdominal adiposity (i.e., higher visceral fat in spite of lower body mass index [BMI]), lower level of adiponectin, and a higher level of highly sensitive C-reactive protein [4–7]. Moreover, they have greater propensity to develop cardiovascular (CV) complications such as coronary artery disease (CAD) and atherosclerosis at any age point [4, 8] and have significant pro-coagulant affinities [9–11]. At the same BMI, more Asian Indians develop metabolic syndrome (MS) and diabetes compared to their western counterparts [12]. In spite of a relatively lower rate of obesity as defined by international BMI cut-off points, Indians tend to have larger waist circumference (WC) and waist-to-hip ratios (WHR), indicating a greater degree of central body obesity [13]. Asian Indians also have increased metabolic risk compared to their western counterparts due to high leptin levels [14], greater IR [6, 15, 16], higher insulin sensitivity index and lower acute insulin response to glucose [17], early loss of β-cell function [6], “concept of thin-fat Indian,” or “sarcopenic obesity” [18].

Numerous international and national guidelines are in place for the management of T2DM. Adopting country-specific guidelines improves treatment outcomes in diabetes. The Research Society for the Study of Diabetes in India (RSSDI) therefore published the clinical practice recommendations for the management of T2DM in 2015, which was adapted from the 2014 global guidelines for type 2 diabetes by IDF. The recommendations were specifically designed considering the diverse socioeconomic and cultural background of Indians. The 2015 RSSDI guidelines were very well accepted by the healthcare practitioners across India and helped in decision-making.

However, diabetes research is continuously evolving. Every year, several publications are being added to the literature which can significantly impact holistic diabetes care. Addition of new medicines to the diabetes management armamentarium, which is beneficial in terms of reduction in morbidity and mortality risk, is transforming the treatment of T2DM. Keeping this in mind, in the 2017 update, all of the sections have been updated with recent evidence. Moreover, three new sections, Hypoglycemia, Diabetes and CV risk, and Technologies, have been incorporated and some of the annexures revised. The objective of this updated RSSDI 2017 clinical practice recommendations is to provide evidence-based recommendations for the treatment of patients with T2DM. It is expected that these recommendations will help define practically implementable best practices not only for the management of T2DM but also help in timely prevention of acute and chronic complications of diabetes by primary care physicians across India.

**References**

1. International Diabetes Federation. The International Federation (IDF) diabetes atlas, seventh edition. 2015. Available from http://www.diabetesatlas.org/
2. IDF diabetes atlas—7th edition 2015. Across the globe, available at http://www.diabetesatlas.org/across-the-globe.html
3. Akhtar SN, Dhillon P. Prevalence of diagnosed diabetes and associated risk factors: evidence from the large-scale surveys in India. Journal of Social Health and Diabetes. 2017;5(1):28.
4. Mohan V, Sandeep S, Deepa R, Shah B, Varghese C. Epidemiology of type 2 diabetes: Indian scenario. Indian Journal of Medical Research. 2007;125(3):217.
5. Joshi SR, Anjana RM, Deepa M, Pradeepa R, Bhansali A, Dhandania VK, Joshi PP, Unnikrishnan R, Nirmal E, Subashini R, Madhu SV. Prevalence of dyslipidemia in urban and rural India: the ICMR–INDIAB study. PLOS One. 2014;9(5):e96808.
6. Shah VN, Mohan V. Diabetes in India: what is different? Current opinion in endocrinology. Diabetes and Obesity. 2015;22(4):283–9.
7. Unnikrishnan R, Anjana RM, Mohan V. Diabetes in South Asians: is the phenotype different? Diabetes. 2014;63(1):53–5.
8. Enas EA, Mohan V, Deepa M, Farooq S, Pazhoor S, Chennikkara H. The metabolic syndrome and dyslipidemia among Asian Indians: a population with high rates of diabetes and premature coronary artery disease. J Cardiometab Syndr. 2007;2(4):267–75.
9. Hughes K, Aw TC, Kuperan P, Choo M. Central obesity, insulin resistance, syndrome X, lipoprotein(a), and cardiovascular risk in Indians, Malays, and Chinese in Singapore. J Epidemiol Community Health. 1997;51(4):394–9
10. Kain K, Blaxill JM, Catto AJ, Grant PJ, Carter AM. Increased fibrinogen levels among South Asians versus Whites in the United Kingdom are not explained by common polymorphisms. Am J Epidemiol. 2002;156(2):174–9.
11. Anand SS, Yusuf S, Vuksan V, Devanesen S, Teo KK, Montague PA, et al. Differences in risk factors, atherosclerosis, and cardiovascular disease between ethnic groups in Canada: the Study of Health Assessment and Risk in Ethnic groups (SHARE). Lancet. 2000;356(9226):279–284.
12. Ma RC, Chan JC. Type 2 diabetes in East Asians: similarities and differences with populations in Europe and the United States. Annals of the New York Academy of Sciences. 2013;1281(1):64–91
13. Ramachandran A, Snehalatha C, Viswanathan V, Viswanathan M, Haffner SM. Risk of noninsulin dependent diabetes mellitus conferred by obesity and central adiposity in different ethnic groups: a comparative analysis between Asian Indians, Mexican Americans and Whites. Diabetes Res Clin Pract 1997;36(2):121–5.
14. Lilja M, Rolandsson O, Shaw JE, Pauvaday V, Cameron AJ, Tuomilehto J, Alberti KG, Zimmet PZ, Söderberg S. Higher leptin levels in Asian Indians than Creoles and Europids: a potential explanation for increased metabolic risk. International journal of obesity. 2010;34(5):878.
15. Gao H, Salim A, Lee J, Tai ES, Van Dam RM. Can body fat distribution, adiponectin levels and inflammation explain differences in insulin resistance between ethnic Chinese, Malays and Asian Indians? International Journal of Obesity. 2012;36(8):1086.
16. Abdullah N, Attia J, Oldmeadow C, Scott RJ, Holliday EG. The architecture of risk for type 2 diabetes: understanding Asia in the context of global findings. International Journal of Endocrinology. 2014.
17. Kodama K, Tojjar D, Yamada S, Toda K, Patel CJ, Butte AJ. Ethnic differences in the relationship between insulin sensitivity and insulin response. Diabetes care. 2013;36(6):1789–96
18. Joshi SR. Diabetes care in India. Annals of Global health. 2015;81(6):830–8.

#### **Methodology**

The methodology followed to develop the current recommendations is similar to that used to prepare RSSDI 2015 recommendations. A steering committee of RSSDI involving experienced diabetologists and endocrinologists across India was constituted, which met twice in the year 2017. The steering committee deliberated and defined the scope of the recommendations. As in the case of 2015 version, for all sections of the recommendations, expert panel groups were constituted including one steering committee member as a coordinator with several national experts to review all available Indian, Asian, and global evidence and make suitable recommendations.

The members of the expert panel reviewed the previous RSSDI recommendations and formulated recommendations based on available research evidence and economic and logistic constraints prevalent in India. Where there was little or no evidence, the group relied on their clinical experience and expertise, judgment, and consensus to make the recommendations. The recommendations of the expert panel groups were reviewed by the steering committee and were then finalized by the writing group as a draft consensus document.

The decision on the analytical reevaluation of the recommendations proposed by RSSDI 2017 was based on the Indian evidence published between 1990 and 2017 and following a review of relevant local factors in the Indian context. This included Indian evidence from indexed literature searches, articles published in the *International Journal of Diabetes in Developing* Countries (IJDDC), *RSSDI Textbook of Diabetes* (third edition) [1, 2], *Journal of Association of Physicians of India* (JAPI), and personal communications from authors. Where Indian evidence was not available, Asian/global evidence was considered where available.

Similar to the 2015 document, only “Recommended care” and “Limited care” setting of the IDF guidelines have been considered while the “Comprehensive care” was omitted. This practical approach facilitates implementation of cost-effective evidence-based care in limited resource settings like India.

- *Recommended care* constitutes evidence-based care which is cost-effective. Interventions should be made available to all people with diabetes with an aim of any healthcare system to achieve this level of care.
- *Limited care* is the lowest level of care that seeks to achieve the major objectives of diabetes management provided in healthcare settings with very limited resources such as drugs, personnel, technologies, and procedures.

**References**

1. Chandalia HB, Sridhar GR, Das AK, Madhu SV, Mohan V, Rao PV. RSSDI textbook of diabetes mellitus. 2014, 3rd edition, Jaypee Brothers Medical Publishers (P) Ltd. New Delhi, India.
2. http://www.rssdi.in/journal_ijddc.php

## **Diagnosis of diabetes**

### **RSSDI 2017 recommendations**

#### **Recommended care**

Diabetes can be diagnosed with any of the following criteria:

- Fasting plasma glucose (FPG) ≥ 126 mg/dL* *or*
- Oral glucose tolerance test (OGTT) using 75 g of anhydrous glucose with FPG ≥ 126 mg/dL and/or 2-h plasma glucose ≥ 200 mg/dL *or*
- Glycated hemoglobin (A1C) ≥ 6.5%** *or*
- Random plasma glucose ≥ 200 mg/dL in the presence of classical diabetes symptoms
- Asymptomatic individuals with a single abnormal test should have the test repeated to confirm the diagnosis unless the result is unequivocally abnormal.

#### **Limited care**

Diabetes can be diagnosed with any of the following criteria:

- FPG ≥ 126 mg/dL* *or*
- 75 g OGTT (using 75 g of anhydrous glucose) with FPG ≥ 126 mg/dL and/or 2-h plasma glucose ≥ 200 mg/dL *or*
- Random plasma glucose ≥ 200 mg/dL in the presence of classical diabetes symptoms
- Asymptomatic individuals with a single abnormal test should have the test repeated to confirm the diagnosis unless the result is unequivocally abnormal.

*FPG is defined as glucose estimated after no caloric intake for at least 8–12 h.

**Using a method that is National Glycohemoglobin Standardization Program (NGSP) certified. For more on A1C and NGSP, please visit http://www.ngsp.org/index.asp [10]

Note:

1. Point of care device for estimation of A1C is not recommended for diagnosis.
2. Capillary glucose estimation methods are not recommended for diagnosis.
3. Venous plasma is used for estimation of blood glucose.
  - Plasma must be separated soon after collection because the blood glucose levels drop by 5–8% hourly if whole blood is stored at room temperature.
  For more details on glucose estimation, visit http://www.ncbi.nlm.nih.gov/books/NBK248/ [11].

#### **Preamble**

Traditionally, measuring FPG and OGTT is often considered for diagnosis [1] despite several international guidelines recommending A1C as a diagnostic tool for detecting diabetes/prediabetes [2, 3]. The optimal cut-off value of A1C to diagnose diabetes is determined in a way that individuals with A1C levels above a certain cut-off value have a much larger probability of having or developing diabetes-related complications [4]. However, in several countries including India, there is no consensus on a suitable cut-off point of A1C for diagnosis of diabetes. Moreover, measuring A1C is more expensive than FPG [5] and standardization of measurement techniques and laboratories is poorly practiced across the country [6]. Nonetheless, a recent review has included A1C as a criterion for the diagnosis of diabetes in India [7]. In lieu of this, the panel felt that using A1C as sole criteria for diagnosis of diabetes is inappropriate in resource constraint settings and framing recommendations based on fasting or 2-h plasma glucose or OGTT to detect or diagnose diabetes would be more appropriate in limited resource settings like India.

#### **Considerations**

The decision about setting diagnostic thresholds values was based on the cost-effective strategies for diagnosing diabetes that were reviewed in Indian context.

#### **Rationale and evidence**

##### *A1C cut-off for diagnosis of diabetes in Indian patients*

- The panel opined that A1C cut-off point of 6.5% is optimal for the diagnosis of diabetes in Indian patients. This was based on the data available from four centers in India: Chandigarh, Chennai, Bangalore, and Andhra Pradesh. Data from a community-based randomized cross-sectional study in urban Chandigarh suggest that A1C cut point of 6.5% has optimal specificity of 88%, while cut-off point of 7.0% has sensitivity of 92% for the diagnosis of diabetes [8]. On the other hand, data from Chennai Urban Rural Epidemiology Study (CURES) demonstrated 88.0% sensitivity and 87.9% specificity for detection of diabetes when A1C cut-off point is 6.1% (based on 2-h postload plasma glucose) and 93.3% sensitivity and 92.3% specificity when A1C cut-off point is 6.4% (when diabetes was defined as FPG ≥ 7.0 mmol/l) [1]. Furthermore, *Mohan et al.* derived and validated the A1C cut-off value of > 6.3% in the ethnic population of Rayalaseema area of Andhra Pradesh state. Study reports that there is no significant difference in the training and validation data set and concludes that A1C > 6.3% appeared to be the optimal cut-off value for the diagnosis of T2DM [9].
- The panel opined that A1C cannot be used as “sole” measurement for the diagnosis of diabetes in Indian settings. However, panel emphasized that A1C can be used in settings where an appropriate standardized method is available.

#### **Implementation**

Individuals should be educated on the advantages of early diagnosis and should be encouraged to participate in community screening programs for diagnosis.

**References**

1. Mohan V, Vijayachandrika V, Gokulakrishnan K, Anjana RM, Ganesan A, Weber MB, et al. A1C cut points to define various glucose intolerance groups in Asian Indians. Diabetes Care. 2010;33(3):515–9.
2. Inzucchi SE, Bergenstal RM, Buse JB, Diamant M, Ferrannini E, Nauck M, et al. Management of hyperglycemia in type 2 diabetes, 2015: a patient-centered approach: update to a position statement of the American Diabetes Association and the European Association for the Study of Diabetes. Diabetes Care. 2015;38(1):140–9.
3. Handelsman Y, Bloomgarden ZT, Grunberger G, Umpierrez G, Zimmerman RS, Bailey TS, et al. American Association of Clinical Endocrinologists and American College of Endocrinology—clinical practice guidelines for developing a diabetes mellitus comprehensive care plan—2015. Endocr Pract. 2015;21 Suppl 1:1–87.
4. Kim JM, Kim DJ. The optimal cutoff value of glycated hemoglobin for detection of diabetic retinopathy. Diabetes Metab J. 2015;39(1):16–26.
5. Bennett CM, Guo M, Dharmage SC. HbA(1c) as a screening tool for detection of type 2 diabetes: a systematic review. Diabet Med. 2007;24(4):333–43.
6. Kondaveeti SB, Shaker A, Chidambaram R. Utility of glycated albumin in diagnosis of type 2 diabetes: an Indian perspective study. International Journal of Biomedical and Advance Research. 2014;5(12):615–8.
7. Madhu SV, Srivastava S. Diabetes mellitus: diagnosis and management guidelines. Journal International Medical Sciences Academy. 2015;1:47–50.
8. Kumar PR, Bhansali A, Ravikiran M, Bhansali S, Dutta P, Thakur JS, et al. Utility of glycated hemoglobin in diagnosing type 2 diabetes mellitus: a community-based study. J Clin Endocrinol Metab. 2010;95(6):2832–5.
9. Mohan A, Reddy SA, Sachan A, Sarma KV, Kumar DP, Panchagnula MV, Rao PS, Kumar BS, Krishnaprasanthi P. Derivation & validation of glycosylated haemoglobin (HbA 1c) cut-off value as a diagnostic test for type 2 diabetes in south Indian population. Indian Journal of Medical Research. 2016;144(2):220.
10. NGSP harmonizing haemoglobin A1c testing. Available at: http://www.ngsp.org/index.asp (Last accessed on 27 Aug 2015).
11. McMillin JM. Blood Glucose. Chapter 141. Clinical methods: the history, physical, and laboratory examinations. 3rd edition. Available at: http://www.ncbi.nlm.nih.gov/books/NBK248/ (Last accessed on 27 Aug 2015).

## **Screening/early detection of diabetes/prediabetes**

### **RSSDI 2017 recommendations**

#### **Recommended care**

- Each health service provider should decide whether to have a program to detect people with undiagnosed diabetes
  - This decision should be based on the prevalence of undiagnosed diabetes and available support from healthcare system/service capable of effectively treating newly detected cases of diabetes
  - Opportunistic screening for undiagnosed diabetes and prediabetes is recommended. It should include:
    - Individuals presenting to healthcare settings for unrelated illness
    - Family members of diabetes patients
    - Antenatal care
    - People over the age of 30 years should be encouraged for voluntary testing for diabetes
  - Community screening may be done wherever feasible
- Detection programs should be usually based on a two-step approach:
  - Step 1: Identify high-risk individuals using a risk assessment questionnaire
  - Step 2: Glycemic measure in high-risk individuals
- Where a random non-FPG level ≥ 100 mg/dL to ˂ 200 mg/dL is detected, FPG should be measured or OGTT should be performed
- Use of A1C as a sole diagnostic test for screening of diabetes/prediabetes is not recommended
- People with high blood glucose during screening need further diagnostic testing to confirm diabetes while those with screen-negative to diabetes should be retested as advised by the physicians
- Paramedical personnel such as nurses or other trained workers should be included in any basic diabetes care team

#### **Limited care**

- Detection programs should be opportunistic and limited to high-risk individuals in very limited settings
- The principles for screening are as for recommended care
- Diagnosis should be based on FPG or capillary plasma glucose if only point-of-care testing is available
- Using FPG alone for diagnosis has limitations as it is less sensitive than 2-h OGTT in Indians

### **Prediabetes**

#### **Recommended care**

- People with screen-positive for prediabetes (FPG = 100 to 125 mg/dL or 2-h plasma glucose in the 75-g OGTT = 140 to 199 mg/dL or A1C = 5.7 to 6.4%) should be monitored for development of diabetes annually and simultaneously screened and treated for modifiable risk factors for cardiovascular disease (CVD) such as hypertension, dyslipidemia, smoking, and alcohol consumption
- Screening strategies should be linked to healthcare system with capacity to provide advice on lifestyle modifications:
  - Screening strategies should be aligned with ongoing support national programs available at community health centers or above
  - Patients with impaired glucose tolerance (IGT), impaired fasting glucose (IFG) should be referred to these ongoing support programs
- People with prediabetes should modify their lifestyle including:
  - Attempts to lose 5 to 10% of body weight if overweight or obese
  - Participate in moderate physical activity (e.g., walking) for at least 150 mins/week
  - 6–8 h of sleep daily
- Healthy lifestyle measures including diet and physical activity are equally important for non-obese patients with prediabetes
- People with prediabetes failing to achieve any benefit on lifestyle modifications after 6 months may be initiated on oral antidiabetic agents (OADs)
  - Metformin: In younger individuals with one or more additional risk factors for diabetes regardless of BMI
  - Alternatively, alpha-glucosidase inhibitors (AGIs) such as acarbose or voglibose may be initiated if metformin is not tolerated
- Other pharmacological interventions with pioglitazones, orlistat, vitamin D, or bariatric surgery are not recommended
- People with prediabetes should be educated on:
  - Weight management
  - Physical activity
  - Alcohol and tobacco consumption

#### **Limited care**

- The principles of detection and management of prediabetes are same as for recommended care
- Linkages to healthcare system with capacity to provide advice on lifestyle modifications and alignment with ongoing support national programs available at community health centers where patients detected with prediabetes can be referred are critical

#### **Preamble**

Chronic hyperglycemia is associated with significantly higher risk of developing diabetes-related microvascular and macrovascular complications. Early detection of diabetes/prediabetes through screening increases the likelihood of identifying asymptomatic individuals and provides adequate treatment to reduce the burden of diabetes and its complications. Through a computer-simulated model on the data from the Anglo-Danish-Dutch study of intensive treatment in people with screen-detected diabetes in primary care (ADDITION-Europe), *Herman et al.* have demonstrated that the absolute risk reduction (ARR) and relative risk reduction (RRR) for CV outcomes are substantially higher at 5 years with early screening and diagnosis of diabetes when compared to 3 years (3.3% ARR, 29% RRR) or 6 years of delay (4.9% ARR, 38% RRR) [1]. Adopting a targeted approach and utilizing low-cost tools with meticulous planning and judicious allocation of resources can make screening cost-effective even in resource-constrained settings like India [2]. Furthermore, in a systematic review and meta-analysis, screening for T2DM and prediabetes is found to be cost-effective when initiated at around 45–50 years of age with repeated testing every 5 years [3].

Prediabetes is defined as blood glucose concentration higher than normal but lower than established thresholds for diagnosis of diabetes. People with prediabetes are defined by having IGT (2-h plasma glucose in the 75-g OGTT = 140 to 199 mg/dL) or IFG (FPG = 100 to 125 mg/dL). It is a state of intermediate hyperglycemia with increased risk of developing diabetes and associated CV complications and therefore early detection and treatment of prediabetic IGT and IFG is necessary to prevent the rising epidemic of diabetes and its associated morbidity and mortality. Although IDF guideline does not deal with screening and management of prediabetes, the American Diabetes Association (ADA) recommends screening for prediabetes and T2DM through informal assessment of risk factors or with an assessment tool guidelines [4]. Given the high prevalence rates of prediabetes in our country, RSSDI panel members opine that including screening and management aspects of prediabetes is logical and will provide an important opportunity for prevention of diabetes in India.

#### **Considerations**

The decision about conducting a screening program should be based on the following local factors than were reviewed in Indian context: limited resources, lack of quality assurance in labs, high-risk population for diabetes, large unrecognized burden of undiagnosed diabetes, high prevalence of prediabetes, among fastest converting population form prediabetes to diabetes, large rural–urban divide, largely sedentary population in urban areas, onset of T2DM at least a decade earlier that in western countries, newer technologies for screening, cost of early detection to the individual, capacity for carrying out screening, and capacity to treat/manage screen positive individuals with diabetes and prediabetes.

#### **Rationale**

##### *Opportunistic screening*

- The panel opine that screening should be opportunistic but not community based as they are less effective outside healthcare setting and poorly targeted, i.e., it may fail to identify individuals who are at risk. In a cross-sectional study on 215 participants in a tertiary care hospital in Haryana, opportunistic screening showed that for every seven patients with known diabetes there are four undiagnosed diabetes patients [5]. Opportunistic screening is more cost-effective with better feasibility within the healthcare system while minimizing the danger of medicalization of a situation. Furthermore, patients diagnosed through opportunity screening have good prognosis over those diagnosed by clinical onset of symptoms [6]. However, community screening may be carried out wherever feasible.
- The panel suggest opportunistic screening in:
  - Individuals presenting to healthcare settings for unrelated illness
  - Adult family members of patients with diabetes
  - Antenatal care
  - People over the age of 30 years should be encouraged for voluntary testing for diabetes

##### *Risk assessment questionnaire*

- There are two risk scores specific for Indians developed by Madras Diabetes Research Foundation (MDRF) and by *Ramachandran et al.* [7] (Annexures I and II). The latter is a simple one with few risk variables listed and can be applied at any work site by the paramedical personnel. Both risk scores are validated and are being used widely in our country. The Indian Diabetes Risk Score (IDRS) tool has been found to be useful for identifying undiagnosed subjects with diabetes in India and could make screening programs more cost-effective [8]. It is also used in several national programs for prevention of not only diabetes but also cardiometabolic diseases such as stroke. Also its applicability in identifying prevalence of diabetes-related complications such as CAD, peripheral vascular disease (PVD), and neuropathy among T2DM patients has been found to be successful [9].

##### *Random plasma glucose level*

- The panel endorse the IDF recommendation on the need to measure FPG and perform OGTT based on random plasma glucose levels which are associated with the development of diabetes (2 h plasma glucose ≥ 200 mg/dL) or prediabetes (2 h plasma glucose ≥ 140 to <200 mg/dL) [10]. According to IDF guidelines, FPG values ≤100 mg/dL are considered normal. Anything above 100 mg/dL is considered to be at risk of developing diabetes. Moreover, people with FPG levels between 100 and 125 mg/dL have IFG, suggesting an increased risk of developing T2DM. Confirming the FPG levels ≥ 126 mg/dL by repeating the test on another day, indicates that a person has diabetes [11]. In a cross-sectional study on 13,792 non-fasting National Health and Nutrition Examination Surveys (NHANES) participants without diagnosed diabetes, random blood sugar level of ≥ 100 mg/dL was strongly associated with undiagnosed diabetes [12]. In addition, prediction of diabetes carried out on the basis of this data showed that random blood glucose ≥ 100 mg/dL was 81.6% (95% CI = 74.9%, 88.4%) sensitive and 78% (95% CI = 76.6%, 79.5%) specific to detect undiagnosed diabetes, which is better than current screening guidelines [13]. In patients with no history of diabetes or prediabetes, random blood glucose screening is effective in promoting additional screening among high-risk age groups and encourages subjects to make lifestyle changes [14].
- The panel opine that although the present criteria of IFG (100 to 125 mg/dL) may be sensitive and has lesser variability, measuring 2-h plasma glucose levels may give more accuracy and confidence in targeting this population for prevention strategies.

##### *A1C as criteria for screening*

- A systematic review and meta-analysis of 49 studies involving patients ≥ 18 years of age has found that A1C as screening test for prediabetes has lesser sensitivity (49%) and specificity (79%) [15]. Moreover, the use of ADA recommended A1C threshold value of 6.5% for diagnosis of diabetes may result in significant underdiagnosis [16]. The predictive value of A1C for T2DM depends on various factors such as ethnicity, age, and presence of iron deficiency anemia (IDA) [17–20]. In a cohort study on individuals from Swedish and Middle-East ancestry, A1C ≥ 48 mmol/mol had a predictive sensitivity of 31 and 25%, respectively, for T2DM [18]. Furthermore, A1C values ≥ 42 and ≥ 39 mmol/mol as predictors for prediabetes were associated with a sensitivity of 15 and 34% in individuals of Swedish and 17 and 36% in individuals of Middle-East ancestry. Similarly, a systematic review and meta-analysis of 12 studies including 49,238 individuals without T2DM reveal that A1C values are higher in Blacks (0.26% (2.8 mmol/mol), *p* < 0.001), Asians (0.24% (2.6 mmol/mol), *p* < 0.001), and Latinos (0.08% (0.9 mmol/mol); *p* < 0.001) when compared to Whites [20]. Moreover, significantly high A1C levels are observed in patients with IDA when compared to healthy subjects (5.51 ± 0.696 vs 4.85 ± 0.461%, *p* < 0.001) and A1C levels decline significantly after treatment with iron supplements in IDA subjects (5.51 ± 0.696 before treatment vs 5.044 ± 0.603 posttreatment; *p* < 0.001) [17].
- The panel opine that the use of A1C as sole criteria for screening of diabetes/prediabetes would be inappropriate in most settings in our country at this time. However, A1C may be utilized for screening if it is being done from a laboratory known to be well equipped with external quality assurance.
- The panel also caution on the concerns of high prevalence of anemia and high prevalence of hemoglobinopathies in certain regions/populations particularly from the North East as these can have significant impact when A1C is used as diagnostic test for screening.

##### *Diagnosis of prediabetes*

- The panel endorse the ADA [4] criteria for diagnosis of prediabetes for Indian context

**Table:** 

Glycemic parameter	Values
FPG	100 to 125 mg/dL
2-h plasma glucose in the 75-g OGTT	140 to 199 mg/dL
A1C	5.7 to 6.4%

##### *Rescreening*

- The panel emphasize on striking balance between cost of screening and cost of treating complications.
- On the basis of expert opinion of the panel, general population should be evaluated for the risk of diabetes by their healthcare provider on annual basis beginning at age 30.
- Yearly or more frequent testing should be considered in individuals if the initial screen test results are in the prediabetes range or present with one or more risk factors that may predispose to development of diabetes.
- The panel opine that screening programs should be linked with healthcare system and ongoing national prevention programs that will facilitate effective and easy identification of people at high risk of developing diabetes and its complications.

##### *Paramedical personnel*

- Paramedical personnel can play a key role as facilitator in imparting basic self-management skills to patients with diabetes and those at risk of diabetes. They can be actively involved in engaging people with diabetes or at risk of diabetes in implementing diet and lifestyle changes, behavioral changes, weight management, prepregnancy counselling, and other preventive education.
- Nurses or other trained workers in primary care settings and in hospital outpatient settings can:
  - Help in identification of people at risk of diabetes
  - Help in recognition of symptoms of diabetes, hypoglycemia, and ketosis
  - Help in timely referral of these cases
- Nurses or nurse educators in secondary and tertiary care settings can:
  - Perform all the above activities
  - Help in prevention and treatment of hypoglycemia
  - Help in problems with insulin use

#### **Evidence**

It has been observed that Indians are more prone to diabetes at a younger age and at a lower BMI compared to their western counterparts [21, 22]. The reason for this difference has been attributed to “Asian Indian phenotype” characterized by low BMI, higher body fat, visceral fat and WC, lower skeletal muscle mass, and profoundly higher rates of IR [23, 24]. The 10-year follow-up data of the CURES that assessed incident rates of dysglycemia in Asian Indians are now available [25]. According to the study, Asian Indians were found to have one of the highest incidence rates of diabetes (diabetes, prediabetes, and any dysglycemia = 22.2, 29.5, and 51.7 per 1000 person-years, respectively), with rapid conversion from normoglycemia to dysglycemia (45.1%). In a cross-sectional study on slum dwellers in Bangalore, prevalence of diabetes and prediabetes was identified as 12.33 and 11.57% in people aged 35 years or above [26]. Moreover, female gender, increasing age, overweight and obesity, sedentary lifestyle, tobacco consumption, and diet habits were strongly associated with prevalence of diabetes and prediabetes. Similarly, in a cross-sectional study in Tamil Nadu, prevalence of diabetes and prediabetes was identified as 10.1 and 8.5%, respectively [27]. Risk factors associated with prediabetes in this study were age of 40 years, male gender, BMI > 23 kg/m^2^, WHR for men > 1 and women > 0.8, alcohol intake, and systolic blood pressure (SBP) > 140 mmHg. Likewise, in a household survey in Punjab using World Health Organization STEP wise Surveillance (WHO STEPS) questionnaire, prevalence of diabetes and prediabetes was identified as 8.3 and 6.3%, respectively [28]. Risk factors that were significantly associated with diabetes were age (45–69 years), marital status, hypertension, obesity, and family history of diabetes. A study on 163 north Indian subjects proposed severity of IR and family history of diabetes as determinants of diminished beta-cell function leading to diabetes in MS [29]. Predictors of progression to dysglycemia were advancing age, family history of diabetes, 2-h plasma glucose, A1C, low and high density lipoprotein (HDL) cholesterol, and physical inactivity. Despite the escalating burden, the current evidence on the prevention of T2DM and its complications in India still remain scanty. Though the general practitioners in India are well aware of symptoms and complications of T2DM, they are oblivious regarding the use of standard screening tests resulting in significant delay in diagnosis and treatment [30]. Considering significant resource constraints together with awareness levels of patients and physicians, there is a need for prevention strategies that are culturally relevant and cost-effective [31]. Following section covers evidence from India studies on various strategies that are helpful in detecting and minimizing the risk of development of diabetes and its associated complications.

- Simplified tools for detection of diabetes such as IDRS developed by MDRF and Diabetes Risk Score for Asian Indians devised by Prof. A. Ramachandran are found to be useful for identifying undiagnosed patients with diabetes in India. Use of these tools could make screening programs more cost-effective [7, 8]. Studies from different regions of India including Jammu and Kashmir, Chennai, Haryana, Delhi, Jabalpur, and Kerala estimated the utility of MDRF-IDRS in identifying risk for diabetes mellitus (DM) and prediabetes in Indian adult population and found statistically significant association between IDRS and DM patients indicating MDRF-IDRS to be efficient tool to screen and diagnose the huge pool of undiagnosed diabetics in India [33–37].
- Other novel non-invasive screening tools such as EZSCAN for detection of prediabetes, diabetes, and microvascular complications [38–40], EZSCAN and SUDOSCAN for chronic kidney diseases (CKD) [41, 42], pedobarography and SUDOSCAN for diabetic peripheral neuropathy [43–47], Michigan Neuropathy Screening Instrument (MNSI), and Optimal Scaling Combination (OSC) for diabetic foot problems [48] have also been evaluated in Asian population with T2DM. However, there are a lot of false positive and false negative results with these non-invasive screening tools and currently the panel does not recommend using these tools for diagnosis of diabetes or prediabetes, in the absence of the gold standard tests based on blood glucose testing outlined above.
- It is also found by some researchers that identifying the presence of multiple risk factors could be used as a simple measure of identifying people at high risk of diabetes [49].
- The panel suggest that individuals with diabetes or at risk of developing diabetes should be advised on lifestyle changes and implementing strategies focusing on diet, exercise, and weight loss to prevent the risk of progression and thus complications of diabetes [50].
- Several landmark studies have shown that lifestyle intervention could prevent the progression to T2DM by about 30–60% [51]. Evidence from literature suggests that initial lifestyle interventions are cost-effective [52] and can significantly reduce the incidence of diabetes in Asian Indians with IGT or with combined IGT + IFG [53, 54]. In patients in whom metformin is contraindicated, AGIs such as acarbose or voglibose may be used, as they confer lesser side effects compared to other OADs. Furthermore, lifestyle intervention with diet and exercise in those with IGT can significantly decrease the incidence of diabetes and its complications [55, 56] while providing long-term beneficial effects for up to 20 years [57]. A systematic review and meta-analysis of 50 trials identified that lifestyle intervention reduced risk of progression to diabetes by 36% over 6 months to 6 years which attenuated to 20% by the time of follow-up results of the trials were measured [15]. Another systematic review and meta-analysis show that physical activity in prediabetes subjects improves oral glucose tolerance, FPG and A1C levels, and maximum oxygen uptake and body composition [58]. Results indicate that physical activity promotion and participation slow down the progression of disease and decrease the morbidity and mortality associated with T2DM. Optimal sleep (7–8 h per night) has been shown to maintain metabolic health, aid in weight loss, and increase insulin sensitivity, while short duration (< 5–6 h) or longer duration (> 8–9 h) of sleep was associated with increased risk of diabetes [59, 60]. Similar results were observed in a systematic review and meta-analysis of 10 articles which determined that the pooled relative risks for T2DM were 1.09 (95% CI = 1.04, 1.15) for each 1-h shorter sleep duration among individuals who slept < 7 h/day and 1.14 (1.03, 1.26) for each 1 h increment of sleep duration among individuals who slept longer, when compared to 7-h sleep duration per day [61].
- Interventions predominantly based on counselling and education are found to be effective in preventing/reducing the risk of developing diabetes and its complication and also helps in improving dietary patterns of individuals with prediabetes and diabetes [31, 62]. Mobile phone messaging was found to be an inexpensive and most effective alternative way to deliver educational and motivational advice and support towards lifestyle modification in high-risk individuals [63].
- Dietary interventions such as high-carbohydrate low-fat diet [64], fiber-rich [65], and protein-rich diet [66, 67] were found to have definite role in prevention of diabetes. Furthermore, components of whole grains, and fruit and green leafy vegetables such as cereal fiber and magnesium, are consistently associated with lower risk of developing T2DM [68].
- Evidence from the CURES and Prevention Awareness Counselling and Evaluation (PACE) diabetes project suggests that awareness and knowledge regarding diabetes is inadequate among patients in India and implementation of educational programs at massive level can greatly improve the awareness on diabetes and its associated CVD [69, 70]. Moreover, mass awareness and screening programs through community empowerment were found to effectively prevent and control diabetes and its complications such as foot amputations [71].
- Currently, the role of yoga and fenugreek in the prevention of diabetes is being evaluated in the Indian prevention of Diabetes Study by RSSDI.

#### **Implementation**

A clear and transparent decision should be made about whether or not to endorse a screening strategy. If the decision is in favor of screening, this should be supported by local protocols and guidelines and public and healthcare professional education campaigns.

**References**

1. Herman WH, Ye W, Griffin SJ, Simmons RK, Davies MJ, Khunti K, Rutten GE, Sandbaek A, Lauritzen T, Borch-Johnsen K, Brown MB. Early detection and treatment of type 2 diabetes reduce cardiovascular morbidity and mortality: a simulation of the results of the Anglo-Danish-Dutch Study of Intensive Treatment in People With Screen-Detected Diabetes in Primary Care (ADDITION-Europe). Diabetes Care. 2015;38(8):1449–55.
2. Unnikrishnan R, Mohan V. Why screening for type 2 diabetes is necessary even in poor resource settings. J Diabetes Complications. 2015;29(7):961–4.
3. Einarson TR, Bereza BG, Acs A, Jensen R. Systematic literature review of the health economic implications of early detection by screening populations at risk for type 2 diabetes. Current medical research and opinion. 2017;33(2):331–58.
4. American Diabetes Association. Promoting health and reducing disparities in populations. Standards of Medical Care in Diabetes 2017. Diabetes Care 2017; 40.
5. Majra JP, Verma R. Opportunistic screening for random blood glucose level among adults attending a rural tertiary care centre in Haryana during world health day observation activity. International Journal of Community Medicine and Public Health. 2017;4(6):1951–6.
6. Venugopal V, Selvaraj K, Majumdar A, Chinnakali P, Roy G. Opportunistic screening for diabetes mellitus among adults attending a primary health center in Puducherry. Int J Med Sci Public Health. 2015;4:1206–1.
7. Ramachandran A, Snehalatha C, Vijay V, Wareham NJ, Colagiuri S. Derivation and validation of diabetes risk score for urban Asian Indians. Diabetes Res Clin Pract. 2005;70(1):63–70.
8. Mohan V, Deepa R, Deepa M, Somannavar S, Datta M. A simplified Indian Diabetes Risk Score for screening for undiagnosed diabetic subjects. J Assoc Physicians India. 2005;53:759–63.
9. Mohan V, Vassy JL, Pradeepa R, Deepa M, Subashini S. The Indian type 2 diabetes risk score also helps identify those at risk of macrovascular disease and neuropathy (CURES-77). J Assoc Physicians India 2010;58:430–3.
10. Priya M, Anjana RM, Chiwanga FS, Gokulakrishnan K, Deepa M, Mohan V. 1-hour venous plasma glucose and incident prediabetes and diabetes in Asian Indians. Diabetes Technol Ther. 2013;15(6):497–502.
11. Inzucchi SE, Bergenstal RM, Buse JB, Diamant M, Ferrannini E, Nauck M, Peters AL, et al. Management of hyperglycemia in type 2 diabetes, 2015: a patient-centered approach: update to a position statement of the American Diabetes Association and the European Association for the Study of Diabetes. Diabetes Care. 2015;38(1):140–9.
12. Bowen ME, Xuan L, Lingvay I, Halm EA. Random blood glucose: a robust risk factor for type 2 diabetes. The Journal of Clinical Endocrinology & Metabolism. 2015;100(4):1503–10.
13. Bowen ME, Xuan L, Lingvay I, Halm EA. Performance of a random glucose case-finding strategy to detect undiagnosed diabetes. American Journal of Preventive Medicine. 2017;52(6):710–6.
14. Elman K, Wainstein J, Boaz M, Jacubovitz D, Bar‐Dayan Y. Random blood glucose screening at a public health station encouraged high risk subjects to make lifestyle changes. International Journal of Clinical Practice. 2017.
15. Barry E, Roberts S, Oke J, et al. Efficacy and effectiveness of screen and treat policies in prevention of type 2 diabetes: systematic review and meta-analysis of screening tests and interventions. BMJ. 2017;356:i6538.
16. Gupta Y, Kapoor D, Desai A, Praveen D, Joshi R, Rozati R et al. Conversion of gestational diabetes mellitus to future type 2 diabetes mellitus and the predictive value of HbA1c in an Indian cohort. Diabet Med. 2017;34(1):37–43.
17. Madhu SV, Raj A, Gupta S, Giri S, Rusia U. Effect of iron deficiency anemia and iron supplementation on A1C levels—implications for diagnosis of prediabetes and diabetes mellitus in Asian Indians. Clinica Chimica Acta. 2017;468:225–9.
18. Hellgren M, Steiner KH, Bennet L. Haemoglobin A1c as a screening tool for type 2 diabetes and prediabetes in populations of Swedish and Middle-East ancestry. Primary Care Diabetes. 2017.
19. Bertran EA, Berlie HD, Taylor A, Divine G, Jaber LA. Diagnostic performance of HbA1c for diabetes in Arab vs. European populations: a systematic review and meta‐analysis. Diabetic Medicine. 2017;34(2):156–66.
20. Cavagnolli G, Pimentel AL, Freitas PA, Gross JL, Camargo JL. Effect of ethnicity on HbA1c levels in individuals without diabetes: systematic review and meta-analysis. PLOS One. 2017;12(2):e0171315.
21. Anjana RM, Pradeepa R, Deepa M, Datta M, Sudha V, Unnikrishnan R, et al., ICMR–INDIAB Collaborative Study Group. Prevalence of diabetes and prediabetes (impaired fasting glucose and/or impaired glucose tolerance) in urban and rural India: phase I results of the Indian Council of Medical Research-INdia DIABetes (ICMR-INDIAB) study. Diabetologia 2011;54(12):3022–7.
22. Ramachandran A, Ma RC, Snehalatha C. Diabetes in Asia. The Lancet. 2010;375(9712):408–18.
23. Ramachandran A, Snehalatha C, Viswanathan V, Viswanathan M, Haffner SM. Risk of noninsulin dependent diabetes mellitus conferred by obesity and central adiposity in different ethnic groups: a comparative analysis between Asian Indians, Mexican Americans and Whites. Diabetes Res Clin Pract 1997;36(2):121–5.
24. Sharp PS, Mohan V, Levy JC, Mather HM, Kohner EM. Insulin resistance in patients of Asian Indian and European origin with non-insulin dependent diabetes. Horm Metab Res 1987;19(2):84–5.
25. Anjana RM, Shanthi Rani CS, Deepa M, Pradeepa R, Sudha V, Divya Nair H, et al. Incidence of diabetes and prediabetes and predictors of progression among Asian Indians: 10-year follow-up of the Chennai Urban Rural Epidemiology Study (CURES). Diabetes Care. 2015;38(8):1441–8.
26. Dasappa H, Fathima FN, Prabhakar R, Sarin S. Prevalence of diabetes and pre-diabetes and assessments of their risk factors in urban slums of Bangalore. Journal of family medicine and primary care. 2015;4(3):399.
27. Muthunarayanan L, Ramraj B, Russel JK. Prevalence of prediabetes and its associated risk factors among rural adults in Tamil Nadu. Archives of Medicine and Health Sciences. 2015;3(2):178.
28. Tripathy JP, Thakur JS, Jeet G, Chawla S, Jain S, Pal A, Prasad R, Saran R. Prevalence and risk factors of diabetes in a large community-based study in North India: results from a STEPS survey in Punjab, India. Diabetology & Metabolic Syndrome. 2017;9(1):8.
29. Pratyush DD, Tiwari S, Singh S, Singh SK. Risk factors of diabetes in North Indians with metabolic syndrome. Diabetes & Metabolic Syndrome: Clinical Research & Reviews. 2016;10(2):S68–71.
30. Patel N, Deshpande S, Godbole V, Champaneri V, Singh N. Awareness and approach towards diagnosis and treatment of diabetes type 2 and its complication among general practioners of western Vadodara. Int J Diabetes Dev Ctries. 2014.
31. Rawal LB, Tapp RJ, Williams ED, Chan C, Yasin S, Oldenburg B. Prevention of type 2 diabetes and its complications in developing countries: a review. Int J Behav Med. 2012;19(2):121–33.
32. Gupta RK, Shora TN, Verma AK, Raina SK. Utility of MDRF-IDRS (Madras Diabetes Research Foundation-Indian Diabetes Risk Score) as a tool to assess risk for diabetes—a study from north-west India. International Journal of Diabetes in Developing Countries. 2015 (to be published) Available on: http://link.springer.com/article/10.1007/s13410-015-0346-8 (Last accessed on 27 Aug 2015).
33. Nagalingam S, Sundaramoorthy K, Arumugam B. Screening for diabetes using Indian diabetes risk score. International Journal of Advances in Medicine. 2017;3(2):415–8.
34. Rajput M, Garg D, Rajput R. Validation of simplified Indian diabetes risk scores for screening undiagnosed diabetes in an urban setting of haryana. Diabetes & Metabolic Syndrome: Clinical Research & Reviews. 2017.
35. Acharya AS, Singh A, Dhiman B. Assessment of diabetes risk in an adult population using Indian Diabetes Risk Score in an urban resettlement colony of Delhi. Journal of the Association of Physicians of India. 2017;65:46.
36. Bhadoria AS, Kasar PK, Toppo NA. Validation of Indian diabetic risk score in diagnosing type 2 diabetes mellitus against high fasting blood sugar levels among adult population of central India. Biomed J. 2015;38:359–60.
37. Sathish T, Oldenburg B, Tapp RJ, Shaw JE, Wolfe R, Sajitha B, D’Esposito F, Absetz P, Mathews E, Zimmet PZ, Thankappan KR. Baseline characteristics of participants in the Kerala Diabetes Prevention Program: a cluster randomized controlled trial of lifestyle intervention in Asian Indians. Diabetic Medicine. 2016.
38. Ramachandran A, Moses A, Shetty S, Thirupurasundari CJ, Seeli AC, Snehalatha C, et al. A new non-invasive technology to screen for dysglycaemia including diabetes. Diabetes Res Clin Pract. 2010;88(3):302–6.
39. Bajaj S, Pandey RK, Chaurasia AK, Shukla RP. Use of EZSCAN for detection of pre-diabetes and diabetes and comparison with standard screening methods. Sri Lanka Journal of Diabetes Endocrinology and Metabolism. 2015;5(1).
40. Bajaj S, Tiwari A, Chaurasia AK, Shukla RP. Detection of microvascular complications of type 2 diabetes by EZSCAN and its comparison with standard screening methods. J. Evid. Based Med. Healthc. 2016;3(66):3579–83.
41. Ozaki R, Cheung KK, Wu E, Kong A, Yang X, Lau E, et al. A new tool to detect kidney disease in Chinese type 2 diabetes patients: comparison of EZSCAN with standard screening methods. Diabetes Technol Ther. 2011;13(9):937–43.
42. Luk AO, Fu WC, Li X, Ozaki R, Chung HH, Wong RY, So WY, Chow FC, Chan JC. The clinical utility of SUDOSCAN in chronic kidney disease in Chinese patients with type 2 diabetes. PLOS One. 2015;10(8):e0134981.
43. Skopljak A, Muftic M, Sukalo A, Masic I, Zunic L. Pedobarography in diagnosis and clinical application. Acta Informatica Medica. 2014;22(6):374.
44. Fawzy OA, Arafa AI, El Wakeel MA, Kareem SH. Plantar pressure as a risk assessment tool for diabetic foot ulceration in Egyptian patients with diabetes. Clinical medicine insights. Endocrinology and diabetes. 2014;7:31.
45. Yajnik CS, Kantikar VV, Pande AJ, Deslypere JP. Quick and simple evaluation of sudomotor function for screening of diabetic neuropathy. ISRN endocrinology. 2012.
46. Mao F, Liu S, Qiao X, Zheng H, Xiong Q, Wen J, Liu L, Tang M, Zhang S, Zhang Z, Ye H. Sudoscan is an effective screening method for asymptomatic diabetic neuropathy in Chinese type 2 diabetes mellitus patients. Journal of Diabetes Investigation. 2016.
47. Fang F, Wang YF, Gu MY, Chen H, Wang DM, Xiao K, et al. Pedobarography—a novel screening tool for diabetic peripheral neuropathy? Eur Rev Med Pharmacol Sci. 2013;17(23):3206–12.
48. Chang CH, Peng YS, Chang CC, Chen MY. Useful screening tools for preventing foot problems of diabetics in rural areas: a cross-sectional study. BMC Public Health. 2013;13:612.
49. Priscilla S, Nanditha A, Simon M et al. A pragmatic and scalable strategy using mobile technology to promote sustained lifestyle changes to prevent type 2 diabetes in India—outcome of screening. Diabetes Res Clin Pract. 2015;110:335–40.
50. Chaturvedi N. The burden of diabetes and its complications: trends and implications for intervention. Diabetes Res Clin Pract. 2007;76 Suppl 1:S3–12.
51. Edwardson CL, Gray LJ, Yates T, Barber SR, Khunti K, Davies MJ. Detection and early lifestyle intervention in those at risk of type 2 diabetes. Eur Med J2014;2:48–57.
52. Ramachandran A, Snehalatha C, Yamuna A, Mary S, Ping Z. Cost-effectiveness of the interventions in the primary prevention of diabetes among Asian Indians: within-trial results of the Indian Diabetes Prevention Programme (IDPP). Diabetes Care. 2007;30(10):2548–52.
53. Ramachandran A, Snehalatha C, Mary S, Mukesh B, Bhaskar AD, Vijay V; Indian Diabetes Prevention Programme (IDPP). The Indian Diabetes Prevention Programme shows that lifestyle modification and metformin prevent type 2 diabetes in Asian Indian subjects with impaired glucose tolerance (IDPP-1). Diabetologia. 2006;49(2):289–97.
54. R3amachandran A, Arun N, Shetty AS, Snehalatha C. Efficacy of primary prevention interventions when fasting and postglucose dysglycemia coexist: analysis of the Indian Diabetes Prevention Programmes (IDPP-1 and IDPP-2). Diabetes Care. 2010;33(10):2164–8.
55. Pan XR, Li GW, Hu YH, Wang JX, Yang WY, An ZX, et al. Effects of diet and exercise in preventing NIDDM in people with impaired glucose tolerance. The Da Qing IGT and Diabetes Study. Diabetes Care. 1997;20(4):537–44.
56. Kosaka K, Noda M, Kuzuya T. Prevention of type 2 diabetes by lifestyle intervention: a Japanese trial in IGT males. Diabetes Res Clin Pract. 2005;67(2):152–62.
57. Li G, Zhang P, Wang J, Gregg EW, Yang W, Gong Q, et al. The long-term effect of lifestyle interventions to prevent diabetes in the China Da Qing Diabetes Prevention Study: a 20-year follow-up study. Lancet. 2008;371(9626):1783–9.
58. Jadhav RA, Hazari A, Monterio A, Kumar S, Maiya AG. Effect of physical activity intervention in prediabetes: a systematic review with meta-analysis. Journal of Physical Activity and Health. 2017.
59. Cappuccio FP, D’Elia L, Strazzullo P, Miller MA. Quantity and quality of sleep and incidence of type 2 diabetes: a systematic review and meta-analysis. Diabetes Care. 2010;33(2):414–20.
60. Cedernaes J, Schiöth HB, Benedict C. Determinants of shortened, disrupted, and mistimed sleep and associated metabolic health consequences in healthy humans. Diabetes. 2015;64(4):1073–80.
61. Shan Z, Ma H, Xie M, Yan P, Guo Y, Bao W, Rong Y, Jackson CL, Hu FB, Liu L. Sleep duration and risk of type 2 diabetes: a meta-analysis of prospective studies. Diabetes care. 2015;38(3):529–37.
62. Balagopal P, Kamalamma N, Patel TG, Misra R. A community-based diabetes prevention and management education program in a rural village in India. Diabetes Care. 2008;31(6):1097–104.
63. Ramachandran A, Snehalatha C, Ram J, Selvam S, Simon M, Nanditha A, et al. Effectiveness of mobile phone messaging in prevention of type 2 diabetes by lifestyle modification in men in India: a prospective, parallel-group, randomised controlled trial. Lancet Diabetes Endocrinol. 2013;1(3):191–8.
64. Ahuja MMS. North Indian food practice and dietary fibre. Int J Diabetes Dev Ctries. 1992;12:121
65. Trivedi B, Maniyar KT, Patel B. Effect of fibre diet (guar) on cholesterol, blood glucose and body weight. Int J Diabetes Dev Ctries. 1999;19;31–3.
66. Modi SV, Borges VJ, Chandalia HB. Low carbohydrate or high carbohydrate: what is right? Review. Int J Diabetes Dev Ctries. 2005;25;58–62.
67. Sekar V, Sundaram A, Lakshmi B, Kalaivani AR, Mala S, Banupriya M et al. The effect of modified pulse-carbohydrate diet on weight and HbA1c in type 2 diabetic patients. Int J Diabetes Dev Ctries. 2006;25;16–18.
68. Franz MJ, Zhang Z, Venn BJ. Lifestyle interventions to stem the tide of type 2 diabetes. In Nutrition Guide for Physicians and Related Healthcare Professionals 2017 (pp. 103–112). Springer International Publishing.
69. Mohan D, Raj D, Shanthirani CS, Datta M, Unwin NC, Kapur A, et al. Awareness and knowledge of diabetes in Chennai—the Chennai Urban Rural Epidemiology Study [CURES-9]. J Assoc Physicians India. 2005;53:283–7.
70. Somannavar S, Lanthorn H, Deepa M, Pradeepa R, Rema M, Mohan V. Increased awareness about diabetes and its complications in a whole city: effectiveness of the “prevention, awareness, counselling and evaluation” [PACE] Diabetes Project [PACE-6]. J Assoc Physicians India. 2008;56:497–502.
71. Somannavar S, Lanthorn H, Pradeepa R, Narayanan V, Rema M, Mohan V. Prevention awareness counselling and evaluation (PACE) diabetes project: a mega multi-pronged program for diabetes awareness and prevention in South India (PACE- 5). J Assoc Physicians India. 2008;56:429–35.

## **Obesity and diabetes**

### **RSSDI 2017 recommendations**

#### **Recommended care**

- The cut-off points for overweight and obesity in Indian T2DM patients are as follows:
  - BMI 18–22.9 kg/m^2^: normal
  - BMI 23–24.9 kg/m^2^: overweight
  - BMI ≥ 25 kg/m^2^: generalized obesity
  - WC ≥ 90 cm for men and ≥ 80 cm for women: abdominal obesity (AO)
- Maintaining healthy lifestyle is recommended for management of metabolic syndrome. This includes:
  - Moderate calorie restriction (to achieve a 5–10% loss in body weight)
  - Increase in physical activity; up to 150 min/week of moderate to vigorous intensity physical activity
  - Change in dietary composition (low-calorie diet)
  - Combination of aerobic and resistance training exercise
  - Change in behavioral pattern
- Overweight and obese people with T2DM should be initiated on exercise therapy, prescribing a combination of aerobic and muscle strengthening activities
- Pharmacotherapy for obese patients with T2DM should be considered in addition to lifestyle changes in those with BMI > 27 kg/m^2^ without comorbidity or a BMI > 25 kg/m^2^ with comorbidity
  - Metformin should be first-line drug for all T2DM patients
  - Glucagon-like peptide (GLP)-1 analogues, dipeptidyl peptidase (DPP) 4 inhibitors, and sodium-glucose cotransporter (SGLT) 2 inhibitors may be preferred as add-ons to metformin in obese T2DM patients
- Lipase inhibitors (orlistat) may be used for inducing weight loss
- Surgical treatment (bariatric surgery) is indicated in patients with BMI > 32.5 kg/m^2^ with comorbidity and BMI > 37.5 kg/m^2^ without comorbidity
- Surgical options for weight loss surgery include:
  - Restrictive procedures: laparoscopic adjustable gastric banding (LAGB) and sleeve gastrectomy
  - Malabsorptive procedures: bilio-pancreatic diversions (BPD)
  - Combined procedures: Roux-en-Y gastric bypass (RYGB)
  - Experimental procedures: illeal interposition and duodeno-jejunal bypass, various implantable pulse generator
- Comprehensive lifestyle changes including dietary modification, exercise, behavioral management, pharmacotherapy and bariatric surgery are the most effective interventions for weight management in T2DM patients

#### **Preamble**

Obesity is a highly prevalent metabolic disorder that is often associated with T2DM [1, 2]. Obesity is clinically defined as a BMI of ≥ 30 kg/m^2^ (a BMI of 30 represents an overweight of approximately 30 lb. (14 kg) for any given height) [3]. However, WHO and International Obesity Task Force (IOTF) suggested BMI cut-offs of 23 and 25 kg/m^2^ for Asian Indian adults for overweight and obesity, respectively [4, 5]. Furthermore, the guidelines defined generalized obesity (GO, BMI ≥ 25 kg/m^2^), AO (WC ≥ 90 cm for men and ≥ 80 cm for women), and combined obesity (CO, GO plus AO) for Asian population [5]. In India, the prevalence of obesity is rising at an alarming rate, especially affecting urban population [2, 6]. The Indian Council of Medical Research-INdia DIABetes (ICMR-INDIAB) study currently report that 135, 153, and 107 million individuals in India will have GO, AO, and CO, respectively, in extrapolation to the whole country. Furthermore, female gender, hypertension, diabetes, higher socioeconomic status, physical inactivity, and urban residence were significantly associated with GO, AO, and CO, in Indian populations [7]. Indians are at increased predisposition to diabetes that has been attributed to the “Asian Indian Phenotype” characterized by lesser GO as measured by BMI and greater central body obesity and more truncal fat as shown by greater WC and WHR [2, 8–11]. High AO contributes significantly to metabolic alterations such as IR, dysglycemia, and dyslipidemia [12–16, 17]. Obesity-induced IR may cause T2DM by increasing the allostatic load on the pancreas which eventually leads to failure of pancreas. High consumption of sugars among children and adults in India may also have clinical significance in view of the high tendency for Indians to develop IR, abdominal adiposity, and hepatic steatosis and the increasing “epidemic” of T2DM [2, 18]. Because Asian Indians tend to develop diabetes at a significantly lower BMI and WC than white Europeans, lower thresholds of BMI to define overweight (BMI = 23–24.9 kg/m^2^) and obesity (BMI ≥ 25 kg/m^2^) were proposed by IDF and National Institute of Health and Care Excellence (NICE) [19, 20].

In light of increasing prevalence of obesity in both developed and developing countries and a higher risk for developing IR, dyslipidemia, dysglycemia, and a higher CV risk at lower levels of BMI in Indians, a consensus meeting was convened in New Delhi in 2008 (published in 2009) to redefine the cut-offs for BMI and WC for diagnosing overweight and obesity in Indian population [21, 22]. According to this consensus statement, a BMI of 18–22.9 kg/m^2^ should be considered as normal, a BMI of 23–24.9 kg/m^2^ should be considered as overweight, and BMI more than 25 kg/m^2^ indicates presence of obesity. The upper limit for WC for men and women was defined as 90 cm and 80 cm, respectively.

#### **Considerations**

The following local factors were considered when framing recommendations for obesity that were reviewed in Indian context: high prevalence of obesity, high abdominal adiposity, increased fasting insulin and IR, nutritional factors, and atherogenic lipid profile [increased triglycerides and low density lipoprotein (LDL) and low HDL].

#### **Rationale and evidence**

##### *Identification*

- Indian Diabetes Risk Score is a simple technique for screening of diabetes, which uses four risk factors: age, AO, family history of diabetes, and physical activity. Several studies have highlighted the importance of IDRS in the screening of diabetes in Indian population [23–26]. In a cross-sectional study comparison of IDRS and Framingham Risk Score (FRS) by obesity and lipid abnormality status in women of Asian Indian origin hinted that IDRS can predict CV and diabetes risk more effectively than FRS and serve as simple and cost-effective tool for a primary care physician to identify at risk individuals for diabetes and cardiovascular diseases [27].

##### *Lifestyle intervention: behavioral therapy, exercise, and therapy*

- Lifestyle interventions including diet therapy, physical activity, and behavioral and psychosocial strategies have shown positive health outcomes in obese T2DM patients [28]. The Diabetes Prevention Program (DPP) [29] and the Look AHEAD (Action for Health in Diabetes) trial [30] report clinically significant weight losses averaging 4 to 5% (or 4–5 kg) at 3–4 years. Similarly, a randomized controlled trial (RCT) including Asian Indians report that lifestyle intervention with less education lost a model-predicted 3.30 kg more in weight and 4.95 cm more in WC than those with more formal education [31]. Moreover, a population-based cross-sectional study with 15,145 participants report that an additive interaction exists between poor sleep quality, AO, and family history of diabetes in relation to IFG [32].
- The diet therapy for obese T2DM patients should be based on the criteria of decreased energy intake and increased energy expenditure to produce the negative energy balance. This includes low-calorie diet and diet with caloric restriction and with varying combination of macronutrients [33].
- Behavioral therapy includes modifiable factors such as eating patterns and exercise habits that can have significant impact on the management of obesity. A review in Indian scenario suggested that slow eating techniques along with stimulus control (not distracted by television, books, or other materials) have positive effect on weight loss [33]. In obese T2DM patients, IDF recommends to maintain healthy lifestyle through behavioral therapy that includes moderate calorie restriction to promote weight loss (5–10% loss of body weight in the first year) [34], moderate increase in physical activity, and change in dietary composition. Other important components of behavioral therapy embrace self-monitoring, goal setting, and stimulus or cue control. Such strategies help in setting up realistic goals, guide patients in identifying stimulus that lead to excessive nutrient intake, and eliminate them accordingly [35].
- A recent systematic review and meta-analysis report that diet and physical exercise resulted in significant improvement of body weight in south Asian adults but had no effect on BMI and WC. Furthermore, no alteration in these parameters was observed in south Asian children [36].
- Body weight has been shown to be inversely associated with physical activity [37]. Subjects with low physical activity have threefold greater risk of major weight gain in men and almost a fourfold in women [38, 39]. Moreover, this association was stronger for women than for men and for obese compared to normal weight or overweight individuals [40]. Furthermore, slow and prolonged exercise is associated with fatty acid oxidation with beneficial effects on body weight [41, 42]. RCT comprising 262 sedentary men and women report that the combination of aerobic and resistance training exercise reduced WC from − 1.9 to − 2.8 cm and mean fat mass of − 1.7 (− 2.3 to − 1.1 kg; *p* < 0.05) compared with the non-exercise group [43].
- Therefore, the panel opined that prescribing a combination of aerobic and resistance training exercises in individuals with T2DM can improve metabolic control while reducing obesity and its related complications.

##### *Pharmacotherapy for obese T2DM*

- Though lifestyle modifications are effective in preventing diabetes relapse or remission [44], they often fail requiring initiation of pharmacotherapy. Metformin is the first choice drug with some evidence for weight loss [45, 46]. The DPP and Indian Diabetes Prevention Program (IDPP) trial report that metformin and lifestyle intervention greatly reduce the risk of T2DM in overweight or obese patients [47, 48]. Use of GLP-1 analogues [49], SGLT-2 inhibitors [50, 51], and to some extent DPP-4 inhibitors [52, 53] has been shown to induce weight loss and should be considered as add-on to metformin in obese T2DM patients. Furthermore, GLOBE study report that fixed dose combination (FDC) of acarbose/metformin in Indian T2DM patients was associated with significant reduction in body weight (− 1.7 ± 2.2 kg compared to control (*p* < 0.0001)) [54].
- Orlistat (tetrahydrolipstatin), a lipase inhibitor, is the only approved agent for weight loss in India. It causes modest weight loss by blocking fat absorption from gut and when used in combination with lifestyle changes was found to be effective in prevention of diabetes [10, 55]. Furthermore, a recent systematic review and meta-analysis report that treatment with orlistat and lifestyle intervention resulted in significantly greater weight loss (*p* < 0.001) and improved glycemic control (*p* < 0.001) in overweight and obese T2DM patients compared with lifestyle intervention alone [56].

##### *Surgery*

- The surgical options for weight loss include LAGB and sleeve gastrectomy, RYGB, BPD, illeal interposition and duodenojejunal bypass, and various implantable pulse generators [21].
- Surgical treatment (bariatric surgery) is indicated in patients with BMI > 32.5 kg/m^2^ with comorbidity or BMI > 37.5 kg/m^2^ without comorbidity and who fail to lose weight with medical management [21], although hard evidence for this is lacking. Evidence from several studies suggests that bariatric surgery provides durable glycemic control compared with intensive medical therapy [57–60]. Moreover, gastric bypass has been observed to uniquely restore the pancreatic β-cell function and reduce truncal fat, thus reversing the core defects in diabetes [57]. In addition, a systematic review and meta-analysis of RCTs report that RYGB surgery is superior to medical treatment for short- to medium-term remission of T2DM and improvement of metabolic condition and CV risk factors [61].
- Bariatric surgery is an effective option for severely obese patients with poorly controlled T2DM and weight loss due to gastric bypass surgery is associated with good glycemic control [62]. In patients who had undergone bariatric surgery, about 8% showed complete remission of diabetes while more than 90% showed a significant decrease in their insulin or OADs requirement [62].
- Laparoscopic sleeve surgery and RYBG were found to be safe and effective treatment options among obese Indian population with T2DM with significant remission rates (> 95%, *p* < 0.001), larger reductions in A1C, and diabetes medication usage [63–65].

**References**

1. Haslam DW, James WP. Obesity. Lancet. 2005;366(9492):1197–209.
2. Gulati S, Misra A. Abdominal obesity and type 2 diabetes in Asian Indians: dietary strategies including edible oils, cooking practices and sugar intake. European Journal of Clinical Nutrition. 2017;71(7):850–7.
3. National Heart, Lung and Blood Institute. Clinical practice guidelines. Available at: https://www.nhlbi.nih.gov/research/resources/obesity/education/clinical-guidelines.htm (last accessed date: 18 Aug 2015)
4. Ranjani H, Mehreen TS, Pradeepa R, Anjana RM, Garg R, Anand K, Mohan V. Epidemiology of childhood overweight & obesity in India: a systematic review. The Indian Journal of Medical Research. 2016;143(2):160.
5. World Health Organization Western Pacific Region, International Association for the Study of Obesity, International Obesity Task Force. Asia Pacific perspective: Redefining 171 obesity and its treatment. Australia: Health Communications Australia; 2000.
6. Misra A, Khurana L. Obesity and the metabolic syndrome in developing countries. J Clin Endocrinol Metab. 2008;93(11 Suppl 1):S9–30.
7. Pradeepa R, Anjana RM, Joshi SR, Bhansali A, Deepa M, Joshi PP, Dhandania VK, Madhu SV, Rao PV, Geetha L, Subashini R. Prevalence of generalized & abdominal obesity in urban & rural India-the ICMR-INDIAB Study (Phase-I) [ICMR-INDIAB-3]. Indian Journal of Medical Research. 2015;142(2):139.
8. Pandya H, Lakhani JD, Patel N. Obesity is becoming synonym for diabetes in rural areas of India also—an alarming situation. Int J Biol Med Res. 2011;2(2):556–60.
9. Ramachandran A, Snehalatha C, Viswanathan V, Viswanathan M, Haffner SM. Risk of noninsulin dependent diabetes mellitus conferred by obesity and central adiposity in different ethnic groups: a comparative analysis between Asian Indians, Mexican Americans and Whites. Diabetes Res Clin Pract 1997;36(2):121–5.
10. Misra A, Khurana L. The metabolic syndrome in South Asians: epidemiology, determinants, and prevention. Metab Syndr Relat Disord. 2009;7(6):497–514.
11. Misra A, Vikram NK, Gupta R, Pandey RM, Wasir JS, Gupta VP. Waist circumference cutoff points and action levels for Asian Indians for identification of abdominal obesity. Int J Obes (Lond). 2006;30(1):106–11.
12. Mohan V, Sharp PS, Cloke HR, Burrin JM, Schumer B, Kohner EM. Serum immunoreactive insulin responses to a glucose load in Asian Indian and European type 2 (non-insulin-dependent) diabetic patients and control subjects. Diabetologia 1986;29(4):235–7.
13. Sharp PS, Mohan V, Levy JC, Mather HM, Kohner EM. Insulin resistance in patients of Asian Indian and European origin with non-insulin dependent diabetes. Horm Metab Res 1987;19(2):84–5.
14. Misra A and Khurana L. Obesity and the metabolic syndrome in developing countries. J Clin Endocrinol Metab. 2008;93(11 Suppl 1):S9–30.
15. Snehalatha C, Viswanathan V, Ramachandran A. Cutoff values for normal anthropometric variables in Asian Indian adults. Diabetes Care. 2003;26(5):1380–4.
16. Ramachandran A. Diabetes & obesity—the Indian angle. Indian J Med Res. 2004;120(5):437–9.
17. Kaur J. A comprehensive review on metabolic syndrome. Cardiology research and practice. 2014.
18. The IDF consensus worldwide definition of the Metabolic Syndrome. 2006. Available on: https://www.idf.org/webdata/docs/IDF_Meta_def_final.pdf (Last accessed on 27 Aug 2015)
19. NICE.PH46 Assessing body mass index and waist circumference thresholds for intervening to prevent ill health and premature death among adults from black, Asian and other minority ethnic groups in the UK 2013. Available at: http://www.nice.org.uk/guidance/PH46 (Last accessed on 27 Aug 2015)
20. Gulati S, Misra A. Sugar intake, obesity, and diabetes in India. Nutrients. 2014;6(12):5955–74.
21. Misra A, Chowbey P, Makkar BM, Vikram NK, Wasir JS, Chadha D, et al. Consensus statement for diagnosis of obesity, abdominal obesity and the metabolic syndrome for Asian Indians and recommendations for physical activity, medical and surgical management. J Assoc Physicians India. 2009;57:163–70.
22. Misra A, Shrivastava U. Obesity and dyslipidemia in South Asians. Nutrients. 2013;5(7):2708–33.
23. Nagalingam S, Sundaramoorthy K, Arumugam B. Screening for diabetes using Indian diabetes risk score. International Journal of Advances in Medicine. 2017;3(2):415–8.
24. Rajput M, Garg D, Rajput R. Validation of simplified Indian diabetes risk scores for screening undiagnosed diabetes in an urban setting of haryana. Diabetes & Metabolic Syndrome: Clinical Research & Reviews. 2017.
25. Acharya AS, Singh A, Dhiman B. Assessment of diabetes risk in an adult population using Indian Diabetes Risk Score in an urban resettlement colony of Delhi. Journal of the Association of Physicians of India. 2017;65:46.
26. Bhadoria AS, Kasar PK, Toppo NA. Validation of Indian diabetic risk score in diagnosing type 2 diabetes mellitus against high fasting blood sugar levels among adult population of central India. Biomed J. 2015;38:359–60.
27. Bhagat M, Ghosh A. Comparison of Framingham Risk Score and Indian diabetes risk score by obesity status and lipids abnormality in women of Asian Indian origin: Santiniketan women study. Int J Diabetes Dev Ctries. 2011;31(2):123–4.
28. Hagobian TA, Phelan S. Lifestyle interventions to reduce obesity and diabetes. American Journal of Lifestyle Medicine. 2013;7(2):84–98.
29. Fujimoto WY, Jablonski KA, Bray GA, Kriska A, Barrett-Connor E, Haffner S, Hanson R, Hill JO, Hubbard V, Stamm E, Pi-Sunyer FX. Body size and shape changes and the risk of diabetes in the diabetes prevention program. Diabetes. 2007;56(6):1680–5.
30. Wing RR et al. Look AHEAD Research Group. Long term effects of a lifestyle intervention on weight and CV risk factors in individuals with type 2 diabetes: four year results of the Look AHEAD trial. Archives of internal medicine. 2010;170(17):1566.
31. Gurka MJ, Wolf AM, Conaway MR, Crowther JQ, Nadler JL, Bovbjerg VE. Lifestyle intervention in obese patients with type 2 diabetes: impact of the patient’s educational background. Obesity. 2006;14(6):1085–92.
32. Qin Y, Lou P, Chen P, Zhang L, Zhang P, Chang G, Zhang N, Li T, Qiao C. Interaction of poor sleep quality, family history of type 2 diabetes, and abdominal obesity on impaired fasting glucose: a population-based cross-sectional survey in China. International Journal of Diabetes in Developing Countries. 2016;36(3):277–82.
33. Sahu R.K, Prashar D. Current treatment strategies for obesity including Indian scenario. Asian Journal of Pharmaceutics (AJP): Free full text articles from Asian J Pharm. 2016;10(03).
34. Pascale RW, Wing RR, Butler BA, Mullen M, Bononi P. Effects of a behavioural weight loss program stressing calorie restriction versus calorie plus fat restriction in obese individuals with NIDDM or a family history of diabetes. Diabetes Care. 1995;18(9):1241–8.
35. Avenell A, Broom J, Brown TJ, Poobalan A, Aucott L, Stearns SC, et al. Systematic review of the long-term effects and economic consequences of treatments for obesity and implications for health improvement. Health Technol Assess. 2004;8(21):iii-iv,1–182.
36. Brown T, Smith S, Bhopal R, Kasim A, Summerbell C. Diet and physical activity interventions to prevent or treat obesity in South Asian children and adults: a systematic review and meta-analysis. Int J Environ Res Public Health. 2015;12(1):566–94.
37. Bowen L, Taylor AE, Sullivan R, Ebrahim S, Kinra S, Krishna KR, Kulkarni B, Ben-Shlomo Y, Ekelund U, Wells JC, Kuper H. Associations between diet, physical activity and body fat distribution: a cross sectional study in an Indian population. BMC Public Health. 2015;15(1):281.
38. Williamson DF, Madans J, Anda RF, Kleinman JC, Kahn HS, Byers T. Recreational physical activity and ten-year weight change in a US national cohort. Int J Obes Relat Metab Disord. 1993;17(5):279–86.
39. Swift DL, Johannsen NM, Lavie CJ, Earnest CP, Church TS. The role of exercise and physical activity in weight loss and maintenance. Progress in Cardiovascular Diseases. 2014;56(4):441–7.
40. Littman AJ, Kristal AR, White E. Effects of physical activity intensity, frequency, and activity type on 10-y weight change in middle-aged men and women. International Journal of Obesity. 2005;29(5):524.
41. Melanson EL, MacLean PS, Hill JO. Exercise improves fat metabolism in muscle but does not increase 24-h fat oxidation. Exercise and Sport Sciences Reviews. 2009;37(2):93.
42. Ballor DL, Smith DB, Tommerup LJ, Thomas DP. Neither high- nor low-intensity exercise promotes whole-body conservation of protein during severe dietary restrictions. Int J Obes. 1990;14(3):279–87.
43. Church TS, Blair SN, Cocreham S, Johannsen N, Johnson W, Kramer K, Mikus CR, Myers V, Nauta M, Rodarte RQ, Sparks L. Effects of aerobic and resistance training on hemoglobin A1c levels in patients with type 2 diabetes: a randomized controlled trial. JAMA. 2010;304(20):2253–62.
44. Mottalib A, Sakr M, Shehabeldin M, Hamdy O. Diabetes remission after nonsurgical intensive lifestyle intervention in obese patients with type 2 diabetes. Journal of Diabetes Research. 2015.
45. Lee A, Morley JE. Metformin decreases food consumption and induces weight loss in subjects with obesity with type II non-insulin-dependent diabetes. Obes Res. 1998;6(1):47–53.
46. Hendricks EJ. Off-label drugs for weight management. Diabetes, Metabolic Syndrome and Obesity: Targets and Therapy. 2017;10:223.
47. Bray GA et al. Diabetes Prevention Program Research Group. Long-term safety, tolerability, and weight loss associated with metformin in the Diabetes Prevention Program Outcomes Study. Diabetes care. 2012;35(4):731–7.
48. Ramachandran A, Snehalatha C, Mary S, Mukesh B, Bhaskar AD, Vijay V. The Indian Diabetes Prevention Programme shows that lifestyle modification and metformin prevent type 2 diabetes in Asian Indian subjects with impaired glucose tolerance (IDPP-1). Diabetologia. 2006;49(2):289–97.
49. Potts JE, Gray LJ, Brady EM, Khunti K, Davies MJ, Bodicoat DH. The Effect of glucagon-like peptide 1 receptor agonists on weight loss in type 2 diabetes: a systematic review and mixed treatment comparison meta-analysis. PLOS One. 2015;10(6):e0126769.
50. Monami M, Nardini C, Mannucci E. Efficacy and safety of sodium glucose co-transport-2 inhibitors in type 2 diabetes: a meta-analysis of randomized clinical trials. Diabetes Obes Metab. 2014;16(5):457–66.
51. Gupta S, Shaikh S, Joshi P, Bhure S, Suvarna V. Long-term efficacy and safety of empagliflozin monotherapy in drug-naïve patients with type 2 diabetes in Indian subgroup: results from a 76-week extension trial of phase III, double-blind, randomized study. Indian journal of endocrinology and metabolism. 2017;21(2):286.
52. Kodera R, Shikata K, Nakamura A, Okazaki S, Nagase R, Nakatou T, Haisa S, Hida K, Miyashita K, Makino H. The glucose-lowering efficacy of sitagliptin in obese Japanese patients with type 2 diabetes. Internal Medicine. 2017;56(6):605–13.
53. Chen JF, Chang CM, Kuo MC, Tung SC, Tsao CF, Tsai CJ. Impact of baseline body mass index status on glucose lowering and weight change during sitagliptin treatment for type 2 diabetics. Diabetes Research and Clinical Practice. 2016;120:8–14.
54. Saboo B, Reddy GC, Juneja S, Kedia AK, Manjrekar P, Rathod R, behalf of GLOBE Investigators. Effectiveness and safety of fixed dose combination of acarbose/metformin in Indian type 2 diabetes patients: results from observational GLOBE Study. Indian Journal of Endocrinology and Metabolism. 2015;19(1):129.
55. Torgerson JS, Hauptman J, Boldrin MN, Sjöström L. XENical in the prevention of diabetes in obese subjects (XENDOS) study: a randomized study of orlistat as an adjunct to lifestyle changes for the prevention of type 2 diabetes in obese patients. Diabetes Care. 2004;27(1):155–61.
56. Aldekhail NM, Logue J, McLoone P, Morrison DS. Effect of orlistat on glycaemic control in overweight and obese patients with type 2 diabetes mellitus: a systematic review and meta‐analysis of randomized controlled trials. Obesity Reviews. 2015;16(12):1071–80.
57. Kashyap SR, Bhatt DL, Wolski K, Watanabe RM, Abdul-Ghani M, Abood B, et al. Metabolic effects of bariatric surgery in patients with moderate obesity and type 2 diabetes: analysis of a randomized control trial comparing surgery with intensive medical treatment. Diabetes Care. 2013;36(8):2175–82.
58. Schauer PR, Bhatt DL, Kirwan JP, Wolski K, Brethauer SA, Navaneethan SDet al; STAMPEDE Investigators. Bariatric surgery versus intensive medical therapy for diabetes—3-year outcomes. N Engl J Med. 2014;370(21):2002–13.
59. Schauer PR, Kashyap SR, Wolski K, Brethauer SA, Kirwan JP, Pothier CE, et al. Bariatric surgery versus intensive medical therapy in obese patients with diabetes. N Engl J Med. 2012; 366(17):1567–76.
60. Schauer PR, Bhatt DL, Kirwan JP, Wolski K, Aminian A, Brethauer SA, Navaneethan SD, Singh RP, Pothier CE, Nissen SE, Kashyap SR. Bariatric surgery versus intensive medical therapy for diabetes—5-year outcomes. New England Journal of Medicine. 2017;376(7):641–51.
61. Yan Y, Sha Y, Yao G, Wang S, Kong F, Liu H, Zhang G, Zhang H, Hu C, Zhang X. Roux-en-Y gastric bypass versus medical treatment for type 2 diabetes mellitus in obese patients: a systematic review and meta-analysis of randomized controlled trials. Medicine. 2016;95(17).
62. Singhai A, Sharma P, Jha RK, Jain P. Effect of sleeve gastrectomy and gastric bypass on diabetic control in Indore, India. Saudi J Obesity 2014;2:59–62.
63. Dasgupta A, Wasir J, Beloyartseva M, Malhotra S, Mithal A. An observational longitudinal study of the impact of sleeve gastrectomy on glycemic control in type 2 diabetes mellitus. Diabetes Technol Ther. 2013;15(12):990–5.
64. Lakdawala M, Shaikh S, Bandukwala S, Remedios C, Shah M, Bhasker AG. Roux-en-Y gastric bypass stands the test of time: 5-year results in low body mass index (30–35 kg/m^2^) Indian patients with type 2 diabetes mellitus. Surgery for Obesity and Related Diseases. 2013;9(3):370–8.
65. Bhasker AG, Remedios C, Batra P, Sood A, Shaikh S, Lakdawala M. Predictors of remission of T2DM and metabolic effects after laparoscopic Roux-en-Y gastric bypass in obese Indian diabetics—a 5-year study. Obesity surgery. 2015;25(7):1191–7.

## **Diet therapy**

### **RSSDI 2017 recommendations**

#### **Recommended care**

- A healthy Indian Mediterranean diet pattern is recommended due to its protective role in high-risk individuals with T2DM
- High-carbohydrate diets with relatively large proportion of unrefined carbohydrate and fiber-rich foods such as pulses, legumes, unprocessed vegetables, and fruits are recommended. Millets and brown rice are preferred to polished white rice
- Low-carbohydrate ketogenic diet is preferred than low-caloric diet in patients with T2DM
- Diet containing carbohydrates may be monitored by carbohydrate counting, glycemic index, glycemic load, exchanges, or experience-based estimation
- Protein intake equivalent to at least 15% of total daily calories is recommended
- Diet containing high proportion of fat and monounsaturated fats like sunflower oil significantly increases the risk of metabolic syndrome (MS); therefore, they are not recommended in obese T2DM patients
- Saturated fatty acid (SFA) intake should be less than 10% of total calories. Use of *trans* fatty acids (TFAs) should be avoided in high-risk individuals
- Intake of non-nutritive artificial sweeteners in moderate amounts may be considered
- High fiber diet in soluble form improves the glycemic and lipid profile
- Combining foods with high and low glycemic indices, such as adding fiber-rich foods to a meal or snack, improves the glycemic and lipaemic profiles
- Cardio-protective diet should include:
  - More leafy vegetables, vegetable salads, fruits, nuts, whole grains, coarse grains, sprouted grams, spices, and other foods rich in fiber and antioxidants
  - Moderate amounts of low fat milk and milk products, vegetable oils with SFA, monounsaturated fatty acids (MUFA) and polyunsaturated fatty acids (PUFA) (1:1:08), flesh foods (fish, chicken without skin, white of the egg, red meat), and artificial sweeteners
  - Avoid: Alcohol, excess sugar, industrial *trans* fat, saturated fats, and foods that are refined, processed, salt-rich, cholesterol-rich and deep-fried, polished rice, high fructose corn syrup (HFCS)
  - Total dietary salt intake should be reduced (< 5 g/day) in population at high risk of hypertension
- Provide access to a dietician (nutritionist) or other healthcare professionals trained in nutrition, at or around the time of diagnosis offering an initial consultation and during follow-up sessions as required, preferably individually or in groups
- Individualize advice on food and meals to match needs, preferences, and culture
- Advice on reducing energy intake and control of foods with high amounts of added refined sugars, fats, and alcohol
- Provide advice on the use of foods in the prevention and management of hypoglycemia where appropriate

#### **Limited care**

- Nutritional counselling may be provided by any healthcare personnel, with training in nutrition therapy, but not necessarily an accredited dietician nutritionist)
- “Mass awareness campaign for healthy diet and lifestyle” is essential for prevention of diabetes in India

#### **Preamble**

Nutritional and diet therapy remains an integral part of diabetes management. As there is no “one-size-fits-all” meal plan or eating pattern, RSSDI recommendations emphasis on development of individualized eating plan, based on individual’s health needs, personal and cultural preferences, access to healthful choices, health literacy, and numeracy [1, 2] in consonance with ADA [3]. The primary goal of the diet therapy is to improve health by providing calories for normal growth and development while achieving and maintaining optimal glycemia and normalizing dyslipidaemia [4]. Therefore, diets are often altered or/modified with respect to amount of carbohydrate, the type of fat, and the quantity and type of protein to meet these needs. Evidence from epidemiological and experimental studies focusing on nutritional intervention in the prevention of T2DM suggests that intake of foods with more non-starch polysaccharides and omega-3 fatty acids with low glycemic index (GI) may play a protective role, whereas excess intake of saturated fats and TFAs may contribute to the increased risk [2]. In people who are accustomed to consuming sugar sweetened foods, ADA recommends the use of non-nutritive sweeteners in moderate amounts as they have the potential to reduce overall calorie and carbohydrate intake [3]. However, the exact nature of diet appropriate for patients with T2DM still remains a matter of debate due to lack of tools and strategies that help to decide on healthy eating patterns to minimize the burden of disease [6]. Among Asian Indians from South Asia, intake of refined carbohydrate, saturated fatty acids (SFA) and n-6 PUFA as well as TFAs is higher and intake of n-3 PUFA and fiber is lower [7, 8]. In addition, attitudes, cultural differences, and religious and social beliefs and imbalances in dietary patterns pose significant barriers in effective prevention and management of T2DM [9]. Evidence suggest that intake of MUFAs among Asian Indians ranged from 4.7 to 16.4% that indirectly could contribute to increasing obesity, metabolic syndrome, and T2DM [10, 11]. In this context, it is felt that RSSDI recommendations on diet therapy can suggest approaches to the dietary management of diabetes in Asian Indians (Annexure III).

#### **Considerations**

The panel endorse most of the IDF recommendations on diet therapy with a few modifications based on the local factors that were reviewed in Indian context including high prevalence of both obesity and undernutrition, poor access to healthy food choices, and inadequate physical activity in some.

#### **Rationale and evidence**

##### *Carbohydrate monitoring*

- Several parameters like carbohydrate counting, glycemic index (GI), glycemic load (GL), exchanges, or experience-based estimation can be used for monitoring of carbohydrate content in food, which may be useful in diet management for T2DM patients [12].

##### *High-carbohydrate, low-fat diets*

- The most beneficial metabolic profile is provided by a high-carbohydrate low-fat diet, and the worst metabolic profile results from low-carbohydrate high-fat diets.
- High-carbohydrate diabetes diets are effective when relatively large amounts of unrefined carbohydrate and fiber are included such as legumes, unprocessed vegetables, and fruits.
- Carbohydrate intake is intertwined with fat intake, and low carbohydrate diets usually tend to be high on fat and/or high protein. Fat intake should occur mainly in the form of MUFA with a parallel decrease in SFAs and TFAs. Such a diet is particularly beneficial in patients with impaired glucose tolerance, diabetes, and obesity.
- A recent review suggests that high-carbohydrate diets are at least as effective as low-carbohydrate diets, associated with significant weight loss and a reduction in plasma glucose, A1C, and low density lipoprotein-cholesterol (LDL-C) levels in patients with T2DM. However, high-carbohydrate diets may raise serum triglyceride levels and reduce high density lipoprotein-cholesterol (HDL-C) levels, increasing the risk of cardiovascular disease, which can be improved with the consumption of a low GI/GL foods and high fiber [13].
- Evidence suggests that in patients with diabetes, weight loss achieved due to intake of low-carbohydrate diets is linked to duration of the diet restriction and reduced energy intake but not with restriction of carbohydrates alone. Therefore, obese diabetes patients should consider switching to a diet reduced in calories and fat to reduce the incidence of T2DM and myocardial infarction [14, 15].
- Asian Indians increasingly tend to consume high-carbohydrate diet in the form of refined grain. Data from Chennai Urban Rural Epidemiological Study (CURES) suggests that higher consumption of refined grains is significantly associated with higher waist circumference (*p* < 0.0001), systolic blood pressure (*p* < 0.0001), diastolic blood pressure (*p* = 0.03), fasting blood glucose (*p* = 0.007), serum triglyceride (*p* < 0.0001), lower high density lipoprotein cholesterol (*p* < 0.0001), and IR (*p* < 0.001). Individuals who consumed refined grains were more likely to have metabolic syndrome (odds ratio [OR], 7.83; 95% confidence interval, 4.72–12.99] and IR compared to those who did not consume [16].
- In another study that examined the association of dietary carbohydrates and glycemic load with the risk of T2DM among urban adult Asian Indian population, consumption of refined grain (OR 5.31 [95% CI = 2.98, 9.45]; *p* < 0.001), total carbohydrate (OR 4.98 [% CI = 2.69, 9.19], *p* < 0.001), glycemic load (OR 4.25 [95% CI = 2.33, 7.77]; *p* < 0.001), and glycemic index (OR 2.51 [95% CI = 1.42, 4.43]; *p* = 0.006) were positively associated with the risk of T2DM while dietary fiber intake was inversely associated with T2DM (OR 0.31 [95% CI = 0.15, 0.62]; *p* < 0.001) [17].
- Data from a population-based, cross-sectional study reporting dietary intake of urban Indian adults indicate that carbohydrates are the major source of energy (64%), followed by fat (24%) and protein (12%) among South Indian population. Refined cereals contributed to the bulk of the energy (45.8%), followed by visible fats and oils (12.4%) and pulses and legumes (7.8%). Intake of micronutrient-rich foods such as fruits and vegetables (265 g/day), and fish and seafood (20 g/day) was far below the FAO/WHO recommendation. This suggests that these aberrant dietary patterns among urban South Indians contribute to the diabetes risk in this population [18].
- The Urban Rural Epidemiological Study (CURES 147) report that white rice among all refined grains has high GI and contributes for almost 50 and 73% of daily total calories in diet in the urban and rural South Indians, respectively [19].
- Evidence suggests that improving the carbohydrate quality of the diet by replacing the common cereal staple white rice with brown rice could have beneficial effects on reducing the risk of diabetes and related complications. It was observed that consumption of brown rice was associated with significant reduction in 24-h glycemic response (*p* = 0.02) and fasting insulin response (*p* = 0.0001) among overweight Asian Indians [20].
- As Indians consume relatively more carbohydrates, it is very difficult to alter the amount of carbohydrate in their diets. Therefore, substituting brown rice in place of white rice can be an acceptable option and may reduce the risk of T2DM [8].
- Sugar and sugar-sweetened beverages have been found to increase the glycemic load. *Mohan et al*. report that intake of sugar in urban South Indians is mostly in the form of added sugar in hot beverages which contributed around 3.6% of total glycemic load compared to white rice (66%) [17].
- Recent studies report that the intake of total sugar (25.0 kg/capita) among Indians exceeds the average global annual per capita consumption (23.7 kg) [8, 21].

##### *Low-carbohydrate, ketogenic diet*

- A recent RCT reports that patients with T2DM had improved their glycemic control (*p* = 0.002) and lost more weight (*p* < 0.001) after being randomized to a very low-carbohydrate ketogenic diet (VLCKD) and lifestyle online program rather than a conventional, low-fat diabetes diet online program [22].
- Another trial from Kuwait reports that low-carbohydrate ketogenic diet (LCKD) had beneficial effect and improved glycemic control in patients with T2DM compared to low-caloric diet (LCD) [23].

##### *Low GI of pulses and pulse-incorporated cereal foods*

- Dal, roti, rice, and curry are the typical examples of Indian mixed diets that are unique from basic or less-mixed diets of Westerners, Black Africans, and other Asians. Different carbohydrate foods mixed with cereals exhibit GIs intermediate between the GI of each food individually. Within-individual variations in GI and insulinaemic indices of cereal-pulse mixtures are attributable to viscosity of food, high un-absorbable carbohydrate content, or delayed gastric emptying [24].
- Evidence suggests that replacing high GI diets with low GI diets combined with grams and pulses as staple will ensure satiety and adequate calories. Combining acarbose with such modified diet was associated with significant decline in postprandial blood glucose in T2DM patients with secondary failure with OADs [25].
- Similarly, use of thepla (wheat flour, Bengal gram flour, and oil) was associated with lower hyperglycemic and hyperinsulinemia effect in T2DM patients. Enhanced insulin secretion by pulses (gram flour) is attributed to lower GI of mixed diets in non-insulin dependent diabetes patients [26].
- A retrospective analysis shows that modified pulse-carbohydrate (75% pulse + 25% cereals) was associated with significant reduction in A1C (*p* < 0.01) and greater reduction in body weight compared to standard diet (75% cereals + 25% pulse) [27].
- A systematic review and meta-analysis of 12 RCTs report that diets including tree nuts at a median dose of 56 g/day significantly lowered A1C (MD  =  − 0.07% [95% CI = − 0.10, − 0.03%]; *p*  =  0.0003) and fasting glucose (MD  = − 0.15 mmol/L [95% CI = − 0.27, − 0.02 mmol/L]; *p*  =  0.03) compared with control diets in patients with diabetes [28].
- In a systematic review of dietary patterns in India, it was identified that diet rich in rice and pulses was associated with lower risk of diabetes whereas diet rich in sweets and snacks was associated with high risk [29].
- Consumption of legumes may be beneficial in T2DM prevention in older adults at high cardiovascular risk [30].

##### *Consumption of oils among Indian population*

- A recent study comprising 27,012 rural South Indians reports that the highest quintile of fat intake was significantly associated with the prevalence of abdominal obesity and impaired fasting glucose (33%, *p* < 0.001 and 23.3%, *p* = 0.003, respectively). Furthermore, sunflower oil as the main cooking oil was significantly associated with a higher risk of these components of the metabolic syndrome (MS) (*p* < 0.001) compared to traditional oils and palmolein [31].
- Another study evaluating the risk of MS with type of vegetables oils used for cooking among Asian Indians suggests that the prevalence of MS was higher among sunflower oil users (30.7%) than palmolein (23.2%) and traditional oil (17.1%, *p* < 0.001) users. Higher linoleic acid percentage, vitamin E, and linoleic acid/alpha-linolenic acid ratio in sunflower oil were assumed to contribute to increased risk of MS among Asian Indians [32].
- Dietary intervention with cooking oils containing high concentration of MUFA (canola and olive oil) compared to commonly used refined oils in Asian Indians with non-alcoholic fatty liver disease was associated with significant improvements in grading of fatty liver (*p* < 0.01), liver span (*p* < 0.05), measures of insulin resistance (in olive group) (*p* < 0.001), and lipids (high density lipoprotein in olive group, *p* = 0.004; triglyceride in the canola oil group, *p* = 0.02) [33].
- Indian diets predominantly vegetarian are relatively low in saturated FA, high in n-6 polyunsaturated fatty acids (PUFA), and very low in n-3 PUFA. They appear as a good dietary composition as per global standards. But the undeniable increase in the incidence of obesity, diabetes, and cardiovascular diseases in India draws the focus on a balance between fats, carbohydrates, and proteins, rather than an emphasis on individual macronutrients.

##### *Fiber and diabetes mellitus*

- Fiber-rich diet has got a definite role in the treatment of diabetes mellitus, obesity, and hypercholesterolemia or hyperlipidemia [34]. The beneficial effects of fiber-rich food in diabetes patients may be attributed to slow release of the absorbed glucose into the blood circulation resulting in decreased insulin secretion [35].
  - Diabetes patients on high carbohydrate and fiber diets are found to have lower postprandial glycemia and serum insulin concentration.
  - In obese diabetes patients, diet rich in fiber is particularly useful as it increases satiety, reduces the food intake, and also shows blood glucose reducing effect as is manifested by diminished GI.
- A recent RCT with full cross-over design including 56 Indian subjects reports that flatbreads with 15% chickpea flour (CPF) and 3 or 4% guar gum (GG) significantly reduced PPG (both ≥ 15% reduction in positive incremental AUC, *p* < 0.01) and postprandial insulin (PPI) (both ≥ 28% reduction in total AUC, *p* < 0.0001) compared with flatbreads made from control flour [36].
- Evidence suggests that high fiber diet, particularly of the soluble type, significantly improves glycemic control, decreases hyperinsulinemia, and lowers plasma lipid concentrations in patients with T2DM [37, 38].
- Furthermore, a recent randomized, controlled, parallel arm study reports that daily consumption of 3 g of soluble fiber from 70 g of oats was associated with beneficial effects on the lipid parameters, specifically total cholesterol and low density lipoprotein cholesterol in Asian Indians [39].
- Nonetheless, a meta-analysis of 17 prospective cohort studies did not find any direct correlation between dietary fiber intake and risk of T2DM [40].

##### *High prevalence of hypertension among Indian population: need for cardio-protective diet* [41, 42] (Annexure IV)

- A recent systematic review reports that the overall mean weighted salt intake was 10.98  g/day among Indians, which is higher than the current WHO recommendation (< 5 g/day) [43]. Higher intake of salt was significantly associated with higher prevalence of hypertension (*p* < 0.0001) and increased systolic and diastolic blood pressure (*p* < 0.0001). This calls for urgent steps to decrease salt consumption of the population at high risk [44].
- A recent systematic review and meta-analysis of population-based studies including several studies from India report that the prevalence of high salt intake is > 87% in urban India. Moreover, the study concludes that excessive salt intake has a greater impact on the prevalence of hypertension in urban than rural regions [45].
- Evidence from CURES indicate that higher intake of fruit and vegetables is associated with significant reduction in systolic blood pressure (*p* = 0.027), BMI (*p* < 0.0001), waist circumference (*p* < 0.0001), total cholesterol (*p* = 0.017), and LDL-cholesterol concentration (*p* = 0.039) [46]. This suggests that increased intake of fruits and vegetables may have protective role against CVD risk in Asian Indians who have high rates of premature CAD [46, 47].
- Lifestyle and dietary modifications are recommended as first-line management therapies for lipid and glucose control in patients diagnosed with diabetes or those with confirmed CVD. In newly diagnosed T2DM patients, initial dietary therapy substantially reduces plasma triglyceride, marginally improves total cholesterol and sub-fractions, and results in a potentially less atherogenic profile suggesting that healthy dietary habits help reduce the occurrence and mortality due to CVD events in people with and without established CAD [48–50].
- It has been observed that combining foods of known GI can alter the glycemic and lipemic profiles favorably, i.e., differences between foods of high and low glycemic indices may be kept minimal [13, 51, 52]. Addition of dietary fiber such as dicoccum wheat to the regular diet was associated with significant reduction in total lipids (*p* < 0.01), triglycerides (*p* < 0.01), and LDL-cholesterol (*p* < 0.05) and effectively reduced cardiovascular risk factors [53]. Moreover, a systematic review and meta-analysis including 22 cohort studies report an inverse relationship between total dietary fiber intake and risk of CVD (risk ratio 0.91 per 7 g/day [95% CI = 0.88 to 0.94]) and CAD (0.91 [0.87 to 0.94]) [54].
- Evidence from a 24-week randomized control trial in Asian Indians suggests that single food intervention with pistachio nuts has beneficial effects on the cardiometabolic profile in terms of significant improvements in WC (*p* < 0.01), FPG (*p* < 0.04), total cholesterol (*p* < 0.02), LDL cholesterol (*p* < 0.006), high sensitivity C-reactive protein (*p* < 0.05), tumor necrosis factor-α (*p* < 0.03), free fatty acids (*p* < 0.001), thiobarbituric acid reactive substances (*p* < 0.01), and adiponectin levels (*p* < 0.001) [55].
- Similarly, a systematic review and meta-analysis of RCTs report that pistachios, but not other nuts, significantly reduce SBP (MD = − 1.82; 95% CI = − 2.97, − 0.67; *p* = 0.002). Moreover, pistachios (MD = − 0.80; 95% CI = − 1.43, − 0.17; *p* = 0.01) and mixed nuts (MD = − 1.19; 95% CI = − 2.35, − 0.03; *p* = 0.04) have a significant DBP reducing effect [56].

##### *Indian consensus dietary guidelines to prevent obesity, metabolic syndrome, and diabetes*

- Excess consumption of calories, saturated fats, TFAs, simple sugars, salt, and low intake of fiber together with sedentary lifestyles led to an increase in obesity, T2DM, and CVD in both urban and rural populations of India [57]. In light of this, consensus dietary guidelines for Asian Indians are framed with an intention to curb rising epidemics of obesity, MS, hypertension, T2DM, and CVD. The consensus guidelines suggest:
  - Reduction in the intake of refined carbohydrates
  - Preferential intake of complex carbohydrates and low glycemic index foods
  - Intake of low-carbohydrate ketogenic foods
  - Higher intake of fiber
  - Lower intake of saturated fats
  - Optimal ratio of unsaturated to saturated fatty acids
  - Avoiding *trans* fatty acids
  - Slightly higher protein intake
  - Lower intake of salt
  - Restricted intake of refined sugar less than 10% of total daily energy [58]

##### *Diet-related non-communicable diseases (NCDs) in India*

- Studies evaluating secular trends in dietary intake in relation to NCDs in India suggest that, over the past three decades (1973–2004), a rapid transition in nutrition occurred with concurrent increase in obesity, hypertension, MS, T2DM, and CAD. Evidence indicates that there is a 7% decrease in energy derived from carbohydrates and a 6% increase in energy derived from fats. Decreased intake of coarse cereals, pulses, fruits, and vegetables, together with increased intake of meat products and salt, coupled with declining levels of physical activity resulted in escalated burden of NCDs in India [59]. Moreover, WHO revealed that four metabolic risk factors such as obesity, raised blood pressure, raised blood glucose, and raised blood total cholesterol levels and four behavioral risk factors such as tobacco use, unhealthy diet, physical inactivity, and harmful use of alcohol had largest contribution to the significant proportions of NCDs in India [60].

#### **Implementation**

Implementation of dietary management therapies demands knowledgeable and competent dietitians and nutritionists who are trained in providing effective dietary interventions that are consistent with individual’s needs and demands. Self-management and counselling in nutrition (for individuals or groups) should first include assessment, identification of the nutrition problem, and later implementation of nutritional strategies followed by nutrition monitoring and evaluation of individual outcomes. Nationwide community intervention programs aimed at creating awareness about the consequences of unhealthy food choices and replacing them with healthy food choices is urgently needed in India. Evidence from initial studies suggests that simple 4-week nutritional counselling provided to increase patient’s nutritional knowledge can significantly improve fasting and postprandial blood glucose levels in illiterate to semi-literate patients with T2DM [61], but long-term interventional studies are lacking. Increasing taxation on sugar-sweetened beverages has been shown to decrease the incidence of obesity and T2DM, suggesting that prevention strategies, encompassing multiple stakeholders (government, industry, and consumers), may decrease excessive sugar consumption in the Indian population [58].

**References**

1. Evert AB, Boucher JL, Cypress M, Dunbar SA, Franz MJ, Mayer-Davis EJ, et al. Nutrition therapy recommendations for the management of adults with diabetes. Diabetes Care. 2013;36(11):3821–42.
2. Feinman RD, Pogozelski WK, Astrup A, Bernstein RK, Fine EJ, Westman EC, et al. Dietary carbohydrate restriction as the first approach in diabetes management: critical review and evidence base. Nutrition. 2015;31(1):1–13.
3. American Diabetes Association. Standards of medical care in diabetes. 2017. Available at: http://professional.diabetes.org/sites/professional.diabetes.org/files/media/dc_40_s1_final.pdf
4. Sharma R, Chaturvedi S. Nutrition management of diabetes mellitus. RSSDI textbook of diabetes mellitus. 3rd edition. Jaypee Brothers Medical Publishers (P) Ltd. 2014, Chapter 30:465–80.
5. Steyn NP, Mann J, Bennett PH, Temple N, Zimmet P, Tuomilehto J, et al. Diet, nutrition and the prevention of type 2 diabetes. Public Health Nutr. 2004;7(1A):147–65.
6. Sahay BK. Dietary carbohydrates content in Indian diabetic patients. Medicine Update. 2012;22:235–9. Chapter 5.2.
7. Misra A, Khurana L, Isharwal S, Bhardwaj S. South Asian diets and insulin resistance. Br J Nutr. 2009;101(4):465–73.
8. Mohan V, Ruchi V, Gayathri R, Bai MR, Sudha V, Anjana RM, Pradeepa R. Slowing the diabetes epidemic in the World Health Organization South-East Asia Region: the role of diet and physical activity. WHO-South East-Asia Journal of Public Health. 2016; 5 (1):5–16
9. Misra A, Ramchandran A, Jayawardena R, Shrivastava U, Snehalatha C. Diabetes in South Asians. Diabet Med. 2014;31(10):1153–62.
10. Misra A, Singhal N, Khurana L. Obesity, the metabolic syndrome, and type 2 diabetes in developing countries: role of dietary fats and oils. J Am Coll Nutr. 2010;29(3 Suppl):289S–301S.
11. Wasir JS, Misra A. The metabolic syndrome in Asian Indians: impact of nutritional and socio-economic transition in India. Metab Syndr Relat Disord. 2004;2(1):14–23.
12. Gupta S. Diet in diabetes. Medicine. 2011:139.
13. Jung CH, Choi KM. Impact of high-carbohydrate diet on metabolic parameters in patients with type 2 diabetes. Nutrients. 2017;9(4):322.
14. Sackner-Bernstein J, Kanter D, Kaul S. Dietary intervention for overweight and obese adults: comparison of low-carbohydrate and low-fat diets. A meta-analysis. PLOS One. 2015;10(10):e0139817.
15. Srivastava S, Madhu SV. Low carbohydrate diet in management of obesity—a short review. Int J Diabetes Dev Ctries. 2005;25:42–5.
16. Radhika G, Van Dam RM, Sudha V, Ganesan A, Mohan V. Refined grain consumption and the metabolic syndrome in urban Asian Indians (Chennai Urban Rural Epidemiology Study 57). Metabolism. 2009;58(5):675–81.
17. Mohan V, Radhika G, Sathya RM, Tamil SR, Ganesan A, Sudha V. Dietary carbohydrates, glycaemic load, food groups and newly detected type 2 diabetes among urban Asian Indian population in Chennai, India (Chennai Urban Rural Epidemiology Study 59). British Journal of Nutrition. 2009;102:1498–1506.
18. Radhika G, Sathya RM, Ganesan A, Saroja R, Vijayalakshmi P, Sudha V, Mohan V. Dietary profile of urban adult population in South India in the context of chronic disease epidemiology (CURES-68). Public Health Nutrition. 2011;14:591–8.
19. Sowmya N, Lakshmipriya N, Arumugam K, Venkatachalam S, Vijayalakshmi P, Ruchi V, Geetha G, Anjana RM, Mohan V, Krishnaswamy K, Sudha V. Comparison of dietary profile of a rural south Indian population with the current dietary recommendations for prevention of non-communicable diseases (CURES 147). The Indian Journal of Medical Research. 2016;144(1):112.
20. Mohan V, Spiegelman D, Sudha V, Gayathri R, Hong B, Praseena K, et al. Effect of brown rice, white rice, and brown rice with legumes on blood glucose and insulin responses in overweight Asian Indians: a randomized controlled trial. Diabetes Technology and Therapeutics, 2014;16:317–25.
21. Gulati S, Misra A. Sugar intake, obesity, and diabetes in India. Nutrients. 2014;6(12):5955–74.
22. Saslow LR, Mason AE, Kim S, Goldman V, Ploutz-Snyder R, Bayandorian H, Daubenmier J, Hecht FM, Moskowitz JT. An online intervention comparing a very low-carbohydrate ketogenic diet and lifestyle recommendations versus a plate method diet in overweight individuals with type 2 diabetes: a randomized controlled trial. Journal of medical Internet research. 2017;19(2).
23. Hussain TA, Mathew TC, Dashti AA, Asfar S, Al-Zaid N, Dashti HM. Effect of low-calorie versus low-carbohydrate ketogenic diet in type 2 diabetes. Nutrition. 2012;28(10):1016–21.
24. Chandalia H, Neogi R, Mehta S. Mechanism of low glycemic index of pulses and pulse-incorporated cereal foods. Int J Diabetes Dev Ctries. 1992;12:9–11.
25. Gosh JM. Trial of low glycemic diet and acarbose therapy for control of post-prandial hyperglycemia in type 2 diabetes mellitus: preliminary report. Int J Diabetes Dev Ctries. 2005;25:80–4.
26. Singhania PR, Senray K. Postprandial glycemic and insulin responses to processed foods made from wheat flour. Int J Diabetes Dev Ctries. 2012;32(4):224–8.
27. Sekar V, Sundaram A, Lakshmi B, Kalaivani AR, Mala S, Banupriya M, et al. The effect of modified pulse-carbohydrate diet on weight and A1C in type 2 diabetic patients. Int J Diabetes Dev Ctries. 2006;25:16–8.
28. Viguiliouk E, Kendall CW, Mejia SB, Cozma AI, Ha V, Mirrahimi A, Jayalath VH, Augustin LS, Chiavaroli L, Leiter LA, de Souza RJ. Effect of tree nuts on glycemic control in diabetes: a systematic review and meta-analysis of randomized controlled dietary trials. PLOS One. 2014;9(7):e103376.
29. Green R, Milner J, Joy EJ, Agrawal S, Dangour AD. Dietary patterns in India: a systematic review. British Journal of Nutrition. 2016;116(1):142–8.
30. Becerra-Tomás N, Díaz-López A, Rosique-Esteban N, Ros E, Buil-Cosiales P, Corella D, Estruch R, Fitó M, Serra-Majem L, Arós F, Lamuela-Raventós RM. Legume consumption is inversely associated with type 2 diabetes incidence in adults: a prospective assessment from the PREDIMED study. Clinical Nutrition. 2017.
31. Narasimhan S, Nagarajan L, Vaidya R, Gunasekaran G, Rajagopal G, Parthasarathy V, Unnikrishnan R, Anjana RM, Mohan V, Sudha V. Dietary fat intake and its association with risk of selected components of the metabolic syndrome among rural South Indians. Indian Journal of Endocrinology and Metabolism. 2016;20(1):47.
32. Lakshmipriya N, Gayathri R, Praseena K, Vijayalakshmi P, Geetha G, Sudha V, et al. Type of vegetable oils used in cooking and risk of metabolic syndrome among Asian Indians. International Journal of Food Sciences and Nutrition, 2013; 64:131–9.
33. Nigam P, Bhatt S, Misra A, Chadha DS, Vaidya M, Dasgupta J, Pasha QM. Effect of a 6-month intervention with cooking oils containing a high concentration of monounsaturated fatty acids (olive and canola oils) compared with control oil in male Asian Indians with nonalcoholic fatty liver disease. Diabetes Technol Ther.2014;16(4):255–61.
34. Trivedi B, Maniyar KT, Patel B. Effect of fibre diet (guar) on cholesterol, blood glucose and body weight. Int J Diabetes Dev Ctries. 1999;19:31–3.
35. Mehta K, Kaur A. Dietary fibre. Review. Int J Diabetes Dev Ctries. 1992;12:12–8.
36. Boers HM, MacAulay K, Murray P, Dobriyal R, Mela DJ, Spreeuwenberg MA. Efficacy of fibre additions to flatbread flour mixes for reducing post-meal glucose and insulin responses in healthy Indian subjects. British Journal of Nutrition. 2017;117(3):386–94.
37. Chandalia M, Garg A, Lutjohann D, von Bergmann K, Grundy SM, Brinkley LJ. Beneficial effects of high dietary fiber intake in patients with type 2 diabetes mellitus. New England Journal of Medicine. 2000;342(19):1392–8.
38. Narayan S, Lakshmipriya N, Vaidya R, Bai MR, Sudha V, Krishnaswamy K, Unnikrishnan R, Anjana RM, Mohan V. Association of dietary fiber intake with serum total cholesterol and low density lipoprotein cholesterol levels in Urban Asian-Indian adults with type 2 diabetes. Indian Journal of Endocrinology and Metabolism. 2014;18(5):624.
39. Gulati S, Misra A, Pandey RM. Effects of 3 g of soluble fiber from oats on lipid levels of Asian Indians-a randomized controlled, parallel arm study. Lipids in Health and Disease. 2017;16(1):71.
40. Yao B, Fang H, Xu W, Yan Y, Xu H, Liu Y, Mo M, Zhang H, Zhao Y. Dietary fiber intake and risk of type 2 diabetes: a dose–response analysis of prospective studies. European Journal of Epidemiology. 2014;29(2):79–88.
41. Sivasankaran S. The cardio-protective diet. The Indian Journal of Medical Research. 2010;132(5):608.
42. Raghuram TC. Diet in diabetes with heart disease. Int J Diabetes Dev Ctries. 1995;15;123–6.
43. Johnson C, Praveen D, Pope A, Raj TS, Pillai RN, Land MA, Neal B. Mean population salt consumption in India: a systematic review. Journal of hypertension. 2017;35(1):3–9.
44. Radhika G, Sathya RM, Sudha V, Ganesan A, Mohan V. Dietary salt intake and hypertension in an urban south Indian population—[CURES-53]. J Assoc Physicians India. 2007;55:405–11.
45. Subasinghe AK, Arabshahi S, Busingye D, Evans RG, Walker KZ, Riddell MA, Thrift AG. Association between salt and hypertension in rural and urban populations of low to middle income countries: a systematic review and meta-analysis of population based studies. Asia Pacific Journal of Clinical Nutrition. 2016;25(2):402–13.
46. Radhika G, Sudha V, Sathya RM, Ganesan A, Mohan V. Association of fruit and vegetable intake with cardiovascular risk factors in urban South Indians. British Journal of Nutrition. 2008; 99: 398–405
47. Sachdeva S, Sachdev TR, Sachdeva R. Increasing fruit and vegetable consumption: challenges and opportunities. Indian Journal of Community Medicine: Official Publication of Indian Association of Preventive & Social Medicine. 2013;38(4):192.
48. Manley SE, Stratton IM, Cull CA, Frighi V, Eeley EA, Matthews DR, et al. Effects of three months’ diet after diagnosis of type 2 diabetes on plasma lipids and lipoproteins (UKPDS 45). UK Prospective Diabetes Study Group. Diabet Med. 2000;17(7):518–23.
49. Mozaffarian D, Wilson PW, Kannel WB. Beyond established and novel risk factors: lifestyle risk factors for cardiovascular disease. Circulation 2008;117(23):3031–8.
50. Mozaffarian D. Dietary and policy priorities for cardiovascular disease, diabetes, and obesity. Circulation. 2016;133(2):187–225.
51. ChatterjeeS, Sen A, Mookerjee GC, Mukherjee KL. The effect of Psyllium fibre supplement on lipid profile in patients with non-insulin-dependant diabetes mellitus (NIDDM). Int J Diabetes Dev Ctries. 1992;12:5–8.
52. Nuttal FQ, Mooradian AD, DeMarais R, Parker S. The glycemic effect of different meals approximately isocaloric and similar in protein, carbohydrate and fat content as calculated using ADA exchange lists. Diabetes Care. 1983;6(5):432–5.
53. Yenagi NB, Hanchinal RR, Patil CS, Koppikar V, Halagiet M. Glycemic and lipidemic response to dicoccum wheat (*Triticum dicoccum*) in the diet of diabetic patients. Int J Diabetes Dev Ctries. 2001;21;153–5.
54. Threapleton DE, Greenwood DC, Evans CE, Cleghorn CL, Nykjaer C, Woodhead C, Cade JE, Gale CP, Burley VJ. Dietary fibre intake and risk of cardiovascular disease: systematic review and meta-analysis. BMJ. 2013;347:f6879.
55. Gulati S, Misra A, Pandey RM, Bhatt SP, Saluja S. Effects of pistachio nuts on body composition, metabolic, inflammatory and oxidative stress parameters in Asian Indians with metabolic syndrome: a 24-wk, randomized control trial. Nutrition. 2014;30(2):192–7.
56. Mohammadifard N, Salehi-Abarghouei A, Salas-Salvadó J, Guasch-Ferré M, Humphries K, Sarrafzadegan N. The effect of tree nut, peanut, and soy nut consumption on blood pressure: a systematic review and meta-analysis of randomized controlled clinical trials. The American Journal of Clinical Nutrition. 2015:ajcn091595.
57. Misra A, Sharma R, Gulati S, Joshi SR, Sharma V, Ghafoorunissa, et al. National Dietary Guidelines Consensus Group. Consensus dietary guidelines for healthy living and prevention of obesity, the metabolic syndrome, diabetes, and related disorders in Asian Indians. Diabetes Technol Ther. 2011;13(6):683–94.
58. Gulati S, Misra A. Sugar intake, obesity, and diabetes in India. Nutrients. 2014;6(12):5955–74.
59. Misra A, Singhal N, Sivakumar B, Bhagat N, Jaiswal A, Khurana L. Nutrition transition in India: secular trends in dietary intake and their relationship to diet-related non-communicable diseases. J Diabetes. 2011;3(4):278–92.
60. World Health Organization. Burden of NCDs and their risk factors in India (Excerpted from Global Status Report on NCDs-2014)
61. Chandalia HB, Bagrodia J. Effect of nutritional counseling on the blood glucose and nutritional knowledge of diabetic subjects. Diabetes Care. 1979;2(4):353–6.

## **Lifestyle management**

### **RSSDI 2017 recommendations**

#### **Recommended care**

- Lifestyle advice including diet and physical activity should be given to all people with T2DM during the time of diagnosis.
- Lifestyle intervention is a cost-effective approach in prevention of T2DM.
- Lifestyle interventions should be reviewed yearly or at the time of any treatment or at every visit.
- Advice people with T2DM that lifestyle modification, by changing patterns of eating and physical activity, can be effective in controlling many of the adverse risk factors related to T2DM.
- The intensity of physical activity should be monitored by International Physical Activity Questionnaire (IPAQ)-long form or accelerometry in high-risk T2DM patients.
- Introduce physical activity gradually, based on the individual’s willingness and ability, and setting individualized and specific goals.
- Intensity of physical exercise should be individualized, as intensive activity produces/aggravates complications in some T2DM patients.
- A total of 60 min of physical activity is recommended every day for healthy Indians in view of the high predisposition to develop T2DM and CAD.
  - At least 30 min of moderate-intensity aerobic activity
  - Fifteen minutes of work-related activity
  - Fifteen minutes of muscle-strengthening exercises (at least 3 times/week)
- In the absence of contraindications, encourage resistance training three times in a week.
- Provide guidance for adjusting medications (insulin) and/or adding carbohydrate for physical activity.
- Yogic practices lead to improvement in glycemic control, reduction in BP, correction of dyslipidemia, reduction of IR and correction of hyperinsulinemia, and reduction of weight along with elimination of stress.
- Yogic practices can be combined with other forms of physical activity when it should be done for 30 min every day. For those individuals not having other forms of physical activity, it is recommended that yogic practices are carried out for 45–60 min to achieve the metabolic benefits.
- Yoga may be as effective as or better than exercise in improving a variety of health-related outcome measures.
- Yoga results in better outcome than exercise in heart rate variability (HRV), kidney function, menopausal symptoms, psychiatric symptoms, pain, sleep disturbances, stress, and lipid profile.

#### **Limited care**

- The principles and content of lifestyle management are as for recommended care.
- Encourage increased duration and frequency of physical activity (where needed), up to 30–45 min on all days of the week or an accumulation of at least 150 min per week of moderate-intensity aerobic activity (50–70% of maximum heart rate).

#### **Preamble**

Lifestyle intervention is an integral part of diabetes management along with dietary and pharmacological interventions. Lifestyle interventions, which include increased physical activity, dietary modification, as well as weight reduction among overweight people, have potential to prevent T2DM in high-risk individuals [1, 2]. Obesity, which is one of the foremost reasons for T2DM development and associated with higher CV risk, can be controlled with significant lifestyle modifications. Major forms of lifestyle modifications considered for patients with T2DM include dietary modification and increasing physical activity (both aerobic and resistance training). A wealth of literature supports the fact that regular physical activity reduces morbidity and mortality in patients with T2DM. Regular aerobic training not only reduces glycemic burden but also helps to prevent atherosclerotic CVD by several mechanisms. In older adults with diabetes, regular walking was associated with reduced all-cause and CVD mortality [3–6]. Interestingly, regular exercise is shown to provide similar benefits in patients with T2DM when these exercises are done in single or multiple bouts, as long as the recommended length of activity is performed. Recent reports indicate positive effects of resistance training in patients with T2DM. All T2DM patients without complications and even those with complications can perform mild–moderate physical activity with appropriate caution. Yoga is also known to reduce glycemic parameters, improve insulin sensitivity, and reduce the use of OADs [4, 7]. Since Asians are considered to do less physical activity compared to their western counter parts [8], lifestyle modification is of paramount importance for blood glucose control and CV protection in patients with T2DM.

#### **Considerations**

The panel endorsed most of the IDF recommendations on lifestyle modifications with additions on the specific role of yoga in the Indian context. The panel considered evidences of physical activity on glycemic parameters in view of the high predisposition of Asian Indians to develop T2DM and CAD. The benefits of yogic practices, individually and when combined with physical activity, are also considered.

#### **Rationale and evidence**

##### *Identification*

- International Physical Activity Questionnaire-long form and accelerometry are the tools used for monitoring the intensity of physical activity. Several studies used these devices for monitoring the extent of exercise in south Asian population [9, 10].

##### *Physical activity*

- Hepatic glucose production and peripheral glucose uptake maintain glucose homeostasis in the resting and fed states. However, both phenomena are affected due to progressive IR and cause hyperglycemia. Moreover compared to other ethnic groups, Asian Indians are more prone to MS and IR at a relatively young age [11–13].
- Physical inactivity is considered as a major risk factor of T2DM [13]. Evidence suggests that adequate physical activity may reduce up to 27% risk of T2DM [14].
- The IDPP report that the RRR for T2DM was 28.5% with lifestyle modification (*p* = 0.018), 26.4% with metformin (*p* = 0.029), and 28.2% with both (*p* = 0.022), as compared with the control group [15].
- A recent systematic review including 20 articles report that lifestyle intervention was also cost-effective for the primary prevention of T2DM [16]. Similar identifications have also been reported in South Indian patients [7].
- Pooled data from two IDPP (2006 and 2013) studies including 709 patients report that lifestyle modification reduces risk of T2DM by 35.4%; risk reduction was equally effective in patients with different BMI (obese and non-obese) and age groups (> 45 and < 45 years) [17].
- Physical activity tends to increase the blood flow to the muscles resulting in increased uptake of glucose and oxygen. The effects of aerobic training on glycemic control are well established. Adults with T2DM following a simple aerobic walking program report a significant decrease in glycemic parameters (A1C and FPG) as well as BMI [18, 19].
- A recent cross-sectional study reports that south Asian adults required more intense physical activity (232 min/week) compared to white Europeans (150 min/week) in order to obtain the same cardiometabolic risk factor score [10].
- Short-term progressive resistance training program either in untrained or supervised training has shown to significantly decrease elevated blood glucose levels, lipid parameters, and body weight in Asian Indians with T2DM [20, 21].
- A recent meta-analysis examined the effects of combined training of aerobic and resistance training vs each alone on A1C reduction and other physiological parameters, in patients with T2DM. Data from seven studies including 192 male and 240 female patients revealed that combined training decreased glycemic burden, abdominal adipose tissue, and lipid profiles (total cholesterol and triglycerides) without any adverse effects. [22].
- In T2DM patients with sedentary lifestyle and in whom structured aerobic exercise is not feasible, practicing resistance training and home-based walking were found to be safe, effective, and beneficial with significant decrease in A1C (*p* < 0.05), FPG, as well as depression and improved quality of life (QoL) [23].
- Physical activity is also known to improve the overall health status, ameliorate depressive symptoms, and decrease the rate of hospitalizations in patients with T2DM. A 2-year follow-up study in T2DM patients reports that physical activity status is an independent predictor of lower hospitalizations and an important strategy to reduce healthcare costs [24].
- Evidence indicates that exercise also improves psychological distress in patients with T2DM and results in improved well-being [25].
- Data suggests that intensive physical exercise reduces the risk of CVD in patients with IGT [26, 27]. A 23-year follow-up Chinese study report that lifestyle intervention for 6 years was associated with less cumulative incidence of CVD mortality (11.9 vs 19.6%, *p* = 0.033) and all-cause mortality (28.1 vs 38.4%, *p* = 0.049) compared to the control group [27].
- A recent review reports that exercise also produces some risk in certain individuals with T2DM like patients with CAD, diabetic foot, diabetic neuropathy, denervation, and loss of proprioception [28].
- However, recently, ADA recommended that adults with T2DM should engage in 150 min or more of moderate to vigorous intensity physical activity per week, spread over at least 3 days/week, with no more than two consecutive days without activity [29]. However, for the resource-limited settings, the IDF guideline for T2DM encourages increased duration and frequency of physical activity (where needed), up to 30–45 min on 3–5 days/week or an accumulation of 150 min/week of moderate-intensity aerobic activity (50–70% of maximum heart rate) [30]. These recommendations were discussed and a balanced recommendation was framed based on the vast experience of panel members.
- Furthermore, in view of the high predisposition of Asian Indians to develop T2DM and CAD, the physical activity consensus guidelines for Asian Indians [31] suggest a total of 60 min of physical activity every day, although hard evidence for a clear benefit of this in Indians is lacking. This can include:
  - At least 30 min of moderate-intensity aerobic activity
  - Fifteen minutes of work-related activity
  - Fifteen minutes of muscle-strengthening exercises
- Based on the previous recommendations, the RSSDI statement, in its previous version, recommended all patients with T2DM to perform regular exercises with either mild or moderate intensity (Annexure V).

##### *Behavioral lifestyle intervention*

- Behavioral lifestyle intervention (BLI) has a great role in the management of T2DM as it bridges the gap between motivation and action [32].
- A recent RCT reports that the A1C levels of the patients taking BLI were significantly reduced (− 1.56 ± 1.81, *p* < 0.05); however, there was no change observed in the control group at 6-month period [33].
- Similarly, another study conducted in Dutch primary care on high-risk individuals reports that insight into the process of behavior change can contribute to better adapted and potentially more effective interventions for diabetes prevention [32].
- Moreover, a systematic review which included Indian RCTs revealed that behavioral change strategy is an essential part of an effective lifestyle modification program and can be effectively used for prevention of T2DM [34].

##### *Yoga*

- Another important way of overcoming the chronic stress and negative affective state in patients with T2DM is through mind-body therapy, especially “yoga” [4, 35, 36]. Yoga is an old, traditional, psychological, physical, and spiritual exercise regimen in India [37].
- In the process of yoga, abdominal stretching leads to rejuvenation/regeneration of pancreatic cells and increases the utilization and metabolism of glucose in peripheral tissues, liver, and adipose tissues through enzymatic process. Furthermore, it improves blood supply to the muscles and muscular relaxation and enhances insulin receptor expression causing increment of glucose uptake and thus reduces blood sugar [28].
- Yoga appears to be a suitable alternative to supplement lifestyle intervention programs. Yoga, as a practice, is a holistic philosophy, in which physical exercises are intertwined with lifestyle and behavioral changes of the community, including diet, relaxation, and stress management.
- Recently, two meta-analyses including RCTs report that yogic exercises help in improving glycemic parameters and lipid profiles in patients with T2DM. However, high-quality RCTs are required to prove the long-term efficacy of yoga in patients with T2DM [35, 36].
- A systematic review of 33 controlled trials, including 80% studies from India, reveals that yogasans may result in significant improvements in glycemic control, lipid levels, and body composition. Furthermore, yoga may also lower oxidative stress and BP, enhance pulmonary and autonomic function, mood, sleep, and QoL, and reduce medication use in adults with T2DM [38].
- Several RCTs also report that yogic exercises reduce blood glucose, A1C, triglycerides, total cholesterol, very low density lipoprotein (VLDL), medication requirement [39], and oxidative stress [40]. improve cardiac autonomic functions [41], and result in greater weight loss and reduction in WC [42] in Indian T2DM patients.
- It has been previously documented from studies on healthy individuals that long-term practice of yoga leads to lower metabolic rates [43, 44], lower levels of the stress hormone cortisol [45], changes in the activity of the autonomous nervous system [46, 47], and increases in insulin sensitivity at target tissues which in turn reduces IR and subsequently increases peripheral utilization of glucose [48].
- Practicing yoga and pranayama for a period of 3 months in patients with T2DM showed beneficial effects on metabolic parameters (A1C, FPG, and PPG) and anthropometric measurements [49]. In addition, yogasanas tend to exhibit positive effect on glucose utilization and fat redistribution in these patients [50]. Patients practicing specific yogasanas for up to 40 days responded with significant decrease in FPG, PPG, WHR, and changes in insulin levels.
- Evidence also suggests that beneficial effects of yoga go beyond glycemic control with clinical improvement in nerve function observed in T2DM with sub-clinical neuropathy [51, 52]. In patients practicing specific yogasanas for up to 40 days, the right hand and left hand median nerve conduction velocity had increased from 52.81 ± 1.1 to 53.87 ± 1.1 m/s and 52.46 ± 1.0 to 54.75 ± 1/1 m/s, respectively [51].
- Yoga results in better outcome than exercise in HRV, kidney function, menopausal symptoms, psychiatric symptoms, pain, sleep disturbances, stress, and lipid profile [28].
- Some of the asanas like Kapaal Bhatti where there is a valsalva-like maneuver have a risk of producing vitreous hemorrhage in patients with significantly advanced retinopathy; hence, one should exercise caution and obtain clearance from their physician before performing such asanas.
- The expert panel opined that a combined approach of physical activity and yoga would provide more beneficial effects on metabolic control as well as several other physiological parameters in patients with T2DM.
- Currently, the role of yoga and fenugreek in the prevention of diabetes is being evaluated in the Indian Prevention of Diabetes Study by RSSDI.

#### **Implementation**

Implementation of lifestyle management in patients with T2DM requires adequate awareness and education from the treating physician. It is imperative that all healthcare professionals encourage patients to practice the combined approach of diet and physical activity along with pharmacological intervention. In this regard, providing patients with structured educational programs using information leaflets on practices and procedures of physical activity could greatly enhance the adherence and overall health of patients with T2DM.

**References**

1. Daivadanam M, Absetz P, Sathish T, Thankappan KR, Fisher EB, Philip NE, Mathews E, Oldenburg B. Lifestyle change in Kerala, India: needs assessment and planning for a community-based diabetes prevention trial. BMC Public Health. 2013;13(1):95.
2. Tuomilehto J, Schwarz P, Lindström J. Long-term benefits from lifestyle interventions for type 2 diabetes prevention. Diabetes Care. 2011;34(Supplement 2):S210–4.
3. Misra A, Nigam P, Hills AP, Chadha DS, Sharma V, Deepak KK, Vikram NK, Joshi S, Chauhan A, Khanna K, Sharma R, Mittal K, Passi SJ, Seth V, Puri S, Devi R, Dubey AP, Gupta S; Physical Activity Consensus Group. Consensus physical activity guidelines for Asian Indians. Diabetes Technol Ther. 2012;14(1):83–98.
4. Smith TC, Wingard DL, Smith B, Kritz-Silverstein D, Barrett-Connor E. Walking decreased risk of cardiovascular disease mortality in older adults with diabetes. J Clin Epidemiol. 2007;60(3):309–17.
5. Cheng SJ, Yu HK, Chen YC, Chen CY, Lien WC, Yang PY, Hu GC. Physical activity and risk of cardiovascular disease among older adults. International Journal of Gerontology. 2013;7(3):133–6.
6. Sadarangani KP, Hamer M, Mindell JS, Coombs NA, Stamatakis E. Physical activity and risk of all-cause and cardiovascular disease mortality in diabetic adults from Great Britain: pooled analysis of 10 population-based cohorts. Diabetes care. 2014;37(4):1016–23.
7. Madhu SV. World Diabetes Day 2015: healthy living & diabetes. The Indian journal of medical research. 2015;142(5):503.
8. Phadke UK. Physical activity and exercise in diabetes mellitus. RSSDI textbook of diabetes mellitus. 2014; Chapter 32: Page 509–20.
9. Emadian A, Thompson J. A mixed-methods examination of physical activity and sedentary time in overweight and obese South Asian Men living in the United Kingdom. International Journal of Environmental Research and Public Health. 2017;14(4):348.
10. Iliodromiti S, Ghouri N, Celis-Morales CA, Sattar N, Lumsden MA, Gill JM. Should physical activity recommendations for South Asian adults be ethnicity-specific? Evidence from a cross-sectional study of South Asian and White European Men and Women. PLOS One. 2016;11(8):e0160024.
11. Misra A, Vikram NK. Insulin resistance syndrome (metabolic syndrome) and obesity in Asian Indians: evidence and implications. Nutrition 2004; 20:482–491.
12. Misra A, Misra R, Wijesuriya M, Banerjee D. The metabolic syndrome in South Asians: continuing escalation & possible solutions. Indian J Med Res 2007;125:345–54.
13. Mohan V, Ruchi V, Gayathri R, Bai MR, Sudha V, Anjana RM, Pradeepa R. Slowing the diabetes epidemic in the World Health Organization South-East Asia Region: the role of diet and physical activity. WHO-South East-Asia Journal of Public Health. 2016; 5 (1):5–16.
14. Lee IM, Shiroma EJ, Lobelo F, Puska P, Blair SN, Katzmarzyk PT, Lancet Physical Activity Series Working Group. Effect of physical inactivity on major non-communicable diseases worldwide: an analysis of burden of disease and life expectancy. The lancet. 2012;380(9838):219–29.
15. Ramachandran A, Snehalatha C, Mary S, Mukesh B, Bhaskar AD, Vijay V. The Indian Diabetes Prevention Programme shows that lifestyle modification and metformin prevent type 2 diabetes in Asian Indian subjects with impaired glucose tolerance (IDPP-1). Diabetologia. 2006;49(2):289–97.
16. Alouki K, Delisle H, Bermúdez-Tamayo C, Johri M. Lifestyle interventions to prevent type 2 diabetes: a systematic review of economic evaluation studies. Journal of Diabetes Research. 2016;2016.
17. Nanditha A, Snehalatha C, Ram J, Selvam S, Vijaya L, Shetty SA, Arun R, Ramachandran A. Impact of lifestyle intervention in primary prevention of type 2 diabetes did not differ by baseline age and BMI among Asian‐Indian people with impaired glucose tolerance. Diabetic Medicine. 2016.
18. Shenoy S, Guglani R, Sandhu JS. Effectiveness of an aerobic walking program using heart rate monitor and pedometer on the parameters of diabetes control in Asian Indians with type 2 diabetes. Prim Care Diabetes. 2010;4(1):41–5.
19. Qiu S, Cai X, Schumann U, Velders M, Sun Z, Steinacker JM. Impact of walking on glycemic control and other cardiovascular risk factors in type 2 diabetes: a meta-analysis. PLOS One. 2014;9(10):e109767.
20. Misra A, Alappan NK, Vikram NK, Goel K, Gupta N, Mittal K, Bhatt S, Luthra K. Effect of supervised progressive resistance-exercise training protocol on insulin sensitivity, glycemia, lipids, and body composition in Asian Indians with type 2 diabetes. Diabetes Care. 2008;31(7):1282–7.
21. Hameed UA, Manzar D, Raza S, Shareef MY, Hussain ME. Resistance training leads to clinically meaningful improvements in control of glycemia and muscular strength in untrained middle-aged patients with type 2 diabetes mellitus. N Am J Med Sci. 2012;4(8):336–43.
22. Hou Y, Lin L, Li W, Qiu J, Zhang Y, Wang X. Effect of combined training versus aerobic training alone on glucose control and risk factors for complications in type 2 diabetic patients: a meta-analysis. International Journal of Diabetes in Developing Countries. 2015;35(4):524–32.
23. Aylin k, Arzu D, Sabri S, Handan TE, Ridvan A. The effect of combined resistance and home-based walking exercise in type 2 diabetes patients. Int J Diab Dev Ctries. 2009;29(4):159–164.
24. Vasconcelos JP, de Bruin VM, Daniele TM, de Bruin PF, e Forti AC. Physical activity reduces the risk for hospitalizations in patients with type 2 diabetes. International Journal of Diabetes in Developing Countries. 2015;35(2):237–9.
25. Yavari A, Abbasi NM, Vahidi R, Najafipoor F, Farshi MG. Effect of exercise on psychological well-being in T2DM. Journal of Stress Physiology & Biochemistry. 2011;7(3).
26. Chen Y, Wang J, An Y, Gong Q, He Y, Zhang B, Li H, Shuai Y, Tang X, Jiang Y, Hong J. Effect of lifestyle interventions on reduction of cardiovascular disease events and its mortality in pre-diabetic patients: long-term follow-up of Da Qing Diabetes Prevention Study. Zhonghua nei ke za zhi. 2015;54(1):13–7.
27. Li G, Zhang P, Wang J, An Y, Gong Q, Gregg EW, Yang W, Zhang B, Shuai Y, Hong J, Engelgau MM. Cardiovascular mortality, all-cause mortality, and diabetes incidence after lifestyle intervention for people with impaired glucose tolerance in the Da Qing Diabetes Prevention Study: a 23-year follow-up study. The Lancet Diabetes & Endocrinology. 2014;2(6):474–80.
28. Thangasami SR, Chandani AL, Thangasami S. Emphasis of yoga in the management of diabetes. J Diabetes Metab. 2015;6(613):2.
29. American Diabetes Association. Standards of medical care in diabetes. 2017. Available at: http://professional.diabetes.org/sites/professional.diabetes.org/files/media/dc_40_s1_final.pdf
30. International Diabetes Federation Guideline Development Group. Global guideline for type 2 diabetes. Diabetes Res Clin Pract. 2014;104(1):1–52
31. Misra A, Nigam P, Hills AP, Chadha DS, Sharma V, Deepak KK, et al. Physical Activity Consensus Group. Consensus physical activity guidelines for Asian Indians. Diabetes Technol Ther. 2012;14(1):83–98.
32. Vermunt PW, Milder IE, Wielaard F, Baan CA, Schelfhout JD, Westert GP, van Oers HA. Behavior change in a lifestyle intervention for type 2 diabetes prevention in Dutch primary care: opportunities for intervention content. BMC family practice. 2013;14(1):78.
33. Abdi S, Sadiya A, Ali S, Varghese S, Abusnana S. Behavioural Lifestyle Intervention Study (BLIS) in patients with type 2 diabetes in the United Arab Emirates: a randomized controlled trial. BMC Nutrition. 2015;1(1):37.
34. Baker MK, Simpson K, Lloyd B, Bauman AE, Singh MA. Behavioral strategies in diabetes prevention programs: a systematic review of randomized controlled trials. Diabetes Research and Clinical Practice. 2011;91(1):1–2.
35. Cui J, Yan JH, Yan LM, Pan L, Le JJ, Guo YZ. Effects of yoga in adults with type 2 diabetes mellitus: a meta‐analysis. Journal of Diabetes Investigation. 2017;8(2):201–9.
36. Eun CS, Dol KS. Effects of yogic exercise on glycemic control and lipid profiles in type 2 diabetes: a meta-analysis of randomized controlled trials. Indian Journal of Traditional Knowledge 2017; 16:S109–16
37. Aljasir B, Bryson M, Al-shehri Bandar. Yoga practice for the management of type ii diabetes mellitus in adults: a systematic review. Evid Based Complement Alternat Med : eCAM. 2010;7(4):399–408.
38. Innes KE, Selfe TK. Yoga for adults with type 2 diabetes: a systematic review of controlled trials. Journal of Diabetes Research. 2015;2016.
39. Nagarathna R, Usharani MR, Rao AR, Chaku R, Kulkarni R, Nagendra HR. Efficacy of yoga based life style modification program on medication score and lipid profile in type 2 diabetes—a randomized control study. International Journal of Diabetes in Developing Countries. 2012;32(3):122–30.
40. Hegde SV, Adhikari P, Kotian S, Pinto VJ, D’souza S, D’souza V. Effect of 3-month yoga on oxidative stress in type 2 diabetes with or without complications. Diabetes care. 2011;34(10):2208–10.
41. Jyotsna VP, Ambekar S, Singla R, Joshi A, Dhawan A, Kumar N, Deepak KK, Sreenivas V. Cardiac autonomic function in patients with diabetes improves with practice of comprehensive yogic breathing program. Indian Journal of Endocrinology and Metabolism. 2013;17(3):480.
42. McDermott KA, Rao MR, Nagarathna R, Murphy EJ, Burke A, Nagendra RH, Hecht FM. A yoga intervention for type 2 diabetes risk reduction: a pilot randomized controlled trial. BMC Complementary and Alternative Medicine. 2014;14(1):212.
43. Chaya MS, Nagendra HR. Long-term effect of yogic practices on diurnal metabolic rates of healthy subjects. Int J Yoga. 2008;1(1):27–32
44. Seo DY, Lee S, Figueroa A, Kim HK, Baek YH, Kwak YS, Kim N, Choi TH, Rhee BD, Ko KS, Park BJ. Yoga training improves metabolic parameters in obese boys. The Korean Journal of Physiology & Pharmacology. 2012;16(3):175–80.
45. Yadav RK, Magan D, Mehta N, Sharma R, Mahapatra SC. Efficacy of a short-term yoga-based lifestyle intervention in reducing stress and inflammation: preliminary results. J Altern Complement Med. 2012;18(7):662–667.
46. Khattab K, Khattab AA, Ortak J, Richardt G, Bonnemeier H. Iyengar yoga increases cardiac parasympathetic nervous modulation among healthy yoga practitioners. Evid Based Complement Alternat Med. 2007;4(4):511–517.
47. Streeter CC, Gerbarg PL, Saper RB, Ciraulo DA, Brown RP. Effects of yoga on the autonomic nervous system, gamma-aminobutyric-acid, and allostasis in epilepsy, depression, and post-traumatic stress disorder. Medical Hypotheses. 2012;78(5):571–9.
48. Chimkode SM, Kumaran SD, Kanhere VV, Shivanna R. Effect of yoga on blood glucose levels in patients with type 2 diabetes mellitus. Journal of Clinical and Diagnostic Research: JCDR. 2015;9(4):CC01.
49. Balaji PA, Smitha R, Ali SS. Effects of yoga—pranayama practices on metabolic parameters and anthropometry in type 2 diabetes. IMRJ. 2011;1(10):1–4.
50. Malhotra V, Singh S, Tandon OP, Sharma SB. The beneficial effect of yoga in diabetes. Nepal Med Coll J. 2005;7(2):145–7.
51. Malhotra V, Singh S, Tandon OP, Madhu SV, Prasad A, et al. Effect of Yoga asanas on nerve conduction in type 2 diabetes. Indian J Physiol Pharmacol. 2002;46(3):298–306.
52. Naik D, Thomas N. Yoga—a potential solution for diabetes & metabolic syndrome. The Indian Journal of Medical Research. 2015;141(6):753.

## **Education**

### **RSSDI 2017 recommendations**

#### **Recommended care**

- Identification of high-risk individuals for T2DM and early intervention in the form of health education is recommended for prevention of diabetes.
- A patient-centered, structured self-management education is an integral part of the care of all people with T2DM.
- Diabetes self-management education is recommended for all people with diabetes at the time of diagnosis and it should be a continuous process and should be done whenever needed.
- Use an appropriately trained multidisciplinary team to provide education to groups of people with diabetes or individually if group work is considered unsuitable. Where desired, include a family member or friend or any care taker.
- The education program should focus more on rural patients as they have less knowledge and awareness regarding diabetes.
- Include in education teams a healthcare professional with specialist training in diabetes and delivery of education for people with diabetes.
- Diabetes education should be focused towards the assessment of change in behavior of people and promote self-management in person with T2DM.
- Ensure that education is accessible to all people with diabetes, taking account of culture, ethnicity, psychosocial, literacy, and disability issues. Consider delivering education in the community or at a local diabetes center, through technology and in different languages. Include education about the potential risk of alternative medicine and common myths about diabetes.
- Use techniques of active learning (engagement in the process of learning and with content related to personal experiences), adapted to personal choices and learning styles.
- Use modern communications technologies to advance the methods of delivery of diabetes education.
- Provide ongoing diabetes self-management support.

#### **Limited care**

- The principles are as for Recommended care but education may be provided by a smaller team (physician and educator) or in very limited situations by an appropriately skilled individual.
- Consider how available technologies can best be used to deliver education.

#### **Preamble**

Diabetes self-management education (DSME) is recognized as an important component of management of T2DM. Individuals with diabetes tend to make dramatic impact on the progression and development of diabetes by involving in their own diabetes self-care practices [1]. Moreover, diabetes education improves metabolic control, prevents or delay complications, improves QoL, and empowers people to make informed choices to manage their condition [2]. It is expected that patient who is well-educated has better understanding of the disease and self-manages the condition more effectively [3]. Evidence from literature suggests that implementation of DSME has been successful in lowering glycemic levels in uncontrolled patients [4, 5]. The DSME is an ongoing process that facilitates knowledge, skill, and ability necessary for diabetes self-care [5, 6]. It is guided by evidence-based standards while incorporating needs, goals, and life experiences of the person with diabetes [7]. India is a country with diverse social, economic, cultural, and educational patterns with majority of population residing in rural areas. Evidence has also revealed that rural people have less knowledge and awareness than urban Indian people [8]. It is presumed that the poor level of awareness on diabetes in India is due to high percentage of individuals with low or no literacy [9, 10].

#### **Considerations**

The panel endorsed the IDF recommendations on education as such. However, evidence from India together with local factors such as cost, literacy, malnutrition, body weight, and BMI were reviewed in the Indian context and are reflected in the recommendations.

#### **Rational and evidence**

##### *Educational programs and their outcomes*

- In management of T2DM patient, structured diabetes care program (Freedom 365*) of ongoing diabetes education on diet and lifestyle correction, biochemical investigations, clinical monitoring, and treatment at regular intervals was associated with better clinical outcomes compared to routine medical care. The program played a pivotal role in improving the patient’s quality of care by overcoming clinical inertia and improving adherence to therapy while preventing the occurrence/progression of diabetes-associated complications [11].
- Organized diabetes education that involves improving knowledge on better control of disease symptoms, disease regimens, and dangers in practice was found to positively impact lifestyle changes, self-control abilities, and at the same time improve the QoL in T2DM patients [12].
- A recent systematic review including 118 unique interventions reports that DSME was associated with a statistically significant mean reduction in A1C (− 0.74 for intervention and − 0.17 for control groups) [5].
- The National Diabetes Education Program (NDEP) course was designed to enable educators in India to provide a complete perspective of the disease condition, the importance of self-care, blood glucose monitoring, diet and physical activity, self-injection of insulin, medication adherence, and the long-term benefits of compliance and a basic awareness of the various complications of diabetes. Following its implementation, most of participants acknowledge that they learned new skills and they were benefited by increase in knowledge, confidence, and improved attitude towards diabetes care among the participants [13].
- In order to minimize the increasing burden of NCDs, the Ministry of Health and Family Welfare, Government of India, has launched the National Programme on Prevention and Control of Diabetes, Cardiovascular Diseases and Stroke (NPDCS) on 8 January 2008 with several objectives including health promotion and health education for the community [14].
- Besides diabetes, educational intervention was also successful in reducing some of the obesity parameters and improving dietary patterns in individuals with prediabetes and diabetes. Initiation of primary prevention strategies through education right from elementary schools could reduce IFG by 17% suggesting such interventions may delay T2DM or even change the course of disease for improved outcomes among vulnerable population groups [15].
- Awareness about early detection and treatment of hyperglycemia in pregnancy is also important, as it will not only offer better fetal outcome but a good glycemic control during pregnancy will provide better intrauterine metabolic environment, which may help to prevent the development of diabetes, obesity, and MS in these offspring of diabetic mother in their later life [16].

##### *Knowledge and awareness*

- The ICMR-INDIAB study reports that the awareness of diabetes in urban India was significantly higher than rural residents (58.4 vs 36.8%, *p* < 0.001). Furthermore, participants from Tamil Nadu had the highest (31.7) and Jharkhand the lowest (16.3) knowledge score, and among self-reported subjects with diabetes, Maharashtra had the highest (70.1) and Tamil Nadu the lowest score (56.5) [8].
- Similarly, another ICMR-INDIAB study including 14,277 participants reveals that only 480 subjects were with self-reported diabetes (254 urban and 226 rural) and the level of glycemic control among subjects with self-reported diabetes in India is poor [17].
- A population-based study from a South Indian state reports that among 6211 participants, good knowledge and positive attitude were observed in 3457 (55.6%) and 3280 (52.8%) people, respectively. Furthermore, literacy had a significant association with good knowledge, attitude, and practice in general and T2DM population. Overall, women had significantly better knowledge (*p* < 0.001) [18].
- A recent study from east Delhi, India reports that self-learning module (SLM) was significantly associated with increasing knowledge on aspects of effect of diabetes on foot (*p* < 0.05) and foot care and its steps (*p* < 0.05) than the control group in T2DM patients [19].
- Though general practitioners in India are well aware and updated about symptoms and screening of T2DM, there is lack of effective approach towards screening and treatment of complications. Most of the patients were not advised on non-pharmacological measures and diabetes education, while interpretation of test results for screening of disease and its complications appeared to be a major flaw in general practice [20].
- Evidence from several studies determining the level of knowledge and awareness on diabetes across India suggests that most of the patients had poor knowledge and awareness about their condition [8, 21–27]. Low socioeconomic status, old age, cultural factors, lack of access to healthcare, family history of diabetes, and importantly low literacy levels were the major predictors of poor glycemic control among patient with T2DM.

##### *Challenges in diabetes management in India* [28–31]

- The awareness about the disease and its complications is less than satisfactory.
- There is lack of knowledge attitude practice studies to determine the gaps in knowledge among diabetics and physicians in the areas of individual diabetes care in India.
- Inadequate knowledge, delay in clinical response, and poor control are some of the physician-related issues that need to be addressed through diabetes education.
- Patients’ lack of knowledge about diabetes care is a significant barrier that can impede their ability to manage their disease. In view of this, there is an imperative need for more structured diabetes education programs in India.
- Lack of strong referral system to provide quality care, i.e., early diagnosis, prevention, and control of chronic complications in diabetes.
- Indian studies have also shown that barriers to insulin therapy are due to lack of awareness, causing wrong perception and false beliefs. People those who are already on insulin therapy showed to have better understanding and acceptability towards insulin therapy than those that are not on insulin; still intensification remains a challenge in these patients [32].
- Implementing efficacious health service intervention like patient education in a real-world resource-constrained setting is challenging and may not prove effective in improving patient outcomes. Therefore, interventions need to consider patients’ and healthcare providers’ experiences and perceptions and how macro-level policies translate into practice within local health systems [33].

##### *Assessing the need for evidence-based education* [34–38]

- A qualified diabetes educator who can be a nurse, a dietician, or a social worker can fill up the important void of poor awareness and play a major role in optimal diabetes care.
- Continuous medical education and additional trainings are needed to help health professionals integrate new knowledge and transform old practices.
- There is a need to assess the impact of existing education and training programs in the management of diabetes.
- Investment must be made to ensure that specialized diabetes education is accessible to healthcare personnel and people with diabetes.
- General practitioners and physicians should be periodically updated on recent guidelines on diagnosis, treatment, as well as management goals.
- Key aims of diabetes education are to change behavior of people and promote self-management.
- Steps to improve awareness in diabetes care in India:
  - Physician and family physician education
  - Need for continuing medical education
  - Education for patients with diabetes
  - Diabetes education programs in India
- Study has shown that a pharmacist may also be involved with clinicians as a part of collaborative diabetes care and has documented positive clinical, humanistic, and economic outcomes, which emphasized the value of multidisciplinary collaborative care for Asian diabetes patients and supported the effectiveness of this approach in managing chronic diseases [38].
- Counselling is the most important strategy capable of bringing about sustained lifestyle changes.

#### **Implementation**

- Major components of implementing these recommendations are the recruitment of personnel and their training in the principles of both diabetes education and behavior change strategies. The staff are required to develop theoretically based, patient-centered, ongoing follow-up education programs for people with diabetes. Educational strategies and materials matched to the needs and culture of the community served with attention to health literacy are necessary. Institutional support at the practice, community, and healthcare system levels is critically important.
- Diabetes discrimination at education institutes and workplace is often the result of a lack of knowledge about diabetes. Because diabetes is usually a “hidden” disability, many do not understand what it is like to have diabetes. By educating such authorities about diabetes and their needs and abilities, they may be able to get fair treatment.
- Mass awareness campaign through various print and electronic media will also be an effective model of education.

**References**

1. Intensive blood-glucose control with sulphonylurea or insulin compared with conventional treatment and risk of complications in patients with type 2 diabetes (UKPDS 33). UK Prospective Diabetes Study (UKPDS) Group. Lancet. 1998;352(9131):837–53.
2. Horigan G, Davies M, Findlay‐White F, Chaney D, Coates V. Reasons why patients referred to diabetes education programmes choose not to attend: a systematic review. Diabetic Medicine. 2017;34(1):14–26.
3. Shrivastava SR, Shrivastava PS, Ramasamy J. Role of self-care in management of diabetes mellitus. J Diabetes Metab Disord. 2013;12(1):14.
4. Norris SL, Lau J, Smith SJ, Schmid CH, Engelgau MM. Self-management education for adults with type 2 diabetes: a meta-analysis of the effect on glycemic control. Diabetes Care. 2002;25(7):1159–71.
5. Chrvala CA, Sherr D, Lipman RD. Diabetes self-management education for adults with type 2 diabetes mellitus: a systematic review of the effect on glycemic control. Patient education and counseling. 2016;99(6):926–43.
6. Scain SF, Friedman R, Gross JL. A structured educational program improves metabolic control in patients with type 2 diabetes: a randomized controlled trial. Diabetes Educ. 2009;35(4):603–11.
7. Mulcahy K, Maryniuk M, Peeples M, Peyrot M, Tomky D, Weaver T, et al. Diabetes self-management education core outcomes measures. Diabetes Educ. 2003;29(5):768–70, 773–84, 787–8 passim.
8. Deepa M, Bhansali A, Anjana RM, Pradeepa R, Joshi SR, Joshi PP, et al. Knowledge and awareness of diabetes in urban and rural India: the Indian Council of Medical Research India Diabetes Study (Phase I): Indian Council of Medical Research India Diabetes 4. Indian J Endocr Metab 2014;18:379–85.
9. Mohan D, Raj D, Shanthirani CS, Datta M, Unwin NC, Kapur A, et al. Awareness and knowledge of diabetes in Chennai—the Chennai Urban Rural Epidemiology Study [CURES-9]. J Assoc Physicians India. 2005;53:283–7.
10. Ramachandran A, Snehalatha C, Baskar AD, Mary S, Kumar CK, Selvam S, et al. Temporal changes in prevalence of diabetes and impaired glucose tolerance associated with lifestyle transition occurring in the rural population in India. Diabetologia 2004;47(5):860–5.
11. Singh DK and Tari V. Structured diabetes care (Freedom 365*) provides better glycemic control than routine medical care in type 2 diabetes: proof of concept observational study. Int J Diabetes Dev Ctries. 2015.
12. Jankowska-Polańska B, Fal AM, Uchmanowicz I, Seń M, Polański J, Kurpas D. Influence of organized diabetic education on self-control and quality of life of patients with type 2 diabetes. International Journal of Diabetes in Developing Countries. 2015;35(2):79–87..
13. Joshi S, Joshi SR, Mohan V. Methodology and feasibility of a structured education program for diabetes education in India: the National Diabetes Educator Program. Indian J Endocrinol Metab. 2013;17(3):396–401.
14. Verma R, Khanna P, Mehta B. National programme on prevention and control of diabetes in India: need to focus. Australas Med J. 2012;5(6):310–5.
15. Balagopal P, Kamalamma N, Patel TG, Misra R. A community-based diabetes prevention and management education program in a rural village in India. Diabetes Care. 2008;31(6):1097–104.
16. American Diabetes Association. Standards of medical care in diabetes—2017 abridged for primary care providers. Clinical Diabetes. 2017;35(1):5–26.
17. Unnikrishnan R, Anjana RM, Deepa M, Pradeepa R, Joshi SR, Bhansali A, Dhandania VK, Joshi PP, Madhu SV, Rao PV, Lakshmy R. Glycemic control among individuals with self-reported diabetes in India—the ICMR–INDIAB study. Diabetes technology & therapeutics. 2014;16(9):596–603.
18. Hussain R, Rajesh B, Giridhar A, Gopalakrishnan M, Sadasivan S, James J, Vijayan PP, John N. Knowledge and awareness about diabetes mellitus and diabetic retinopathy in suburban population of a South Indian state and its practice among the patients with diabetes mellitus: a population-based study. Indian Journal of Ophthalmology. 2016;64(4):272.
19. Devi R, Kapoor B, Singh MM. Effectiveness of self-learning module on the knowledge and practices regarding foot care among type II diabetes patients in East Delhi, India. International Journal of Community Medicine and Public Health. 2017;3(8):2133–41.
20. Patel N, Deshpande S, Godbole V, Champaneri V, Singh N. Awareness and approach towards diagnosis and treatment of diabetes type 2 and its complication among general practioners of western Vadodara. International Journal of Diabetes in Developing Countries. 2015;35(3):138–42.
21. Mathew AC, Jacob N, Jose S, Rathan P, Suvetha K, Senthil Kumar R, Yunsheng Ma. Knowledge about risk factors, symptoms and complications of diabetes among adults in South India. Int J Med Sci Public Health. 2014;3(9):1086–92.
22. Singh AK, Mani K, Krishnan A, Aggarwal P, Gupta SK. Prevalence, awareness, treatment and control of diabetes among elderly persons in an urban slum of Delhi. Indian J Community Med. 2012;37(4):236–9.
23. Muninarayana C, Balachandra G, Hiremath SG, Iyengar K, Anil NS. Prevalence and awareness regarding diabetes mellitus in rural Tamaka, Kolar. Int J Diabetes Dev Ctries. 2010;30(1):18–21.
24. Saurabh S, Sarkar S, Selvaraj K, Kar SS, Kumar SG, Roy G. Effectiveness of foot care education among people with type 2 diabetes in rural Puducherry, India. Indian J Endocr Metab 2014;18(1):106–10.
25. Kide S, Rangari A, Shiral R, Mane N, Yadav P, Ambulkar K, et al. Knowledge and awareness of diabetes amongst diabetes patients in Wardha region. Int J Diabetes Dev Ctries 2014;34(4):232.
26. Limaye TY, Wagle SS, Kumaran K, Joglekar CV, Nanivadekar A, Yajnik CS. Lack of knowledge about diabetes in Pune—the city of knowledge. Int J Diabetes Dev Ctries. 2015
27. Long P, Long KNG, Ankit Kedia, Gren LH, Andrew Smith, Jatrik Biswas. A cross-sectional study of diabetic knowledge in West Bengal, India: an analysis based on access to health care. Int J Diabetes Dev Ctries. 2015.
28. Mahajan PB. Role of medical colleges in prevention and control of diabetes in India: a ten point approach. Int J Diabetes Dev Ctries 2011;31(1):41–2.
29. Venkataraman K, Kannan AT, Mohan V. Challenges in diabetes management with particular reference to India. Int J Diabetes Dev Ctries. 2009;29(3):103–9.
30. Krishnan A, Gupta V, Ritvik BN, Thakur JS. How to effectively monitor and evaluate NCD programmes in India. Indian Journal of Community Medicine: Official Publication of Indian Association of Preventive & Social Medicine. 2011;36(Suppl1):S57.
31. Shrivastava SR, Shrivastava PS, Ramasamy J. Role of self-care in management of diabetes mellitus. Journal of Diabetes & Metabolic Disorders. 2013;12(1):14.
32. Gupta K, Gupta S. Barriers to insulin therapy. Journal of Diabetes Education:2013b; 3; 17–23.
33. Bhojani U, Kolsteren P, Criel B, De Henauw S, Beerenahally TS, Verstraeten R, Devadasan N. Intervening in the local health system to improve diabetes care: lessons from a health service experiment in a poor urban neighborhood in India. Global health action. 2015;8.
34. Hasan H, Zodpey S, Saraf A. Diabetes care in India: assessing the need for evidence-based education. South‐East Asian Journal of Medical Education, 2011;5(2):15–18.
35. Madhu SV, Lalitha K. Education needs of diabetic patients with low socio-economic and literacy levels. Presented at the 15th International Diabetes Federation (IDF) Congress, Kobe, Japan, 1994 (Abstract # 10C3OP1804, published in scientific supplement: p. 137).
36. Chandalia HB, Modi SV. Counseling strategies. J Obes Metab Res 2014;1:43–5.
37. Babu MS, Gowdappa HB, Kalpana T, Vidyalaxmi K, Nikhil B, Chakravarthy T. Knowledge, attitude and practices of diabetic patients—a cross sectional study in a tertiary care hospital in Mysore. The Journal of the Association of Physicians of India. 2015;63(8):96.
38. Siaw MY, Ko Y, Malone DC, Tsou KY, Lew YJ, Foo D, Tan E, Chan SC, Chia A, Sinaram SS, Goh KC. Impact of pharmacist‐involved collaborative care on the clinical, humanistic and cost outcomes of high‐risk patients with type 2 diabetes (IMPACT): a randomized controlled trial. Journal of Clinical Pharmacy and Therapeutics. 2017.

## **Oral antidiabetic agents**

### **RSSDI 2017 recommendations**

#### **Recommended care**

- Begin oral glucose-lowering medications when lifestyle interventions alone are unable to maintain blood glucose control at target levels
  - Maintain support for lifestyle measures throughout the use of these medications.
  - Consider each initiation or dose increase of oral glucose-lowering medications as a trial, monitoring the response through self-monitoring blood glucose (SMBG) or A1C in 2–3 months.
  - Consider cost and benefit risk ratio when choosing a medication (patient-centric approach should be used).
  - Consider discontinuing ineffective therapies.
  - Hypoglycemia, weight gain, and cost of therapy are also important parameters in deciding therapy.
- First-line therapy
  - Begin with metformin unless there is evidence of renal impairment or other contraindication.
  - Titrate the dose over early weeks to minimize discontinuation due to gastrointestinal intolerance.
  - Monitor renal function and use metformin with caution if estimated glomerular filtration rate (eGFR) < 45 mL/min/1.73 m^2^. Do not use if eGFR is < 30 mL/min.
  - Other options include a sulfonylurea (or glinide) for rapid response where glucose levels are high or a DPP4 inhibitor or AGI; these agents can also be used initially in place of metformin where it is not tolerated or contraindicated.
  - In some circumstances, dual therapy may be indicated initially if it is considered unlikely that single agent therapy will achieve glucose targets.
- Second-line therapy
  - When glucose control targets are not being achieved, a sulfonylurea or a thiazolidinedione or a SGLT2 inhibitor, or a DPP-4 inhibitor, or AGI can be added using a patient-centric approach.
- Third-line therapy
  - When glucose control targets are no longer being achieved, start insulin or add a third oral agent.
  - Options for third oral agent include an AGI, a DPP4 inhibitor, a SGLT2 inhibitor, or a thiazolidinedione (depending on second-line agent used).
  - From July 2014, hydroxychloroquine (HYQ) has been approved by DCGI as an “adjunct to diet and exercises to improve glycemic control in patients with T2DM on sulfonylurea and metformin combination”
- Fourth-line therapy
  - Begin insulin therapy when optimized oral blood glucose-lowering medications and lifestyle interventions are unable to maintain target glucose control.
  - Intensify insulin therapy if insulin is being used already.

#### **Limited care**

- The principles are as for recommended care taking particular note of cost and availability of generic therapies.

#### **Preamble**

The primary aims of controlling glycemic levels are to avoid acute symptoms of hyperglycemia, to avoid fluctuation in blood glucose over time, and to prevent/delay the development of various complications associated with diabetes without hampering QoL of patients. The treatment should also aim at preserving β-cell function and prevent or slow the rate of apoptosis that will in turn delay the natural progression of the disease. Particularly from a patient point of view stability of metabolic control over time may be another specific goal that needs to be considered. Given the progressive loss of β-cell function in T2DM, treatment with OADs is ensued if the target A1C is not achieved with initial lifestyle modification. However, the properties of any antidiabetic agent that play a role in the choice of drug(s) in individual patients may vary because diabetes itself has a different mechanism responsible for its pathophysiology. Several guidelines provide treatment algorithms on ways in which glucose-lowering agents can be used either alone or in combination. The current guideline based on the current clinical evidences provides overview on available OADs and tries to come up with some practically applicable recommendations for optimal management of T2DM in Asian Indians.

#### **Considerations**

The decision on choice of OAD therapy in T2DM patients was based on the cost, safety, and efficacy factors that were reviewed in Indian context.

#### **Rationale and evidence**

##### *Antidiabetic agents*

- *Biguanides*: Metformin remains the first-line drug in the management of patients with T2DM due to its efficacy and being weight neutral, economical, and devoid of major adverse effects such as hypoglycemia. Its mechanism of action predominately involves reducing hepatic glucose output [1–3]. Metformin is associated with initial gastrointestinal side effects, and caution needs to be taken to avoid its use in patients at risk for lactic acidosis (e.g., in advanced renal insufficiency, alcoholism). Even though some CV benefits from metformin have been noted, the clinical trial data are not robust.
- *Sulfonylureas*: They are the oldest class of OADs which are also known as insulin secretagogues. They show their effect by closure of ATP-sensitive potassium channels on β-cells, stimulating insulin release [4, 5]. Although they are effective in controlling glucose levels, their use is associated with modest weight gain and risk of hypoglycemia. Modern sulfonylureas particularly gliclazide modified release (MR) and glimepiride have a lower risk of hypoglycemia and are preferred to be used in south Asian T2DM patients [5]. Shorter-acting secretagogues, the meglitinides (or glinides), also stimulate insulin release through similar mechanisms but may be associated with comparatively less hypoglycemia [6] but they require more frequent dosing. Moreover, modern sulfonylureas exhibit more reductions of A1C than glinides [7].
- *Thiazolidinediones*: They are peroxisome proliferator activated receptor γ activators [8] that improve insulin sensitivity in skeletal muscle and reduce hepatic glucose production [1, 2]. The risk of hypoglycemia is negligible and may be more durable in their effectiveness than sulfonylureas and metformin [9, 10]. Pioglitazone appeared to have a pleotropic effects on cardiovascular events as a secondary outcome in one large trial involving patients with overt macrovascular disease [11]. Pioglitazone had been linked with a possible increased risk of bladder cancer [12]; however, a recent systematic review report that the association of bladder cancer with pioglitazone was not found to be significant [13]. Moreover, data from a retrospective study in India involving 2222 (pioglitazone users, *n* = 1111; pioglitazone non-users, *n* = 1111) T2DM patients found no evidence of bladder cancer in any of the group, including patients with age > 60 years, duration of diabetes > 10 years, and uncontrolled diabetes [14]. Recognized side effects of thiazolidinediones include weight gain, fluid retention leading to edema, and/or heart failure in predisposed individuals and patients with increased risk of bone fractures [9, 11, 13].
- *DPP4 inhibitors*: Drugs like vildagliptin, saxagliptin, gemigliptin, sitagliptin, teneligliptin, and linagliptin are incretin enhancers; they enhance circulating concentrations of active GLP-1 and gastric intestinal polypeptide (GIP) [15, 16]. Their major effect is the regulation of insulin and glucagon secretion; they are weight neutral. Furthermore, recent CV studies with DPP-4 inhibitors have shown that these agents do not increase the CV risk [17–19]. Recent studies have reported a higher incidence of heart failure hospitalization with saxagliptin and alogliptin [17, 18]. However, the reason for the same is not clear and the primary outcomes of non-fatal MI, non-fatal stroke, and CV death were not different in intervention arm as compared to placebo arm. Sitagliptin showed no difference in the rate of hospitalization for heart failure [20]. The other DPP4 inhibitors as yet do not have outcome studies published.
- Typically, none of the incretin-based classes cause hypoglycemia by themselves. Teneligliptin is a new agent in the class of DPP-4 inhibitor reported to be effective, safe, and well tolerated in Indian T2DM patients as monotherapy or in combination [21, 22]. However, data available for teneligliptin is limited and there are no RCTs available for teneligliptin.
- *SGLT2 inhibitors*: They provide insulin-independent glucose-lowering by blocking glucose reabsorption in the proximal renal tubule by inhibiting SGLT2 receptor [23]. These agents provide modest weight loss and BP reduction when used as monotherapy [23, 24]. Dapagliflozin, canagliflozin, and empagliflozin are the three Food and Drug Administration (FDA)-approved agents used in patients with T2DM [23]. SGLT2 inhibitors have the potential to reduce CV risk in patients with T2DM [25, 26] not only through beneficial effects on glycemic control but also via beneficial effects on body weight, BP, lipids, and serum uric acid [27, 28]. SGLT2 inhibitors significantly reduce BP in patients with T2DM [29, 30]. Nonetheless, SGLT-2 inhibitors are associated with an increased risk of genital infection [31].
- *AGIs*: Acarbose, voglibose, and miglitol are the AGIs which retard gut carbohydrate absorption [32, 33] reduce postprandial hyperglycemia. The main adverse effects are gastrointestinal, flatulence, distention, nausea, and diarrhea. Moreover, a recent systematic review and meta-analysis report that AGIs might associate with increased risk of hepatotoxicity. However, the evidence is limited and no clinically important AEs were observed [34].
- *Colesevelam*, a bile acid sequestrant whose mechanism of glucose-lowering action remains poorly understood and whose major additional benefit is LDL-C reduction [35, 36], is used infrequently in treatment course of diabetes. In a pilot study of colesevelam HCl 3.75 g/day in patients with T2DM, LDL-C and total cholesterol were decreased by 11.7 and 7.8%, respectively, and also A1C was decreased by 0.5% [37]. Adverse effects mainly observed were constipation and GI discomfort.
- *Hydroxychloroquine (HYQ)*: Recently, HYQ has been approved by DCGI in the management of T2DM in India. It has a modest effect on reducing A1C along with reduction of pro-inflammatory markers. It was shown to reduce white blood cell (WBC) count and increase adiponectin in many studies [38, 39]. When used in patients with rheumatoid arthritis, it has shown to delay new onset diabetes, a promising action helpful in prevention [40]. Moreover, in Indian patients with uncontrolled T2DM, significant difference in glycemic efficacy was not found between HYQ and pioglitazone [41]. Several ongoing trials will throw more light in days to come. Adverse effects are mainly gastrointestinal, nausea, vomiting. Blurring of vision, retinopathy, and maculopathy are uncommon if the recommended daily dose is not exceeded [42, 43].
- The dopamine agonist bromocriptine (quick release formulation) is also available as an antihyperglycemic agent and supposedly acts by mimicking the morning surge of dopamine [44, 45]. Its mechanism of action and precise role are unclear.
- The glucose-lowering effectiveness of OADs is said to be high with metformin, sulfonylureas, and thiazolidinedione (expected A1C reduction ~ 1.0–1.5%) and comparatively lower for meglitinides, DPP4 inhibitor, SGLT2 inhibitor, AGIs, HYQ, colesevelam, and bromocriptine (~ 0.5–1.0%) [10, 46–49]. However, older drugs have typically been tested in clinical trial participants with higher baseline A1C, which is associated with greater treatment emergent glycemic reductions, irrespective of therapy type. In head-to-head trials, any differential effects on glucose control between different OADs are small. So agent- and patient-specific properties, such as ease of administration, dosing frequency, side effect profiles, cost, and other benefits, often help in their selection.
- Two-drug combination therapies with metformin (such as metformin plus thiazolidinediones, metformin plus sulfonylureas, metformin plus SGLT2 inhibitors, and metformin plus DPP4 inhibitors) were more effective in reducing A1C than metformin monotherapy by about 1% [10]. In addition, triple FDC of metformin and sulfonylurea plus pioglitazone or voglibose are also available in India. Even though they have to be administered with caution and there is some ambiguity regarding their timing of administration, several studies have proved their efficacy in Indian population [50].
- RSSDI wheel given along with this recommendation book will help a practitioner choose an ideal drug for his patient based on cost, weight, hypoglycemia risk, and other comorbid conditions

**References**

1. Bailey CJ, Turner RC. Metformin. N Engl JMed. 1996;334(9):574–9.
2. Lamanna C, Monami M, Marchionni N, Mannucci E. Effect of metformin on cardiovascular events and mortality: a meta-analysis of randomized clinical trials. Diabetes Obes Metab. 2011;13(3):221–8.
3. Atal S, Atal S, Vyas S, Phadnis P. Bio-enhancing effect of Piperine with Metformin on lowering blood glucose level in Alloxan induced diabetic mice. Pharmacognosy research. 2016;8(1):56.
4. Bryan J, Crane A, Vila-Carriles WH, Babenko AP, Aguilar-Bryan L. Insulin secretagogues, sulfonylurea receptors and K(ATP) channels. Curr Pharm Des. 2005;11(21):2699–716.
5. Kalra S, Aamir AH, Raza A, Das AK, Khan AA, Shrestha D, Qureshi MF, Fariduddin M, Pathan MF, Jawad F, Bhattarai J. Place of sulfonylureas in the management of type 2 diabetes mellitus in South Asia: a consensus statement. Indian journal of endocrinology and metabolism. 2015;19(5):577.
6. Gerich J, Raskin P, Jean-Louis L, Purkayastha D, Baron MA. PRESERVE-β: two-year efficacy and safety of initial combination therapy with nateglinide or glyburide plus metformin. Diabetes Care 2005;28:2093–9.
7. Guardado-Mendoza R, Prioletta A, Jiménez-Ceja LM, Sosale A, Folli F. The role of nateglinide and repaglinide, derivatives of meglitinide, in the treatment of type 2 diabetes mellitus. Archives of Medical Science: AMS. 2013;9(5):936.
8. Yki-Jarvinen H. Thiazolidinediones. N Engl J Med 2004;351(11):1106–18.
9. Kahn SE, Haffner SM, Heise MA, Herman WH, Holman RR, Jones NP et al. Glycemic durability of rosiglitazone, metformin, or glyburide monotherapy. N Engl J Med 2006;355:2427–43.
10. Bolen S, Tseng E, Hutfless S, Segal JB, Suarez-Cuervo C, Berger Z, Wilson LM, Chu Y, Iyoha E, Maruthur NM. Diabetes medications for adults with type 2 diabetes: an update 2016. Available at https://www.ncbi.nlm.nih.gov/books/NBK362863/pdf/Bookshelf_NBK362863.pdf
11. Dormandy JA, Charbonnel B, Eckland DJ, Erdmann E, Massi-Benedetti M, Moules IK et al. Secondary prevention of macrovascular events in patients with type 2 diabetes in the PROactive study (PROspective pioglitAzone Clinical Trial In macroVascular Events): a randomised controlled trial. Lancet 2005;366(9493):1279–89.
12. Lewis JD, Ferrara A, Peng T, Hedderson M, Bilker WB, Quesenberry CP Jr. et al. Risk of bladder cancer among diabetic patients treated with pioglitazone: interim report of a longitudinal cohort study. Diabetes Care 2011;34(4):916–22.
13. Pai SA, Kshirsagar NA. Pioglitazone utilization, efficacy & safety in Indian type 2 diabetic patients: a systematic review & comparison with European Medicines Agency Assessment Report. The Indian Journal of Medical Research. 2016;144(5):672.
14. Gupta S, Gupta K, Ravi R, Mehta V, Banerjee S, Joshi S, Saboo B. Pioglitazone and the risk of bladder cancer: an Indian retrospective cohort study. IJEM. 2015;19(5):639–43.
15. Deacon CF. Dipeptidyl peptidase-4 inhibitors in the treatment of type 2 diabetes: a comparative review. Diabetes Obes Metab 2011;13(1):7–18.
16. Chawla S, Kaushik N, Singh NP, Ghosh RK, Saxena A. Effect of addition of either sitagliptin or pioglitazone in patients with uncontrolled type 2 diabetes mellitus on metformin: a randomized controlled trial. J Pharmacol Pharmacother. 2013;4(1):27–32.
17. Scirica BM, Bhatt DL, Braunwald E, Steg PG, Davidson J, Hirshberg B, et al. Saxagliptin and cardiovascular outcomes in patients with type 2 diabetes mellitus. N Engl J Med. 2013;369(14):1317–26.
18. White WB, Cannon CP, Heller SR, Nissen SE, Bergenstal RM, Bakris GL, et al. Alogliptin after acute coronary syndrome in patients with type 2 diabetes. N Engl J Med. 2013;369(14):1327–35.
19. TECOS: Sitagliptin Cardiovascular Outcome Study. ClinicalTrials.gov identifier: NCT00790205. Available at: http://clinicaltrials.gov/ct2/show/NCT00790205.(Last accessed on 27 Aug 2015).
20. Green JB, Bethel MA, Armstrong PW, Buse JB, Engel SS, Garg J, Josse R, Kaufman KD, Koglin J, Korn S, Lachin JM. Effect of sitagliptin on cardiovascular outcomes in type 2 diabetes. New England Journal of Medicine. 2015;373(3):232–42.
21. Mohammed R, Ahmed I, Banu A. Efficacy of teneligliptin in type 2 diabetes mellitus. International Journal of Research in Medical Sciences. 2016;4(10):4607–10.
22. Ghosh S, Trivedi S, Sanyal D, Modi KD, Kharb S. Teneligliptin real-world efficacy assessment of type 2 diabetes mellitus patients in India (TREAT-INDIA study). Diabetes, Metabolic Syndrome and Obesity: Targets and Therapy. 2016;9:347.
23. Wong EY, A review of sodium glucose co-transporter 2 (SGLT2) inhibitors for type 2 diabetes mellitus. Pharm Pharmacol Int J 2016, 4(2): 00070
24. DeFronzo RA, Norton L, Abdul-Ghani M. Renal, metabolic and cardiovascular considerations of SGLT2 inhibition. Nature Reviews Nephrology. 2017;13(1):11–26.
25. Neal B, Perkovic V, Mahaffey KW, de Zeeuw D, Fulcher G, Erondu N, Shaw W, Law G, Desai M, Matthews DR. Canagliflozin and cardiovascular and renal events in type 2 diabetes. New England Journal of Medicine. 2017.
26. Zinman B, Wanner C, Lachin JM, Fitchett D, Bluhmki E, Hantel S, Mattheus M, Devins T, Johansen OE, Woerle HJ, Broedl UC. Empagliflozin, cardiovascular outcomes, and mortality in type 2 diabetes. New England Journal of Medicine. 2015;373(22):2117–28.
27. Basile JN. The potential of sodium glucose cotransporter 2 (SGLT2) inhibitors to reduce cardiovascular risk in patients with type 2 diabetes (T2DM). J Diabetes Complications. 2013;27(3):280–6.
28. Liu XY, Zhang N, Chen R, Zhao JG, Yu P. Efficacy and safety of sodium-glucose cotransporter 2 inhibitors in type 2 diabetes: a meta-analysis of randomized controlled trials for 1 to 2 years. Journal of Diabetes and its Complications. 2015;29(8):1295–303.
29. Baker WL, Smyth LR, Riche DM, Bourret EM, Chamberlin KW, White WB. Effects of sodium-glucose co-transporter 2 inhibitors on blood pressure: a systematic review and meta-analysis. J Am Soc Hypertens. 2014;8(4):262–75.e9.
30. Reed JW. Impact of sodium–glucose cotransporter 2 inhibitors on blood pressure. Vascular Health and Risk Management. 2016;12:393.
31. Zaccardi F, Webb DR, Htike ZZ, Youssef D, Khunti K, Davies MJ. Efficacy and safety of sodium-glucose co-transporter-2 inhibitors in type 2 diabetes mellitus: systematic review and network meta-analysis. Diabetes Obes Metab. 2016;18(8):783–94.
32. Van de Laar FA, Lucassen PL, Akkermans RP, van de Lisdonk EH, de Grauw WJ. Alpha-glucosidase inhibitors for people with impaired glucose tolerance or impaired fasting blood glucose. Cochrane Database Syst Rev. 2006;(4):CD005061.
33. Cai X, Han X, Luo Y, Ji L. Comparisons of the efficacy of alpha glucosidase inhibitors on type 2 diabetes patients between Asian and Caucasian. PLOS One. 2013;8(11):e79421.
34. Zhang L, Chen Q, Li L, Kwong JS, Jia P, Zhao P, Wang W, Zhou X, Zhang M, Sun X. Alpha-glucosidase inhibitors and hepatotoxicity in type 2 diabetes: a systematic review and meta-analysis. Scientific Reports. 2016;6.
35. Sekhri K, Saha L. Colesevelam hydrochloride: a novel agent in patients with type 2 diabetes. International Journal of Applied and Basic Medical Research. 2011 Jul;1(2):113.
36. Fonseca VA, Handelsman Y, Staels B. Colesevelam lowers glucose and lipid levels in type 2 diabetes: the clinical evidence. Diabetes Obes Metab 2010;12(5):384–92.
37. Zieve FJ, Kalin MF, Schwartz SL, Jones MR, Bailey WL. Results of the glucose-lowering effect of WelChol study (GLOWS): a randomized, double-blind, placebo-controlled pilot study evaluating the effect of colesevelam hydrochloride on glycemic control in subjects with type 2 diabetes. Clin Ther. 2007;29(1):74–83. PubMed PMID: 17379048.
38. Sames E, Paterson H, Li C. Hydroxychloroquine-induced agranulocytosis in a patient with long-term rheumatoid arthritis. European Journal of Rheumatology. 2016;3(2):91.
39. Wasko MC, McClure CK, Kelsey SF, Huber K, Orchard T, Toledo FG. Antidiabetogenic effects of hydroxychloroquine on insulin sensitivity and beta cell function: a randomised trial. Diabetologia. 2015;58(10):2336–43.
40. Wasko MC, Hubert HB, Lingala VB, Elliott JR, Luggen ME, Fries JF, Ward MM. Hydroxychloroquine and risk of diabetes in patients with rheumatoid arthritis. JAMA. 2007;298(2):187–93.
41. Pareek A, Chandurkar N, Thomas N, Viswanathan V, Deshpande A, Gupta OP, Shah A, Kakrani A, Bhandari S, Thulasidharan NK, Saboo B. Efficacy and safety of hydroxychloroquine in the treatment of type 2 diabetes mellitus: a double blind, randomized comparison with pioglitazone. Current medical research and opinion. 2014;30(7):1257–66.
42. Melles RB, Marmor MF. The risk of toxic retinopathy in patients on long-term hydroxychloroquine therapy. JAMA ophthalmology. 2014;132(12):1453–60.
43. Yam JC, Kwok AK. Ocular toxicity of hydroxychloroquine. Hong Kong Medical Journal. 2006;12(4):294.
44. Weiland CM, Hilaire ML. Bromocriptine mesylate (Cycloset) for type 2 diabetes mellitus. American Family Physician. 2013;87(10):718.
45. Defronzo RA. Bromocriptine: a sympatholytic, D2-dopamine agonist for the treatment of type 2 diabetes. Diabetes Care 2011;34(4):789–94.
46. Bolen S, Feldman L, Vassy J Wilson L, Yeh HC, Marinopoulos S et al. Systematic review: comparative effectiveness and safety of oral medications for type 2 diabetes mellitus. Ann Intern Med 2007;147(6):386–99.
47. Peters A. Incretin-based therapies: review of current clinical trial data. Am J Med 2010;123:S28–37.
48. Bennett WL, Maruthur NM, Singh S, Segal JB, Wilson LM, Chatterjee R et al. Comparative effectiveness and safety of medications for type 2 diabetes: an update including new drugs and 2-drug combinations. Ann Intern Med 2011;154(9):602–13.
49. Thomsen RW, Baggesen LM, Søgaard M, Pedersen L, Nørrelund H, Buhl ES, Haase CL, Johnsen SP. Early glycaemic control in metformin users receiving their first add-on therapy: a population-based study of 4,734 people with type 2 diabetes. Diabetologia. 2015;58(10):2247–53.
50. John M, Gopinath D, Kalra S. Triple fixed drug combinations in type 2 diabetes. Indian Journal of Endocrinology and Metabolism. 2015;19(3):311.

## **Injectables**

### **RSSDI 2017 recommendations**

#### **Insulin therapy**

- A three-step protocol involving initiation, titration, and intensification is recommended for all patients requiring insulin.

#### **Initiation**

- “Providers should avoid using insulin as a threat or describing it as a sign of personal failure or punishment” [1].
- Ensure timely commencement of insulin.
- Even though triple oral therapy may be effective, patients taking two OADs and having an A1C > 8.0% and/or long-standing T2DM are less likely to achieve their glycemic goals with a third OAD. Even if glycemic goals are achieved, there is often limited durability of glycemic control. Therefore, consider initiating insulin in patients with uncontrolled or symptomatic or complicated T2DM.
- Individuals with symptomatic hyperglycemia and metabolic decompensation should receive an initial antihyperglycemic regimen containing insulin with or without metformin.
- The therapeutic choice of regimen, preparation, and delivery device should be made through a process of shared, informed decision-making, by both patient and physician.
- Initiate with once daily basal insulin, once daily premixed/co-formulation insulin, or twice daily premixed insulin, either alone or in combination of GLP-1 analogues (either alone or in combination with basal insulin, in same pen device) or in combination with other OADs, based upon clinical features, glucose profile, risk of hypoglycemia, and patient preference
- Basal bolus insulin regimens may be needed in severe hyperglycemia and in life-threatening or organ/limb-threatening clinical situations.
- Analogue insulins may be used in preference to human insulins with possible lower risk of nocturnal and symptomatic hypoglycemia; however, economic considerations must be taken into account.
- Newer longer acting basal insulins [IDeg U100/IGlar U300] and co-formulations [IDegAsp] may have lesser hypoglycemia.
- Match the timing of insulin and meals.
- Counselling/education about SMBG and hypoglycemia prevention, recognition, and treatment is recommended to all patients initiating with insulin. Provide guidance for adjusting insulin dose adjustments, administration, storage, and other practical aspects.

#### **Titration**

- Initiate insulin as defined in the algorithm, using a self-titration regimen (dose increases of 2–4 units (U) every 3 days or biweekly) or with more frequent contact with a healthcare professional.
- Aim for premeal glucose levels of < 115 mg/dL and PPG levels of 140–180 mg/dL. These targets can be individualized, based upon the risk of hypoglycemia and the urgency for glycemic control
- Titration should be done at regular and short intervals, to attain glycemic goals without causing hypoglycemia.
- Titration can be carried out as guided by the physician, trained paramedical staff, or by the patient/caregivers who have been educated and empowered.

#### **Intensification**

- Intensification of insulin therapy is recommended when patients fail to achieve glycemic goals even after optimal dose titration.
- Several options can be considered during intensification:
  - Switch to premix insulin twice daily or (rarely) thrice daily
  - Use high mix insulins or adopt a heteromix insulin regimen
  - Switch to insulin co-formulation-based regimen
  - Add prandial insulin (basal plus or basal bolus) with largest meal of the day
  - Add GLP-1 analogues
- The choice of intensification strategy should be based upon dietary pattern, lifestyle, gluco-phenotype, risk of hypoglycemia and weight gain, affordability, as well as patient preference.
- Basal plus regimen can be used as a stepwise approach to insulin intensification, leading to basal-bolus prescription. It is associated with lesser risk of hypoglycemia and weight gain than basal bolus regimen.
- Both premix insulin therapy and co-formulation insulins are acceptable methods of intensification. Co-formulation insulin offers the advantage of lower risk of hypoglycemia and nocturnal hypoglycemia.
- Follow insulin intensification as recommended in the algorithm VIII.

#### **GLP-1 analogues**

- GLP-1 analogues are viable second-line or third-line options for the management of patients with uncontrolled hyperglycemia.
- GLP-1 analogues can be considered in overweight/obese patients as second-line therapy in patients with metformin inadequacy.
- GLP-1 RAs can be considered in overweight/obese patients as first-line therapy in patients with metformin intolerance.
- GLP-1 analogues can be added to insulin therapy if glycemic goals are not achieved with reasonably high doses of insulin or if unacceptable weight gain or hypoglycemia occurs. Dose reduction of insulin may be needed in such cases. Transient gastrointestinal side effects may occur.
- In patients with T2DM, antihyperglycemic agents with proven CV benefit should be considered to reduce the risk of CV events. Liraglutide has been indicated by FDA “as an adjunct to standard treatment of CV risk factors to reduce the risk of major adverse CV events (CV death, non-fatal MI, or non-fatal stroke) in adults with T2DM and high CV risk” [2].

#### **Limited care**

- Less expensive human insulins (regular insulin, neutral protamine Hagedorn (NPH) insulin, and conventional premixed human insulin) are able to achieve most of the healthcare gains with insulin therapy.
- Insulin supplies should be assured and be of consistent quality and type.

#### **Comprehensive care**

Insulin pump: Sufficient evidence from randomized controlled studies to support the use of insulin pump in patients with T2DM is lacking; however, it is a potential option in selected individuals [3]. Nonetheless, longitudinal data suggest that insulin pumps may be useful in patients with severe IR and poor glycemic control despite adhering to recommended insulin therapy, diet, and exercise [4]. Furthermore, pump therapy in appropriate patients may reduce risk of hypoglycemia and weight gain [4].

#### **Preamble**

Type 2 diabetes mellitus is a progressive disease in which patients nearly lose > 50% of their β-cell function by the time of diagnosis. Furthermore, this loss steadily continues to decline at approximately 3–5% per year [5, 6]. Intensive glycemic management has reduced the mortality, morbidity, and risk of diabetes-related complications in patients with T2DM [6, 7]. Most of the medications available to control glycemia have a final common pathway in the form of targeting β-cells or IR and are dependent upon the presence of insulin for their therapeutic effect. The durability of these medications varies and their safety is occasionally under scrutiny. Over a period, patients fail to achieve or maintain A1C levels even with multiple OADs and will require insulin therapy.

Most guidelines recommend early insulin therapy in patients with high A1C at the time of presentation [1, 8, 9]. The knowledge available through landmark trials in last decade warrants that glycemic control should be intensive in early stages of diabetes, preferably in first 4 years of diagnosis of diabetes [10–12]. This makes us understand that the indications of insulin therapy in T2DM should be more proactive, albeit not at the cost of severe symptomatic hypoglycemia, especially in patients where hypoglycemia may be deleterious.

The traditional postponement of insulin therapy up to prolonged failure of lifestyle and oral agents to achieve glycemic control has been revised in the last decade to incorporate insulin therapy much earlier, often in combination with OADs or GLP-1 analogues. All healthcare professionals and primary care physicians must understand the significance of legacy effect that was very clearly demonstrated in the long-term cohorts of UK Prospective Diabetes Study (UKPDS) trial [6]. An Indo-centric way of defining legacy effect is metabolic karma [14].

Non-insulin injectables such as GLP-1 analogues and amylin analogues (pramlintide) have been approved in various countries. The GLP-1 analogues improve glycemic control through multiple mechanisms, with low risk of hypoglycemia and clinically relevant weight loss [13]. As pramlintide is not available in India, it is not covered in these recommendations.

#### **Considerations**

The decision on choice of injectable therapy in T2DM patients is based on clinical, pharmacological, as well as psychosocial factors. Apart from these, local factors such as cost, quality, cold chain maintenance, and perennial availability of insulin preparations as well as delivery devices must be considered in the Indian context.

## **Rationale and evidence**

### **Insulin therapy**

- Due to the progressive nature of diabetes and β-cell dysfunction, insulin replacement therapy is frequently required in T2DM patients [5, 7]. Optimally, insulin aims to create a normal glycemic profile as possible without unacceptable weight gain or chances of hypoglycemia [15, 16].
- A current National Insulin Summit (NIS) consensus from India reports that insulin therapy is the most effective antidiabetic agent and can reduce A1C by 1.5–3.5% from baseline when used as monotherapy in T2DM patients, whereas other antidiabetic agents can only reduce A1C to a range of 0.5–2.0% [16]. However, most patients and physicians are reluctant to initiate insulin early due to fear of injection, hypoglycemia, weight gain, and other complications [18].
- *Indications of Insulin in T2DM in newly detected patients*:
  - Those patients, who at the time of diagnosis are symptomatic, have one of the following:
    - A1C > 9%
    - FPG > 250 mg/dL
    - PPG > 300 mg/dL
    - Positive ketonuria
    - Catabolic status
    - Pregnant or planning pregnancy
  - When patient presents with metabolic/cardiovascular/medical/surgical/obstetric crisis, insulin is safer as well as more effective.
    - In these cases, insulin may be started as monotherapy or with metformin if the latter is not contraindicated and is well tolerated.
    - As the patient’s glucose toxicity resolves, the regimen can potentially be de-escalated, and a switch over to oral therapy may be considered.
- *Indications of insulin in T2DM in patients with already established diagnosis*:
  - If a trial of adequate doses of two to three non-insulin agents for 3–6 months fails to achieve A1C to target levels, or if organ dysfunction contraindicates use of oral agents, addition of insulin may be justified as the landmark studies suggest that achieving intensive glycemic control (if not contraindicated) in initial few years of diagnosis is of profound benefit.
    - Individualization of therapy requires taking several factors into consideration, including an assessment of the patient’s risk for hyperglycemia and related complications vs the risks of therapy, presence of comorbid conditions, assessment of psychological status, capacity for self-care, economic considerations, and family and social support systems. Lifestyle modification including medical nutrition therapy, exercise, smoking cessation, and stress management must be promoted at all times.
    - A1C targets must be determined as per criteria set for individualized therapy [16] and efficacy of each agent of reducing A1C as combination therapy must be considered.
    - Near-normal glycemic targets should be considered for younger patients with recent onset of T2DM and few or no micro- or macrovascular complications, while slightly higher A1C targets may be considered for older patients with long-standing T2DM and evidence of CVD [17].
    - If a patient already on two or more agents and continues to have A1C in excess of 9%, insulin may be initiated even if patient is asymptomatic.
    - While initiating insulin, doses of sulfonylurea should be reduced and a strict watch must be kept on hypoglycemia.
    - It should be explained to the patient during every visit following diagnosis of diabetes that insulin is one of the options available to manage their diabe-tes and that it may turn out to be the best and eventually necessary way of maintaining glucose control, especially in the longer term.
    - Adequate doses of oral agents do not necessarily mean the highest administrable doses because, in most of the cases, doubling the doses of these medicines does not necessarily increment their effects.
- *The insulin strategy*:
  - While starting the insulin therapy, the following features have to be considered in a sequential order: choosing the right regimen, identifying the appropriate preparation, prescribing the available strength of the preparation, matching it with the correct delivery device, deciding the proper insulin dose, and following the optimal titration strategy.
  - Ideally, an insulin treatment program should be designed specifically for an individual patient, to match the supply of insulin to his or her dietary/exercise habits and prevailing glucose trends, as revealed through self-monitoring. Anticipated glucose-lowering effects should be balanced with the convenience of the regimen, in the context of an individual’s specific therapy goals.
- *Patient education*:
  - Proper patient education regarding monitoring of glucose, insulin injection technique, insulin storage, recognition/ treatment of hypoglycemia, and sick day management is imperative. Where available, certified diabetes educators can be invaluable in guiding the patient through treatment course of diabetes.
- *Adverse events and barriers*:
  - Hypoglycemia is a major safety concern with insulin treatment. It is considerably prevalent in patients with T2DM who are on insulin treatment and also fairly common with usage of other antidiabetic agents [18].
  - Weight gain may occur with insulin therapy. However, evidence from a study in T2DM patients, evaluating the effect of different treatment modalities on weight gain indicate that sulfonylurea + insulin was associated with significant weight gain followed by insulin group, sulfonylurea group, and sulfonylurea + metformin + insulin group, and sulfonylurea + metformin group. Weight gain due to treatment was significantly related to pretreatment weight loss and patients with improved metabolic control tend to attain stable body weight [19]. A recent Cochrane review reports that combination therapy of insulin with sulfonylurea or pioglitazone resulted in additional weight gain compared to insulin monotherapy. Combination with DPP4 inhibitors resulted in weight neutrality and combination with metformin or AGIs produced weight loss compared to insulin monotherapy [20].
  - Various barriers to insulin use prevail in society. A recent National Insulin Summit (NIS) consensus lists the barriers to insulin therapy as related to patient/community, physician/provider, and drug/device and proposes different bridges to overcome these hurdles. Patient-related barriers such as inability to inject, monitor, or titrate the insulin dose, weight gain, hypoglycemia, and lack of awareness of uncontrolled diabetes can be bridged with patient education and training, support and counselling, and social marketing. Physician and provider barriers such as inadequate communication or motivation skills, inability to initiate, optimize or intensify insulin, and lack of awareness may be addressed through relevant skill development training, and continuing medical education (CME). Furthermore, drug- or device-specific barriers such as suboptimal effects of insulin, lack of flexibility, and device discomfort can be surmounted through CME, flexible insulin regimens, and preparations and modern devices [21].
- *Initiation of insulin therapy (Annexure VI)*:
  - As initial therapy, unless the patient is passing through an acute medical, surgical, or obstetric crisis, or in metabolic decompensation, premixed or basal insulin is typically initiated [1, 3, 8, 22]. The general concept is to first correct the fasting hyperglycemia with a dinner/bed time injection and then address postprandial hyperglycemia.
  - Choice of initial insulin is often dictated by subjective features such as disease severity and ability of the patient to self-inject at specific times of the day. Even though FPG and PPG measurements together provide sufficient information to choose an insulin type, it is difficult to make an appropriate decision when they are considered separately. Similarly, choice of insulin on the basis of A1C value alone can be challenging. Therefore, to facilitate an objective rationale for the physicians, the following simple ratios could be helpful: ratio of prandial and FPG index ((PPG-FPG)/FPG), ratio of FPG to A1C, and ratio of 1,5-anhydroglucitol and A1C [23].
  - Recommendations from guidelines:
    - American Association of Clinical Endocrinologists and American College of Endocrinology (AACE/ACE) and ADA/European Association for the Study of Diabetes (EASD) guidelines recommend initiating insulin therapy with basal insulin. The IDF recommends initiation of insulin with either basal insulin (Annexure VII) or premixed insulin (Annexure VIII) when combination of oral therapies fails to achieve glycemic target of A1C < 7.0% [1, 3, 8, 24]. The JAPI 2017 consensus recommends both basal and premix insulin at the initiation, with the decision based on glycemic profile (FPG and PPG) of the patients [7, 25]. However, Indian National Consensus Group (INCG) 2013 recommends only premixed insulin at the initiation since PPG response to a meal is more pronounced in ethnic Asian communities [26].
    - The INCG recommends initiation of insulin in newly diagnosed patients with FPG > 250 mg/dL, PPG > 300 mg/dL, and A1C > 9% or if patient fails on maximal tolerated/optimal doses of two or three OADs. If A1C levels are between > 7 and ≤ 7.5 % after initial treatment with metformin, guidelines recommend initiation of second OAD/GLP-1 analogues. However, if A1C levels still remain above 7% after 3 months of dual therapy, the guidelines recommend addition of premixed insulin once or twice daily to metformin therapy. If A1C levels are > 7.5 and ≤ 8.5%, it recommends addition of premixed insulin once daily to initial metformin therapy. It recommends titration of premixed insulin therapy from once to twice daily, if A1C levels remain above 8.5%. Similarly, A1C levels above 7% and FPG > 100 mg/dL require titration of premixed insulin once/twice daily till FPG levels < 100 mg/dL [26].
    - A recent consensus published in JAPI 2017 recommends that premix insulin analogues may be preferred over human premix insulins due to the lower incidence of major and nocturnal hypoglycemia and flexibility of administration. However, IDegAsp may be preferred over premix insulin analogues in view of possible lower incidence of overall and nocturnal hypoglycemia and superior fasting plasma control [27].
  - Basal insulin:
    - Basal insulin provides relatively uniform 24-h insulin coverage, which controls blood glucose by suppressing hepatic glucose production in between meals and during sleep. Either intermediate-acting (NPH), long-acting (insulin glargine (IGlar), insulin detemir (IDet), or ultra-long acting insulin degludec (IDeg) formulations may be used. The latter three are associated with less overnight hypoglycemia than NPH and possibly slightly less weight gain [28–31]. The dose requirement of these basal insulin analogues may differ. Most comparative trials show a higher average unit requirement with IDet compared to IGlar [32, 33]. Initiation of IDeg, as compared to IGlar, may be associated with significantly lesser patient-reported hypoglycemic episodes and lesser dose of insulin requirement while achieving similar glycemic control among patients with T2DM [34].
    - Basal insulin can be started at a daily dose, preferably at bed time, of 10 U/day or 0.1 to 0.2 U/kg body weight/day when A1C < 8.0% or 0.2 to 0.3 U/kg body weight/day when A1C < 8.0%. If glycemic goals are not attained, titrate 10–15% or 2–4 U every 2–3 days. Alternatively titration can also be done on the basis of FPG as follows: add 20% of total daily dose (TDD) or 4 U when FPG > 180 mg/dL, add 10% of TDD or 2 U when FPG is between 140 and 180 mg/dL, and add 1 U when FPG is between 110 and 139 mg/dL. When hypoglycemia is reported, reduce the dose by 4 U or 10–20%. Additionally, dose reduction during hypoglycemia on the basis of blood glucose levels is recommended as follows: reduce by 10–20% when blood glucose < 70 mg/dL and reduce 20–40% when blood glucose < 40 mg/dL. Consider discontinuing or reducing the dose of sulfonylurea when basal insulin is initiated, as hypoglycemia risk is high when both are used together.
  - Premix insulin:
    - Premix insulin can be started once daily with 10 U either in the morning if predinner glucose is high or in the night, if the prebreakfast glucose is high. If a patient on biphasic insulin aspart (BIAsp) 30 once or twice daily has within-target FPG but an A1C > 7%, a switch to BIAsp 30 twice or thrice daily should be considered. If their FPG is above target, the dose should be titrated to achieve FPG 72–108 mg/dL; however, if hypoglycemia occurs, an additional daily dose should be added rather than further dose titration [25, 27, 35]. When the daily insulin dose in once daily regimen exceeds 20 U, intensify the regimen to twice daily such that the dose is distributed as two third in morning and one third in evening. However, when the single dose exceeds 30 U, the dose can be split into two equal doses, which reduces the chance of hypoglycemia. Also, premix insulin may be started twice daily in case of patients with higher A1C, or if blood glucose control is suboptimal [26].
    - A current systematic review including several RCTs in Asian patients reports that premix insulin is associated with a mean change of A1C of − 0.12 to − 4.2% from baseline to endpoint (improvement was generally more pronounced with insulin initiation vs intensification). Moreover, the efficacy and safety outcomes for premixed insulin analogues are similar to those for basal or basal-bolus insulin [36].
- *Intensification of insulin therapy (Annexure IX)*:
  - Although most of the patients with T2DM requiring insulin therapy can be successfully treated with one or two doses of insulin, some, because of progressive diminished in their insulin secretory capacity, will require prandial insulin therapy as well. This is typically achieved with regular insulin administered about 30 min before meals or rapid insulin analogues such as insulin lispro (ILis), insulin aspart (IAsp), or insulin glulisine (IGlu), which can be injected just before or with the meal. They result in better PPG control than human regular insulin.
  - Recommendations from guidelines:
    - The INCG 2013 and the JAPI 2014 recommend to intensify premix insulin to twice and thrice daily if A1C > 7% and FPG > 110 mg/dL [5, 26].
    - Similarly NIS 2016 recommends that in unmet needs of glycemic control with premix/basal insulin, twice daily IDegAsp should be preferred over premix insulin analogues for intensification. Furthermore, an analogue-based basal-bolus regimen may be preferred (if cost is not an issue) over human basal-bolus regimen and IDeg over IGlarg due to reduced risk of nocturnal hypoglycemia in patients with T2DM [37].
  - Basal insulin:
    - Basal insulin needs intensification when the PPG > 140 mg/dL and A1C > 7% irrespective of normal FPG < 100 mg/dL. Moreover, patients who are not at target (A1C < 7%) with high doses of basal insulin (> 0.5 U/kg) or those who are at increased risk of nocturnal hypoglycemia need further insulin intensification [7].
  - Premix insulin:
    - A combined analysis of two-phase III RCTs reports that IDegAsp twice daily compared to BIAsp 30 twice daily resulted in similar glycemic control with a lower risk of nocturnal hypoglycemia (*p* = 0.0001) in patients with T2DM previously treated with insulin [38].
    - Moreover, a 26-week randomized treat-to-target trial in Asian T2DM patient reports that IDegAsp effectively improved long-term glycemic control and provided superior reductions in FPG with a lower dose and numerically less nocturnal hypoglycemia compared to BIAsp 30 [39].

#### **GLP-1 analogues**

- The injectable GLP-1 analogues like liraglutide, exenatide, lixisenatide, dulaglutide, and albiglutide imitate the effects of endogenous GLP-1, thereby stimulating pancreatic insulin secretion in a glucose-dependent fashion, suppressing pancreatic glucagon output, slowing gastric emptying, and decreasing appetite. Their main advantage is weight loss, which can be significant in some of the patients. Limiting side effects of these agents are nausea and vomiting, particularly early in the course of treatment [40].
- Albiglutide is non-inferior to IGlar at reducing A1C, with modest weight loss and less hypoglycemia [41]. Similarly, dulaglutide in combination with ILis results in a significantly greater improvement in glycemic control than IGlar [42]. Furthermore, a systematic review and meta-analysis of RCTs report that with dulaglutide, exenatide (once weekly), and liraglutide, the absolute reduction in A1C at 6 months was 0.9–1.4% and was significantly better than exenatide twice daily and conclude that once-weekly GLP-1 analogues are a convenient therapeutic option for use as add-on to metformin [43]. There have been concerns regarding an increased risk of pancreatitis with GLP-1 analogues but recently published ELIXA and LEADER studies do not show any increased risk of pancreatitis, pancreatic cancer, or thyroid cancer with lixisenatide or liraglutide [44, 45].
- Furthermore, in the LEADER trial, the primary composite outcome of first occurrence of death from CV causing non-fatal MI or non-fatal stroke was significantly less with liraglutide as compared to placebo (HR, 0.87; 95% CI, 0.78, 0.97; *p* < 0.001 for non-inferiority; *p* = 0.01 for superiority) [45]. On the basis of this result, liraglutide is approved for its CV benefits as well by FDA “as an adjunct to standard treatment of CV risk factors to reduce the risk of major adverse CV events (CV death, non-fatal MI, or non-fatal stroke) in adults with T2DM and high CV risk” [2].

#### **Combination injectable therapy (insulin + GLP-1 analogues) [1]**

- Consider advancing to combination injectable therapy if the basal insulin has been titrated to acceptable FPG level or if the dose is 0.5 U/kg/day and A1C remains above the target.
- Metformin can be continued and other antidiabetic agents can be discontinued on individual basis when combination therapy is initiated. Generally, GLP-1 analogues are not discontinued after initiating basal insulin; however, when more complex insulin regimens are started, they should be stopped. Similarly, sulfonylureas and DPP4 inhibitors should also be stopped or their dose reduced.
- Basal insulin plus GLP-1 analogues are non-inferior to basal plus insulin regimens. This combination is also associated with less hypoglycemia risk and promotes weight loss instead of weight gain. However, it may be associated with transient gastrointestinal side effects.

#### **Implementation**

Timely initiation and appropriate intensification of injectable therapy is a challenging aspect of diabetes care. This must be addressed by focusing on patient education and motivation, as well as updating knowledge of healthcare professionals. Lifestyle modification, self-monitoring, and insulin education should be integral parts of insulin therapy in T2DM. Structured guidelines and protocols should be shared and glycemic audits of persons on oral medications performed to address the issue.

**References**

1. American Diabetes Association. Standards of medical care in diabetes. 2017. Available at:http://professional.diabetes.org/sites/professional.diabetes.org/files/media/dc_40_s1_final.pdf
2. FDA Advisory Committee briefing materials. Available at: https://www.fda.gov/downloads/AdvisoryCommittees/CommitteesMeetingMaterials/Drugs/EndocrinologicandMetabolicDrugsAdvisoryCommittee/UCM563335.pdf. Accessed on 17 Sep 2017.
3. International Diabetes Federation Guideline Development Group. Global guideline for type 2 diabetes. Diabetes Res Clin Pract. 2014;104(1):1–52.
4. Reznik Y, Cohen O. Insulin pump for type 2 diabetes. Diabetes Care. 2013;36(Supplement 2):S219–25.
5. Shah S, Sharma SK, Singh P, Muruganathan A, Das AK. Consensus evidence-based guidelines for insulin initiation, optimization and continuation in type 2 diabetes mellitus. J Assoc Doctors India. 2014;62(7 Suppl):49–54.
6. Holman RR, Paul SK, Bethel MA, Matthews DR, Neil HA. 10-year follow-up of intensive glucose control in type 2 diabetes. New England Journal of Medicine. 2008;359(15):1577–89.
7. Ghosh S, Unnikrishnan AG, Saboo B, Kesavadev J, Aravind SR, Bajaj S, Rajput R, Seshadri K, Verma N, Gupta A, Makkar BM. Evidence-based recommendations for insulin intensification strategies after basal insulin in type 2 diabetes. Diabetes & Metabolic Syndrome: Clinical Research & Reviews. 2017.
8. Handelsman Y, Bloomgarden ZT, Grunberger G, Umpierrez G, Zimmerman RS, Bailey TS, Blonde L, Bray GA, Cohen AJ, Dagogo-Jack S, Davidson JA. American Association of Clinical Endocrinologists and American College of Endocrinology—clinical practice guidelines for developing a diabetes mellitus comprehensive care plan—2015. Endocrine Practice. 2015;21(s1):1–87.
9. Baruah MP, Kalra S, Bose S, Deka J. An audit of insulin usage and insulin injection practices in a large Indian cohort. Indian Journal of Endocrinology and Metabolism. 2017;21(3):443.
10. UK Prospective Diabetes Study (UKPDS) Group. Intensive blood-glucose control with sulphonylureas or insulin compared with conventional treatment and risk of complications in patients with type 2 diabetes (UKPDS 33). Lancet. 1998;352(9131):837–53.
11. Lachin J, Genuth S, Nathan D, Davis M. The Diabetes Control and Complications Trial/Epidemiology of Diabetes Interventions and Complications Research Group. Retinopathy and nephropathy in patients with type 1 diabetes four years after a trial of intensive therapy. N Engl J Med 2000;342:381–9.
12. Raccah D. Basal insulin treatment intensification in patients with type 2 diabetes mellitus: a comprehensive systematic review of current options. Diabetes & Metabolism. 2017.
13. Kalra S, Baruah MP, Sahay RK, Unnikrishnan AG, Uppal S, Adetunji O. Glucagon-like peptide-1 receptor agonists in the treatment of type 2 diabetes: past, present, and future. Indian Journal of Endocrinology and Metabolism. 2016;20(2):254.
14. Kalra S, Kawatra P. Metabolic karma in diabetes care: medico-philosophical reflections. Indian Journal of Endocrinology and Metabolism. 2017;21(4):643.
15. Cryer PE. Hypoglycaemia: the limiting factor in the glycaemic management of type I and type II diabetes. Diabetologia 2002;45(7):937–48.
16. Inzucchi SE, Bergenstal RM, Buse JB, Diamant M, Ferrannini E, Nauck M, Peters AL, Tsapas A, Wender R, Matthews DR. Management of hyperglycaemia in type 2 diabetes: a patient-centered approach. Position statement of the American Diabetes Association (ADA) and the European Association for the Study of Diabetes (EASD). Diabetologia. 2012;55(6):1577–96.
17. Ismail-Beigi F, Moghissi E, Tiktin M, Hirsch IB, Inzucchi SE, Genuth S. Individualizing glycemic targets in type 2 diabetes mellitus: implications of recent clinical trials. Ann Intern Med. 2011;154(8):554–9.
18. Edridge CL, Dunkley AJ, Bodicoat DH, Rose TC, Gray LJ, Davies MJ, Khunti K. Prevalence and incidence of hypoglycaemia in 532,542 people with type 2 diabetes on oral therapies and insulin: a systematic review and meta-analysis of population based studies. PLOS One. 2015;10(6):e0126427.
19. Chandalia HB, Lamba PS, Chandalia SH, Singh DK, Modi SV, Shaikh SA. Weight gain in type 2 diabetics with different treatment modalities. Metab Syndr Relat Disord. 2005;3(2):130–6.
20. Vos RC, van Avendonk MJ, Jansen H, Goudswaard AN, van den Donk M, Gorter K, Kerssen A, Rutten GE. Insulin monotherapy compared with the addition of oral glucose‐lowering agents to insulin for people with type 2 diabetes already on insulin therapy and inadequate glycaemic control. The Cochrane Library. 2016.
21. Kalra S, Ghosal S, Shah P. Consensus on bridges for barriers to insulin therapy. The Journal of the Association of Physicians of India. 2017;65(3):23.
22. Holman RR, Farmer AJ, Davies MJ, Levy JC, Darbyshire JL, Keenan JF et al. Three-year efficacy of complex insulin regimens in type 2 diabetes. N Engl J Med 2009;361(18):1736–47.
23. Kalra S, Gupta Y. Insulin initiation: bringing objectivity to choice. Journal of Diabetes & Metabolic Disorders. 2015;14(1):17.
24. Inzucchi SE, Bergenstal RM, Buse JB, Diamant M, Ferrannini E, Nauck M, et al. Management of hyperglycemia in type 2 diabetes, 2015: a patient-centered approach: update to a position statement of the American Diabetes Association and the European Association for the Study of Diabetes. Diabetes Care. 2015;38(1):140–9.
25. Kovil R, Chawla M, Rajput R, Singh AK, Sinha B, Ghosal S, Ballani P, Gupta S, Tanna S, Bandukwala SM, Shah T. Consensus on insulin dose and titration algorithms in ambulatory care of type 2 diabetes in India. Journal of the Association of Physicians of India. 2017;65:17.
26. AK Das, BK Sahay, V Seshiah, V Mohan, A Muruganathan, Ajay Kumar et al. Indian National Consensus Group: national guidelines on initiation and intensification of insulin therapy with premixed insulin analogs. APII India, Diabetologia; Chapter 51, http://www.apiindia.org/medicine_update_2013/chap51.pdf . Accessed on September 2015.
27. Mohan V, Kalra S, Kesavadev J, Singh AK, Kumar A, Unnikrishnan AG, Chawla R, Mukherjee JJ, Sahay RK, Kumar10 JS, Bhoraskar11 A. Consensus on initiation and intensification of premix insulin in type 2 diabetes management. Journal of the Association of Physicians of India. 2017;65:59.
28. Hermansen K, Davies M, Derezinski T, Martinez Ravn G, Clauson P, Home P. A 26-week, randomized, parallel, treat-to-target trial comparing insulin detemir with NPH insulin as add-on therapy to oral glucose-lowering drugs in insulin-naive people with type 2 diabetes. Diabetes Care 2006;29(6):1269–74.
29. Riddle MC. The treat-to-target trial and related studies. Endocr Pract. 2006;12 Suppl 1:71–9.
30. Horvath K, Jeitler K, Berghold A, Ebrahim SH, Gratzer TW, Plank J, Kaiser T, Pieber TR, Siebenhofer A. Long‐acting insulin analogues versus NPH insulin (human isophane insulin) for type 2 diabetes mellitus. The Cochrane Library. 2007.
31. Owens DR, Traylor L, Mullins P, Landgraf W. Patient-level meta-analysis of efficacy and hypoglycaemia in people with type 2 diabetes initiating insulin glargine 100 U/mL or neutral protamine Hagedorn insulin analysed according to concomitant oral antidiabetes therapy. Diabetes Research and Clinical Practice. 2016.
32. Laubner K, Molz K, Kerner W, Karges W, Lang W, Dapp A, Schütt M, Best F, Seufert J, Holl RW. Daily insulin doses and injection frequencies of neutral protamine Hagedorn (NPH) insulin, insulin detemir and insulin glargine in type 1 and type 2 diabetes: a multicenter analysis of 51,964 patients from the German/Austrian DPV‐wiss database. Diabetes/Metabolism Research and Reviews. 2014;30(5):395–404.
33. Rosenstock J, Davies M, Home PD, Larsen J, Koenen C, Schernthaner G. A randomised, 52-week, treat-to-target trial comparing insulin detemir with insulin glargine when administered as add-on to glucose-lowering drugs in insulin-naïve people with type 2 diabetes. Diabetologia. 2008;51(3):408–16.
34. Ghosal S, Sinha B, Gangopadhyay KK. Insulin glargine versus insulin degludec in patients failing on oral therapy in type 2 diabetes: a retrospective real world comparative data from India. Diabetes & Metabolic Syndrome: Clinical Research & Reviews. 2016;10(3):161–5.
35. Unnikrishnan AG, Tibaldi J, Hadley-Brown M et al. Practical guidance on intensification of insulin therapy with BIAsp 30: a consensus statement. Int J Clin Pract. 2009;63:1571–7.
36. Sheu WH, Ji L, Lee WJ, Jabbar A, Han JH, Lew T. Efficacy and safety of premixed insulin analogs in Asian patients with type 2 diabetes: a systematic review. Journal of Diabetes Investigation. 2017.
37. Kesavadev J, Rajput R, John M, Annamalai AK, Rao PV. Consensus statement on choice of insulin therapy in type 2 diabetes. Journal of the Association of Physicians of India. 2016; Suppl: 13–18
38. Christiansen JS, Niskanen L, Rasmussen S, Johansen T, Fulcher G. Lower rates of hypoglycemia during maintenance treatment with insulin degludec/insulin aspart versus biphasic insulin aspart 30: a combined analysis of two Phase 3a studies in type 2 diabetes. Journal of Diabetes. 2016.
39. Kaneko S, Chow F, Choi DS, Taneda S, Hirao K, Park Y, Andersen TH, Gall MA, Christiansen JS. Insulin degludec/insulin aspart versus biphasic insulin aspart 30 in Asian patients with type 2 diabetes inadequately controlled on basal or pre-/self-mixed insulin: a 26-week, randomised, treat-to-target trial. Diabetes Research and Clinical Practice. 2015;107(1):139–47.
40. Kim W, Egan JM. The role of incretins in glucose homeostasis and diabetes treatment. Pharmacological Reviews. 2008;60(4):470–512.
41. Weissman PN, Carr MC, Ye J, Cirkel DT, Stewart M, Perry C, Pratley R. HARMONY 4: randomised clinical trial comparing once-weekly albiglutide and insulin glargine in patients with type 2 diabetes inadequately controlled with metformin with or without sulfonylurea. Diabetologia. 2014;57(12):2475–84.
42. Blonde L, Jendle J, Gross J, Woo V, Jiang H, Fahrbach JL, Milicevic Z. Once-weekly dulaglutide versus bedtime insulin glargine, both in combination with prandial insulin lispro, in patients with type 2 diabetes (AWARD-4): a randomised, open-label, phase 3, non-inferiority study. The Lancet. 2015;385(9982):2057–66.
43. Orme ME, Nguyen H, Lu JY, Thomas SA. Comparative effectiveness of glycemic control in patients with type 2 diabetes treated with GLP-1 receptor agonists: a network meta-analysis of placebo-controlled and active-comparator trials. Diabetes, Metabolic Syndrome and Obesity: Targets and Therapy. 2017;10:111.
44. Bentley-Lewis R, Aguilar D, Riddle MC, Claggett B, Diaz R, Dickstein K, et al. Rationale, design, and baseline characteristics in evaluation of LIXisenatide in acute coronary syndrome, a long-term cardiovascular end point trial of lixisenatide versus placebo. Am Heart J. 2015;169(5):631–8.e7.
45. Marso SP, Daniels GH, Brown-Frandsen K, Kristensen P, Mann JF, Nauck MA, Nissen SE, Pocock S, Poulter NR, Ravn LS, Steinberg WM. Liraglutide and cardiovascular outcomes in type 2 diabetes. New England Journal of Medicine. 2016;375(4):311–22.

## **Alternate therapies**

### **RSSDI 2017 recommendations**

#### **Recommended care**

- Clinicians trained in modern system of medicine are advised not to prescribe alternate therapies to treat T2DM. However, some of the alternate therapies such as plant-based preparations and yoga may be used along with pharmacological therapy in certain patients with T2DM to reduce complications. However, these therapies should not replace conventional diabetes therapies.

As diabetes is a disease which is chronic and controllable, rather than curable, patients are often daunted by the possibility of lifelong allopathic medications. Therefore, some patients use complementary and alternative medicinal (CAM) therapies for its prevention and management [1]. Ayurveda, yoga, unani, siddha, homeopathy, and naturopathy systems of medicine are often integrated into diabetes healthcare delivery. More than 300 Indian plant and mineral products have been reported, with sub-optimal therapeutic effects in diabetes. Evidence has established a few beneficial effects of methi, vijaysar, gurmar, neem, amla, ghritkumari, turmeric, black pepper, date fruit, onion, and karela in diabetes [1–17]. Moreover, yoga, pranayama, meditation, acupuncture, massage therapy, aromatherapy, and many relaxation techniques are being practiced in India, for prevention and management of diabetes [17–19]. Furthermore, some dietary supplements including chromium, alpha-lipolic acid, omega-3 fatty acids, magnesium, and zinc are also having some beneficial effects in the management of diabetes [1]. Although they do not substitute physical activity, they may supplement non-pharmacologic therapies in diabetes. Many of these alternative therapies have not been subjected to or have not withstood the rigorous scientific studies such as RCTs. Hence, the use of these agents may be considered as an adjunct to standard care. Moreover, there is a need to generate evidence on the benefits as well as toxicity of these agents by rigorously conducted clinical trials.

**References**

1. Kesavadev J, Saboo B, Sadikot S, Das AK, Joshi S, Chawla R, Thacker H, Shankar A, Ramachandran L, Kalra S. Unproven therapies for diabetes and their implications. Advances in Therapy. 2017:1–8.
2. Upasani SV, Ingle PV, Patil PH, Nandedkar RY, Shah VS, Surana SJ. Traditional Indian spices useful in diabetes mellitus—an updated review. J. Pharm. BioSci. 2013;4:157–161.
3. Modak M, Dixit P, Londhe J, Ghaskadbi S, Devasagayam TP. Indian herbs and herbal drugs used for the treatment of diabetes. J Clin Biochem Nutr. 2007;40(3):163–73.
4. Sharma R. Efficacy of the bitter principles on post-glucose blood glucose values. 1983;3:39–42. Available at: http://diabetes.org.in/journal/1983_dec/original_paper4.pdf (Last accessed on 27 Aug 2015).
5. Sharma RD, Raghuram TC, Rao NS. Effect of fenugreek seeds on blood glucose and serum lipids in type I diabetes. Eur J Clin Nutr. 1990;44(4):301–6.
6. Raghuram TC, Sharma RD, Pasricha S, Menon KK, Radhaiah G. Glycemic index of fenugreek recipes and its relation to dietary fibre. Intl J Diab Dev Countries. 1992;12:1–4. Available at: http://ksiconnect.icrisat.org/wp-content/uploads/2014/05/p-v-Rao.pdf (Last accessed on 27 Aug 2015)
7. Flexible dose open trial of Vijayasar in cases of newly-diagnosed non-insulin-dependent diabetes mellitus. Indian Council of Medical Research (ICMR), Collaborating Centres, New Delhi. Indian J Med Res. 1998;108:24–9.
8. Fuangchan A, Sonthisombat P, Seubnukarn T, Chanouan R, Chotchaisuwat P, Sirigulsatien V, Ingkaninan K, Plianbangchang P, Haines ST. Hypoglycemic effect of bitter melon compared with metformin in newly diagnosed type 2 diabetes patients. Journal of Ethnopharmacology. 2011;134(2):422–8.
9. Joseph B, Jini D. Antidiabetic effects of *Momordica charantia* (bitter melon) and its medicinal potency. Asian Pacific Journal of Tropical Disease. 2013;3(2):93–102.
10. Salam MA, El-Gengaihi SE, Zikry EN. Preliminary clinical trials of karela, *Momordica charantia*, on non-insulin-dependent diabetes mellitus patients. Egyptian Pharmaceutical Journal. 2015;14(1):69.
11. Gaddam A, Galla C, Thummisetti S, Marikanty RK, Palanisamy UD, Rao PV. Role of Fenugreek in the prevention of type 2 diabetes mellitus in prediabetes. Journal of Diabetes & Metabolic Disorders. 2015;14(1):74.
12. Hariharan RS, Venkataraman S, Sunitha P, Rajalakshmi S, Samal KC, Routray BM, Jayakumar RV, Baiju K, Satyavati GV, Muthuswamy V, Gupta AK. Efficacy of vijayasar (*Pterocarpus marsupium*) in the treatment of newly diagnosed patients with type 2 diabetes mellitus: a flexible dose double-blind multicenter randomized controlled trial. Diabetologia Croatica. 2005;34(1):13–20.
13. Khaliq T, Sarfraz M, Ashraf MA. Recent progress for the utilization of *Curcuma longa*, *Piper nigrum* and *Phoenix dactylifera* seeds against type 2 diabetes. The West Indian Medical Journal. 2015;64(5):527.
14. Akash MS, Rehman K, Chen S. Spice plant *Allium cepa*: dietary supplement for treatment of type 2 diabetes mellitus. Nutrition. 2014;30(10):1128–37.
15. Walia K, Boolchandani R. Role of amla in type 2 diabetes mellitus—a review. Research Journal of Recent Sciences. 2015;4:31–5.
16. Tiwari P, Ahmad K, Hassan Baig M. *Gymnema sylvestre* for diabetes: from traditional herb to future’s therapeutic. Current Pharmaceutical Design. 2017;23(11):1667–76.
17. Pandey A, Tripathi P, Pandey R, Srivatava R, Goswami S. Alternative therapies useful in the management of diabetes: a systematic review. Journal of Pharmacy & Bioallied Sciences. 2011;3(4):504.
18. Chimkode SM, Kumaran SD, Kanhere VV, Shivanna R. Effect of yoga on blood glucose levels in patients with type 2 diabetes mellitus. J Clin Diagn Res. 2015;9(4):CC01–3.
19. Nagarathna R, Usharani M R, Rao A R, Chaku R, Kulkarni R, NagendraH R. Efficacy of yoga based life style modification program on medication score and lipid profile in type 2 diabetes—a randomized control study. Int J Diabetes Dev Ctries. 2012;32(3):122–130.

## **Individualizing therapy**

### **RSSDI 2017 recommendations**

#### *ABCD (EFGH) approach for diabetes management*

Choice of any antidiabetic agent should take into account the patient’s general health status and associated medical disorders. This patient-centric approach may be referred to as the ABCD (EFGH) approach for diabetes management. As shown in the figure, for any T2DM patients, first line of therapy should be metformin unless it is not tolerated by the patient or contraindicated.

### **Individualized treatment**

- For patients who have been diagnosed with diabetes, consider a combination of metformin and one of the treatment options based on patients **A**ge, **B**MI, **CKD**, **D**uration of diabetes**, E**stablished CVD, **F**inancial condition, **Glyce**mic status, and **H**ypoglycemia concern.
- Drug choice should be based on patient preferences as well as presence of various comorbidities and complications and drug characteristics, with the goal of reducing blood glucose levels while minimizing side effects, especially hypoglycemia and weight gain.
- A comparative effectiveness meta-analysis suggests that overall each new class of non-insulin agents added to initial therapy lowers A1C around 0.9–1.1% [1]. Moreover, current National Insulin Summit (NIS) consensus from India reports that all oral antidiabetic agents can reduce A1C to a range of 0.5–2.0% and injectables (GLP-1 RA and insulin) reduce A1C to a range of 0.5–3.5% when used as monotherapy [2].

## RSSDI therapeutic wheel


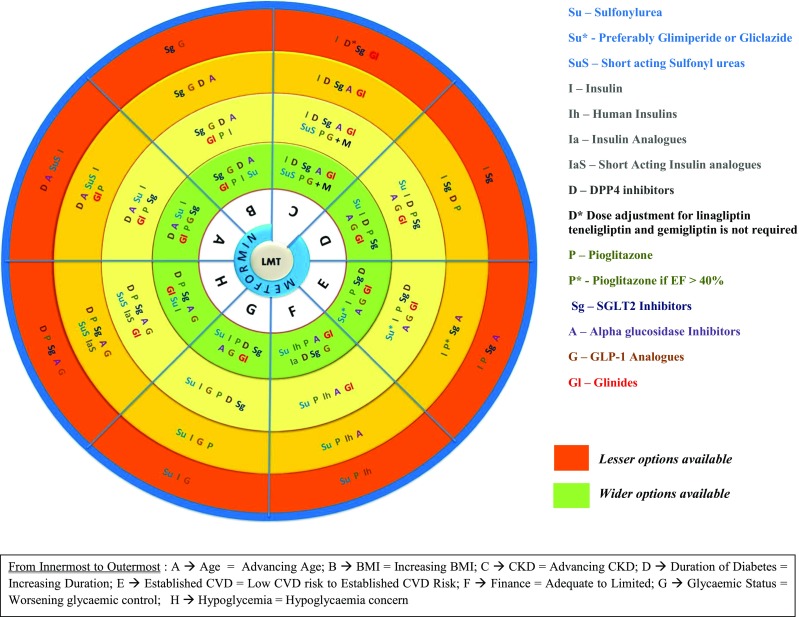


Note: Hydroxychloroquinone has recently been approved by DCGI for the treatment of T2DM as third-line therapy; SGLT2 inhibitors are recommended in patients with high CV risk

### **Age**

- eGFR-adjusted doses of gliptins may be a suitable addition to metformin for elderly patients to avoid hypoglycemia and weight gain [3]. Recent double blind RCTs have reported that gliptins are efficacious and safe with no tolerability issue when used as add-on therapy in elderly patients with T2DM [4–7].
- Agents belonging to AGIs could also be an important choice in elderly patients. These agents have moderate efficacy and minimal side effects like hypoglycemia, but the major limiting factor for their use is the gastrointestinal side effects, such as flatulence and diarrhea [8]. A double blind RCT revealed that compared to diet alone, addition of acarbose improved the glycemic profile and insulin sensitivity in elderly patients with T2DM [9].
- Glitazones are a safer alternative in patients with preserved cardiac function. However, postmenopausal females must be spared for their use because of high predisposition to osteoporosis. Furthermore, the use of glitazones is restricted in elderly T2DM patients owing to the complications like weight gain, fluid retention, peripheral edema, aggravation of congestive heart failure, and especially increased risk of bladder cancer associated with their use [10].
- Newer sulfonylureas like gliclazide MR and glimepiride (due to low risk of hypoglycemia) and glinides (due to shorter half-life) can be safely used in elderly patients with T2DM [11]. A recent RCT (GENERATION) did not find any significant difference between saxagliptin and glimepiride in elderly T2DM patients [12].
- Evidence regarding the use of GLP-1 RA and SGLT2 inhibitors in elder T2DM patients is limited. However, available data suggests that agents of both the classes provide good glycemic control in patients with T2DM. However, certain drawbacks like cost, injection, and limited availability with GLP-1RAs and increased risk of genital and urinary tract infections, hypovolemia, postural hypotension, and weight loss with SGLT2 inhibitors may limit their usage in some older T2DM patients [10].
- Early initiation of insulin in older patients was found to be beneficial without increasing risk of hypoglycemia or greater total direct healthcare costs [13]. Evidence suggests that basal insulin analogues like glargine and detemir were effective and safe without any risk of hypoglycemia and weight gain [14, 15]. Moreover, a pooled analysis from five RCTs revealed that addition of insulin glargine compared to NPH insulin to oral antidiabetic drugs in older adults was effective with low risk of hypoglycemia [16].
- In addition, individualization of therapy is desirable based on risk of hypoglycemia, comorbidities, functionality, cost, and personal preference.

### **BMI**

- While prescribing pharmacological treatments for overweight or obese patients with T2DM, providers should first consider antidiabetes medications which cause either weight neutrality or weight loss. Metformin, AGIs, GLP-1 RAs, and SGLT-2 inhibitors are associated with weight loss characteristics, and DPP-4 inhibitors appear to be weight neutral [17, 18]. A systematic review and meta-analysis of 62 randomized trials revealed that, when compared to other antidiabetic agents, SGLT2 inhibitors and GLP-1 RAs were associated with clinically significant body weight loss (range, 1.15–2.26 kg) as add-on to metformin [19].
- GLP-1 RA seems to be the best add-on therapy for those having high BMI. This group of medications has the highest weight-reducing property in addition to excellent efficacy. A recent systematic review and mixed treatment comparison meta-analysis report that GLP-1 RAs are associated with weight loss (− 1.62 to − 1.01 kg) in overweight or obese patients with T2DM with no difference in weight loss between different types of GLP-1 RAs [20].
- SGLT-2 inhibitors also have a weight reduction property. Evidence suggests that SGLT-2 inhibitors were associated with weight loss in patients with T2DM [21, 22]. Medicines in this group have an additional advantage of excellent tolerance and can be given orally. However, their glycemic efficacy seems to be less than that of GLP-1 RA and also experience with this group of agents is less than that with GLP-1 RA [23].
- Gliptins are weight neutral and so can be used as third line of agents [24, 25].
- Use of newer sulfonylureas compared to older sulfonylureas and other OADs does not result in significant weight gain in patients with T2DM [26–28]. Last option for such kind of patients should be insulin or glitazones since they are having weight gain properties.

### **Complications (CKD)**

- In the same manner, if we focus on complications (renal impairment), preference of therapy would be gliptins as add-on therapy with metformin [29]. Few of the gliptins need dose adjustment as per eGFR while vildagliptin needs dose adjustment in hepatic insufficiency. Linagliptin and teneligliptin do not require any dose adjustment in renal disease [30–33].
- Repaglinide is another agent which may be used across all stages of renal insufficiency. Similarly, glitazones may be used in CKD; however, one has to be careful about fluid retention [34, 35].
- Short-acting sulfonylureas like glipizide can be preferred in patients with moderate/severe renal impairment. Furthermore, in mild/moderate renal impairment, gliclazide and glimepiride may also be used, preferably at lower doses [11].
- GLP-1 RAs, owing to their GI adverse effect, limit their use in renal insufficiency patients [34].
- AGIs may be used in patients with mild to moderate renal disease [35].
- Insulin may be used in any stages of renal insufficiency and is the best agent for this purpose. Short-acting insulin analogues are preferred over conventional insulins [36] and insulin doses should be reduced with falling eGFR and A1C targets can be increased slightly [37, 38].

### **Duration of diabetes**

- Patients with long-standing T2DM are very challenging to treat because these patients often lack sufficient β-cell function to respond to some oral glucose-lowering agents, may have profound comorbidities, and may have renal impairment [39]. As results of recent trials have suggested to utilize an aggressive approach in cases where duration of diabetes <5 years, SU or glinide, as an add-on therapy to metformin, will be the best choices, as they are very potent agents [40]. Addition of glitazones may also be useful at this stage [41].
- Apart from this, ADA 2013 stated that lean patients with long duration of disease may benefit from gliptins or sulfonylureas with early use of insulin [42].
- Basal insulin analogues are often used in patients with long-standing diabetes to address insulinopenic states [39].
- Incretin-based therapies, particularly GLP-1 receptor agonists, provide postprandial control with lower risks of hypoglycemia than prandial insulin [39]. GLP-1 RA may score over gliptins for this indication as they are more effective than gliptins. Therefore, gliptins may be considered as second add-on option.
- SGLT-2 inhibitors may also be useful as second add-on agent due to their insulin-independent action which is pathophysiologically different [43].
- AGIs are last choices due to their moderate efficacy.

### **Established CVD**

- Intensive glycemic control with antidiabetic drugs reduces cardiovascular risk and complications in patients with T2DM [44].
- In patients with established CVD, DPP4 inhibitors, GLP-1 analogues, and SGLT-2 inhibitors may be preferred [45].
- Pioglitazone should not be used in heart failure [45] or patients with low ejection fraction [46]. Moreover, pioglitazone has been shown in different studies to reduce CVD risk [47, 48].
- Glimepiride and gliclazide MR can be preferred over conventional sulfonylureas in patients at increased risk of CVD or with CVD [11].
- GLP-1 RAs may be suitable alternative for patients who are overweight or obese. AGIs may be preferred in patients with postprandial hyperglycemia.
- Recent data from EMPA-REG and CANVAS studies have shown that SGLT-2 inhibitors reduce CV risk and CV mortality and may be preferred [49, 50].

### **Financial concern**

- Considering that many Indian patients do not have medical insurance and treatment needs to be continued lifelong, cost of therapy also plays an essential role in T2DM patients from Indian subcontinent.
- SUs should be the first choice with metformin by considering their cost. Then after AGIs or glitazones should be used at next therapy level [51]. In the next level, the therapeutic option should be glinides or insulin.
- High cost will prevent the use of insulin analogues, gliptins, SGLT-2 inhibitors, and GLP-1 RA in most of the patients [52].

### **Glycemic status**

- Good glycemic control of patients is directly correlated with efficacy of any antidiabetic agent.
- The order of glucose-lowering agents according to their efficacy of A1C reduction is insulin, sulfonylureas, GLP-1 RAs, pioglitazone, gliptins, SGLT-2 inhibitors, and AGIs [2, 53].
- Insulin followed by GLP-1RA, SUs, and glitazones have highest efficacy in terms of reducing A1C [54].
- As a second-line agent, insulin should be preferred, followed by GLP-1 RA, sulfonylureas, gliptins, and others [55].
- Gliptins, SGLT-2 inhibitors, or AGIs should be considered as add-on therapy if these agents are not able to achieve glycemic targets.
- It is always to be understood that good efficacy, in most cases, comes with a price written on it in the form of increased incidence of hypoglycemia or prohibitive cost.

### **Hypoglycemia concern**

- Hypoglycemia is the biggest hurdle that any medical fraternity is facing during treatment course of diabetes.
- Sulfonylureas have an increased risk of severe hypoglycemia compared with metformin or thiazolidinedione monotherapy. Moreover, sulfonylureas as a second-line agent have a greater risk of severe hypoglycemia than DPP-4 inhibitors and SGLT-2 inhibitors [53].
- A traditional meta-analysis reported that only sulfonylureas (relative risk (RR), 4.57) and glinides (RR, 7.50) were associated with increased risk of hypoglycemia, whereas thiazolidinediones (RR, 0.56), AGIs (RR, 0.42), DPP-4 inhibitors (RR, 0.63), and GLP-1 RAs (RR, 0.89) were not associated [56].
- On introducing DPP-4i on a background of secretagogues, the dose of secretagogues needs to be reduced and close monitoring of blood glucose is necessary [57]. Similarly, while introducing SGLT-2i on a background of insulin or secretagogues, the dose of insulin or secretagogues needs to be reduced [58].
- In patients with history of hypoglycemia or for those at high risk of hypoglycemia, GLP-1 RA or gliptins should be considered as first choice with metformin [59]. Other options include SGLT-2 inhibitors, glitazones, and AGIs.
- Last option for such patients should be glinides, sulfonylureas, or insulin since there are high chances of hypoglycemia with these agents.
- Patients requiring to avoid hypoglycemia include:
  - Those with established CV disease
  - Elderly patients
  - Those suffering from retinopathy and cannot perform SMBG without help of others
  - Those who stay alone, especially in remote areas
  - Those who are having poor longevity
  - Those who are having documented hypoglycemia unawareness
  - Those who met with severe symptomatic hypoglycemia requiring hospitalization

**References**

1. Bennett WL, Maruthur NM, Singh S, Segal JB, Wilson LM, Chatterjee R et al. Comparative effectiveness and safety of medications for type 2 diabetes: an update including new drugs and 2-drug combinations. Ann Intern Med 2011;154(9):602–13.
2. Jothydev Kesavadev RR, John M, Annamalai AK, Rao PV. Consensus statement on choice of insulin therapy in type 2 diabetes. JAPI, 2016: 13–18
3. Type 2 diabetes—newer agents (partial update); NICE Clinical Guideline. 2009 Available at: patient.info/doctor/antihyperglycaemic-agents-used-for-type-2-diabetes (Last accessed on 27 Aug 2015).
4. Barnett AH, Huisman H, Jones R, von Eynatten M, Patel S, Woerle HJ. Linagliptin for patients aged 70 years or older with type 2 diabetes inadequately controlled with common antidiabetes treatments: a randomised, double-blind, placebo-controlled trial. The Lancet. 2013;382(9902):1413–23.
5. Strain WD, Lukashevich V, Kothny W, Hoellinger MJ, Paldánius PM. Individualised treatment targets for elderly patients with type 2 diabetes using vildagliptin add-on or lone therapy (INTERVAL): a 24 week, randomised, double-blind, placebo-controlled study. The Lancet. 2013;382(9890):409–16.
6. Chien MN, Lee CC, Chen WC, Liu SC, Leung CH, Wang CH. Effect of sitagliptin as add-on therapy in elderly type 2 diabetes patients with inadequate glycemic control in Taiwan. International Journal of Gerontology. 2011;5(2):103–6.
7. Strain WD, Agarwal AS, Paldánius PM. Individualizing treatment targets for elderly patients with type 2 diabetes: factors influencing clinical decision making in the 24-week, randomized INTERVAL study. Aging (Albany NY). 2017;9(3):769.
8. Dardano A, Penno G, Del Prato S, Miccoli R. Optimal therapy of type 2 diabetes: a controversial challenge. Aging (Albany NY). 2014;6(3):187.
9. Josse RG, Chiasson JL, Ryan EA, Lau DC, Ross SA, Yale JF, Leiter LA, Maheux P, Tessier D, Wolever TM, Gerstein H. Acarbose in the treatment of elderly patients with type 2 diabetes. Diabetes Research and Clinical Practice. 2003;59(1):37–42.
10. Dunning T, Sinclair A, Colagiuri S. New IDF guideline for managing type 2 diabetes in older people. Diabetes Research and Clinical Practice. 2014;103(3):538–40.
11. Kalra S, Aamir AH, Raza A, Das AK, Khan AA, Shrestha D, Qureshi MF, Fariduddin M, Pathan MF, Jawad F, Bhattarai J. Place of sulfonylureas in the management of type 2 diabetes mellitus in South Asia: a consensus statement. Indian Journal of Endocrinology and Metabolism. 2015;19(5):577.
12. Schernthaner G, Durán-Garcia S, Hanefeld M, Langslet G, Niskanen L, Östgren CJ, Malvolti E, Hardy E. Efficacy and tolerability of saxagliptin compared with glimepiride in elderly patients with type 2 diabetes: a randomized, controlled study (GENERATION). Diabetes, Obesity and Metabolism. 2015;17(7):630–8.
13. Bhattacharya R, Zhou S, Wei W, Ajmera M, Sambamoorthi U. A real-world study of the effect of timing of insulin initiation on outcomes in older medicare beneficiaries with type 2 diabetes mellitus. Journal of the American Geriatrics Society. 2015;63(5):893–901.
14. Karnieli E, Baeres FM, Dzida G, Ji Q, Ligthelm R, Ross S, Svendsen AL, Yale JF. Observational study of once-daily insulin detemir in people with type 2 diabetes aged 75 years or older. Drugs & Aging. 2013;30(3):167–75.
15. Pandya N, DiGenio A, Gao L, Patel M. Efficacy and safety of insulin glargine compared to other interventions in younger and older adults: a pooled analysis of nine open-label, randomized controlled trials in patients with type 2 diabetes. Drugs & Aging. 2013;30(6):429–38.
16. Lee P, Chang A, Blaum C, Vlajnic A, Gao L, Halter J. Comparison of safety and efficacy of insulin glargine and neutral protamine hagedorn insulin in older adults with type 2 diabetes mellitus: results from a pooled analysis. Journal of the American Geriatrics Society. 2012;60(1):51–9.
17. American Diabetes Association. 6. Obesity management for the treatment of type 2 diabetes. Diabetes Care. 2016;39(Supplement 1):S47–51.
18. Svacina S. Treatment of obese diabetics. Advances in Experimental Medicine and Biology. 2011;771:459–64.
19. Mearns ES, Sobieraj DM, White CM, Saulsberry WJ, Kohn CG, Doleh Y, Zaccaro E, Coleman CI. Comparative efficacy and safety of antidiabetic drug regimens added to metformin monotherapy in patients with type 2 diabetes: a network meta-analysis. PLOS One. 2015;10(4):e0125879.
20. Potts JE, Gray LJ, Brady EM, Khunti K, Davies MJ, Bodicoat DH. The effect of glucagon-like peptide 1 receptor agonists on weight loss in type 2 diabetes: a systematic review and mixed treatment comparison meta-analysis. PLOS One. 2015;10(6):e0126769.
21. Monami M, Nardini C, Mannucci E. Efficacy and safety of sodium glucose co-transport-2 inhibitors in type 2 diabetes: a meta-analysis of randomized clinical trials. Diabetes, Obesity and Metabolism. 2014;16(5):457–66.
22. Tosaki T, Kamiya H, Himeno T, Kato Y, Kondo M, Toyota K, Nishida T, Shiroma M, Tsubonaka K, Asai H, Moribe M. Sodium-glucose co-transporter 2 inhibitors reduce the abdominal visceral fat area and may influence the renal function in patients with type 2 diabetes. Internal Medicine. 2017;56(6):597–604.
23. Cauthon K, Yendapally R. A review of GLP-1 receptor agonists and SGLT2 inhibitors for type 2 diabetes. Available at: http://c.ymcdn.com/sites/www.texaspharmacy.org/resource/resmgr/Conference/Review_for_Type_2_Diabetes_7.pdf (Last accessed on 27 Aug 2015)
24. Kodera R, Shikata K, Nakamura A, Okazaki S, Nagase R, Nakatou T, Haisa S, Hida K, Miyashita K, Makino H. The glucose-lowering efficacy of sitagliptin in obese Japanese patients with type 2 diabetes. Internal Medicine. 2017;56(6):605–13.
25. Chen JF, Chang CM, Kuo MC, Tung SC, Tsao CF, Tsai CJ. Impact of baseline body mass index status on glucose lowering and weight change during sitagliptin treatment for type 2 diabetics. Diabetes Research and Clinical Practice. 2016;120:8–14.
26. Zoungas S, Chalmers J, Neal B, Billot L, Li Q, Hirakawa Y, Arima H, Monaghan H, Joshi R, Colagiuri S, Cooper ME. Follow-up of blood-pressure lowering and glucose control in type 2 diabetes. New England Journal of Medicine. 2014;371(15):1392–406.
27. Martin S, Kolb H, Beuth J, Van Leendert R, Schneider B, Scherbaum WA. Change in patients’ body weight after 12 months of treatment with glimepiride or glibenclamide in type 2 diabetes: a multicentre retrospective cohort study. Diabetologia. 2003;46(12):1611–7.
28. Hassanein M, Abdallah K, Schweizer A. A double-blind, randomized trial, including frequent patient-physician contacts and Ramadan-focused advice, assessing vildagliptin and gliclazide in patients with type 2 diabetes fasting during Ramadan: the STEADFAST study. Vasc Health Risk Manag. 2014;10:319–26.
29. BMJ Blogs: Gliptins—where are we now? Available at: http://blogs.bmj.com/diabetes/2013/01/30/gliptins-where-are-we-as-of-now/ (Last accessed on 27 Aug 2015)
30. Russo E, Penno G, Del Prato S. Managing diabetic patients with moderate or severe renal impairment using DPP-4 inhibitors: focus on vildagliptin. Diabetes Metab Syndr Obes. 2013;6:161–70.
31. Penno G, Garofolo M, Del Prato S. Dipeptidyl peptidase-4 inhibition in chronic kidney disease and potential for protection against diabetes-related renal injury. Nutrition, Metabolism and Cardiovascular Diseases. 2016;26(5):361–73.
32. Kishimoto M. Teneligliptin: a DPP-4 inhibitor for the treatment of type 2 diabetes. Diabetes, Metabolic Syndrome and Obesity: Targets and Therapy. 2013;6:187.
33. Abubaker M, Mishra P, Swami OC. Teneligliptin in management of diabetic kidney disease: a review of place in therapy. Journal of Clinical and Diagnostic Research: JCDR. 2017;11(1):OE05.
34. Ioannidis I. Diabetes treatment in patients with renal disease: is the landscape clear enough? World Journal of Diabetes. 2014;5(5):651.
35. Madhu SV. Use of oral anti-diabetic agents in diabetes with chronic kidney disease. Medicine. 2011:157.
36. Urata H, Mori K, Emoto M, Yamazaki Y, Motoyama K, Morioka T, Fukumoto S, Koyama H, Shoji T, Ishimura E, Inaba M. Advantage of insulin glulisine over regular insulin in patients with type 2 diabetes and severe renal insufficiency. Journal of Renal Nutrition. 2015;25(2):129–34.
37. Abe M, Kalantar-Zadeh K. Haemodialysis-induced hypoglycaemia and glycaemic disarrays. Nature Reviews Nephrology. 2015;11(5):302–13.
38. Foundation NK. KDOQI clinical practice guideline for diabetes and CKD: 2012 update. American Journal of Kidney Diseases. 2012;60(5):850–86.
39. Moghissi ES. Treating patients with diabetes of long duration: GLP-1 receptor agonists and insulin in combination. The Journal of the American Osteopathic Association. 2014;114(5_suppl_2):S22–9.
40. Handelsman Y, Bloomgarden ZT, Grunberger G, Umpierrez G, Zimmerman RS, Bailey TS, Blonde L, Bray GA, Cohen AJ, Dagogo-Jack S, Davidson JA. American Association of Clinical Endocrinologists and American College of Endocrinology—clinical practice guidelines for developing a diabetes mellitus comprehensive care plan—2015. Endocrine Practice. 2015;21(s1):1–87.
41. Hanefeld M. Pioglitazone and sulfonylureas: effectively treating type 2 diabetes. Int J Clin Pract Suppl. 2007;(153):20–7.
42. Raz I, Riddle MC, Rosenstock J, Buse JB, Inzucchi SE, Home PD, Del Prato S, Ferrannini E, Chan JC, Leiter LA, LeRoith D. Personalized management of hyperglycemia in type 2 diabetes.
43. Kalra S. Sodium glucose co-transporter-2 (SGLT2) inhibitors: a review of their basic and clinical pharmacology. Diabetes Therapy. 2014;5(2):355–66.
44. Ray KK, Seshasai SR, Wijesuriya S, Sivakumaran R, Nethercott S, Preiss D, Erqou S, Sattar N. Effect of intensive control of glucose on cardiovascular outcomes and death in patients with diabetes mellitus: a meta-analysis of randomised controlled trials. The Lancet. 2009;373(9677):1765–72.
45. Xu J, Rajaratnam R. Cardiovascular safety of non-insulin pharmacotherapy for type 2 diabetes. Cardiovascular Diabetology. 2017;16(1):18.
46. Giles TD, Miller AB, Elkayam U, Bhattacharya M, Perez A. Pioglitazone and heart failure: results from a controlled study in patients with type 2 diabetes mellitus and systolic dysfunction. Journal of Cardiac Failure. 2008;14(6):445–52.
47. Dormandy JA, Charbonnel B, Eckland DJ, Erdmann E, Massi-Benedetti M, Moules IK, Skene AM, Tan MH, Lefèbvre PJ, Murray GD, Standl E. Secondary prevention of macrovascular events in patients with type 2 diabetes in the PROactive Study (PROspective pioglitAzone Clinical Trial In macroVascular Events): a randomised controlled trial. The Lancet. 2005;366(9493):1279–89.
48. Kernan WN, Viscoli CM, Furie KL, Young LH, Inzucchi SE, Gorman M, Guarino PD, Lovejoy AM, Peduzzi PN, Conwit R, Brass LM. Pioglitazone after ischemic stroke or transient ischemic attack. N Engl J Med. 2016;2016(374):1321–31.
49. Zinman B, Wanner C, Lachin JM, Fitchett D, Bluhmki E, Hantel S, Mattheus M, Devins T, Johansen OE, Woerle HJ, Broedl UC. Empagliflozin, cardiovascular outcomes, and mortality in type 2 diabetes. New England Journal of Medicine. 2015; 373(22):2117–28.
50. Neal B, Perkovic V, Mahaffey KW, de Zeeuw D, Fulcher G, Erondu N, Shaw W, Law G, Desai M, Matthews DR. Canagliflozin and cardiovascular and renal events in type 2 diabetes. New England Journal of Medicine. 2017.
51. Kalra S, Chadha M, Sharma SK, Unnikrishnan AG. Untapped diamonds for untamed diabetes: the α-glucosidase inhibitors. Indian J Endocrinol Metab. 2014;18(2):138–41.
52. Second- and third-line pharmacotherapy for type 2 diabetes: update. Ottawa (ON): Canadian Agency for Drugs and Technologies in Health; 2013. Available at: http://www.ncbi.nlm.nih.gov/books/NBK169628/ (Last accessed on 27 Aug 2015).
53. Bolen S, Tseng E, Hutfless S, Segal JB, Suarez-Cuervo C, Berger Z, et al. Diabetes medications for adults with type 2 diabetes: an update. 2016. Available at http://www.ncbi.nlm.nih.gov/pubmedhealth/PMH0088154/pdf/PubMedHealth_PMH0088154.pdf.
54. Gallwitz B. The future of combination therapies of insulin with a glucagon-like peptide-1 receptor agonists in type 2 diabetes—is it advantageous? European Endocrinology, 2014;10(2):98–9. Available at: http://www.touchendocrinology.com/articles/future-combination-therapies-insulin-glucagon-peptide-1-receptor-agonists-type-2-diabetes. (Last accessed on 27 Aug 2015)
55. Thomsen RW, Baggesen LM, Søgaard M, Pedersen L, Nørrelund H, Buhl ES, Haase CL, Johnsen SP. Early glycaemic control in metformin users receiving their first add-on therapy: a population-based study of 4734 people with type 2 diabetes. Diabetologia. 2015;58(10):2247–53.
56. Phung OJ, Scholle JM, Talwar M, Coleman CI. Effect of noninsulin antidiabetic drugs added to metformin therapy on glycemic control, weight gain, and hypoglycemia in type 2 diabetes. JAMA. 2010;303(14):1410–8.
57. Salvo F, Moore N, Arnaud M, Robinson P, Raschi E, De Ponti F, Bégaud B, Pariente A. Addition of dipeptidyl peptidase-4 inhibitors to sulphonylureas and risk of hypoglycaemia: systematic review and meta-analysis. BMJ. 2016;353:i2231.
58. Nauck MA. Update on developments with SGLT2 inhibitors in the management of type 2 diabetes. Drug design, development and therapy. 2014;8:1335.
59. McCulloch DK, Nathan DM, Mulder JE. Management of persistent hyperglycemia in type 2 diabetes mellitus. 2015. Available at: http://www.uptodate.com/contents/management-of-persistent-hyperglycemia-in-type-2-diabetes-mellitus?source=search_result&search=management+of+persistent+hyperglycaemia+in+type+II+diabetes+mellitus&selectedTitle=1~150 (Last accessed on 27 Aug 2015)

## **Postprandial hyperglycemia**

### **RSSDI 2017 recommendations**

#### **Recommended care**

- Postprandial hyperglycemia is defined as 2-h plasma glucose level of > 200 mg/dL during OGTT with 75 g anhydrous glucose.
- Postprandial hyperglycemia is harmful and should be addressed.
- Treatment strategies to lower PPG in people with postprandial hyperglycemia should be implemented.
- PPG should be measured 1–2 h after a meal.
  - The target for PPG is 160 mg/dL as long as hypoglycemia is avoided.
- A variety of both non-pharmacologic and pharmacologic therapies should be considered to target PPG.
  - Medical nutrition therapy (MNT) that includes diet with low glycemic load is recommended in all patients with postprandial hyperglycemia.
  - Pharmacological agents to lower postprandial hyperglycemia include:
    - AGIs (acarbose or voglibose), DPP4 inhibitors, or GLP-1 analogues (preferably short acting) are recommended as first-line therapy.
    - Glinides and short-acting sulfonylureas are recommended as second-line agents.
    - Rapid-acting insulin analogues should be preferred over the regular insulin when postprandial hyperglycemia is a concern.
    - Combination therapy of AGI with other agents may be considered for better control of postprandial hyperglycemia.
  - SMBG should be considered because it is currently the most practical method for monitoring postprandial glycemia.
  - Efficacy of treatment regimens should be monitored as frequently as needed to guide therapy towards achieving PPG target.

#### **Limited care**

- The principles for management of postprandial hyperglycemia are as for recommended care.

#### **Preamble**

Poorly controlled diabetes is associated with increased risk of micro- and macrovascular complications which further depend upon both fasting and PPG levels [1–4]. Evidence from large controlled clinical trials suggest that intensive glycemic control can significantly decrease the development and/or progression of these complications [1, 5–8]. However, tight glycemic control needs to be instituted early in the disease course so that legacy effect can be seen as reduced number of complications even after many years. The relationship between hyperglycemia and CVD is complex with evidence suggesting that an acute increase of glycemia, particularly after a meal, contributes to the increased risk of diabetes-related complications and have a direct detrimental effect on CVD in patients with T2DM [9–11]. Until recently, the predominant focus of diabetes treatment has been on lowering A1C levels, with emphasis on FPG [12, 13]. Nevertheless, control of fasting hyperglycemia alone is insufficient to obtain optimal glycemic control. Emerging evidence suggests that reducing PPG excursion is as important or perhaps more important for achieving desired glycemic targets [14]. Usually A1C is supposed to be largely contributed by FPG when it is away from target. As and when it comes closer to the target, PPG starts taking upper hand and contributes predominantly. But in Asian Indians, scenario remains quite different. In Indians, the PPG remains relatively high all across the A1C spectrum and especially very high even at higher A1C values [15–17]. Relative contribution of postprandial hyperglycemia to A1C levels in patients with T2DM is higher than FPG levels when the A1C levels are below 7.5% and it decreases progressively as A1C levels increase [18]. Therefore, targeting both PPG and FPG is an important strategy for achieving optimal glycemic control. The purpose of these recommendations is to assist clinicians in developing strategies to consider and effectively manage postmeal glucose in people with T2DM in Asian countries.

#### **Considerations**

India has a high prevalence of diabetes and onset of diabetes is a decade early. Postprandial hyperglycemia is more prominent in Indians due to high traditional diets with high glycemic index. Literature is limited regarding postprandial hyperglycemia despite a definite role in micro and macrovascular complications.

#### **Rationale and evidence**

##### *Definition of postprandial hyperglycemia*

- ADA 2017 defines postprandial hyperglycemia as a 2-h plasma glucose level of more than 200 mg/dL (11.1 mmol/L) during an OGTT. It recommends the use of glucose load equivalent to 75 g anhydrous glucose dissolved in water as prescribed by WHO. On the other hand, IDF 2011 defines postprandial hyperglycemia as a plasma glucose level of 140 mg/dL or more after 1–2 h of food intake [19, 20].
- Asian Indians display marked rise in prandial glucose excursion after consumption of 75 g of bread meal compared to their Caucasian counterparts [21, 22].

##### *Harmful effects of postprandial hyperglycemia*

- Elevations in PPG are due to the loss of first phase insulin secretion, decreased insulin sensitivity in peripheral tissues, and consequent decreased suppression of hepatic glucose output after meals due to insulin deficiency [13].
- Postprandial hyperglycemia is an independent risk factor for the development of several complications including [23]:
  - Macrovascular disease
  - Retinopathy
  - Cancer
  - Impaired cognitive function in elderly people with T2DM
  - Increased carotid intima-media thickness
  - Decreased myocardial blood volume and myocardial blood flow
  - Oxidative stress, inflammation, and endothelial dysfunction
  - Renal failure
  - Neuropathy
  - Amputation
- Evidence from an Indian study in subjects with a history of T2DM for more than 25 years suggests that postprandial hyperglycemia was associated with increased risk of both diabetic nephropathy and neuropathy [15, 24]. The Kumamoto study suggested reductions in retinopathy and nephropathy with reduced PPG [6, 19].
- The causes of postprandial hyperglycemia are influenced by many factors which include a rapid flux of glucose from the gut, impaired insulin release, and endogenous glucose production by the liver and peripheral IR [11].

##### *Addressing postprandial hyperglycemia*

- Currently, there is lack of data linking improved clinical outcomes with that of correcting postprandial hyperglycemia. Neither the Hyperglycemia and Its Effect After Acute Myocardial Infarction on Cardiovascular Outcomes in Patients With Type 2 Diabetes Mellitus (HEART2D) study nor the Nateglinide and Valsartan in Impaired Glucose Tolerance Outcomes Research (NAVIGATOR) study could demonstrate direct benefit of lowering postprandial hyperglycemia in reducing CVD in patients with T2DM [25–27].
- However, emerging evidence indicates that agents that target PPG show significant positive trends in risk reduction for all selected CV events. Findings from the Study to Prevent Non-Insulin-Dependent Diabetes Mellitus (STOP-NIDDM) trial also showed that treating people with IGT with acarbose is associated with a significant reduction in the risk of CVD and hypertension [28]. The ongoing Acarbose Cardiovascular Evaluation Trial (ACE) will further elucidate whether acarbose therapy can reduce cardiovascular-related morbidity and mortality in patients with IGT who have established CAD or acute coronary syndrome (ACS) and will also determine if acarbose therapy can prevent or delay transition to T2DM in this patient population [29]. The panel opined that addressing postprandial hyperglycemia is important with a recommended target of 160 mg/dL, as long as hypoglycemia is avoided.
- Postprandial hyperglycemia is an important pathophysiological state contributing to the pathogenesis of CVD in people with and without diabetes. It should be routinely monitored in T2DM patients. Serum glucose level 2-h post-OGTT must be performed as it is a powerful predictor of all-cause premature death and CV risk and a better indicator than FPG [30–32]. Management of postprandial hyperglycemia is central to long-term glycemic control and an essential part of CVD prevention in IGT and T2DM. The level of implementation of routine screening for postmeal hyperglycemia, using the OGTT, should be improved in the Asia-Pacific region, combined with wider use of effective interventions to manage postprandial hyperglycemia [33].

##### ***Strategies to prevent postprandial hyperglycemia***

*Non-pharmacological*

- Physical activity and MNT are the cornerstones of non-pharmacologic therapy in T2DM patients.
- In general, adults with diabetes are advised to perform moderate-intensity aerobic physical activity at least 150 min/week. They are also advised to perform resistance training three times per week; even older people are advised to perform this [19]. A randomized crossover study showed that, in T2DM patients, walking after meals is more effective for lowering postprandial glycemia [34].
- Traditional Asian Indian and Chinese diets are carbohydrate-rich (as high as 80% of the macronutrient composition) with high glycemic index values [35]. Consumption of rice is very high in South India which is associated with four- to fivefold increase in risk of diabetes [36]. The higher carbohydrate load in the Indian diet leads to greater PPG excursion, increased glucosidase, and incretin activity in the gut which leads to higher lipaemic peaks and associated CVD [35]. Evidence suggests that diets with low glycemic index values are beneficial in controlling postprandial hyperglycemia [13, 37, 38].
- To promote effective postprandial hyperglycemia control, the panel emphasized on advising patients on MNT which should include:
  - Carbohydrate should constitute 45–65% of total caloric intake, with a minimum of 130 g/day for adults
    - Consumption of low glycemic index foods
    - Increase intake of soluble and insoluble fiber
    - Consumption of fruits and vegetables in place of refined carbohydrate
  - 10–15% proteins and fats less than 30%
  - Saturated fats should be less than 7%
  - Cholesterol less than 300 mg/day
    - *Pharmacological*
- Based on limited Indian evidence available from literature, the panel relied on expert opinion for pharmacological management of postprandial hyperglycemia which includes the following:

- Therapies which have been available for some time include AGIs (acarbose and voglibose), glinides (rapid-acting insulin secretagogues), short-acting sulfonylureas (glipizide), and insulins (rapid-acting human insulins/insulin analogues and biphasic [premixed] human insulins/insulin analogues).
- In addition, new classes of therapies for managing postprandial hyperglycemia such as GLP-1 analogues (preferably the short-acting exenatide) and DPP-4 inhibitors have shown significant benefits in reducing PPG excursions and lowering A1C.
- Use of glinides is limited to the treatment of postprandial hyperglycemia only if sulfonylureas are contraindicated or economic consideration prohibits the use of newer costlier agents.
- AGIs (acarbose, miglitol, and voglibose) can be used as first-line drug in early T2DM, as well as in combination with nearly all established OADs and insulin. AGIs have been shown to effectively control postprandial hyperglycemia while providing additional benefits in terms of CV risk protection [39, 40]. Moreover, AGIs tend to inhibit carbohydrate absorption from gut which can be of particular importance in Indian settings where there are increased odds for PPG and lipid excursion due to consumption of diets with high glycemic index.

#### **Implementation**

Frequent monitoring of glucose levels using techniques such as SMBG can significantly improve glycemic control besides detecting PPG excursion. SMBG is currently the optimal method for assessing plasma glucose levels. Evidence suggests that structured SMBG followed by therapeutic interventions results in greater A1C reduction in people with T2DM compared with programs without structured SMBG [41–43]. Therefore, the panel opined that SMBG with appropriate patient education is necessary for optimal management of postmeal hyperglycemia.

**References**

1. Chawla A, Chawla R, Jaggi S. Microvasular and macrovascular complications in diabetes mellitus: distinct or continuum? Indian Journal of Endocrinology and Metabolism. 2016;20(4):546–551.
2. Ketema EB, Kibret KT. Correlation of fasting and postprandial plasma glucose with HbA1c in assessing glycemic control; systematic review and meta-analysis. Archives of Public Health. 2015;73:43.
3. The relationship of glycemic exposure (HbA1c) to the risk of development and progression of retinopathy in the diabetes control and complications trial. Diabetes. 1995;44(8):968–83.
4. Stratton IM, Adler AI, Neil HA, Matthews DR, Manley SE, Cull CA, et al. Association of glycaemia with macrovascular and microvascular complications of type 2 diabetes (UKPDS 35): prospective observational study. BMJ. 2000;321(7258):405–12.
5. Diabetes Control and Complications Trial (DCCT) Research Group. The effect of intensive treatment of diabetes on the development and progression of long-term complications in insulin-dependent diabetes mellitus. N Engl J Med 1993;329(14):977–86.
6. Ohkubo Y, Kishikawa H, Araki E, Miyata T, Isami S, Motoyoshi S et al. Intensive insulin therapy prevents the progression of diabetic microvascular complications in Japanese patients with noninsulin-dependent diabetes mellitus: a randomized prospective 6-year study. Diabetes Res Clin Pract 1995;28(2):103–17.
7. UK Prospective Diabetes Study (UKPDS) Group. Intensive blood-glucose control with sulphonylureas or insulin compared with conventional treatment and risk of complications in patients with type 2 diabetes (UKPDS 33). Lancet 1998;352(9131):837–53.
8. UK Prospective Diabetes Study (UKPDS) Group. Effect of intensive blood-glucose control with metformin on complications in overweight patients with type 2 diabetes (UKPDS 34). Lancet 1998; 352(9131):854–65.
9. Martín-Timón I, Sevillano-Collantes C, Segura-Galindo A, del Cañizo-Gómez FJ. Type 2 diabetes and cardiovascular disease: have all risk factors the same strength? World Journal of Diabetes. 2014;5(4):444–470.
10. Ceriello A, Hanefeld M, Leiter L, Monnier L, Moses A, Owens D, et al. Postprandial glucose regulation and diabetic complications. Arch Intern Med. 2004;164(19):2090–5.
11. Sudhir R, Mohan V. Postprandial hyperglycemia in patients with type 2 diabetes mellitus. Treat Endocrinol. 2002;1(2):105–16.
12. Nathan DM, Buse JB, Davidson MB, Hein RJ, Holman RR, Sherwin R et al. Management of hyperglycemia in type 2 diabetes: a consensus algorithm for the initiation and adjustment of therapy: a consensus statement from the American Diabetes Association and the European Association for the Study of Diabetes. Diabetes Care 2006;29(8):1963–72.
13. Jindal R, Gupta N, Siddiqui MA, Wangnoo SK. Post-prandial hyperglycaemia. JIACM 2013;14(3–4):242–6.
14. Ceriello A. The glucose triad and its role in comprehensive glycaemic control: current status, future management. Int J Clin Pract 2010;64(12):1705–11.
15. Aravind SR, Saboo B, Sadikot S et al. Consensus statement on management of postprandial hyperglycemia in clinical practice in India. Journal of the Association of Physicians of India; Vol. 63; August 2015:45–58
16. Wang JS, Tu ST, Lee IT et al. Contribution of postprandial glucose to excess hyperglycaemia in Asian type 2 diabetic patients using continuous glucose monitoring. Diabetes Metab Res Rev. 2011; 27: 79–84.
17. Talib SH, Bhattu SR, Korpe JS. An observational study on correlation of fasting and postprandial glycemic status to various HbA1C quintiles in type II diabetics. IOSR Journal of Dental and Medical Sciences. Volume 6, Issue 1 (Mar-Apr 2013), PP 14–19.
18. Monnier L, Lapinski H, Colette C. Contributions of fasting and postprandial plasma glucose increments to the overall diurnal hyperglycemia of type 2 diabetic patients. Diabetes Care. 2003;26(3):881–5.
19. Marathe PH, Gao HX, Close KL. American Diabetes Association standards of medical care in diabetes 2017. Journal of Diabetes. 2017.
20. Ceriello A, Barakat M, Bahendeka S, Colagiuri S, Gerich J, Hanefeld M et al. Guideline for management of post-meal glucose in diabetes. International Diabetes Federation 2011:1–37; Available at: http://www.idf.org/sites/default/files/postmeal%20glucose%20guidelines.pdf. (Last accessed on 27 Aug 2015).
21. Dickinson S, Colagiuri S, Faramus E, Petocz P, Brand-Miller JC. Postprandial hyperglycemia and insulin sensitivity differ among lean young adults of different ethnicities. J Nutr. 2002;132(9):2574–9.
22. Tan WS, Tan SY, Henry CJ. Ethnic variability in glycemic response to sucrose and isomaltulose. Nutrients. 2017;9(4):347.
23. Singh SK. Post-prandial hyperglycemia. Indian J Endocr Metab 2012;16(Suppl 2):S245–7.
24. Mohan V, Vijayaprabha R, Rema M. Vascular complications in long-term south Indian NIDDM of over 25 years’ duration. Diabetes Res Clin Pract. 1996;31(1–3):133–40.
25. Raz I, Wilson PW, Strojek K, Kowalska I, Bozikov V, Gitt AK, et al. Effects of prandial versus fasting glycemia on cardiovascular outcomes in type 2 diabetes: the HEART2D trial. Diabetes Care 2009;32(3):381–6.
26. Holman RR, Haffner SM, McMurray JJ, Bethel MA, Holzhauer B, Hua TA, et al. Effect of nateglinide on the incidence of diabetes and cardiovascular events. N Engl J Med. 2010;362(16):1463–76.
27. Ceriello A. Postprandial hyperglycemia and cardiovascular disease: is the HEART2D study the answer? Diabetes Care 2009; 32(3):521–522.
28. Chiasson JL, Josse RG, Gomis R, Hanefeld M, Karasik A, Laakso M; Acarbose for prevention of type 2 diabetes mellitus: the STOP-NIDDM randomised trial. Lancet. 2002;359:2072–7.
29. Acarbose Cardiovascular Evaluation Trial (ACE). NCT00829660. Available at: https://www.clinicaltrials.gov/ct2/show/NCT00829660?term=NCT00829660&rank=1
30. Nakagami T, DECODA Study Group. Hyperglycaemia and mortality from all causes and from cardiovascular disease in five populations of Asian origin. Diabetologia 2004;47:385–94
31. The DECODA Study Group. Age- and sex-specific prevalence of diabetes and impaired glucose regulation in 11 Asian cohorts. Diabetes Care 2003;26:1770–80
32. Sacks DB. A1C versus glucose testing: a comparison. Diabetes Care. 2011;34(2):518–523.
33. Sheu WH, Rosman A, Mithal A, Chung N, Lim YT, Deerochanawong C, et al. Addressing the burden of type 2 diabetes and cardiovascular disease through the management of postprandial hyperglycaemia: an Asian-Pacific perspective and expert recommendations. Diabetes Res Clin Pract. 2011;92(3):312–21.
34. Reynolds AN, Mann JI, Williams S, Venn BJ. Advice to walk after meals is more effective for lowering postprandial glycaemia in type 2 diabetes mellitus than advice that does not specify timing: a randomised crossover study. Diabetologia. 2016;59(12):2572–8.
35. Joshi SR. Post-prandial carbohydrate modulation via gut—Indian perspective. J Assoc Physicians India. 2010;58:665.
36. Mohan V, Radhika G, Sathya RM, et al. Dietary carbohydrates, glycaemic load, food groups and newly detected type 2 diabetes among urban Asian Indian population in Chennai, India. The British Journal of Nutrition. 2009:1–9.
37. Ghosh JM. Trial of low glycemic diet and acarbose therapy for control of post-prandial hyperglycemia in type 2 diabetes mellitus, preliminary report. Int J Diab Dev Ctries. 2005;25(3):80–4. Available at: http://www.diabetes.org.in/journal/2005_july-sept/original_article3.pdf (Last accessed on 27 Aug 2015).
38. Augustin LS, Kendall CW, Jenkins DJ, Willett WC, Astrup A, Barclay AW, Björck I, Brand-Miller JC, Brighenti F, Buyken AE, Ceriello A. Glycemic index, glycemic load and glycemic response: an International Scientific Consensus Summit from the International Carbohydrate Quality Consortium (ICQC). Nutrition, Metabolism and Cardiovascular Diseases. 2015;25(9):795–815.
39. Kawamori R, Tajima N, Iwamoto Y, Kashiwagi A, Shimamoto K, Kaku K, et al. Voglibose for prevention of type 2 diabetes mellitus: a randomised, double-blind trial in Japanese individuals with impaired glucose tolerance. Lancet. 2009;373(9675):1607–14.
40. Kitano D, Chiku M, Li Y, Okumura Y, Fukamachi D, Takayama T, et al. Miglitol improves postprandial endothelial dysfunction in patients with acute coronary syndrome and new-onset postprandial hyperglycemia. Cardiovasc Diabetol. 2013;12:92.
41. Polonsky WH, Fisher L, Schikman CH, Hinnen DA, Parkin CG, Jelsovsky Z, et al. Structured self-monitoring of blood glucose significantly reduces A1C levels in poorly controlled, noninsulin-treated type 2 diabetes: results from the Structured Testing Program study. Diabetes Care. 2011;34(2):262–7.
42. Franciosi M, Lucisano G, Pellegrini F, Cantarello A, Consoli A, Cucco L, et al. ROSES: role of self-monitoring of blood glucose and intensive education in patients with type 2 diabetes not receiving insulin. A pilot randomized clinical trial. Diabet Med. 2011;28(7):789–96.
43. Durán A, Martín P, Runkle I, Pérez N, Abad R, Fernández M, et al. Benefits of self-monitoring blood glucose in the management of new-onset type 2 diabetes mellitus: the St Carlos Study, a prospective randomized clinic-based interventional study with parallel groups. J Diabetes. 2010;2(3):203–11.

## **Clinical monitoring**

### **RSSDI 2017 recommendations**

#### **Recommended care**

- Monitor blood glucose control by measuring A1C using high-precision methods standardized to criteria aligned to the international reference values and subject to stringent quality assurance testing when no conditions are present in a patient that would preclude its accurate measurement.
- SMBG helps in adjusting treatment medications and nutrition therapy to achieve A1C targets and also to detect and prevent asymptomatic hypoglycemia and glucose variability. In patients on insulin, a combination of A1C and SMBG is useful in achieving glycemic control.
- Measure A1C every 2 to 6 months depending on level, stability of blood glucose control. and changes in therapy and report A1C results in percentages.
- Provide A1C result, in the laboratory, before the clinical consultation.
- Anemia and abnormal hemoglobin may affect the values obtained for A1C in some assays. To determine whether abnormal hemoglobin is present, use high-performance liquid chromatography (HPLC) or mass spectrometry. Anemia has to be corrected before a proper diagnosis based on A1C values is made.
- Estimated average glucose ([eAG] reported in either mmol/l or mg/dL) is derived from A1C. Only few countries have chosen to report eAG due to its limitations and lack of applicability to all ethnic groups. It may help people with diabetes relate their A1C to daily glucose monitoring levels or highlight when A1C is inappropriate.
- Measure blood glucose when patients are hospitalized, either at site-of-care or in the laboratory. Site-of-care capillary blood glucose meters should be monitored by certified quality assurance schemes. Ascertain whether meters are calibrated against plasma or blood

#### **Limited care**

- If A1C measurement is not available, blood glucose measured either at site-of-care or in the laboratory could be used for clinical monitoring.
- Site-of-care capillary blood glucose meters should be quality controlled by certified quality assurance schemes or by reference to laboratory methods.

### **Targets of glucose control**

#### **Recommended care**

- Advise people with diabetes that maintaining an A1C below 7.0% minimizes the risk of developing complications.
- A lower A1C target may be considered if it is easily and safely achieved.
- A higher A1C target may be considered for people with comorbidities or when previous attempts to optimize control have been associated with unacceptable hypoglycemia.
- An individual’s A1C target should be regularly reviewed by taking benefits, safety, and tolerability into account.
- Treatment should be reviewed and modified if A1C level is above the agreed target on two consecutive occasions.
- Advice those in whom target A1C levels cannot be reached that any improvement is beneficial.
- Target values for glucose control for A1C and capillary plasma glucose are as follows:

**Table:** 

	Normal	Target
A1C	< 5.7%/39 mmol/mol	< 7.0%/53 mmol/mol
FPG	5.5 mmol/l (100 mg/dL)	6.5 mmol/l (115 mg/dL)
PPG	7.8 mmol/l (140 mg/dL)	9.0 mmol/l (160 mg/dL)

#### **Limited care**

- The principles are as for recommended care including assessment of diabetes control by A1C measurement. In very limited settings, diabetes control may need to be based on measurement of plasma glucose levels alone.

#### **Other clinical monitoring**

**Table:** 

Type of monitoring	Recommended care	Limited care
Complete history and physical examination	• A complete history and physical examination is recommended • Periodicity: annually	• As for recommended care
Ophthalmic	• Detailed exam by qualified ophthalmologist • Dilated • Periodicity: at diagnosis and every 2 years if there is no retinopathy	• If ophthalmologists are not available need to adapt low cost technology to enable GPs to learn and use fundus photography
Smoking cessation	• If present counselling by physician at every visit	• As for recommended care
BP measurement	• BP measurement at each visit	• As for recommended care
Measurement of lipids	• At diagnosis or at age 40 and periodically (6 monthly) thereafter	• At diagnosis or at 40 at least
Screening for CVD	• A resting ECG may give useful information on baseline cardiac status and for future reference	• As for recommended care
Microalbuminuria	• At diagnosis and annually thereafter	• If resources are limited and technical issues may consider use of ACEI/ARB if BP is > 140/80
Distal peripheral neuropathy	• At diagnosis and at least annually • Test for vibration with 128 Hz tuning fork or a 10-g monofilament, pinprick sensation ankle jerk	• As recommended by IDF • Additional training required
Peripheral arterial disease	• At diagnosis • History of claudication, distal pulses, and ABI	• As for recommended care • Additional training required
Comprehensive foot care	• At diagnosis and annually • Assessment of foot pulses and testing for loss of protective sensation (10 g monofilament plus testing any one of vibration using 128 Hz tuning fork, pinprick sensation, ankle reflexes, or vibration perception threshold)	• As for recommended care • Additional training required

#### **Preamble**

Monitoring of blood glucose levels is critically important to ensure good glycemic control. It is considered the cornerstone of diabetes care that helps both physicians and patients to adjust the therapy according to patient’s need. Following clinical testing to assess levels of control and progression of T2DM, most guidelines recommend clinicians to perform frequent monitoring of glycemic status by measurement of A1C as a follow-up care of individuals with diabetes [1, 2]. Moreover, A1C measurement is the gold standard for the therapeutic management of diabetes in both research and in clinical settings, which involves assessing glycemic control over the previous 2–3 months [3, 4]. Long-term hyperglycemia as measured by A1C has been shown to be strongly related to development of diabetic microvascular complications though its relation to development of macrovascular complications is less clear. Therefore, patients who are not at targets or at increased risk of developing complications require more intensive monitoring, ranging from frequent self-monitored glucose [3, 5] to continuous glucose monitoring (CGM) [6, 7] to assess daily variations in blood glucose levels [8]. The current recommendations provide an insight on the importance and frequency of monitoring to be performed in order to facilitate medication and lifestyle changes when average A1C values remain above targets levels.

#### **Considerations**

The decision on clinical monitoring of glycemic levels in T2DM patients was based on the local factors such as availability of newer technologies and cost of monitoring that were reviewed in Indian context.

#### **Rationale and evidence**

##### *A1C for monitoring blood glucose*

- Several guidelines and literature pertaining to monitoring emphasize on adjustments to the treatment based on glycemic measurements, methods available for monitoring, and their quality implementation. Evidence suggests that regular monitoring of A1C will facilitate identification of patients with poor glycemic control and help both physicians and patients to take necessary steps to achieve desired glycemic targets [9, 10]. Though frequent monitoring of A1C is associated with reduced diabetes-related complications and improved metabolic control [10, 11], most patients do not understand or are not aware of importance of glycemic monitoring. Therefore, it becomes absolutely necessary to empower the patients with knowledge and understanding on A1C levels for optimal glycemic control that will in turn motivate them to effectively manage their diabetes [10, 12].
- The International Federation of Clinical Chemistry and Laboratory Medicine (IFCC) working group on A1C standardization have established a reference measurement procedure (HPLC-MS or HPLC-CE) for A1C embracing the concept of metrological traceability [13].
- Though IDF recommends reporting IFCC units (mmol A1C per mol unglycated hemoglobin), the panel suggested reporting A1C in DCCT aligned values (%) as majority of the physicians are not familiar with new IFCC units. However, it was emphasized that the healthcare professionals should be made aware of the new IFCC units and encouraged to practice them during routine clinical practice.
- The concept of eAG was introduced following introduction of continuous ambulatory blood glucose monitoring [14]. The eAG may help people with diabetes relate their A1C to daily glucose monitoring and highlight any inaccuracies in A1C measurement relative to glucose levels [15]. There are calculators available for converting A1C to eAG in both millimoles per liter and milligrams per deciliter. Measurement of timed glucose levels are often recommended as a substitute for A1C when the latter is either unavailable or inappropriate.
- In a meta-analysis, *Cavagnolli et al*. found that A1C values are higher in blacks, Asians, and Latinos when compared to white persons [16]. Even though this observation is made in non-diabetes patients, authors claim that these differences might influence the use of same A1C point to diagnose diabetes in all ethnic populations.
- Abnormal hemoglobin levels are known to affect A1C values in a way that can significantly alter the results with regard to diabetes control [17]. Therefore, it is important to consider hematological factors that can confound A1C levels in people with diabetes, best detected using HPLC-based assays.
- Anemia significantly impacts A1C levels. In a cross-sectional study, *Rajagopal et al*. have found that the mean A1C in patients with controlled diabetes with IDA was significantly higher than those without IDA (7.86 ± 0.11 vs 5.45 ± 0.038% (*p* < 0.05)). They also found that A1C values were higher with the reduction of total hemoglobin (*p* < 0.05) [18]. Similarly, *Madhu et al*. have found significantly higher A1C levels in IDA subjects than healthy people (5.51 ± 0.696 vs 4.85 ± 0.461%, *p* < 0.001). They also found a significant decline in A1C levels after iron supplementation (*p* < 0.001) [19]. Therefore, A1C results in diabetes patients with IDA should be interpreted carefully. IDA has to be corrected before a proper diagnosis is made.
- Measurement of blood glucose using blood glucose meters on admission to hospital wards helps to identify patients with hypoglycemia or hyperglycemia. Considering that in developing nations like India, where cost is major barrier for monitoring, these devices should be accurate and cost-effective and field testing specifically tailored for Asian and Indian needs is imperative. Such data is available from only one study in India by Dr. Mohan’s group from Chennai that evaluated the performance of glucose meter for Indian conditions across different values and temperatures [20].

##### *Glucose measurement*

- Plasma glucose is the preferred measure of most modern laboratories. Whole blood gives lower readings due to the volume occupied by hemoglobin. Capillary blood glucose strips measure the glucose in the plasma of the capillary blood sample but may be calibrated to give results either as plasma or sometimes whole blood glucose (check meter instructions).

#### **Implementation**

There should be access to a laboratory or site-of-care test monitored by certified quality assurance schemes for measurement of A1C. People in whom A1C measurement is inappropriate must be identified by careful review of hematological parameters and other factors that can affect A1C values. Provision of capillary blood glucose meters and strips needs to be assured in hospitals and clinics. It is important to ascertain whether there are contraindications for use of a particular type of glucose meter in a particular patient. It is essential to establish whether meters report values for plasma or blood and to ensure that schemes for monitoring the quality of their output are in place. Blood glucose meters should be calibrated on regular basis and their use in hospitals should be restricted to trained personnel.

**References**

1. Golden S, Boulware LE, Berkenblit G, Brancati F, Chander G, Marinopoulos S, et al. Use of glycated hemoglobin and microalbuminuria in the monitoring of diabetes mellitus. Evid Rep Technol Assess (Summ). 2003;(84):1–6.
2. World Health Organization. Use of glycated haemoglobin (HbA1c) in the diagnosis of diabetes mellitus 2011
3. Pimazoni-Netto A, Rodbard D, Zanella on behalf of the Diabetes Education and Control Group MT. Rapid improvement of glycemic control in type 2 diabetes using weekly intensive multifactorial interventions: structured glucose monitoring, patient education, and adjustment of therapy—a randomized controlled trial. Diabetes Technology & Therapeutics. 2011;13(10):997–1004.
4. Rahaghi FN, Gough DA. Blood glucose dynamics. Diabetes Technol Ther. 2008;10(2):81–94.
5. Kirk JK, Stegner J. Self-monitoring of blood glucose: practical aspects. J Diabetes Sci Technol. 2010;4(2):435–9.
6. Klonoff DC, Buckingham B, Christiansen JS, Montori VM, Tamborlane WV, Vigersky RA, et al. Continuous glucose monitoring: an Endocrine Society Clinical Practice Guideline. J Clin Endocrinol Metab. 2011;96(10):2968–79.
7. Bailey TS, Grunberger G, Bode BW, Handelsman Y, Hirsch IB, Jovanovič L, Roberts VL, Rodbard D, Tamborlane WV, Walsh J. American Association of Clinical Endocrinologists and American College of Endocrinology 2016 outpatient glucose monitoring consensus statement. Endocrine Practice. 2016;22(2):231–61.
8. Healy SJ, Dungan KM. Monitoring glycemia in diabetes. Med Clin North Am. 2015;99(1):35–45.
9. Larsen ML, Hørder M, Mogensen EF. Effect of long-term monitoring of glycosylated hemoglobin levels in insulin-dependent diabetes mellitus. N Engl J Med. 1990;323(15):1021–5.
10. Sherwani SI, Khan HA, Ekhzaimy A, Masood A, Sakharkar MK. Significance of hba1c test in diagnosis and prognosis of diabetic patients. Biomarker insights. 2016;11:95.
11. The effect of intensive treatment of diabetes on the development and progression of long-term complications in insulin-dependent diabetes mellitus. The Diabetes Control and Complications Trial Research Group. N Engl J Med. 1993;329(14):977–86.
12. Heisler M, Piette JD, Spencer M, Kieffer E, Vijan S. The relationship between knowledge of recent HbA1c values and diabetes care understanding and self-management. Diabetes Care. 2005;28(4):816–22.
13. John G, English E. IFCC standardised HbA(1c): should the world be as one? Clin Chem Lab Med. 2012;50(7):1243–8.
14. Nathan DM, Kuenen J, Borg R, Zheng H, Schoenfeld D, Heine RJ et al. Translating the A1C assay into estimated average glucose values. Diabetes Care 2008;31(8):1473–8.
15. Bethesda. The A1C test and diabetes. National Diabetes Information Clearinghouse. NIH Publication No.: 11-7816.: National Institute of Diabetes and Digestive and Kidney Diseases, National Institutes of Health; 2011. Available at: http://permanent.access.gpo.gov/gpo22893/A1C_Test_DM-508.pdf (Last accessed on 25 Aug 2015)
16. Cavagnolli G, Pimentel AL, Freitas PA, Gross JL, Camargo JL. Effect of ethnicity on HbA1c levels in individuals without diabetes: systematic review and meta-analysis. PLOS One. 2017;12(2):e0171315.
17. Sacks DB. Hemoglobin variants and hemoglobin A1c analysis: problem solved? Clin Chem 2003;49:1245–7.
18. Rajagopal L, Arunachalam S, Ganapathy S, Ramraj B. Impact of iron deficiency anemia on glycated hemoglobin (HbA1c) levels in diabetics with controlled plasma glucose levels. Annals of Pathology and Laboratory Medicine. 2017;4(2):A148–152.
19. Madhu SV, Raj A, Gupta S, Giri S, Rusia U. Effect of iron deficiency anemia and iron supplementation on HbA1c levels—implications for diagnosis of prediabetes and diabetes mellitus in Asian Indians. Clinica Chimica Acta. 2016.
20. Mohan V, Deepa R, Shefali AK, Poongothai S, Monica M, Karkuzhali K. Evaluation of One Touch HORIZON—a highly affordable glucose monitor. J Assoc Physicians India. 2004;52:779–82.

## **Self-monitoring of blood glucose**

### **RSSDI 2017 recommendations**

#### **Recommended care**

- SMBG is useful to people with diabetes who have the required knowledge, skills, and willingness to use the information obtained through testing to actively adjust treatment with the help of the treating physician and to enhance understanding of diabetes and assess the effectiveness of the management plan on glycemic control
- The purpose(s) of performing SMBG and using SMBG data should be agreed between the person with diabetes and the healthcare provider.
- SMBG on an ongoing basis should be available to those people with diabetes using insulin.
- SMBG protocols (intensity and frequency) should be individualized to address each individual’s specific educational/behavioral/clinical requirements/specific needs and goals (to identify/prevent/manage acute hyper- and hypoglycemia) and provider requirements for data on glycemic patterns and to monitor impact of therapeutic decision-making.
- Intensive/regular SMBG may be recommended if a person with diabetes is on multiple daily insulin injections, pregestational diabetes on insulin, history of hypoglycemia unawareness, have brittle diabetes, or with poor metabolic control on multiple OADs and/or basal insulin SMBG should be performed at least as often as insulin is administered:
  - For patients on intensive insulin regimens who are on multiple doses of insulin or on insulin pumps should be tested three or more times daily (all premeals, postmeals, bedtime, prior to exercise).
- SMBG plays important role when a patient suspect low blood glucose or after treating low blood glucose until they are normoglycemic and prior to critical tasks such as driving. For many patients, this will require testing 6–10 (or more) times daily, although individual needs may vary. Similar considerations apply for pregnant women on insulin.
  - Pregnant women with insulin-treated diabetes should be advised to perform SMBG on a daily basis, failing which, at least weekly monitoring should be encouraged.
  - Ideal SMBG is seven tests/day, i.e., three before and three after each meal and one test at 3 am. As a compromise one fasting test and three tests each after breakfast, lunch, and dinner daily may be more feasible and acceptable, which can further be individualized to twice or thrice a week as the pregnancy advances. Two-hour postmeal monitoring may be easier to remember as this timing is routinely used.
- In accordance with the sick day rule, the frequency of SMBG should be increased in special situations like fever, vomiting, and persistent polyuria with uncontrolled blood glucose, especially if abdominal pain or rapid breathing is present. Ketone test should be performed as and when needed.
- SMBG accuracy is instrument and user dependent, so it is important to evaluate each patient’s monitoring technique, both initially and at regular intervals thereafter. The ongoing need for and frequency of SMBG should be reevaluated at each routine visit.
- SMBG should be considered for people using oral glucose-lowering medications as an optional component of self-management and in association with A1C testing:
  - To provide information on, and help avoid, hypoglycemia.
  - To assess changes in blood glucose control due to medications and lifestyle changes.
  - To monitor the effects of foods on postprandial glycemia.
  - To monitor changes in blood glucose levels during intercurrent illness.
- SMBG may be useful in T2DM, during periods of acute illness; using sulfonylureas or glinides as combination or monotherapy; to identify hypoglycemia especially in the first 3 months of starting sulfonylurea; in those who experience episodes of hypoglycemia and those who have reduced awareness of hypoglycemia; are drivers and those who fast; and under preconception care.
- Regular use of SMBG should not be considered part of routine care where diabetes is well controlled by nutrition therapy or oral medications alone.
- Structured assessment of self-monitoring skills, the quality and use made of the results obtained, and of the equipment used should be made annually.

#### **Limited care**

- SMBG using meters with strips should be considered for people with diabetes using insulin or drugs like sulfonylurea and glinides.

#### **Preamble**

Frequent and precise blood glucose monitoring and concomitant optimal adjustment of insulin to carbohydrate intake and exercise are the basis of diabetes treatment. The SMBG is an essential component of the modern diabetes treatment and the most uncomplicated and commonly used strategy of interim glucose monitoring across the globe [1]. Established advantages with SMBG include achieving target A1C, reducing glucose variability, and prediction of severe hypoglycemia [2]. Therefore, it has been recommended for achieving a specific level of glycemic control and to prevent hypoglycemia in people with diabetes. The objective of SMBG is to assemble detailed information about blood glucose levels at various time points to enable maintenance of a more constant glucose level by more precise regimens. It can be used to aid in the adjustment of a therapeutic regimen in response to blood glucose values and to benefit individuals regulate their dietary intake, physical activity, and insulin doses to improve glycemic control on a day-to-day basis [2, 3]. SMBG serves as a vital adjunct to A1C as it can differentiate the fasting, preprandial, and postprandial hyperglycemic levels; detect the glycemic excursions; recognize and contribute in monitoring resolution of hypoglycemia; and provide immediate feedback to patients about the effects of food choices, activity, and medication on glycemic control [4].

In diabetes patients on intensive insulin regimens, SMBG is suggested at premeals, during snack, and bedtime, occasionally after eating, preexercise, when suspicion of and after correction of hypoglycemia, and before tasks like driving [5, 6]. There is also a positive relationship between frequency of SMBG and improvements in A1C and early detection of lower glucose values prior to symptomatic hypoglycemia may allow correction with a reduced risk for hyperglycemia [7]. Use of SMBG during exercise may also allow improved insulin management and reduce risk for hypoglycemia during and following exercise [8]. Measurement of blood glucose at different times in a day helps in management of improving blood glucose profiles, confirming hypoglycemia, monitoring recovery, and preventing hyperglycemic crises [3].

A recent observation study of diabetes self-management conducted by *Schnell et al*. revealed that SMBG implementation leads to an improvement in metabolic control, along with a significant improvement in diabetes management [9]. SMBG coupled with training in self-titration of insulin doses has resulted not only in better compliance and glycemic control but also increased the chances of achieving tight A1C target.

In patient who have stable oral regimen with A1C within the target range, SMBG data can be used as biofeedback at times of increased stress or changes in diet or physical activity. While patients whose diabetes is not in control or have initiated medication, SMBG data can be helpful in modifying or creating the diabetes management regimen [10].

SMBG has been well studied among individuals with diabetes and has proved to be a useful tool in improving glycemic control effectively and recognizing low blood glucose levels before the development of severe hypoglycemia [5]. Landmark clinical trials of insulin-treated patients have included SMBG as part of the multifactorial interventions to demonstrate the benefit of intensive glycemic control on diabetes complications [11, 12]. Clinical evidence supports a correlation between greater SMBG frequency and lower A1C. Moreover, patient’s specific needs and goals should not only determine but in fact should dictate SMBG frequency and timing [13].

Despite being recommended in various guidelines [13, 14], a large gap between recommended to current practices of SMBG is observed in both developed and developing countries. In India, this strategy is still not properly understood or implemented. Lunch and dinner are our major meals of the day and glycemic variations are not recorded during routine tests which are invariably done in the morning. Availability of meals with varying glycemic indices and affordability of glucose meters and strips are major factors that play a dominant role either in recommending or practicing SMBG. Fortunately, the advent of less expensive meters and a reduction in the cost of the strips has considerably brightened the scenario. Apart from this, SMBG use is also associated with improved cost-effectiveness [15]. In this consensus, we have evaluated various literature and recommendations on SMBG available online and drawn statement to fit Indian scenario.

#### **Considerations**

The decision including SMBG in clinical practice was based on the factors such as availability of and access to glucose meters and foods with varying glycemic indices that were reviewed in Indian context.

#### **Rationale**

##### *The aims of SMBG should include the following* [16, 17]:

- Accurately assess level of metabolic control by individual therapy
- Achievement of realistic targets
- Prevention of both acute and chronic complications of diabetes
- Reduce the effect of extreme glycemic excursions on cognitive function
- Assure proper data collection in various diabetes centers in order to provide an opportunity of comparison
- Enhance and enable improvement in interdisciplinary care for patients with diabetes
- Benefits can be achieved by maintaining proper record either in a form of a diary or electronic record keeping
- Record keeping should incorporate blood glucose readings, insulin dosage, record of special circumstances like illness, eating out, exercise, any episode of hypoglycemia and its severity, and any episode of ketonuria or ketonemia
- SMBG requires an easy procedure for patients to regularly monitor the performance and accuracy of their glucose meter

#### **Optimization**

SMBG accuracy is dependent on the instrument and user. Therefore, it is important to evaluate individual patient’s monitoring technique, both initially and at regular intervals thereafter. Optimal use of SMBG requires proper review and interpretation of the data, both by the patient and healthcare provider [18]. Among patients who check their blood glucose at least once daily, many report taking no action when results are high or low. Patients should be taught how to use SMBG data to adjust food intake, exercise, or pharmacological therapy to achieve specific goals. Ongoing need for and frequency of SMBG should be reevaluated at each routine visit. SMBG is especially important for insulin-treated patients to monitor and prevent asymptomatic hypoglycemia and hyperglycemia [19]. In a prospective Japanese study, *Nishimura et al*. have found that structured blood glucose testing comprising of seven-point SMBG for three consecutive days once every 2 months is beneficial for glycemic control rather than routine testing involving SMBG measurements three times each week before breakfast or dinner [20]. However, daily self-management was better with routine testing. The authors conclude that these two regimens can be individualized when SMBG is used less frequently.

#### **Evidence from India**

- In a study by *Shaji et al*. that assessed knowledge, attitude, and practice of T2DM patients towards self-monitoring and the impact of SMBG on glycemic control, patients who monitored ≥ 3 times had significantly better glycemic control of A1C (7.1–8%) than those who monitored < 3 times (*p* = 0.021) [21].
- Selecting a structured, flexible SMBG pattern that can be tailored to the clinical, educational, behavioral, and financial requirements of individuals with diabetes is recommended. As it is important to determine the frequency and intensity of SMBG needed to support the chosen treatment regimen, one should also consider practical obstacles to monitoring, such as affordability or access and individualize glycemic target and modify monitoring patterns accordingly [16, 22].
- Insulin self-titration interventions based on structured SMBG are associated with significant reduction in A1C during a follow-up of 12 weeks with a trend towards greater effectiveness in improving glycemic control than conventional treatment, with no increase in incidence of hypoglycemia or body weight gain [23].

#### **Implementation**

It is essential to establish whether glucometers report values for plasma or blood and to ensure that schemes for monitoring the quality of their output are in place. Blood glucose meters should be calibrated on a regular basis. Low-cost glucose strips and meters should be developed and made available for wider implementation of SMBG. Strategies that can lower PPG excursions in people with postprandial hyperglycemia should be implemented.

**References**

1. Khadilkar KS, Tushar Bandgar, Vyankatesh Shivane, Anurag Lila, and Nalini Shah. Current concepts in blood glucose monitoring. Indian J Endocrinol Metab. 2013; 17(Suppl 3): S643–S649.
2. Schnell O, Hanefeld M, Monnier L. Self-monitoring of blood glucose: a prerequisite for diabetes management in outcome trials. Journal of Diabetes Science and Technology. 2014;8(3):609–614.
3. Kirk JK, Stegner J. Self-monitoring of blood glucose: practical aspects. journal of diabetes science and technology. 2010;4(2):435–439.
4. Mansouri DA, Alawi HH, Barasyn KB, Bnnounh MN, Haddad NT, Al-Hafdey DA, Khayat EZ. Self-monitoring of blood glucose among diabetic patients attending Al-Eskan Primary Health Care Center in Makkah Al-Mukarramah city. International Journal of Medical Science and Public Health. 2015;4(4):527–37.
5. American Diabetes Association. Standards of medical care in diabetes 2017. Diabetes Care 2017:40, Supplement 1, S11-S135.
6. Wright LA-C, Hirsch IB. Metrics beyond hemoglobin A1C in diabetes management: time in range, hypoglycemia, and other parameters. Diabetes Technology & Therapeutics. 2017;19(Suppl 2):S-16-S-26.
7. Miller KM, Beck RW, Bergenstal RM et al. Evidence of a strong association between frequency of self-monitoring of blood glucose and hemoglobin A1c levels in T1D exchange clinic registry participants. Diabetes Care. 2013; 36(7):2009–14.
8. Zaharieva D, Yavelberg L, Jamnik V, Cinar A, Turksoy K, Riddell MC. The effects of basal insulin suspension at the start of exercise on blood glucose levels during continuous versus circuit-based exercise in individuals with type 1 diabetes on continuous subcutaneous insulin infusion. Diabetes Technology & Therapeutics. 2017;19(6):370–8.
9. Schnell O, Klausmann G, Gutschek B, Garcia-Verdugo RM, Hummel M. Impact on diabetes self-management and glycemic control of a new color-based SMBG meter. J Diabetes Sci Technol. 2017:1932296817706376.
10. Basu P. Biochemistry laboratory manual. Kolkata: Academic Publishers; 2016
11. The Diabetes Control and Complications Trial Research Group. The effect of intensive treatment of diabetes on the development and progression of long‑term complications in insulin-dependent diabetes mellitus. N Engl J Med 1993;329(14):977‑86.
12. United Kingdom Prospective Diabetes study (UKPDS) group. Intensive blood glucose control with sulphonylureas or insulin compared with conventional treatment and risk of complications in type 2 diabetes (UKPDS33). Lancet 1998;352(9131):837‑53.
13. Glycemic Targets. Position statement, American Diabetes Association. Diabetes Care 2015;38(Suppl 1):S33–S40.
14. Handelsman Y, Bloomgarden ZT, Grunberger G, Umpierrez G, Zimmerman RS, Bailey TS et al. American Association of Clinical Endocrinologists and American College of Endocrinology—Clinical practice guidelines for developing a diabetes mellitus comprehensive care plan—2015. Endocr Pract. 2015;21 Suppl 1:1–87.
15. Cameron C, Coyle D, Ur E, Klarenbach S. Cost-effectiveness of self-monitoring of blood glucose in patients with type 2 diabetes mellitus managed without insulin. Canadian Medical Association Journal. 2010;182(1):28–34.
16. Chowdhury S, Ji L, Suwanwalaikorn S, Yu NC, Tan EK. Practical approaches for self-monitoring of blood glucose: an Asia-Pacific perspective. Curr Med Res Opin. 2015;31(3):461–76.
17. Kesavadev J, Sadikot S, Wangnoo S, Kannampilly J, Saboo B, Aravind SR et al. Consensus guidelines for glycemic monitoring in type 1/type 2 & GDM. Diabetes Metab Syndr. 2014;8(3):187–95.
18. Boutati EI, Raptis SA. Self-monitoring of blood glucose as part of the integral care of type 2 diabetes. Diabetes Care 2009;32 (Suppl. 2):S205–10.
19. Kolb H, Kempf K, Martin S, Stumvoll M, Landgraf R. On what evidence-base do we recommend self-monitoring of blood glucose? Diabetes Res Clin Pract 2010;87(2):150–6.
20. Nishimura A, Harashima SI, Fujita Y, Tanaka D, Wang Y, Liu Y, Inagaki N. Effects of structured testing versus routine testing of blood glucose in diabetes self-management: a randomized controlled trial. Journal of Diabetes and its Complications. 2017;31(1):228–33.
21. Shaji S, Rajendran D, Kumpatla S, Viswanathan V. Evaluation of diabetes self-care with self-monitoring of blood glucose among type 2 diabetic patients and its impact on HbA1c. Int J Diabetes Dev Ctries. 2013;33(3):181–2.
22. Unnikrishanan R, Mohan V. Suggested protocols for self-monitoring of blood glucose in India. API textbook, 2013, Chapter 42. Available at: http://www.apiindia.org/medicine_update_2013/chap42.pdf. (Last accessed on 27 Aug 2015
23. Silva DD, Bosco AA. An educational program for insulin self-adjustment associated with structured self-monitoring of blood glucose significantly improves glycemic control in patients with type 2 diabetes mellitus after 12 weeks: a randomized, controlled pilot study. Diabetol Metab Syndr. 2015;7:2.

## **Chronic complications**

### **RSSDI 2017 recommendations for diabetic retinopathy (DR)**

#### **Recommended care**

- Documentation of formal history of vision and visual acuity either by recording it on sheet or electronic medical record (EMR) should be made mandatory.
- Ensure that examination of the eyes of people with T2DM is performed around the time of diagnosis and then routinely every 1–2 years as part of a formal recall process:
  - Measure and document visual acuity, corrected with glasses or pinhole.
  - Assess retinopathy:
    - Using retinal photography through dilated pupils, performed by an appropriately trained healthcare professional or
    - Through examination by an ophthalmologist
- Discuss the reasons for eye examination with the person with diabetes.
- Counsel women who are planning pregnancy on the risk of progression of retinopathy during pregnancy, especially if there is preexisting retinopathy. Ensure regular follow-up throughout pregnancy and up to 1 year postpartum.
- Use tropicamide to dilate pupils, unless contraindicated, after discussing the implications and obtaining agreement of the person with diabetes.
- Classify the findings of eye examination as required: routine review, earlier review, or referral to an ophthalmologist (if not making the examination).
- The following frequency of screening is suggested:
  - 1–2 years, if no retinopathy, depending on clinical situation
  - 12 months, if minimal unchanged retinopathy
  - 2–4 months, after any active ophthalmic intervention
  - 3–6 months if worsening since last examination
  - More often during pregnancy
- The following situations require specialist referral:
  - The same day:
    - Sudden loss of vision
    - Evidence of retinal detachment
  - Within 1 week:
    - Evidence of preretinal and/or vitreous hemorrhage
    - New vessel formation or rubeosisiridis
    - Inability to see or assess disc or fovea
  - Within 1–2 months:
    - Advanced retinal lesions (4:2:1 rule):
      - ▪Microaneurysms or retinal hemorrhages in four quadrants
      - ▪Venous beading in two quadrants
      - ▪IRMAs in one quadrant
    - Unexplained deterioration of visual acuity
    - Macular edema
    - Unexplained retinal findings
    - Cataract
    - Inability to visualize fundus
- Advise that good control of blood glucose, BP, and blood lipids can help to reduce the risk of development or worsening of eye complications.
- Advise that DR is not a contraindication for use of aspirin, if this is indicated for prevention of CVD.
- Advise that tests of intra-ocular pressure should be made periodically.
- Explain guarded prognosis about regaining vision after *intra-ocular lens* (IOL) surgery in mature/hypermature cataract because of poor assessment of retina in the presence of mature cataract

#### **Limited care**

- Use direct fundoscopy through dilated pupils, performed by a member of the healthcare team who is properly trained and has appropriate experience to assess retinopathy.
- Check visual acuity.
- Repeat review, referral, and preventative therapy are as for recommended care.
- Less frequent examinations (every 2 years) may be considered following one or more normal eye examinations

#### **Preamble**

Diabetic retinopathy (DR) is a microvascular complication of diabetes and one of the leading causes of blindness or vision impairment in India [1, 2]. Visual loss from DR could be due to diabetic macular edema (DME) or proliferative diabetic retinopathy (PDR). Global data suggests that the overall prevalence as 35.4% for any DR and around 7.5% for PDR [3]. Factors such as longer duration of diabetes and poorer glycemic and BP control were found to be strongly associated with DR [3, 4]. In a cross-sectional study carried out by All India Ophthalmological Society, prevalence of DR was 21.7% and the rate was high in males (*p* = 0.007), in patients with diabetes duration > 5 years (*p* = 0.001), in patients with age > 40 years (*p* = 0.01), in insulin users (*p* = 0.001), and in patients with history of vascular accidents (*p* = 0.0014) [2]. Furthermore, in the cross-sectional survey of Indian patients with T2DM in chronic complications in newly diagnosed patients with type 2 diabetes mellitus in India (CINDI) and cardiovascular risk factors, micro- and macrovascular complications at diagnosis in patients with young onset type 2 diabetes in India (CINDI 2) studies DR was prevalent in 6.1 and 5.1% patients, respectively [5, 6]. Moreover, the socioeconomic burden resulting from DR induced visual impairment or blindness, particularly in the working age group, is a serious concern [7]. Therefore, it is high time to devise the means of managing DR and bring the problem under control. Early diagnosis, optimization of risk factors, and timely photocoagulation at appropriate places could entirely prevent the blindness from diabetes [8]. Moreover, a systematic approach to health education and creating awareness among patients and various health personnel and matching it with appropriate screening and service delivery mechanisms will go a long way in preventing blindness due to DR. Furthermore, early detection and management of DR with quick referrals and a highly coordinated teamwork between the endocrinologists and the ophthalmologists could reduce the prevalence of DR in India [9]. In addition, these recommendations will provide insights on the management of DR while promoting awareness and thus preventing vision impairment due to DR through cost-effective interventions.

#### **Considerations**

The recommendations on management of DR were taken from IDF 2014. However, few of the IDF recommendations were modified based on the local factors such as limited resources, high prevalence of DR, lack of quality assurance in labs, and availability of newer technologies and therapies for eye screening and treatment which were reviewed in Indian context.

#### **Rationale/evidence**

##### *Screening*

- Several guidelines emphasize on eye screening in T2DM; however, it appears they are divided on the frequency of screening. Some recommend annual screening (NICE-UK) while others recommend screening every 1–2 years (Canadian-Canada, Australian-Australia, and SIGN-Scotland).
- With regard to frequency of screening in limited care setting, the panel endorsed the ADA recommendation which suggests less frequent examinations (every 2–3 years) following one or more normal eye examinations [10].
- Screening methods for DR include direct and indirect ophthalmoscopy, slit-lamp biomicroscopy, stereoscopic color film fundus photography, mydriatic or non-mydriatic digital color, and monochromatic photography [11, 12].

##### *Counselling pregnant women*

- DR is the foremost cause of blindness in women during their motherhood time, and pregnancy increases the short-term risk of DR progression [11]. The possible relationship between DR and the perinatal outcome has been addressed in several studies [13, 14]. Women with high severity of DR were more likely to develop obstetric complications [13, 15] and those with proliferative changes accounted for higher incidence of congenital malformations and/or fetal death [13].
- As pregnancy can induce progression of DR, the panel recommended preconception counselling for women, clearly explaining about the risk of progression of DR during pregnancy especially if they already have proliferative retinopathy. They should be advised on maintaining good glycemic control before and throughout pregnancy under the guidance of healthcare professional. In addition the panel emphasized on the need for close follow-up during pregnancy and up to 1 year postpartum and monitor for progression of DR and co-existing hypertension and renal disease, if any.

##### *Guarded prognosis after IOL surgery*

- Though surgical interventions are crucial for cataract management, most of the patients, particularly those with complicated cataracts, may not restore the vision. These patients eventually develop corneal decompensation, glaucoma, and optic atrophy [16]. Because the prognosis of retina is poor especially in the presence of mature cataract, the panel suggested that it is important to educate the patient about guarded prognosis for regaining vision after IOL surgery.

#### **Evidence**

Though evidence from past studies suggests that prevalence of DR is low in Indians compared to other ethnic groups, emerging data indicate significant increase in prevalence of retinopathy in South Asians compared to Caucasians [17]. Data from a population-based study (CURES) indicate that the overall prevalence of DR in urban South Indian population was 17.6%, with higher prevalence among men than in women (21.3 vs 14.6%; *p* < 0.0001) and among subjects with proteinuria (*p* = 0.002) [18]. Similarly, prevalence of DR in western India was found to be 33.9% [19]. Data from a recent population-based cross-sectional study suggests that one of 10 individuals in rural South India, above the age of 40 years, had evidence of DR [20]. A meta-analysis of seven studies from India found 14.9% of known diabetes patients aged ≥ 30 years and 18.1% among those aged ≥ 50 years had DR. Furthermore, no linear trend was observed between age and the proportion with DR [21]. Duration of diabetes, A1C, male gender, macroalbuminuria, and insulin therapy were found to be strongly associated with increased risk of DR among South Indians [22, 23]. Moreover, the risk of nephropathy (OR, 5.3; *p* < 0.0001) and neuropathy (OR, 2.9; *p* < 0.0001) was significantly higher among T2DM patients with DR compared to those without DR [24]. After adjusting for age, gender, A1C, SBP, serum triglycerides, and duration of diabetes, DR was significantly associated with nephropathy (*p* = 0.005) than with neuropathy [24].

#### **Implementation**

Sufficient number of trained general ophthalmologists and general physicians is required to develop an integrated DR model that facilitates early detection and create awareness on DR. Medical camps should be conducted for screening of diabetes and DR screening camps, which will help to identify people at risk of sight-threatening DR and initiate treatment including laser photocoagulation or vitreous surgery. Mobile vans with a fundus camera or other low-cost tools that can be used in remote rural areas should also be explored. However, successful implementation of program requires team approach, involving both administrative and voluntary organizations.

**References**

1. Patil S, Gogate P, Vora S, Ainapure S, Hingane RN, Kulkarni AN et al. Prevalence, causes of blindness, visual impairment and cataract surgical services in Sindhudurg district on the western coastal strip of India. Indian J Ophthalmol. 2014;62(2):240–5.
2. Gadkari SS, Maskati QB, Nayak BK. Prevalence of diabetic retinopathy in India: the all India ophthalmological society diabetic retinopathy eye screening study 2014. Indian Journal of Ophthalmology. 2016;64(1):38.
3. Solomon SD, Chew E, Duh EJ, Sobrin L, Sun JK, VanderBeek BL, Wykoff CC, Gardner TW. Diabetic retinopathy: a position statement by the American Diabetes Association. Diabetes Care. 2017;40(3):412–8.
4. Yau JW, Rogers SL, Kawasaki R, Lamoureux EL, Kowalski JW, Bek T, et al. Meta-Analysis for Eye Disease (META-EYE) Study Group. Global prevalence and major risk factors of diabetic retinopathy. Diabetes Care. 2012;35(3):556–64.
5. Sosale B, Sosale AR, Mohan AR, Kumar PM, Saboo B, Kandula S. Cardiovascular risk factors, micro and macrovascular complications at diagnosis in patients with young onset type 2 diabetes in India: CINDI 2. Indian Journal of Endocrinology and Metabolism. 2016;20(1):114.
6. Sosale A, Kumar KP, Sadikot SM, Nigam A, Bajaj S, Zargar AH, Singh SK. Chronic complications in newly diagnosed patients with type 2 diabetes mellitus in India. Indian Journal of Endocrinology and Metabolism. 2014;18(3):355.
7. Rani PK, Raman R, Agarwal S, Paul PG, Uthra S, Margabandhu G, et al. Diabetic retinopathy screening model for rural population: awareness and screening methodology. Rural Remote Health. 2005;5(4):350.
8. Chakrabarti R, Chatterjee T. Tip of the iceberg: the need for diabetic retinopathy screening in developing countries. Lessons from Vietnam. The Asia-Pacific Journal of Ophthalmology. 2013;2(2):76–8.
9. Unnikrishnan AG, Kalra S, Tandon N. Diabetic retinopathy care in India: an endocrinology perspective. Indian Journal of Endocrinology and Metabolism. 2016;20(Suppl 1):S1.
10. American Diabetes Association. Standards of medical care in diabetes—2015 abridged for primary care providers. Clin Diabetes. 2015;33(2):97–111.
11. Morrison JL, Hodgson LA, Lim LL, Al-Qureshi S. Diabetic retinopathy in pregnancy: a review. Clinical & Experimental Ophthalmology. 2016;44(4):321–34.
12. Garg S, Davis RM. Diabetic retinopathy screening update. Clinical diabetes. 2009;27(4):140–5.
13. Mallika PS, Tan AK, Aziz S, Asok T, Alwi SS, Intan G. Diabetic retinopathy and the effect of pregnancy. Malaysian Family Physician: the official journal of the Academy of Family Physicians of Malaysia. 2010;5(1):2.
14. Egan AM, McVicker L, Heerey A, Carmody L, Harney F, Dunne FP. Diabetic retinopathy in pregnancy: a population-based study of women with pregestational diabetes. Journal of Diabetes Research. 2015.
15. Lauszus F, Klebe JG, Bek T. Diabetic retinopathy in pregnancy during tight metabolic control. Acta Obstet Gynecol Scand. 2000;79(5):367–70.
16. Chen Z, Lin X, Qu B, Gao W, Zuo Y, Peng W, Jin L, Yu M, Lamoureux E. Preoperative expectations and postoperative outcomes of visual functioning among cataract patients in urban Southern China. PLOS One. 2017;12(1):e0169844.
17. Raymond NT, Varadhan L, Reynold DR, Bush K, Sankaranarayanan S, Bellary S, et al. Higher prevalence of retinopathy in diabetic patients of South Asian ethnicity compared with white Europeans in the community: a cross-sectional study. Diabetes Care. 2009;32(3):410–5.
18. Rema M, Premkumar S, Anitha B, Deepa R, Pradeepa R, Mohan V. Prevalence of diabetic retinopathy in urban India: the Chennai Urban Rural Epidemiology Study (CURES) eye study, I. Invest Ophthalmol Vis Sci. 2005;46(7):2328–33.
19. Ramavat PR, Ramavat MR, Ghugare BW, Vaishnav RG, Joshi MU. Prevalence of diabetic retinopathy in Western Indian type 2 diabetic population: a hospital-based cross-sectional study. J ClinDiagn Res. 2013;7(7):1387–90.
20. Raman R, Ganesan S, Pal SS, Kulothungan V, Sharma T. Prevalence and risk factors for diabetic retinopathy in rural India. SankaraNethralaya Diabetic Retinopathy Epidemiology and Molecular Genetic Study III (SN-DREAMS III), report no. 2. BMJ Open Diabetes Res Care. 2014;2(1):e000005.
21. Jotheeswaran AT, Lovakanth N, Nadiga S, Anchala R, Murthy GVS, Gilbert CE. Estimating the proportion of persons with diabetes developing diabetic retinopathy in India: a systematic review and meta-analysis. Indian Journal of Endocrinology and Metabolism. 2016;20(Suppl 1):S51-S58.
22. Pradeepa R, Anitha B, Mohan V, Ganesan A, Rema M. Risk factors for diabetic retinopathy in a South Indian Type 2 diabetic population—the Chennai Urban Rural Epidemiology Study (CURES) Eye Study 4. Diabet Med. 2008;25(5):536–42.
23. Shah A, Kanaya AM. Diabetes and associated complications in the South Asian population. Current Cardiology Reports. 2014;16(5):476.
24. Pradeepa R, Anjana RM, Unnikrishnan R, Ganesan A, Mohan V, Rema M. Risk factors for microvascular complications of diabetes among South Indian subjects with type 2 diabetes—the Chennai Urban Rural Epidemiology Study (CURES) Eye Study-5. Diabetes Technol Ther. 2010;12(10):755–61.

### **RSSDI 2017 recommendations for diabetic neuropathy (DN)**

#### **Recommended care**

- All patients with T2DM should be assessed for DN at the time of initial diagnosis and annually thereafter.
- Diagnose sensorimotor nerve damage by history and examination (10 g monofilament with or without temperature, non-traumatic pinprick, vibration [128 Htz tuning fork], ankle reflexes), and/or simple quantitative testing (e.g., biothesiometer vibration perception). Use serum B12, thyroid function tests, creatinine/urea, alcohol abuse, and medication history to exclude other causes.
- Diabetic Neuropathy Symptom Score (NSS) and Neuropathy Disability Score (NDS) in T2DM population have been found to be a useful resource in evaluating diabetic sensorimotor polyneuropathy as an important bedside tool.
- Diagnose symptomatic (painful) DN by excluding other possible causes of the symptoms. Manage by stabilizing blood glucose control, and treatment with tricyclic antidepressants, if simple analgesia is not successful. If a 1- month trial of tricyclic therapy is not successful, further treatment options include pregabalin/gabapentin and duloxetine, then tramadol and oxycodone. Further management normally requires referral to a pain control team. Be aware of the psychological impact of continuing symptoms, particularly if sleep is disturbed. In patients with DN and comorbid depression, anxiety, and sleep loss, duloxetine should be preferred.
- Diagnose erectile dysfunction by history (including medication history), exclusion of endocrine conditions (measure prolactin and testosterone), and a trial of a phosphodiesterase type-5 (PDE5) inhibitor (where not contraindicated by nitrate therapy). Consider other approaches such as intra-urethral or intracavernosal drugs and sexual and relationship counselling, where PDE5 inhibitors fail or cannot be used.
- Diagnose gastroparesis by history, trial of a prokinetic drug (metoclopramide, domperidone), and if troublesome by gastric emptying studies.
- Diagnose CV autonomic neuropathy by resting heart rate and heart rate response to provocation tests (lying-standing, Valsalva, deep breathing) and by lying and standing BP. Inform anesthetists when relevant, where this is present.

#### **Limited care**

- Screen and diagnose sensorimotor nerve damage by history of symptoms and sensory assessment by 10 g monofilament or tuning fork with/without non-traumatic disposable pinprick.
- NSS and NDS in T2DM population have been found to be a useful resource in evaluating diabetic sensorimotor polyneuropathy as an important bed side tool.
- Manage symptomatic (painful) diabetic neuropathy by excluding other causes, stabilizing glycemic control, and treatment with tricyclic antidepressants if simple analgesia is not successful. Opiate analgesia may be necessary as locally available.
- Assess erectile dysfunction by history and examination and consider possible contributions of other medication or disease.

#### **Preamble**

Neuropathies are the most common complication of diabetes, affecting up to 50% of patients with T2DM [1]. Metabolic disruptions in the peripheral nervous system (altered protein kinase C activity and increased polyol pathway activity) due to hyperglycemia play a key role in the development of DN [2, 3]. Approximately 30% patients either with known or newly diagnosed diabetes are suffering from DN [4, 5]. In the cross-sectional survey of Indian patients with T2DM in CINDI and CINDI 2 studies, DN was prevalent in 13.15 and 13.2% patients, respectively [6, 7]. The most common form of DN is the distal symmetrical polyneuropathy that involves both tibial and sural nerves [8]. Presence of neuropathy is associated with significant morbidity, including recurrent foot infection and ulcers, impotence in men with diabetes, and sudden death in individuals with CV autonomic neuropathy. Neuropathic pain in patients with diabetes is commonly encountered in clinical practice. Therefore, timely screening and early detection ensure prevention of the progression of DN [9]. The present recommendations provide insights on the management aspects of DN while exploring newer therapeutic options that have emerged in recent years.

#### **Considerations**

The panel endorsed the IDF 2014 recommendations for diagnosis and management of DN. However, few of the recommendations were modified based on local factors such as limited resources, which helps in clinical diagnosis of DN, lack of quality assurance in labs, and need for cost-effective diagnostic techniques which were reviewed in Indian context.

### **Rationale/evidence**

#### *Detection of sensorimotor polyneuropathy*

- Though nerve conduction studies are powerful tools for identifying cases of DN [10], NSS (Annexure X) and NDS (Annexure XI) in T2DM patients were found to be a useful resource for evaluating diabetic sensorimotor polyneuropathy as a bed side tool [11, 12]. A cross-sectional study in T2DM patients that examined the nerve conduction velocities of motor and sensory nerves using NSS and NDS in patients of clinically detectable neuropathy showed significant electrophysiological changes with duration of T2DM [11]. Similar results were observed in another study where NSS and NDS together helped in prompt evaluation of diabetic sensorimotor polyneuropathy and also in diagnosing subclinical cases [13–15]. A study that validated the use of NSS and NDS for clinical diagnosis of peripheral neuropathy in middle-aged 855 T2DM patients showed that NSS and NDS can detect DN with a sensitivity of 71.1% and specificity of 90% and was found to be simple, acceptable, reproducible, and validated method for early diagnosis of DN [12, 16].
- The panel emphasized on neurological examination using NSS and NDS as it is an important bed side tool and a useful resource in evaluating diabetic sensorimotor polyneuropathy.

#### *Management of diabetic neuropathy*

- Duloxetine and pregabalin were approved by the USFDA in 2004, and tapentadol extended release was approved in 2012 for the treatment of painful diabetic neuropathy (PDN) [17].
- Duloxetine is a selective inhibitor of reuptake of both 5-hydroxytryptamine and norepinephrine [18, 19]. Results from randomized controlled clinical trials reveal that duloxetine provides significantly more diabetic neuropathic pain relief than either placebo or routine care with higher degree of safety and tolerability [20–22]. Moreover, a recent Cochrane collaboration review including data from eight studies and 2728 participants reports that both 60- and 120-mg daily doses of duloxetine were efficacious, but lower doses were not associated with improvement in the PDN management [23].
- Pregabalin is a potent gabapentinoid used in the management of PDN. Several double-blind placebo-controlled trials have reported the dose-dependent (600 mg/daily) efficacy of pregabalin; however, a number of side effects including mood disturbance, ankle edema, and sedation also have been reported [24–26].
- Both duloxetine and pregabalin are effective; however, a significant better improvement in QoL of patients was obtained by duloxetine with comparatively mild increase in the price [27].
- Tapentadol, an opioid analgesic, may act via opioid spinal-supraspinal synergy, as well as intrinsic spinally mediated μ-opioid receptor agonist-norepinephrine reuptake inhibitor effect [28]. The efficacy and safety of tapentadol were also published in several clinical trials [29, 30].

#### **Implementation**

Appropriate protocols should be developed for sensory testing and may include formal assessment using the NSS and NDS. Recommended medications should be available according to the level of resources. Medical teams need to remain trained in the diverse manifestations of autonomic neuropathy.

**References**

1. World Health Organization. Diabetes mellitus fact sheet 138.
2. Singh R, Kishore L, Kaur N. Diabetic peripheral neuropathy: current perspective and future directions. Pharmacol Res. 2014;80:21–35.
3. Bayram EH, Sezer AD, Elçioğlu HK. Diabetic neuropathy and treatment strategy—new challenges and applications. InSmart Drug Delivery System 2016. InTech.
4. Rani PK1, Raman R, Rachapalli SR, Pal SS, Kulothungan V, Sharma T. Prevalence and risk factors for severity of diabetic neuropathy in type 2 diabetes mellitus. Indian J Med Sci. 2010;64(2):51–7.
5. Gill HK, Yadav SB, Ramesh V, Bhatia E. A prospective study of prevalence and association of peripheral neuropathy in Indian patients with newly diagnosed type 2 diabetes mellitus. Journal of Postgraduate Medicine. 2014;60(3):270.
6. Sosale B, Sosale AR, Mohan AR, Kumar PM, Saboo B, Kandula S. Cardiovascular risk factors, micro and macrovascular complications at diagnosis in patients with young onset type 2 diabetes in India: CINDI 2. Indian Journal of Endocrinology And Metabolism. 2016;20(1):114.
7. Sosale A, Kumar KP, Sadikot SM, Nigam A, Bajaj S, Zargar AH, Singh SK. Chronic complications in newly diagnosed patients with type 2 diabetes mellitus in India. Indian Journal of Endocrinology and Metabolism. 2014;18(3):355.
8. Kakrani AL, Gokhale VS, Vohra KV, Chaudhary N. Clinical and nerve conduction study correlation in patients of diabetic neuropathy. J Assoc Physicians India. 2014;62(1):24–7.
9. Bansal D, Gudala K, Muthyala H, Esam HP, Nayakallu R, Bhansali A. Prevalence and risk factors of development of peripheral diabetic neuropathy in type 2 diabetes mellitus in a tertiary care setting. J Diabetes Investig. 2014;5(6):714–21.
10. Kannan MA, Sarva S, Kandadai RM, Paturi VR, Jabeen SA, Borgohain R. Prevalence of neuropathy in patients with impaired glucose tolerance using various electrophysiological tests. Neurol India. 2014;62(6):656–61
11. Hussain G, Rizvi SA, Singhal S, Zubair M, Ahmad J. Cross sectional study to evaluate the effect of duration of type 2 diabetes mellitus on the nerve conduction velocity in diabetic peripheral neuropathy. Diabetes MetabSyndr. 2014;8(1):48–52.
12. Chawla A, Bhasin G, Chawla R. Validation of Neuropathy Symptoms Score (NSS) and Neuropathy Disability Score (NDS) in the clinical diagnosis of peripheral neuropathy in middle aged people with diabetes. The Internet Journal of Family Practice. 2013;12(1):1–4.
13. Asad A, Hameed MA, Khan UA, Butt MU, Ahmed N, Nadeem A. Comparison of nerve conduction studies with diabetic neuropathy symptom score and diabetic neuropathy examination score in type-2 diabetics for detection of sensorimotor polyneuropathy. J Pak Med Assoc. 2009;59(9):594–8.
14. Afifi L, Abdelalim AM, Ashour AS, Al-Athwari A. Correlation between clinical neuropathy scores and nerve conduction studies in patients with diabetic peripheral neuropathy. The Egyptian Journal of Neurology, Psychiatry and Neurosurgery. 2016;53(4):248.
15. Kamel SR, Hamdy M, Omar HA, Kamal A, Ali LH, Elkarim AH. Clinical diagnosis of distal diabetic polyneuropathy using neurological examination scores: correlation with nerve conduction studies. Egyptian Rheumatology and Rehabilitation. 2015;42(3):128.
16. Chawla A, Chawla R, Jaggi S. Microvasular and macrovascular complications in diabetes mellitus: distinct or continuum? Indian Journal of Endocrinology and metabolism. 2016;20(4):546.
17. Javed S, Petropoulos IN, Alam U, Malik RA. Treatment of painful diabetic neuropathy. Therapeutic advances in chronic disease. 2015;6(1):15–28.
18. Smith T, Nicholson RA. Review of duloxetine in the management of diabetic peripheral neuropathic pain. Vasc Health Risk Manag. 2007;3(6):833–44.
19. Ormseth MJ, Scholz BA, Boomershine CS. Duloxetine in the management of diabetic peripheral neuropathic pain. Patient Prefer Adherence. 2011;5:343–56.
20. Goldstein DJ, Lu Y, Detke MJ, Lee TC, Iyengar S. Duloxetine vs. placebo in patients with painful diabetic neuropathy. Pain. 2005;116(1–2):109–18.
21. Raskin J, Pritchett YL, Wang F, D’Souza DN, Waninger AL, Iyengar S, et al. A double-blind, randomized multicentre trial comparing duloxetine with placebo in the management of diabetic peripheral neuropathic pain. Pain Med. 2005;6(5):346–56.
22. Wernicke JF, Pritchett YL, D’Souza DN, Waninger A, Tran P, Iyengar S, et al. A randomized controlled trial of duloxetine in diabetic peripheral neuropathic pain. Neurology. 2006;67(8):1411–20.
23. Lunn M, Hughes RA, Wiffen PJ. Duloxetine for treating painful neuropathy, chronic pain or fibromyalgia. The Cochrane Library. 2014.
24. Richter RW, Portenoy R, Sharma U, Lamoreaux L, Bockbrader H, Knapp LE. Relief of painful diabetic peripheral neuropathy with pregabalin: a randomized, placebo-controlled trial. The Journal of Pain. 2005;6(4):253–60.
25. Arezzo JC, Rosenstock J, LaMoreaux L, Pauer L. Efficacy and safety of pregabalin 600 mg/d for treating painful diabetic peripheral neuropathy: a double-blind placebo-controlled trial. BMC Neurology. 2008;8(1):33.
26. Wang D, Bao JB, Zhang K, Ju LF, Yu LZ. Pregabalin for the treatment of neuropathic pain in adults: a systematic review of randomized controlled trials. International Journal of Clinical and Experimental Medicine. 2017;10(1):16–29.
27. Roy MK, Kuriakose AS, Varma SK, Jacob LA, Beegum NJ. A study on comparative efficacy and cost effectiveness of Pregabalin and Duloxetine used in diabetic neuropathic pain. Diabetes & Metabolic Syndrome: Clinical Research & Reviews. 2017;11(1):31–5.
28. Christoph T, Schröder W, Tallarida RJ, De Vry J, Tzschentke TM. Spinal-supraspinal and intrinsic μ-opioid receptor agonist-norepinephrine reuptake inhibitor (MOR-NRI) synergy of tapentadol in diabetic heat hyperalgesia in mice. Journal of Pharmacology and Experimental Therapeutics. 2013;347(3):794–801.
29. Schwartz S, Etropolski M, Shapiro DY, Okamoto A, Lange R, Haeussler J, Rauschkolb C. Safety and efficacy of tapentadol ER in patients with painful diabetic peripheral neuropathy: results of a randomized-withdrawal, placebo-controlled trial. Current Medical Research and Opinion. 2011;27(1):151–62.
30. Vinik AI, Shapiro DY, Rauschkolb C, Lange B, Karcher K, Pennett D, Etropolski MS. A randomized withdrawal, placebo-controlled study evaluating the efficacy and tolerability of tapentadol extended release in patients with chronic painful diabetic peripheral neuropathy. Diabetes Care. 2014;37(8):2302–9.

### **RSSDI 2017 recommendations for diabetic nephropathy**

#### **Recommended care**

- Kidney function should be assessed at diagnosis and annually by:
  - Urine test for albuminuria
  - Measurement of serum creatinine and calculation of eGFR
- Urinary albumin-to-creatinine ratio (ACR) measurement in an early morning first void (mid-stream) spot specimen is the preferred method for assessment of albuminuria/proteinuria. Where a first void specimen is not possible or practical, a random spot urine specimen is acceptable. ACR can be measured in the laboratory or at site-of-care.
- Control hyperglycemia and avoid exercise before testing for albuminuria.
- If ACR is raised (microalbuminuria), i.e., ACR > 25 mg/g in men and > 35 mg/g in women, repeat ACR twice over the following 4 months:
  - Microalbuminuria is confirmed if ACR is elevated in two out of three tests, in the absence of infection or overt proteinuria.
  - If both repeat tests are not raised, check again annually.
  - An ACR > 300 mg/g indicates macroalbuminuria.
- CKD is diagnosed on the basis of a raised urine albumin/protein or a reduced eGFR (< 60 mL/min/1.73 m^2^) calculated from the Modification of Diet in Renal Disease (MDRD) formula and using a standardized creatinine assay.
- Individuals with CKD should be managed as follows:
  - Use angiotensin converting enzyme (ACE) inhibitors or angiotensin receptor blockers (ARBs) in individuals with micro- or macroalbuminuria, titrated to maximum tolerated dose.
  - Intensify management of BP (target ≤ 130/80 mmHg) using BP-lowering medications and dietary modification (low salt intake).
  - Intensify management of blood glucose.
  - Monitor ACR, eGFR, and serum potassium.
  - Advice limiting protein intake to 1 g/kg daily if proteinuric, but many Indian patients may be taking only 0.6–0.8 g/kg/day. So protein restriction may be emphasized only to avoiding extra protein intake from non-vegetarian source.
  - Intensify other renal and CV protection measures.
  - Smoking leads to the progression of end stage renal disease (ESRD) in diabetes so patients must be counselled for quitting smoking.
- Agree referral criteria for specialist renal care between local diabetes specialists and nephrologists. Referral criteria might include eGFR < 30 mL/min/1.73 m^2^, progressive deterioration of kidney function, persistent proteinuria, biochemical or fluid retention problems, or difficult diagnosis (to rule out non-diabetic renal disease where fundus is normal and no proteinuria).

#### **Limited care**

- Check annually for proteinuria in an early morning urine sample (or a random sample) using a dipstick. If test is positive, exclude UTIs by microscopy (and culture if possible).
- Measure serum creatinine and calculate eGFR annually.
- A simple inexpensive screening procedure for urinary protein excretion which can be used as a diagnostic test in outpatient has been reported in Indian population. Estimated proteinuria (EPE) is useful in serial evaluation of kidney function.
- Manage those with proteinuria as follows:
  - If available, consider use of ACE inhibitors or ARBs taking into account cost.
  - Aim for BP ≤ 130/80 mmHg using any BP-lowering medication and control of salt intake.
  - Aim to achieve targets for blood glucose control.
  - Aim to improve lipid profile using available medications.
  - Check proteinuria status annually.
  - Measure serum creatinine and calculate eGFR annually.

**Table:** 

Stratifying target blood pressure as per clinical condition	
ADA 2017 [1]	•< 140/90 mmHg is recommended to decrease CVD mortality and slow down CKD progression • Lesser targets such as < 130/80 mmHg might be considered in individuals with albuminuria and at increased risk of CVD and CKD progression • While achieving < 130 mmHg systolic BP target, especially in old people, care should be taken to avoid diastolic BP levels < 60–70 mmHg
KDIGO 2012 [2]	• In CKD patients, not requiring dialysis, with urinary albumin excretion < 30 mg/day and office BP consistently below 140/90 mmHg, a target of ≤ 140/90 mmHg is recommended • In CKD patients, not requiring dialysis, with urinary albumin excretion > 30 mg/day and office BP consistently > 130/80 mmHg, a target of ≤ 130/80 mmHg is recommended

#### **Preamble**

Diabetic nephropathy is a leading cause of ESRD affecting ∼ 20–30% diabetes patients and is associated with increased CV mortality [3]. It affects 10–40% of T2DM patients who eventually suffer from kidney failure [4, 5]. In the cross-sectional survey of Indian patients with T2DM in CINDI and CINDI 2 studies, DN was prevalent in 1.06 and 0.9% patients, respectively [6, 7]. Cost of treatment for advanced CKD is substantial. Less than 10% of ESRD patients have access to any kind of renal replacement therapy [8, 9]. Thus, in a country with limited resources, it becomes appropriate to direct efforts towards prevention of CKD rather than the treatment. In India, with an increase in the prevalence of diabetes, it becomes imperative to evolve definite guidelines for detection of diabetic nephropathy and suggest practical clinical recommendations to combat it. Improving glycemic control, aggressive antihypertensive treatment, and the use of ACE inhibitors or ARBs will slow down the rate of progression of nephropathy [10, 11]. In addition, protein restriction and other treatment modalities such as phosphate lowering may have benefits in selected patients [12].

#### **Considerations**

The panel endorsed the IDF 2014 recommendations for diagnosis and management of diabetic nephropathy. However, few of the recommendations were modified based on local factors such as limited resources, lack of quality assurance in labs, higher prevalence of diabetic kidney disease, and hypertension and cost of treatment of kidney failure through dialysis or transplantation which were reviewed in Indian context.

#### **Rationale/evidence**

##### *Screening for urinary protein excretion*

- EPE is a method of estimating ACR in a random urine sample to assess renal function in patients with diabetes. EPE was found to be useful in serial evaluation of kidney function in Indian patients with diabetes [13, 14]. Moreover, EPE is a simple and inexpensive screening procedure for urinary protein excretion which can be used as a diagnostic test in outpatient wards, particularly in developing countries like India.
- As EPE is an inexpensive screening procedure to assess kidney function, the panel recommended it for use in Indian population who are at risk of diabetic nephropathy.
- Screening of microalbuminuria and estimation of glycated albumin can help in the clinical management of diabetic nephropathy [15]. Screening for albuminuria by measuring urine albumin concentration or estimating ACR is acceptable in Asian population [16]. However, evidence suggests that vigorous exercise even for short periods (15–20 min) leads to ACR above the microalbuminuria threshold even in healthy subjects [17, 18].
- On the basis of evidence, the panel suggested that physicians should ask about recent vigorous exercise and avoid measuring urine albumin excretion for at least 24 h in the presence of same.

##### *Management*

*Protein restriction*

- IDF recommends limiting protein intake to 1 g/kg body weight daily among individuals with CKD, if they are found proteinuric. Similarly, ADA recommends protein intake should be 0.8 g/kg/body weight/day in patients with CKD [2]. In the Indian context, the source of protein is mainly from vegetable and animal oils and daily protein consumption is about 0.6–0.8 g/kg body weight [19]. Furthermore, protein content in non-vegetarian diet was found to be higher when compared to the vegetarian diet [20]. In addition, evidence suggests that animal protein may aggravate the risk of diabetes [21]. Therefore, the panel emphasized on protein restriction and avoiding extra protein intake, particularly in non-vegetarians with nephropathy.
  - *Smoking*
- Smoking is associated with hyperglycemia, dyslipidemia, and decline in GFR which leads to the progression of ESRD in patients with diabetes [22, 23]. Smoking tends to induce albuminuria and abnormal renal function through formation of advanced glycated end products (AGES) which are responsible for advanced vascular permeability and kidney damage [24]. A recent systematic review reports that consumption of ≥ 15 packs of cigarette/year increases the risk of progression of CKD [25]. Moreover, data from a recent study in India suggests that compared to non-smokers the prevalence of microalbuminuria in smokers was fourfold higher [26].
- The panel opined that patients must be counselled against tobacco use and encouraged to quit smoking to reduce the risk of progression to ESRD.
  - *Referral to specialist*
- The panel endorsed IDF recommendation on referral criteria; however, it was suggested that because most of the patients at this stage of diabetic nephropathy require a specialist care which may not be available at primary care or single physician center. Hence, local diabetes specialists should refer the patient to specialist renal care center/nephrologist. Likewise, nephrologists should refer patients to specialist renal care if the patient presents with following condition:
  - eGFR < 30 mL/min/1.73 m^2^
  - Progressive deterioration of kidney function
  - Persistent proteinuria, biochemical or fluid retention problems or
  - Difficulty in diagnosis (to rule out non-diabetic renal disease where fundus is normal and proteinuria is not present)

#### **Indian evidence**

- Prevalence of microalbuminuria is strongly associated with age, DBP, A1C, FPG, and duration of diabetes [27, 28].
- A positive correlation between urine albumin excretion rate and eGFR < 60 mL/min/1.73 m^2^ was observed indicating that these two parameters provide a complimentary benefit in management of CKD [29].
- Vitamin D deficiency can have significant impact on albuminuria. Therefore, supplementation with calcitriol should be considered in these patients as it has been shown to provide beneficial effects on microalbuminuria [30].

#### **Implementation**

Management of CKD requires access to healthcare professional, laboratory for ACR and creatinine estimations, and availability of multiple blood pressure-lowering medications in particular renin-angiotensin system blockers.

**References**

1. American Diabetes Association. Standards of medical care in diabetes. 2017. Available at: http://professional.diabetes.org/sites/professional.diabetes.org/files/media/dc_40_s1_final.pdf
2. Kidney Disease: Improving Global Outcomes (KDIGO) Blood Pressure Work Group. KDIGO clinical practice guideline for the management of blood pressure in chronic kidney disease. Kidney Int Suppl. 2012;2:337–414.
3. Ahmad J. Management of diabetic nephropathy: recent progress and future perspective. Diabetes MetabSyndr. 2015.
4. Agarwal SK, Srivastava RK. Chronic kidney disease in India: challenges and solutions. Nephron Clin Pract. 2009;111:c197–203.
5. Prasannakumar M, Rajput R, Seshadri K, Talwalkar P, Agarwal P, Gokulnath G, Kotak B, Raza A, Vasnawala H, Teli C. An observational, cross-sectional study to assess the prevalence of chronic kidney disease in type 2 diabetes patients in India (START-India). Indian Journal of Endocrinology and Metabolism. 2015;19(4):520.
6. Sosale B, Sosale AR, Mohan AR, Kumar PM, Saboo B, Kandula S. Cardiovascular risk factors, micro and macrovascular complications at diagnosis in patients with young onset type 2 diabetes in India: CINDI 2. Indian Journal of Endocrinology and Metabolism. 2016;20(1):114.
7. Sosale A, Kumar KP, Sadikot SM, Nigam A, Bajaj S, Zargar AH, Singh SK. Chronic complications in newly diagnosed patients with type 2 diabetes mellitus in India. Indian Journal of Endocrinology and Metabolism. 2014;18(3):355.
8. Kher V. End-stage renal disease in developing countries. Kidney Int. 2002;62:350–62.
9. Wetmore JB, Collins AJ. Global challenges posed by the growth of end-stage renal disease. Renal Replacement Therapy. 2016;2(1):15.
10. Vivian E, Mannebach C. Therapeutic approaches to slowing the progression of diabetic nephropathy—is less best. Drugs in Context. 2013.
11. Lozano-Maneiro L, Puente-García A. Renin-angiotensin-aldosterone system blockade in diabetic nephropathy. Present evidences. Journal of Clinical Medicine. 2015;4(11):1908–37.
12. Sahay M, Sahay R, Baruah MP. Nutrition in chronic kidney disease. Journal of Medical Nutrition and Nutraceuticals. 2014; 3(1): 11–18.
13. Viswanathan V, Chamukuttan S, Kuniyil S, Ambady R. Evaluation of a simple, random urine test for prospective analysis of proteinuria in type 2 diabetes: a six year follow-up study. Diabetes Res ClinPract. 2000;49(2–3):143–7.
14. Kumar PS, Rao UR, Abhilash T, Reddy GS. Evaluation of random urine sample protein: creatinine ratio as an index of 24 h urine protein in patients with various renal disorders in tertiary care center. International Journal of Advances in Medicine. 2016;3(4):855–8.
15. Kondaveeti SB, D K, Mishra S, Kumar R A, Shaker IA. Evaluation of glycated albumin and microalbuminuria as early risk markers of nephropathy in type 2 diabetes mellitus. J ClinDiagn Res. 2013;7(7):1280–3.
16. Jafar TH, Chaturvedi N, Hatcher J, Levey AS. Use of albumin creatinine ratio and urine albumin concentration as a screening test for albuminuria in an Indo-Asian population. Nephrol Dial Transplant. 2007;22(8):2194–200.
17. Heathcote KL, Wilson MP, Quest DW, Wilson TW. Prevalence and duration of exercise induced albuminuria in healthy people. Clin Invest Med. 2009;32(4):E261–5.
18. Sadjadi SA, Jaipaul N. Correlation of random urine protein creatinine (P-C) ratio with 24-hour urine protein and P-C ratio, based on physical activity: a pilot study. Therapeutics and Clinical Risk Management. 2010;6(351):7.
19. Sahay BK. Dietary carbohydrate content in Indian diabetic patients. Medicine Update 2012;22:235–9. Available at: http://www.apiindia.org/pdf/medicine_update_2012/diabetology_02.pdf (Last accessed on 27 Aug 2015).
20. Viswanathan V, Snehalatha C, Varadharani MP, Nair BM, Jayaraman M, Ramachandran A. Prevalence of albuminuria among vegetarian and non-vegetarian south Indian diabetic patients. Indian J Nephrol 2002;12:73–6.
21. Beasley JM, Wylie-Rosett J. The role of dietary proteins among persons with diabetes. Current Atherosclerosis Reports. 2013;15(9):348.
22. Hallan SI, Orth SR. Smoking is a risk factor in the progression to kidney failure. Kidney International. 2011;80(5):516–23.
23. Orth SR. Cigarette smoking: an important renal risk factor—far beyond carcinogenesis. Tob Induc Dis. 2002;1(2):137–55.
24. Shahid SM, Mahboob T. Cigarette smoking: an environmental risk factor for progression of nephropathy in diabetes. Int J Diabetes Dev Ctries. 2007;27 (4);104–7.
25. Júnior E, Fernando U, Elihimas HC, Lemos VM, Leão MD, Sá MP, França EE, Lemos A, Valente LM, Markman Filho B. Smoking as risk factor for chronic kidney disease: systematic review. Jornal Brasileiro de Nefrologia. 2014;36(4):519–28.
26. Gupta RK, Gupta R, Maheshwari VD, Mawliya M. Impact of smoking on microalbuminuria and urinary albumin creatinine ratio in non-diabetic normotensive smokers. Indian J Nephrol. 2014;24(2):92–6.
27. Varghese A, Deepa R, Rema M, Mohan V. Prevalence of microalbuminuria in type 2 diabetes mellitus at a diabetes centre in southern India. Postgrad Med J. 2001;77(908):399–402.
28. Thakkar B, Arora K, Vekariya R, Lulania M, Agnihotri AS. Prevalence of microalbuminuria in newly diagnosed type 2 diabetes mellitus. Natl J Integr Res Med 2011; 2:22–25.
29. Saha TK, Bhattarai AM, Batra HS, Banerjee M, Misra P, Ambade V. Correlation of microalbuminuria with estimated GFR (eGFR) by Cockcroft-Gault and MDRD formula in type 2 diabetics and hypertensives. Indian J ClinBiochem. 2015;30(3):271–4.
30. Xu J, Xiong H, Chen P. The effects of calcitriol on albuminuria in patients with type 2 diabetes mellitus. Int J Diabetes Dev Ctries.2015.

### RSSDI 2017 recommendations for foot care

#### Recommended care

- Assess feet of patients with diabetes as part of an annual review for lesions which require active treatment and for risk factors for ulcer and amputation:
  - History of previous foot ulceration or amputation, symptoms of peripheral arterial disease (PAD), physical or visual difficulty in self-foot care
  - Foot deformity (hammer or clawed toes, bone prominences), visual evidence of neuropathy (dry skin, dilated veins) or incipient ischemia, callus, nail deformity or damage, footwear
  - Detection of neuropathy by 10 g Semmes Weinstein monofilament (or 128-Hz tuning fork), a biothesiometer (VPT) is an option for quantitative assessment (cut-off point for ulcer risk > 25 V), non-traumatic pinprick
  - Palpation of foot pulses (dorsalis pedis and posterior tibial). Doppler or ankle/brachial pressure (ABI) ratio (< 0.9 for occlusive vascular disease) may be used where pulses are diminished to quantify the abnormality
- Discuss the reasons for foot review with each person with diabetes as part of the foot care educational process.
- Must emphasize not to walk bare foot at all including visit to religious places.
- Timely screening and early detection of diabetic neuropathy may help in prevention of the progression to diabetic foot.
- Agree a foot care plan based on the findings of annual foot review with each person with diabetes. Assess and provide necessary foot care education according to individual need and risks of ulcer and amputation.
- Classify risk of ulcer or amputation according to findings of the foot assessment:
  - No added risk: no risk factors and no previous history of foot ulcer or amputation
  - At risk: one risk factor and no previous history of foot ulcer or amputation
  - High risk:
    - Two or more risk factors
    - Previous ulcer or amputation (very high risk)
- Manage according to risk classification level:
  - No added risk: provide foot care education
  - At risk: arrange regular review, approximately every 6 months, by foot care team
    - At each review:
      - Inspect both feet—ensure provision of local management as indicated
      - Evaluate footwear—provide appropriate advice
      - Enhance foot care education
  - High risk:
    - Arrange frequent review every 3–6 months by foot care team
    - At each review:
      - Inspect both feet—ensure provision of local management as indicated
      - Evaluate footwear—provide advice and specialist insoles and shoes if indicated
      - Consider need for vascular assessment or referral if indicated
      - Evaluate and ensure the appropriate provision of intensified foot care education
- People with foot ulceration or infection require the following management:
  - Pressure off loading
  - Refer to multidisciplinary foot care team within 24 h for:
    - Appropriate wound management, dressings, and debridement as indicated
    - Infections should be classified as mild (superficial with minimal cellulitis), moderate (deeper than skin or more extensive cellulitis), or severe (accompanied by systemic signs of sepsis). Consideration of systemic antibiotic therapy (often longer term) for extensive cellulitis or bone infection as indicated; generic penicillin, cephalosporins, macrolides, clindamycin, and/or metronidazole as indicated as first-line medications, with amino-quinolones, or co-amoxicillin as examples of second-line medications
    - Probing to bone, radiology and scans, magnetic resonance imaging, and biopsy where indicated for suspected osteomyelitis
    - Reduce weight bearing, relief of pressure (walking with crutches, rest) off loading, and optimal pressure distribution (casting if indicated)
    - Investigation and treatment (referral) for vascular insufficiency
    - Specialist footwear and orthotic care (e.g., insoles) and individualized discussion of prevention of recurrence, when ulcer has healed
    - Optimal blood glucose control
  - Amputation should not be considered unless:
    - A detailed vascular evaluation has been performed by the vascular team
    - Ischemic rest pain cannot be managed by analgesia or revascularization
    - A life-threatening foot infection cannot be treated by other measures
    - A non-healing ulcer is accompanied by a higher burden of disease that would result in amputation

#### **Limited care**

- Risk assessment and classification would be as for recommended care but with sensory assessment by 10-g monofilament or tuning fork, with or without non-traumatic disposable pinprick only, and peripheral circulation assessment by palpation of pedal pulses.
- NSS and NDS in T2DM population has been found to be a useful resource in evaluating diabetic sensorimotor polyneuropathy as important bedside tool.
- Classification of infection would be as for recommended care but antibiotic therapy would be with generic penicillin, quinolones, macrolides, and/or metronidazole, given intravenously for deep tissue infections, and adjusted by response or culture results.
- Vascular referral would be according to findings and local revascularization facilities.

#### **Preamble**

Diabetic foot problems are one of the most common reasons for hospitalization of patients with diabetes (about 30% of admissions) and consume about 20% of the total healthcare costs of the disease compared to all other diabetic complications [1, 2, 3]. In India, prevalence of foot ulcers in patients with diabetes in clinic population varies from 3 to 14% [4–6]. Peripheral neuropathy, Charcot arthropathy, foot ulcers, infections, and lower extremity amputations are the various lower limb complications seen in diabetes patients [6]. Strategies aimed at preventing foot ulcers are cost-effective and can even be cost-saving if increased education and efforts are focused on those patients with recognized risk factors for the development of foot problem. The management of diabetic foot disease may seem poorly defined by comparison with complications such as nephropathy, hyperlipidemia, and retinopathy, for which clear guidelines exist. A multidisciplinary team approach, particularly in specialized diabetic foot clinics, is very successful in avoiding and treating foot complications. Present guideline focuses on the various mechanisms of managing diabetic foot disease.

#### **Considerations**

The panel endorsed the IDF 2014 recommendations for diagnosis and management of diabetic foot complications. However, few of the recommendations were modified based on local factors such as limited resources and lack of quality assurance in labs, which were reviewed in Indian context.

#### **Rationale/evidence**

##### *Detection and timely screening*

- Vibration perception threshold (VPT) is considered as a gold standard for diagnosis of diabetic peripheral neuropathy. However, simple clinical scores such as NSS and diabetic neuropathy examination (DNE) scores were found to be simple and useful tools for the diagnosis of peripheral neuropathy in patients with diabetes [7, 8]. Moreover, a good correlation between VPT score with tuning fork, monofilament, and ankle reflex was found suggesting that simple bed side tests are useful in clinical practice, even in those subjects in whom foot care practices are not followed [9, 10].
- Using NSS and NDS in T2DM patients has been found to be a useful resource in evaluating diabetic sensorimotor polyneuropathy as an important bed side tool [11–13] (Annexure XII).

##### *Avoid walking bare foot*

- Sociocultural practices like bare foot walking indoors and other religious places, use of improper footwear, and lack of knowledge regarding foot care are significant contributors of diabetic foot complications in India [14–16]. Therefore, the panel emphasized on educating patients on problems associated with walking bare foot [17] and advice on the use of appropriate/therapeutic footwear, particularly those at high risk to prevent the development of foot deformities and ulceration [18].
- A questionnaire-based study evaluating the foot care knowledge and practices with foot complications in 300 Indian patients suggests that majority of these patients were not educated previously about foot care and walked indoors without foot wear. The study emphasized that poor knowledge of foot care and poor footwear practices are important risk factors for foot problems in diabetes and called for a joint effort from doctors and footwear industry and to educate patients about foot care and improve their choice and selection of footwear so as to reduce foot problems [19].

##### *Pressure offloading*

- Pressure modulation commonly referred to as “off-loading” is an important component in the management and treatment of diabetic foot ulcers. It involves mitigation of pressure at an area of high vertical or shear stress [20, 21]. Combining effective, easy to use offloading devices such as total contact casts and removable cast walkers ensures patient compliance, heal foot ulcers, and avert limb amputations [1, 21, 22].
- Mandakini offloading device [23, 24] and Samadhan offloading system [24, 25] were found to be most economical, easy to apply, and effective methods to redistribute the pressure in ulcerative areas.
- A recent systematic review and meta-analysis report that compared with standard dressing changes, negative pressure wound therapy had a higher rate of complete healing of ulcers (RR, 1.48; 95% CI, 1.24–1.76; *p* < 0.001), shorter healing time (MD, − 8.07; 95% CI, − 13.70 to − 2.45; *p* = 0.005), greater reduction in ulcer area (MD, 12.18; 95% CI, 8.50–15.86; *p* < 0.00001), greater reduction in ulcer depth (MD, 40.82; 95% CI, 35.97–45.67; *p* < 0.00001), fewer amputations (RR, 0.31; 95% CI, 0.15–0.62; *p* = 0.001), and no effect on the incidence of treatment-related adverse effects (RR, 1.12; 95% CI, 0.66–1.89; *p* = 0.68) [26].

#### **Implementation**

Availability of basic equipment, appropriate protocols, structured records, and recall systems needs to be supported by appropriate training for professionals providing screening and management services. Liaison needs to be established with orthoptists, footwear suppliers, and cast technicians.

**References**

1. Pendsey S, Epidemiological aspects of diabetic foot. Int. J Diab. Dev Countries 1994;14:37–38.
2. International consensus on the Diabetic Foot, by the International Working Group on the Diabetic Foot, 1999. Available at: http://iwgdf.org/ (Last accessed on 31 July 2015)
3. Ignatyeva VI, Severens JL, Ramos IC, Galstyan GR, Avxentyeva MV. Costs of hospital stay in specialized diabetic foot department in Russia. Value in Health Regional Issues. 2015; 7:80–6.
4. Sinharay K, Paul UK, Bhattacharyya AK, Pal SK. Prevalence of diabetic foot ulcers in newly diagnosed diabetes mellitus patients. J Indian Med Assoc. 2012;110 (9):608–11.
5. Shahi SK, Kumar A, Kumar S, Singh SK, Gupta SK, Singh TB. Prevalence of diabetic foot ulcer and associated risk factors in diabetic patients from North India. The Journal of Diabetic Foot Complications 2012;4(3):83–91.
6. Jyothylekshmy V, Menon AS, Abraham S. Epidemiology of diabetic foot complications in a podiatry clinic of a tertiary hospital in South India. Indian Journal of Health Sciences. 2015;8(1):48.
7. Afifi L, Abdelalim AM, Ashour AS, Al-Athwari A. Correlation between clinical neuropathy scores and nerve conduction studies in patients with diabetic peripheral neuropathy. The Egyptian Journal of Neurology, Psychiatry and Neurosurgery. 2016;53(4):248.
8. Kamel SR, Hamdy M, Omar HA, Kamal A, Ali LH, Elkarim AH. Clinical diagnosis of distal diabetic polyneuropathy using neurological examination scores: correlation with nerve conduction studies. Egyptian Rheumatology and Rehabilitation. 2015;42(3):128.
9. Jayaprakash P, Bhansali A, Bhansali S, Dutta P, Anantharaman R, Shanmugasundar G, et al. Validation of bedside methods in evaluation of diabetic peripheral neuropathy. Indian J Med Res. 2011;133:645–9.
10. Mittal J, Khurana A, Mahajan DS, Dhoat PS. A comparative study of various bedside methods in detection of diabetic polyneuropathy in type 2 diabetes patients. JEMDS. 2013;16:9702–06.
11. Hussain G, Rizvi SA, Singhal S, Zubair M, Ahmad J. Cross sectional study to evaluate the effect of duration of type 2 diabetes mellitus on the nerve conduction velocity in diabetic peripheral neuropathy. Diabetes MetabSyndr. 2014;8(1):48–52.
12. Chawla A, Bhasin G, Chawla R. Validation of Neuropathy Symptoms Score (NSS) and Neuropathy Disability Score (NDS) in the clinical diagnosis of peripheral neuropathy in middle aged people with diabetes. The Internet Journal of Family Practice. 2013;12(1):1–4.
13. Dixit S, Maiya A. Diabetic peripheral neuropathy and its evaluation in a clinical scenario: a review. Journal of Postgraduate Medicine. 2014;60(1):33.
14. Abbas ZG, Viswanathan V. The diabetic foot in Africa and India. Int Diab Monit. 2007;19:8–12.
15. Vijay V, Snehalatha C, Ramachandran A. Sociocultural practices that may affect the development of the diabetic foot. IDF Bulletin. 1997;42:10–2.
16. Viswanathan V, Thomas N, Tandon N, Asirvatham A, Rajasekar S, Ramachandran A et al. Profile of diabetic foot complications and its associated complications—a multicentric study from India. J Assoc Physicians India. 2005;53:933-6a.
17. Viswanathan V, Madhavan S, Rajasekar S, Chamukuttan S, Ambady R. Amputation prevention initiative in South India: positive impact of foot care education. Diabetes Care. 2005;28(5):1019-21b.
18. Viswanathan V, Madhavan S, Gnanasundaram S, Gopalakrishna G, Das BN, Rajasekar S et al. Effectiveness of different types of footwear insoles for the diabetic neuropathic foot: a follow-up study. Diabetes Care. 2004;27(2):474–7.
19. Chandalia HB, Singh D, Kapoor V, Chandalia SH, Lamba PS. Footwear and foot care knowledge as risk factors for foot problems in Indian diabetics. Int J Diabetes Dev Ctries. 2008; 28(4): 109–13.
20. Armstrong DG, Lavery LA, Bushman TR. Peak foot pressures influence the healing time of diabetic foot ulcers treated with total contact casts. J Rehabil Res Dev. 1998;35(1):1–5.
21. Yazdanpanah L, Nasiri M, Adarvishi S. Literature review on the management of diabetic foot ulcer. World Journal of Diabetes. 2015;6(1):37.
22. Wu SC, Crews RT, Armstrong DG. The pivotal role of offloading in the management of neuropathic foot ulceration. Curr Diab Rep. 2005;5(6):423–9.
23. Kari SV. The economical way to off-load diabetic foot ulcers [Mandakini off-loading device]. Indian J Surg. 2010;72(2):133–4.
24. Agrawal VP. Easy ways to offload diabetic foot ulcer in rural setup. International Journal of Biomedical and Advance Research. 2014;5(4):187–9.
25. Shankhdhar K. Improvisation is the key to success: the Samadhan System. Adv Skin Wound Care 2006;19(7):379–83.
26. Liu S, He CZ, Cai YT, Xing QP, Guo YZ, Chen ZL, Su JL, Yang LP. Evaluation of negative-pressure wound therapy for patients with diabetic foot ulcers: systematic review and meta-analysis. Therapeutics and clinical risk management. 2017;13:533.

## **Infections and vaccinations**

### **RSSDI 2017 recommendations**

#### **Recommended care**

- All adult diabetes subjects should be educated about administering pneumococcal and influenza vaccine and those age of > 60 years should be advised to be vaccinated.
- For diabetes subjects, a single dose of pneumococcal conjugate vaccine (PCV13) to start and a second dose (in immunocompromised or > 65 years old) after 1 year and a booster dose after 5 years with pneumococcal polysaccharide vaccine (PPSV23) is recommended.
- Children with diabetes < 2 years of age can be given pneumococcal polysaccharide vaccine and children > 6 months of age can be provided with influenza vaccine.
- Other vaccines may be administered in patients with diabetes based on need.
- Irrespective of age, immunization is recommended in all patients with:
  - Renal failure
  - Diabetes and immune-compromised state due to concomitant conditions
  - Diabetes and chronic lung diseases like chronic obstructive pulmonary disease (COPD) and bronchial asthma
  - Diabetes patients who smoke
  - Poor hygienic conditions (especially slum dwellers) and those who frequently travel to high-risk areas
- Vaccination is contraindicated/postponed in patients with:
  - Hypersensitivity to the active substances or to any of the excipients of the vaccine
  - History of chicken egg allergy particularly when considering flu shot
  - Recent history of Guillain-Barre syndrome within 6 weeks of a previous influenza vaccination in the case of flu shot
  - Postponed in patients with febrile illness or any acute infection
- In patients with chicken egg allergy, chemoprophylaxis with amantadine/rimantadine or immunization using a protocol as reported by Murphy and Strunk may be considered.

#### **Limited care**

- The principles for infections and vaccinations during diabetes are as for recommended care subject to availability and affordability of pneumococcal and influenza vaccines.

#### **Preamble**

The risk of developing infectious diseases due to diabetes is now being considered an important complication of diabetes [1, 2]. Diabetes increases the risk of infection by two to three times in comparison to the non-diabetic population. The morbidity and mortality associated with infectious diseases such as influenza, pneumonia, and hepatitis, which is usually preventable by appropriate vaccination, also appear to be very high in diabetes subjects [3]. Patients with T2DM, especially those with PVD, are at high risk for many types of typical and atypical infections due to immune dysfunction, DN, and poor circulation [4]. Furthermore, skin breakdown in patients with advanced diabetes and PVD provides a portal of entry for bacteria. Longer duration of diabetes and poor glycemic control causes increased risk of pneumonia related hospitalizations in diabetes subjects due to compromised immune system of the host [5]. A recent study demonstrated that patients with high blood glucose level are at increased risk of community-acquired pneumonia [6, 7]. Even certain viral infections can lead to new onset of diabetes in the population who are genetically prone to develop diabetes.

#### **Considerations**

The decision about conducting a screening program should be based on local factors such as limited resources and high prevalence of diabetes-related infections factors that were reviewed in Indian context.

#### **Rationale and evidence**

##### *Infections in diabetes*

- Several factors have been implicated for infections in diabetes, of which, altered immunity is the most predominant one [4, 8]. Other predisposing factors increasing susceptibility to infections include diabetes-related complications, frequent catheterization, and dialysis in chronic renal failure patients. Evidence that these immunological defects can be corrected through good glycemic control support the importance of close monitoring of infectious diseases in subjects with diabetes [9].
- Urinary tract, respiratory tract, and foot and deep soft infections are most common in T2DM occurring with increased incidence and resulting in high mortality [10, 11].
- Following section deals with evidences from Indian and global studies on infections that commonly occur in patients with diabetes.
  - *Influenza*: Diabetes increases the risk of hospitalization after influenza infection and quadruples the risk of intensive care unit (ICU) admission after hospitalization [12]. Death rates among patients with diabetes during influenza epidemics may increase up to 5–15% [13]. Evidence that influenza can trigger coronary complications, when taken in the context of diabetes subjects, gains more significance since the risk for CVD is already two- to fourfold higher in this subgroup [11, 14].
  - *Retroviral infections*: Cirrhosis of liver in diabetes patients results in higher incidence of glucose intolerance (60–96%) and overt diabetes (20–60%) [15]. Elevated rates of inflammation and endothelial cell dysfunction are observed in human immunodeficiency virus (HIV)-infected patients with T2DM [16]. Moreover, in HIV patients undergoing active retroviral therapy, autoimmune diabetes may be caused due to protein inhibitors and nucleoside analogues. Therefore, in HIV patients with compensated cirrhosis and high IR, insulin should be the preferred choice of treatment [15].
  - *Malignant otitis externa*: This commonly occurs in patients with diabetes and is mostly caused by *Pseudomonas aeruginosa* [17]. It can be prevented by creating proper awareness regarding healthy ear cleaning practices like not using commercially available ear buds and other foreign objects or unsterilized cotton. Management protocol comprises of strict glycemic control, correction of electrolyte imbalance, improvement in immuno-competence, aural toileting, hyperbaric oxygen therapy and prolonged systemic, and ototopic antimicrobial therapy (3–6 weeks) with agents such as piperacillin with tazobactam, ciprofloxacin, and cefoperazone [17].
  - *Infections of hand and upper limb*: Diabetes ulcers in the upper limb should be promptly treated with adequate surgical means in order to prevent spreading of infection. Creating awareness on healthy cleaning practices minimizes disability and result in better outcome [18].
  - *UTIs*: These, mostly asymptomatic bacterial infections, occur more frequently in female diabetes patients. In all hospitalized diabetes patients, it is recommended to perform urine culture to detect presence of bacteriuria, a condition leading to an unexplained worsening of the glycemic control in some patients [19].
  - *Hepatitis*: It has been observed that several patients with underlying diabetes suffer from prolonged or complicated course of acute viral hepatitis. It is possible that with impaired hepatocyte regenerating capacity, these patients run a more prolonged and complicated course. In diabetes population, hepatitis B and C produce more comorbidities and prolonged infections.
    - Even though hepatitis B virus (HBV) itself may not cause diabetes directly, cirrhosis derived from HBV infection poses twofold higher risk for T2DM [20]. Infection due to HBV may occur during monitoring of blood glucose and other procedures involving multi-patient use of finger-stick devices designed for single-patient use and inadequate disinfection and cleaning of blood glucose monitors between patients [21].
    - When hepatitis C virus (HCV) infection occurs in diabetes patients, the chronicity as well as risk of infections further increase. In a meta-analysis of 22 studies, it was found that patients with T2DM were at higher risk for acquiring HCV than non-T2DM patients (OR, 53.50; 95% CI, 52.54, 54.82) [22].
    - Hepatitis A is the most common vaccine-preventable virus acquired during travel and it is highly prevalent in the Indian subcontinent. Protection with hepatitis A vaccination is proven to last at least 15 years [23].
  - *Tuberculosis*: Because diabetes impairs host defense mechanism, it has long been known to be a risk factor for active tuberculosis (TB) and reactivation of latent TB [24]. Evidence suggests that the risk of developing TB is increased among patients with diabetes, particularly during the first year after diagnosis of diabetes [25]. Furthermore, it is associated with worse treatment outcomes, higher rates of relapse, and higher mortality rates in patients affected by both diseases. It is estimated that 15% of TB cases globally could be attributed to diabetes and 40% of these cases are from India and China [26]. Moreover, in developing Asian countries, prevalence of TB among diabetes patients was 1.8–9.5 times higher than in the general population [26]. The situation is particularly challenging in low-income and middle-income countries where TB is endemic. Data from a systematic review of 13 observational studies indicate that efforts to diagnose, detect, and treat diabetes early may have a beneficial impact on TB control [27].

##### *Types of vaccines*

- Various types of vaccinations recommended to prevent these infections are:
  - *Pneumococcal vaccination*: Two pneumococcal vaccines are available: PPSV23 and PCV13. Secondary immune response after PCV13 immunization is higher, whereas response is lower after immunization with PPSV23 vaccine [28].
  - The panel recommends the use of PCV13 for adults ≥ 50 years followed by a dose of PPSV23 at least 1 year later (and at least 5 years after their previous PPSV23 dose) depending on the clinical judgment of the physician. These recommendations are in line with the guidelines from the ADA 2017 and are also in synergy with the guidelines released recently by the Indian Society of Nephrology 2016, Indian Academy of Allergy 2017, and the Geriatric Society of India 2015 [29–32].
  - PCV13 is available for vaccination of older adults and must be considered an important step for vaccinating older diabetes patients with age of > 50 years. PPSV23 may be offered to immune-compromised patients with diabetes for additional coverage after PCV13. Repeated vaccination with PPSV23 must be avoided to prevent hypo-responsiveness. Clinical judgment in relation to individual subjects should be relied upon before these recommendations are put into practice.
  - *Influenza vaccination*: In all patients with T2DM with age ≥ 6 months, excluding those who are allergic to eggs, influenza vaccine is recommended [33, 34]. Influenza vaccination among diabetes patients reduced hospital admissions by 79% in two influenza epidemics in England [35].
  - *HBV*: To all unvaccinated patients with diabetes of age 19–59 years, three dose series of HBV is recommended [34]. In unvaccinated patients with ≥ 60 years of age, three dose series vaccine could be considered [34].
- Apart from the vaccines mentioned above, other routinely recommended, age-related vaccines should also be provided to all diabetes patients [34].
- Annexure XIII provides brief information on recommended vaccines for patients with diabetes.

##### *Methods to improve rate of vaccination*

- Despite the importance of vaccination in diabetes patients, vaccination rates are low in them. In a survey on 307 diabetes patients in Singapore, only 30.6% of patients were found to be vaccinated with influenza vaccine [36]. Another cross-sectional survey on 279 diabetes patients in Spain determined the vaccination rates for seasonal influenza, pneumococcus, and hepatitis B as 40, 2, and 2%, respectively [37]. A survey on 274 elderly people in Turkey revealed that the proportion of diabetes patients vaccinated for influenza or pneumococcus or tetanus as 38.1, 13.4, and 9.28%, respectively [38].
- Perception, knowledge, and misconception that vaccines are infective and cause side effects are some of the barriers for avoiding vaccination [36, 37].
- Maintaining a diabetes registry, systemic tracking system, and reminder system serve as tools for improvising the acceptance to vaccination and communicating with the subjects for the need of vaccination which provides awareness on immunization [37, 39]. The combined used of patient outreach letters, special immunization clinics, standing orders, and practitioner reminders on medical records resulted in a remarkable 15-fold increase in pneumococcal vaccinations in diabetes patients in Guam, USA [40]. Similarly, a combination of strategies including dissemination of guidelines, advice on setting up disease and vaccine registers, call and recall systems, and benchmarking of performance remarkably improved influenza and pneumococcal vaccination rates in high-risk individual groups including diabetes patients in UK [41]. Periodic training of the staff accompanied by ongoing assessment of immunization rates and work flow and also a close follow-up with the patient or his caregiver by the treatment team are beneficial in minimizing the risk of inappropriate revaccinations [42].
- The protocols should also aim at implementing a quality assurance process so that the standards of care are maintained [43].

#### **Implementation**

Apart from the micro- and macrovascular events in diabetes, infections due to influenza and pneumococci should be considered as significant public health concern. All clinics providing vaccinations shall maintain the records to assess the efficacy of vaccines regarding occurrence of various complications in vaccinated individuals compared to non-vaccinated subjects. Vaccination strategies in diabetes should evolve as part of routine care and a central registry needs to be maintained.

**References**

1. Shah BR, Hux JE. Quantifying the risk of infectious diseases for people with diabetes. Diabetes Care 2003;26(2):510–3.
2. Klekotka RB, Mizgała E, Król W. The etiology of lower respiratory tract infections in people with diabetes. Advances in Respiratory Medicine. 2015;83(5):401–8.
3. Mohan V, Shanthirani CS, Deepa M, Deepa R, Unnikrishnan RI, Datta M. Mortality rates due to diabetes in a selected urban south Indian population—the Chennai Urban Population Study [CUPS–16]. J Assoc Physicians India 2006;54:113–7.
4. Dryden M, Baguneid M, Eckmann C, Corman S, Stephens J, Solem C, Li J, Charbonneau C, Baillon-Plot N, Haider S. Pathophysiology and burden of infection in patients with diabetes mellitus and peripheral vascular disease: focus on skin and soft-tissue infections. Clinical Microbiology and Infection. 2015;21:S27–32.
5. Kornum JB, Thomsen RW, Riis A, Lervang HH, Schønheyder HC, Sørensen HT. Diabetes, glycemic control, and risk of hospitalization with pneumonia: a population-based case-control study. Diabetes Care 2008;31(8):1541–5.
6. Yende S, van der Poll T, Lee M, Huang DT, Newman AB, Kong L, et al. The influence of pre-existing diabetes mellitus on the host immune response and outcome of pneumonia: analysis of two multicentre cohort studies. Thorax 2010;65(10):870–7.
7. Jensen AV, Egelund GB, Andersen SB, Petersen PT, Benfield T, Faurholt-Jepsen D, Rohde G, Ravn P. The impact of blood glucose on community-acquired pneumonia: a retrospective cohort study. ERJ Open Research. 2017;3(2):00114–2016.
8. Delamaire M, Maugendre D, Moreno M, Le Goff MC, Allannic H, Genetet B. Impaired leucocyte functions in diabetic patients. Diabet Med. 1997;14(1):29–34.
9. Gallacher SJ, Thomson G, Fraser WD, Fisher BM, Gemmell CG, MacCuish AC. Neutrophil bactericidal function in diabetes mellitus: evidence for association with blood glucose control. Diabet Med. 1995;12(10):916–20.
10. Calvet HM, Yoshikawa TT. Infections in diabetes. Infect Dis Clin North Am. 2001;15(2):407–21.
11. Casqueiro J, Casqueiro J, Alves C. Infections in patients with diabetes mellitus: a review of pathogenesis. Indian Journal of Endocrinology and Metabolism. 2012;16(Suppl1):S27.
12. Allard R, Leclerc P, Tremblay C, Tannenbaum TN. Diabetes and the severity of pandemic influenza A (H1N1) infection. Diabetes Care. 2010;33(7):1491–3.
13. Diepersloot RJ, Bouter KP, Hoekstra JB. Influenza infection and diabetes mellitus: case for annual vaccination. Diabetes Care. 1990;13(8):876–82.
14. Warren-Gash C, Smeeth L, Hayward AC. Influenza as a trigger for acute myocardial acute infarction or death from cardiovascular disease: a systematic review. Lancet Infect Dis 2009;9:601–10.
15. Manjunath C, Munichoodappa CS. Insulin resistance in diabetes mellitus due to chronic liver disease and retroviral infection. Int J Diabetes Dev Ctries. 2014;34(3):174.
16. Hove-Skovsgaard M, Gaardbo JC, Kolte L, Winding K, Seljeflot I, Svardal A, Berge RK, Gerstoft J, Ullum H, Trøseid M, Nielsen SD. HIV-infected persons with type 2 diabetes show evidence of endothelial dysfunction and increased inflammation. BMC Infectious Diseases. 2017;17(1):234.
17. Kumar P, Singh U. Malignant otitis externa—a review. Journal of Infectious Diseases and Therapy. 2015:1–4.
18. Kasim KM, Bujang MA, Hau Abdullah MA, Huan PC, Johari J, Ponniah JPS et al. Characteristics and outcome of patients with hand and upper limb infection in a diabetes patients. Int J Diabetes Dev Ctries 2015;35(2):123–8.
19. Chita T, Licker M, Sima A, Vlad A, Timar B, Sabo P et al. Prevalence of urinary tract infections in diabetic patients. Rom J Diabetes Nutr Metab Dis. 2013;20(2):99–105.
20. Zhang J, Shen Y, Cai H, Liu YM, Qin G. Hepatitis B virus infection status and risk of type 2 diabetes mellitus: a meta-analysis. Hepatology Research. 2015;45(11):1100–9.
21. Klonoff DC, Perz JF. Assisted monitoring of blood glucose: special safety needs for a new paradigm in testing glucose. J Diabetes Sci Technol 2010;4:1027–31.
22. Guo X, Jin M, Yang M, Liu K, Li JW. Type 2 diabetes mellitus and the risk of hepatitis C virus infection: a systematic review. Scientific reports. 2013;3:2981.
23. Ott JJ, Irving G, Wiersma ST. Long-term protective effects of hepatitis A vaccines. A systematic review. Vaccine. 2012;31(1):3–11.
24. The Lancet Diabetes Endocrinology. Diabetes and tuberculosis—a wake-up call. Lancet Diabetes Endocrinol. 2014;2(9):677.
25. Heo EY, Choi NK, Yang BR, Koo BK, Hwang SS, Lee CH, et al. Tuberculosis is frequently diagnosed within 12 months of diabetes mellitus. Int J Tuberc Lung Dis. 2015;19(9):1098–101.
26. Zheng C, Hu M, Gao F. Diabetes and pulmonary tuberculosis: a global overview with special focus on the situation in Asian countries with high TB-DM burden. Global health action. 2017;10(1):1–1.
27. Jeon CY, Murray MB. Diabetes mellitus increases the risk of active tuberculosis: a systematic review of 13 observational studies. PLoS Med. 2008;5(7):e152.
28. Jackson LA, Gurtman A, van Cleeff M, Frenck RW, Treanor J, Jansen KU, Scott DA, Emini EA, Gruber WC, Schmoele-Thoma B. Influence of initial vaccination with 13-valent pneumococcal conjugate vaccine or 23-valent pneumococcal polysaccharide vaccine on anti-pneumococcal responses following subsequent pneumococcal vaccination in adults 50 years and older. Vaccine. 2013;31(35):3594–602.
29. American Diabetes Association. Standards of medical care in diabetes—2015. The Journal of Clinical and Applied Research and Education. Diabetes Care. 2015; Vol 38(Supp: 1): S20-S30
30. Indian Society of Nephrology guidelines for vaccination in chronic kidney disease. Indian Journal of Nephrology. 2016;26 (Suppl 1):S1–30. Available at: http://isn-india.org/images/Indian-Society-of-Nephrology-Guidelines-for-vaccination-of-Chronic-Kidney-Disease.pdf
31. http://indianacademyofallergy.com/wp-content/uploads/2017/05/Recommendations-IAACON-2016-Print-version-1.pdf last accessed 10th September 2017.
32. Sharma OP; Expert committee. Indian recommendations for vaccination in older adults 2015. Geriatric Society of India. Available at: http://www.geriatricindia.com/publications.html. Last accessed 10th September 2017.
33. Muruganathan A, Guha S, Munjal YP, Agarwal SS, Parikh KK, Jha V, Jha AK, Abeywicreme I, Tiwaskar M, Nadkar MY, Pal J. Recommendations for vaccination against seasonal influenza in adult high risk groups: South Asian recommendations. The Journal of the Association of Physicians of India. 2016;64(7):3.
34. American Diabetes Association. Standards of medical care in diabetes. 2017. Available at: http://professional.diabetes.org/sites/professional.diabetes.org/files/media/dc_40_s1_final.pdf
35. Colquhoun AJ, Nicholson KG, Botha JL, Raymond NT. Effectiveness of influenza vaccine in reducing hospital admissions in people with diabetes. Epidemiology & Infection. 1997;119(3):335–41.
36. Tan EK, Lim LH, Teoh YL, Ong G, Bock HL. Influenza and seasonal influenza vaccination among diabetics in Singapore: knowledge, attitudes and practices. Singapore Medical Journal. 2010;51(8):623.
37. Alvarez CE, Clichici L, Guzmán-Libreros AP, Navarro-Francés M, Ena J. Survey of vaccination practices in patients with diabetes: a report examining patient and provider perceptions and barriers. Journal of Clinical & Translational Endocrinology. 2017;9:15–7.
38. Sahin S, Tasar PT, Guclu YA, Sengul HS, Bozkurt N, Garip A, Duman S, Akcicek F. Vaccinations rates in the elderly with diabetes mellitus. Advances in Aging Research. 2014;3(04):293.
39. Swenson CJ, Appel A, Sheehan M, Hammer A, Fenner Z, Phibbs S, et al. Using information technology to improve adult immunization delivery in an integrated urban health system. Jt Comm J Qual Patient Saf. 2012;38(1):15–23.
40. Kleschen MZ, Holbrook J, Rothbaum AK, Stringer RA, McInerney MJ, Helgerson SD. Improving the pneumococcal immunization rate for patients with diabetes in a managed care population: a simple intervention with a rapid effect. The Joint Commission Journal on Quality Improvement. 2000;26(9):538–46.
41. Siriwardena AN, Rashid A, Johnson M, Hazelwood L, Wilburn T. Improving influenza and pneumococcal vaccination uptake in high risk groups in Lincolnshire: a quality improvement report from a large rural county. Quality in Primary Care. 2003;11(1):19–28.
42. Centers for Disease Control and Prevention. Recommended adult immunization schedule—United States, 2012. J Midwifery Women’s Health 2012;57(2):188–95.
43. Kesavadev J, Misra A, Das AK, Saboo B, Basu D, Thomas N, et al. Suggested use of vaccines in diabetes. Indian J Endocrinol Metab. 2012;16(6):886–93.

## **Fasting and diabetes**

### **RSSDI 2017 recommendations**

#### **Recommended care**

- Fasting is best avoided in persons with T2DM especially if they also have:
  - Uncontrolled or unstable glycemic values
  - History of recurrent diabetic ketoacidosis (DKA), inter-current illness
  - Significant macrovascular complications
  - Significant microvascular complications
  - History of hypoglycemic unawareness
- T2DM patients should abstain from fasting if they are:
  - On intensive insulin therapy
  - Non-adherent to advice on diet, drug regimens, and daily activities
  - Experiencing frequent hypoglycemic episodes (hypoglycemia without any symptoms)
  - Antenatal or nursing
  - Elderly or children and continuing certain antidiabetic medications
- Patient-centered diabetes education with regular glucose monitoring and adjustment of treatment regimens should be recommended in all T2DM patients in order to minimize AEs related to diabetes such as hypoglycemia during fasting.
- Persons with diabetes who wish to fast must consult a physician prior to fasting and should be encouraged to participate in pre fast counselling/assessment to optimize monitoring/therapeutic strategies for optimal glycemic control.
- Lifestyle modification including patient centered fasting nutrition plan is recommended for all patients with T2DM to avoid further complications.
- During fasting, patients with diabetes should always:
  - Carry some sweets/other source of glucose to use in case of hypoglycemia
  - Carry identification card displaying diabetic status and current medication
  - Test blood glucose levels regularly (especially, if unwell during fasting)
  - Treat promptly if glucose levels are deranged
  - End the fast immediately if dehydrated or hypoglycemic
  - Discuss with the physician regarding change in dose and timing of insulin injections
- Patients with diabetes should be educated to increase the awareness on risks of fasting.
- Hypoglycemia may be prevented in four levels including primordial, primary, secondary, and tertiary, using Anticipate, Suspect, Act to treat, Prevent (ASAP) strategy.
- Metformin, incretin-based therapies like sitagliptin, vildagliptin, and liraglutide, and pioglitazone, newer sulfonylureas like gliclazide MR and glimepiride are the preferable agents to be used during fasting that is spread over a number of days or weeks.
- Rapid-acting insulin analogues may be preferred over regular human insulin due to their less risk of hypoglycemia and less postprandial glucose excursions in patients with T2DM who fast during Ramadan.

#### **Limited care**

- The principles for management of diabetes during fasting are as for recommended care.
- Consume complex carbohydrates like whole grain, potato, berries, citrus fruits, apple, nuts, and legumes at prefasting time and simple carbohydrates like bread, cereals, and rice, at postfasting time to reduce complications.

#### **Preamble**

Fasting is a common religio-cultural practice followed in different forms according to various religions across the world. Hindus perform fasting during Navaratri, Karva Chauth, and Guru Purnima, Muslims observe during Ramadan, Buddhist performs during Lent, and Jains keep fasting in occasion of Paryushana [1–3]. Religious fasting is a means of inculcating discipline in an individual but not to impose excessive hardship [4]. Fasting is a time of great spiritual growth and can also improve physical well-being if properly undertaken. Though preference for religious fasting is a personal decision, persons with diabetes may fast after careful risk assessment and counselling with healthcare professionals and religious leaders, who help the individual make an informed decision [5]. Fasting may place patients who are on antidiabetic medications at higher risk of hypoglycemia and associated complications. Furthermore, in special populations like pregnant individuals, elderly people, persons with concomitant diseases, and those with renal and hepatic impairment, the risk of complications may increase if proper care is not taken. Therefore, a patient-centered approach including diet plan and dose modification/omission with careful monitoring during fasting period may reduce the complications in patients with diabetes. Fasting may have different connotations in different religions. It does not necessarily mean to abstain from food. For example, in the Jain religion, many eat the last meal before sunset and is considered a form of prayer. Similarly in Ramadan, one who cannot fast can feed the needy to offer prayer. Depending on the degree of abstinence from food, fasts may be classified as follows:

**Table:** 

Type of fast
• Complete fasting: giving up food and water completely for a period
• Partial fasting: eating less than you need to avoid hunger
• Limiting the number of food items eaten
• Giving up favorite foods

However, it should be noted that almost all religions provide special concessions to believers who are ill, travelling, or unable to keep fasts due to some reason or the other.

#### **Considerations**

Based on the following factors, the glucose-lowering therapy/strategy during fasting period may be modified/altered.

**Table:** 

Fasting	Glucose-lowering therapy	Individual phenotype	Patient characteristics
• Duration of fast • Restriction of fluids/solids: absolute/partial • Frequency of fast (once weekly/once monthly/once yearly/others)	**•** Potential for hypoglycemia **•** Potential for dehydration **•** Potential for gastrointestinal upset **•** Duration of action	**•** Risk of hypoglycemia **•** Risk of hypoglycemia unawareness **•** Ability to self-monitor blood glucose	**•** Pregnancy **•** Elderly **•** Concomitant diseases **•** Adolescent and children

#### **Rationale and evidence**

##### *Pre fast counselling/assessment/education*

- Discussion about fasting should be initiated prior to the fast. This should include the potential discomforts and risks of fasting and means of mitigating them. The person’s exact perspective of fasting, including duration of fast, allowance for liquids and snacks during the day, acceptance of sublingual foods, and freedom to break the fast in case of significant discomfort, must be clarified [1].
- Prefast assessment comprises comprehensive history taking, physical examination, and investigations aimed at identifying stigmata of target organ damage, so that strategies can be made to optimize health during fasts [6]. Prefast counselling should include appropriate diabetes education, self-management practices, and hypoglycemia awareness training [7].
- An observational study including Indian patients reports that the knowledge of diabetes during the period of Ramadan among the Muslims was only 58.5 and 37.3% patients did not monitor their blood glucose levels in the previous Ramadan [8].
- Concerned physician, pharmacist, and health workers have great role in providing education and making awareness regarding the management of diabetes during fasting [9, 10].
- Factors that may increase the risk of hypoglycemia, hypoglycemia unawareness, and dehydration must be noted [11, 12]. The concept of shared decision-making and person-centeredness must be followed, in letter and in spirit while considering whether a particular individual can fast safely or not [13].
- A structured diabetes educational program should be given to the patients and their families, which gives information on risk quantification, physical activity, glucose monitoring, diet, hypoglycemia, dosage and timing of medications, and identification of the symptoms of complications [5].
- Evidence suggested that structured education program was associated with significantly less weight gain (*p* < 0.001) and hypoglycemic episodes (*p* < 0.001) with reduced risk of acute complications compared to those who were not educated during fasting [14, 15].
- A study including 774 diabetes patients report that those who received individualized education were more likely to modify their diabetes treatment plan during Ramadan (*p* < 0.0001), to perform SMBG at least twice daily during Ramadan (*p* < 0.0001), and to have improved knowledge about hypoglycemic signs and symptoms (*p* = 0.0007). Moreover, BMI (− 1.1 ± 2.4 vs − 0.2 ± 1.7 kg/m^2^, *p* < 0.0001) and A1C (− 0.7 ± 1.1 vs − 0.1 ± 1.3%, *p* < 0.0001) significantly reduced in these patients during Ramadan compared with who received usual care [16].
- SMBG should be considered as an important tool that helps both patients and physicians to practice safe decision-making regarding drug dosage and other aspects of management [17]. Evidence suggests that among patients with T2DM, an increase in frequency of SMBG was associated with better glycemic control in those who were on insulin and were able to adjust their regimen [18, 19]. Furthermore, Ramadan education and awareness in diabetes (READ) program including regular glucose monitoring report significantly less episodes of hypoglycemia and weight gain in patients with T2DM [14].

##### *Lifestyle modification and nutrition*

- Fasting, itself as a form of lifestyle modification for T2DM patients, if utilized properly, may result in several health benefits to the patients [1].
- Prefasting diet should include the diet containing slow release foods and patients with T2DM should not indulge in overeating in the postfasting period in order to avoid postprandial hyperglycemia [1, 20]. Therefore, complex carbohydrates like whole grains, potato, berries, citrus fruits, apple, nuts, and legumes at prefasting and simple carbohydrates like bread, cereals, rice, and pasta at postfasting may be more appropriate to reduce complications [1, 5].
- Diet during Ramadan should be individualized by balancing patient risk and adequacy of glycemic control. The Epidemiology of Diabetes and Ramadan (EPIDIAR) study reports that around 15–30% of the patients with diabetes either gain or lose their body weight during Ramadan period [21].
- Moderate to highly vigorous exercise should not be performed during fasting. However, routine daily activities can be continued [1, 5, 22].

##### *Religious fasts*

Though several guidelines are available for different aspects of diabetes care, fasting in diabetes poses a unique challenge [4, 23]. Designing randomized controlled trails to address the issues related to fasting in patients with diabetes is particularly difficult. Therefore, understanding the physiology of fasting and linking it to pathophysiology and clinical manifestation of diabetes are required to design strategies for glycemic management during fasting [24]. We summarize different religious fasts commonly observed in India that can have significant impact on metabolic and glycemic health in diabetes:

- *Ramadan fasting*: It is a principle ritual followed by Muslims during the sacred month of Ramadan (the ninth lunar month of Islamic/Hijri calendar) [5, 23]. During this month, all healthy adult Muslims abstain from food, drinks, and medication from dawn to dusk (sunset). Believers usually eat two times, one before dawn (Suhur) and one after sunset (Iftar). Hypoglycemia and dehydration are major complications associated with fasting though hyperglycemia may occur, due to overindulgence in food during the two main meals of Suhur and Iftar [25, 26]. Therefore, prefast risk stratification followed by a treatment tailored to individual needs appears to be the best management strategy. In addition, structured education enables patients to self-manage their condition better [5, 27].
- *Jain fasts*: There are two sects in the Jain religion, the Shwetambers and the Digambers. The fasts are similar in both sects, except for the duration of fasting during the pious month of Paryushana 8 days for the Shwetamber sect and 10 days for the Digamber sect. Jains usually fast from dusk to dawn, unlike Hindu fasting which extends from dawn to moon-rise [1, 3].
- *Hindu fasts*: Though not mandatory, most of the Hindus observe day-long and week-long fasts. Karva Chauth, Guru Purnima, Ekadashi, Makar Sakranti, and Holi Ashtami are some of the annual, monthly, and weekly fasts observed as part of various vows. During Navratras, which occur twice a year, Hindus observe longer fasts for a period of 9 days usually from dawn to moon-rise/star-rise. The day-long nature of Hindu fasts however makes it distinct from the month-long fasts of Ramadan and Buddhist Lent. Unlike in Islam, there are no universal rules laid down for Hindu fasts, and therefore data on metabolic effect of these fasts is scanty so far [1, 12].

##### Pharmacological management

- Metformin can be used safely in patients with diabetes during fasting due to minimal incidences of hypoglycemia; however, once daily dosing needs to be adjusted or modified to avoid complications [5].
- Newer generation sulfonylureas (gliclazide MR and glimepiride) should be preferred over older, long-acting sulfonylureas like glibenclamide and chlorpropamide during Ramadan fasting, as they are relatively more safe and economical [4, 5, 27].
- Pioglitazone was found to be safe and efficacious in lowering blood glucose in fasting subjects during Ramadan in combination with other antidiabetic agents [28]. However, it was associated with significant increase in body weight compared with placebo [29].
- Agents that can act on incretin system may maintain adequate glycemic control in a glucose-dependent manner, thus providing a safe alternative therapeutic option during Ramadan [24]:
  - Vildagliptin was found to be effective, safe, and well tolerated in T2DM patients fasting during Ramadan, with a consistently low incidence of hypoglycemia across studies, accompanied by good glycemic and weight control [30].
  - Switching antihyperglycemic treatment to sitagliptin from a sulfonylurea reduced the risk of symptomatic hypoglycemia by approximately 50% in patients who fasted during Ramadan [31, 32].
  - In Treat 4 Ramadan trial, liraglutide compared with sulfonylurea was well tolerated with more patients achieving target A1C, lose or maintain weight with no severe hypoglycemia and with high level of treatment satisfaction [33]. However, LIRA-Ramadan trial did not report any significant difference between liraglutide and sulfonylureas in terms of severe hypoglycemia (23.7 vs 20.9%), although weight loss (*p* = 0.0091) and A1C reduction (*p* < 0.0001) were significant in liraglutide group [34]. This suggests that liraglutide may be considered an effective therapy in combination with metformin during Ramadan.
- SGLT2 inhibitors may be used during fasting, in view of their low risk of hypoglycemia. However, the potential risk of dehydration must be taken into account. In a recent RCT, dapagliflozin reports significantly fewer incidences of hypoglycemia than sulfonylureas (6.9 vs 28.8%, respectively; *p* = 0.002). However, postural hypotension was greater in the dapagliflozin group but did not reach significance [35]. Furthermore, in a recent survey, 70% physicians report that SGLT2 inhibitors are safe and effective for T2DM management during Ramadan [36].
- Use of a rapid acting insulin analogue instead of regular human insulin before meals in patients with T2DM who fast during Ramadan was associated with less hypoglycemia and less PPG excursions [37].
- A recent RCT concluded that 40% dose as IDet at sunrise and 60% as Premix 70 before dinner was non-inferior to standard care in patients with T2DM during Ramadan and was associated with less AEs [38].
- Detailed information on categories of risk in patients with T2DM who fast during Ramadan can be found in Annexure XIV.
- Detailed information on recommended changes in treatment regimens of OADs and insulin in patients with T2DM who fast during Ramadan and other religious fasts can be found from in the annexure XV.

**References**

1. Kalra S, Bajaj S, Gupta Y, Agarwal P, Singh SK, Julka S, et al. Fasts, feasts and festivals in diabetes-1: Glycemic management during Hindu fasts. Indian J Endocrinol Metab. 2015a;19(2):198–203.
2. Latt TS, Kalra S. Managing diabetes during fasting—a focus on Buddhist Lent. Diabetes Voice. 2012;57:42–5.
3. Julka S, Sachan A, Bajaj S, Sahay R, Chawla R, Agrawal N, Saboo B, Unnikrishnan AG, Baruah MP, Parmar G, Kalra S. Glycemic management during Jain fasts. Indian Journal of Endocrinology and Metabolism. 2017;21(1):238.
4. Bashir MI, Pathan M, Raza SA, Ahmad J, Azad Khan AK, Ishtiaq O, et al. Role of oral hypoglycemic agents in the management of type 2 diabetes mellitus during Ramadan. Indian J Endocr Metab 2012;16(4):503–7.
5. International Diabetes Federation (IDF), in collaboration with the Diabetes and Ramadan (DAR) International Alliance. Diabetes and Ramadan: Practical Guidelines, April 2016, Available at https://www.idf.org/sites/default/files/IDF-DAR-Practical-Guidelines-Final-Low.pdf
6. Al Arouj M. Risk stratification of Ramadan fasting in person with diabetes. J Pak Med Assoc. 2015;65(5, Suppl 1):S18–21.
7. Al-Arouj M, Assaad-Khalil S, Buse J, Fahdil I, Fahmy M, Hafez S, et al. Recommendations for management of diabetes during Ramadan: update 2010. Diabetes Care. 2010;33(8):1895–902.
8. Zainudin SB, Ang DY, Soh AW. Knowledge of diabetes mellitus and safe practices during Ramadan fasting among Muslim patients with diabetes mellitus in Singapore. Singapore Medical Journal. 2017;58(5):246.
9. Almansour HA, Chaar B, Saini B. Fasting, diabetes, and optimizing health outcomes for Ramadan observers: a literature review. Diabetes Therapy. 2017:1–23.
10. Kannan S, Mahadevan S, Seshadri K, Sadacharan D, Velayutham K. Fasting practices in Tamil Nadu and their importance for patients with diabetes. Indian Journal of Endocrinology and Metabolism. 2016;20(6):858.
11. Fatima J, Karoli R, Chandra A, Naqvi N. Ramadan fasting in patients with type 2 diabetes mellitus: experience from a teaching hospital. Indian J Endocrinol Metab 2012;16(2):323–4.
12. Gupta L, Khandelwal D, Singla R, Gupta P, Kalra S. Pragmatic dietary advice for diabetes during Navratris. Indian Journal of Endocrinology and Metabolism. 2017;21(1):231.
13. Mohammed GA, Car N, Muacevic-katanec D. Fasting of persons with diabetes mellitus during Ramadan. Diabetologia. Croatica. 2002;31(2):75–84.
14. Bravis V, Hui E, Salih S, Mehar S, Hassanein M, Devendra D. Ramadan Education and Awareness in Diabetes (READ) programme for Muslims with type 2 diabetes who fast during Ramadan. Diabet Med. 2010;27(3):327–31.
15. Ahmedani MY, Haque MS, Basit A, Fawwad A, Alvi SF. Ramadan Prospective Diabetes Study: the role of drug dosage and timing alteration, active glucose monitoring and patient education. Diabet Med. 2012;29(6):709–15
16. McEwen LN, Ibrahim M, Ali NM, Assaad-Khalil SH, Tantawi HR, Nasr G, Mohammadmoradi S, Misha AA, Annabi FA, Ba-Essa EM, Bahijri SM. Impact of an individualized type 2 diabetes education program on clinical outcomes during Ramadan. BMJ Open Diabetes Research and Care. 2015;3(1):e000111.
17. Jabbar A. Glucose monitoring during Ramadan. J Pak Med Assoc. 2015;65(5 Suppl. 1): S51–3.
18. Franciosi M, Pellegrini F, De Berardis G, Belfiglio M, Di Nardo B, Greenfield S, et al. Self-monitoring of blood glucose in non-insulin-treated diabetic patients: a longitudinal evaluation of its impact on metabolic control. Diabet Med. 2005;22(7):900–6.
19. Pfützner A, Weissmann J, Mougiakakou S, Daskalaki E, Weis N, Ziegler R. Glycemic variability is associated with frequency of blood glucose testing and bolus: post hoc analysis results from the ProAct Study. Diabetes Technology & Therapeutics. 2015;17(6):392–7.
20. Chamsi-Pasha H, Aljabri KS. The diabetic patient in Ramadan. Avicenna Journal of Medicine. 2014;4(2):29.
21. Salti I, Bénard E, Detournay B, Bianchi-Biscay M, Le Brigand C, Voinet C, Jabbar A. A population-based study of diabetes and its characteristics during the fasting month of Ramadan in 13 countries results of the Epidemiology of Diabetes and Ramadan 1422/2001 (EPIDIAR) study. Diabetes care. 2004;27(10):2306–11.
22. Ramadan J, Telahoun G, Al-Zaid NS, Barac-Nieto M. Responses to exercise, fluid, and energy balances during Ramadan in sedentary and active males. Nutrition. 1999;15(10):735–9.
23. Pathan MF, Sahay RK, Zargar AH, Raza SA, Khan AK, Siddiqui NI, et al. South Asian Consensus Guideline: use of GLP-1 analogue therapy in diabetes during Ramadan. Indian J Endocr Metab 2012;16(4):525–7.
24. Bajaj S. Newer anti-diabetic drugs in Ramadan. J Pak Med Assoc. 2015;65(5 Suppl.1): S40–3.
25. Islam N. Oral anti-diabetics in Ramadan. J Pak Med Assoc. 2015;65(5 Suppl.1):S37–9.
26. Almalki MH, Alshahrani F. Options for controlling type 2 diabetes during Ramadan. Frontiers in endocrinology. 2016;7.
27. Kalra S, Aamir AH, Raza A, Das AK, Khan AA, Shrestha D, Qureshi MF, Fariduddin M, Pathan MF, Jawad F, Bhattarai J. Place of sulfonylureas in the management of type 2 diabetes mellitus in South Asia: a consensus statement. Indian Journal of Endocrinology and Metabolism. 2015;19(5):577.
28. Vasan S, Thomas N, Bharani, Ameen M, Abraham S, Job V, et al. A double-blind, randomized, multicentre study evaluating the effects of pioglitazone in fasting Muslim subjects during Ramadan. Int J Diab Dev Ctries. 2006;26(2):70–76.
29. Gray LJ, Dales J, Brady EM, Khunti K, Hanif W, Davies MJ. Safety and effectiveness of non-insulin glucose-lowering agents in the treatment of people with type 2 diabetes who observe Ramadan: a systematic review and meta-analysis. Diabetes, Obesity and Metabolism. 2015;17(7):639–48.
30. Hassanein M, Abdallah K, Schweizer A. A double-blind, randomized trial, including frequent patient-physician contacts and Ramadan-focused advice, assessing vildagliptin and gliclazide in patients with type 2 diabetes fasting during Ramadan: the STEADFAST study. Vasc Health Risk Manag. 2014;10:319–26.
31. Aravind SR, Ismail SB, Balamurugan R, Gupta JB, Wadhwa T, Loh SM, et al. Hypoglycemia in patients with type 2 diabetes from India and Malaysia treated with sitagliptin or a sulfonylurea during Ramadan: a randomized, pragmatic study. Curr Med Res Opin. 2012;28(8):1289–96.
32. Lee SW, Lee JY, San San Tan C, Wong CP. Strategies to make Ramadan fasting safer in type 2 diabetics: a systematic review and network meta-analysis of randomized controlled trials and observational studies. Medicine. 2016;95(2).
33. Brady EM, Davies MJ, Gray LJ, Saeed MA, Smith D, Hanif W, et al. A randomized controlled trial comparing the GLP-1 receptor agonist liraglutide to a sulphonylurea as add on to metformin in patients with established type 2 diabetes during Ramadan: the Treat 4 Ramadan Trial. Diabetes Obes Metab. 2014;16(6):527–36.
34. Azar ST, Echtay A, Wan Bebakar WM, Al Araj S, Berrah A, Omar M, Mutha A, Tornøe K, Kaltoft MS, Shehadeh N. Efficacy and safety of liraglutide compared to sulphonylurea during Ramadan in patients with type 2 diabetes (LIRA-Ramadan): a randomized trial. Diabetes Obes Metab. 2016;18(10):1025–33.
35. Wan Seman WJ, Kori N, Rajoo S, Othman H, Mohd Noor N, Wahab NA, Sukor N, Mustafa N, Kamaruddin NA. Switching from sulphonylurea to a sodium-glucose cotransporter 2 inhibitor in the fasting month of Ramadan is associated with a reduction in hypoglycaemia. Diabetes, Obesity and Metabolism. 2016;18(6):628–32.
36. Beshyah SA, Chatterjee S, Davies MJ. Use of SGLT2 inhibitors during Ramadan: a survey of physicians’ views and practical guidance. British Journal of Diabetes. 2016;16(1):20–4.
37. Kalra S, Jawad F. Insulin in Ramadan. J Pak Med Assoc. 2015;65(5, suppl 1):S44–6.
38. Shehadeh N, Maor Y. Effect of a new insulin treatment regimen on glycaemic control and quality of life of Muslim patients with T2DM mellitus during Ramadan fast—an open label, controlled, multicentre, cluster randomised study. International Journal of Clinical Practice. 2015;69(11):1281–8.

## **Diabetes and CV risk**

### **RSSDI 2017 recommendations**

#### **Recommended care**

- Cardiovascular risk factors that should be assessed in all patients at diagnosis and annually include:
  - Dyslipidemia
  - Hypertension
  - Smoking status
  - Family history of premature coronary disease
  - Presence of albuminuria
- Current or previous CVD events, BP, pulse, age, and body weight of patients should be recorded during their first and subsequent visits.
- IDRS, QRISK, and WHO-ISH are simple and effective tools for identifying and predicting CVD risks in patients with T2DM and should be recommended for identifying high-risk individuals.
- Patients with diabetes and CVD risk should follow the ABC treatment goals*.
  - A (A1C): < 7%
  - B (BP): < 130/80 mmHg
  - C (cholesterol-LDL): < 100 mg/dL

(*The treatment target goals should be individualized according to age, risk and comorbidity.)

- All patients should be managed with lifestyle intervention including physical exercise and DASH diet. If required, low-dose antiplatelet drugs, lipid-lowering agents, and BP-lowering medications can be considered.
- Yoga has shown efficacy in improving the dyslipidemia state, lower BMI, and macrovascular complications in patients with T2DM. Thus, yoga classes with different asana are recommended for all patients with CVD risk.
- Foods available in Indian subcontinent like oats, nuts, psyllium husk, cinnamon, flaxseeds, fenugreek, soy, Indian gooseberry, garlic, raagee, and white marudah should be recommended to the patients in order to reduce the CVD complications.
- In high-risk patients, low-dose antiplatelet therapy should be administered along with lifestyle intervention for secondary prevention.
- Statins should be added to lifestyle intervention in all patients with CVD risk, if not contraindicated, to reduce dyslipidemia. The intensity can be modified or titrated according to patient CVD risk, age, side effects, tolerability, LDL-C levels, etc.
- If triglyceride level remains > 200 mg/dL irrespective of statin therapy, addition of fibrates can be considered.
- Glycemic control with glucose-lowering drugs that are proven to be CV safe and beneficial should be recommended to reduce CVD risk and complications in patients with T2DM. Empagliflozin and liraglutide are approved by various regulatory authorities for CV risk reductions, apart from their glucose-lowering ability.
- Weight control should be an important consideration, while choosing glucose-lowering therapy in overweight/obese persons.
- Pharmacological antihypertensive therapy with subsequent titration in addition to lifestyle therapy should be initiated in patients with confirmed office-based BP of > 140/90 mmHg.
- Pharmacological therapy for patients with diabetes and hypertension should comprise of a regimen that includes ACE inhibitor, ARB, thiazide diuretics, calcium channel blockers, and β-blockers. If one class is not tolerated, it should be substituted with other class; however, FDCs of different drug classes may be preferred in patients with diabetes to reduce CVD risks and complications.
- ACE inhibitors are the drug of choice for diabetes, if not contraindicated, and ARBs may be used if ACE inhibitors are not tolerated.
- Other medications for dyslipidemia (bile acid binding resins, ezetimibe, sustained release nicotinic acid, concentrated omega-3 fatty acids) can be considered in patients failing to reach targets with conventional lipid-lowering medications.

#### **Limited care**

- Cardiovascular risk factors like albuminuria and atrial fibrillation should be assessed in all patients at diagnosis and annually.
- Cardiovascular risk may be calculated by using different assessment tools for people with diabetes.

#### **Preamble**

Patients with T2DM are always at higher risk for several CVDs such as CAD, CHF, stroke, PAD, and cardiomyopathy. Furthermore, compared to non-diabetes patients, T2DM patients have a considerably higher risk of CV morbidity and mortality [1]. In addition, the coexistence of risk factors like hypertension, abnormal cholesterol, high triglycerides, obesity, and smoking with T2DM may increase the burden and complications of CVD [2]. In India, CVD attributes to nearly 25% of all deaths. Furthermore, according to the Global Burden of Disease study, age-standardized CVD mortality rate was 272 per 100,000 population in India, which was higher than the global average of 235 per 100,000 population [3]. An Indian population-based study evaluated the prevalence of CVD risk factors in 6198 T2DM patients. A study reports that compared to participants with diabetes vs those without it, prevalence of hypertension was 73.1% (95% CI, 67.2 to 75.0) vs 26.5% (25.2 to 27.8), hypercholesterolemia was 41.4% (38.3 to 44.5) vs 14.7% (13.7 to 15.7), hypertriglyceridemia was 71.0% (68.1 to 73.8) vs 30.2% (28.8 to 31.5), low HDL-C was 78.5% (75.9 to 80.1) vs 37.1% (35.7 to 38.5), and incidence of smoking/smokeless tobacco use was 26.6% (23.8 to 29.4) vs 14.4% (13.4 to 15.4; *p* < 0.001) [4]. In CINDI study, risk factors for macrovascular complications including hypertension, obesity, and dyslipidemia were observed in 23.3, 26, and 27% of patients, respectively, and ischemic heart disease was seen in 6% of patients [5]. Furthermore, in the CINDI 2 study carried out later, CV risk factors such as hypertension, dyslipidemia, BMI > 23 kg/m^2^, and smoking were prevalent in 27.6, 62.4, 84.2, and 24% patients, respectively [6]. In addition, ischemic heart disease, PVD, and stroke were found in 0.7, 2, and 1% patients, respectively. Statin therapy was required in 95.3% of the patients. Moreover, history of diabetes was significantly associated with MI (OR, 2.37, PAR 9.9%, *p* < 0.0001) and stroke (OR, 1.16, 1.05–1.30; 3.9%, 1.9–7.6, *p* < 0.0001) in the effect of potentially modifiable risk factors associated with MI in 52 countries (INTERHEART) study and INTERSTROKE studies respectively [7, 8]. CAD and stroke constitute the majority of CVD mortality in India (83%), with CAD being predominant [9]. Therefore, aggressive controlling of these risk factors may delay or reduce the incidence of CVDs in T2DM patients.

#### **Considerations**

When framing recommendations for diabetes and CV risk, following factors should be reviewed: hypertension, smoking, obesity, increased fasting insulin and IR, lifestyle intervention, and atherogenic lipid profile (abnormal cholesterol, high triglycerides).

Primary prevention of CVDs aims at preventing patients from the event of CAD/CVD. This includes engaging in moderate physical activity, maintaining normal body weight, limiting alcohol consumption, reducing sodium intake, maintaining adequate intake of potassium, and consuming a diet rich in fruits, vegetables, and low-fat dairy products and less saturated and total fat. Secondary prevention of CVDs in patients with diabetes plans to reduce the mortality and morbidity and prevent the repeated CVD event. This comprises treatment with aspirin, β-blockers, ACE inhibitors, and statin. The tertiary prevention intends at rehabilitation, preventing complications, and improving QoL. This can be achieved with some interventional surgical procedures. Quaternary prevention targets at preventing overdiagnosis, overmedicalization, overlabelling, and overtreatment.

#### **Rationale and evidence**

##### *Identification*

Cardiovascular risk factors such as dyslipidemia, hypertension, smoking, family history of premature coronary disease, and the presence of albuminuria should be assessed at least annually in all patients with T2DM [2]. Even the CINDI and CINDI 2 studies on Indian population suggest for the screening of CV complications at the time of diagnosis itself [5, 6].

The following tools have been used by several physicians for assessment of the CVD risk in individuals with diabetes and CVD:

1. FRS [10]
2. QRISK Risk Score [11]
3. IDRS [12]
4. UKPDS Risk Engine [13]
5. SCORE tables [14]
6. WHO risk prediction charts [15]
7. American College of Cardiology/American Heart Association (ACC/AHA) atherosclerotic cardiovascular disease (ASCVD) risk calculator [16]
8. The 3rd Joint British Societies’ risk calculator (JBS) [17]

A cross-sectional study from India including 1007 participants reports that FRS was not able to differentiate the study participants properly with respect to CVD risk. However, the IDRS was found to produce better discriminatory performances in the study population [10]. In addition, IDRS can predict CV and diabetic risk more effectively than FRS and serve as simple and cost-effective tool for a primary care physician to identify at risk individuals for diabetes and CVD in Asian Indian women [12]. In another cross-sectional study, 900 participants were examined for CVD risk with WHO-ISH risk prediction chart. This study concluded that the WHO-ISH 10-year risk prediction chart was easier for assessing the CVD risk factors and risk grouping and could also be used to predict their 10-year risk of stroke or MI [18]. Furthermore, a prospective study reports that Indian patients presenting with acute MI and JBS risk score are likely to identify the largest proportion of the patients as at “high risk” as compared to WHO, FRS, and ACC/AHA [19].

##### ***Management***

- *Lifestyle intervention*: Early identification of metabolic syndromes such as AO, elevated BP, hypertriglyceridemia, reduced HDL cholesterol, borderline high-risk LDL cholesterol and IFG (110 to 126 mg/dL), and design interventions to reduce the CVD risks are the major goals of the primary prevention [20]. Furthermore, close monitoring and maintaining recommended targets for BP (130/80 mmHg), lipid control (LDL < 100 mg/dL), and glycemia (A1C < 7%) are important for the prevention of CVD in patients with T2DM [14, 20]. In addition, physical exercise, weight control, lifestyle modification with changing food habits, and cessation of smoking also prevent the CVD risk in T2DM patients [20].
- *Diet*: Substitution of dietary saturated fat with PUFAs is reported to be associated with improved CV outcomes. Several foods like oats, nuts, psyllium husk, cinnamon, flaxseeds, fenugreek, soy, amla, garlic, finger millet, and white marudah were also reported to have lipid-lowering property [21]. Moreover, American Family Physicians (AFP) advocates that the Mediterranean diet can reduce CV mortality and the DASH eating plan associates with a reduced risk of CAD [22]. Moreover, the following dietary adaptations can be made to lessen the development of CVDs in T2DM patients: reductions in caloric intake (by 500 to 800 kcal/day), total fat intake (especially saturated fat) and food portion sizes, increased consumption of dietary fiber, and moderate alcohol use **[**23].
- *Physical activity*: It is an independent and protective risk factor associated with reduced CV morbidity and mortality (OR, 0.86; *p* < 0.0001), and physical inactivity accounts for 12.2% of the population-attributable risk for acute MI and 6% of CAD with an estimated 0.68-year reduction in life expectancy [22]. The exercise-based cardiac rehabilitation (CR) is the cornerstone for secondary prevention of CVD. CR is associated with a 13 and 26% lower all-cause and CVD mortality respectively and a 31% reduction in hospital admissions at 12 months in patients with CAD [22]. Hence, AFP recommend that physical activity for adults should be at least 150 min of moderate-intensity aerobic activity per week, 75 min of vigorous-intensity aerobic activity per week, or an equivalent combination [24].
- *Yoga*: A randomized parallel study in India compared efficacy of yoga in addition to OADs. A study reports a significant reduction in total cholesterol, triglycerides, LDL-C, and body weight in patients after yoga [25]. Furthermore, evidence suggests that yoga also helps in reducing the blood glucose levels [26–28], lipid levels [26, 27], body weight, and BP [27] in patients with T2DM.
- *Stress management*: Evidence state that psychosocial stress has an association with the etiology and pathogenesis of CVDs [29]. Most notably, the INTERHEART and INTERSTROKE studies report that psychological factors have a strong effect towards MI (OR, 2.67; PAR, 32.5%, *p* < 0.0001) and ischemic stroke (OR, 2.20, 1.78–2.72; 17.4%, 13.1–22·6), respectively [7, 8]. In an RCT, cognitive behavioral therapy (CBT) had a 41% lower rate of fatal and non-fatal first recurrent CVD events (HR, 0.59; 95% CI, 0.42,0.83; *p* = 0.002), 45% fewer recurrent acute MI (0.55, 0.36–0.85; *p* = 0.007), and a non-significant 28% lower all-cause mortality (0.72, 0.40–1.30; *p* = 0.28) than the reference group after adjustment for other outcome-affecting variables during a mean 94 months of follow-up period [30]. Nonetheless, a recent Cochrane review did not find such associations of CVD events with the psychological interventions in CAD patients [31].

##### *Pharmacological management*

- Medical treatment with pharmacotherapies like aspirin, lipid-lowering drugs, and BP-controlling agents improves survival, extends QoL, reduces the need for intervention procedures, such as angioplasty and coronary artery bypass graft surgery, and decreases the incidence of subsequent MI [20].

##### *Antiplatelet therapy*

- Aspirin is widely used for secondary prevention of CVD; however; its use in primary prevention is still controversial [2]. A meta-analysis evaluated the efficacy of aspirin in primary prevention of CVD in T2DM patients. Study reports that low dose aspirin (75 mg/day) was allied with a reduction of MI and stroke among women with diabetes. Furthermore, a meta-analysis demonstrated 35% reduction in MI among men (RR, 0.65; 95% CI, 0.51 to 0.82; *p* < 0.01), but the results were not significant in women (RR, 0.90; 95% CI, 0.71 to 1.14; *p* = 0.37) [32]. However, a systemic review including 10 RCTs reports no CVD benefit and trials with diabetes subgroup analyses also showed any effect [33]. Similarly, a recent meta-analysis evaluated aspirin for primary prevention of CVD in patients with diabetes and reported no difference with respect to the risk of all-cause mortality (OR, 0.93; 95% CI, 0.81–1.06), individual atherosclerotic events, bleeding, gastrointestinal bleeding, or hemorrhagic stroke rates compared to placebo [34]. Furthermore, a meta-analysis (*n* = 4000) by the Antithrombotic Trialists’ (ATT) collaborators showed that the effects of aspirin on major vascular events were similar for patients with or without diabetes: RR 0.88 (95% CI, 0.67–1.15) and RR 0.87 (95% CI, 0.79–0.96), respectively [35].
- In patients with aspirin intolerance/allergy or patients at very high risk for CVD, clopidogrel is recommended [2, 36]. Evidence suggests that clopidogrel was significantly more effective than aspirin in secondary prevention of CVD in patients with diabetes [36]. Furthermore, dual antiplatelet therapy may be reasonable for up to a year after ACS [2].
- A Cochrane systematic review reports that use of clopidogrel plus aspirin was associated with a reduction in the risk of CV events and an increased risk of bleeding compared with aspirin alone. However, only in patients with acute non-ST coronary syndrome benefits outweigh harms [37].

##### *Lipid-lowering agents*

- A high prevalence of lipid abnormality in patients with T2DM positions them at high-risk category in the CVD risk stratifications. Elevated levels of atherogenic cholesterol (AC), generally measured as non-HDL‑C, plays a central role in CVD, especially among Asian Indians [38].
- For management of dyslipidemia, the primary goal is to reduce LDL-C levels to < 100 mg/dL by addition of drug therapy (statins) to maximal diet therapy. Furthermore, fibrates may be added if triglycerides remain > 200 mg/dL in patients receiving statin therapy [20]. Statins reported a significant benefit in CV risk reduction and showed significant primary and secondary prevention of CVD/CAD deaths in patients with diabetes [39–41].
- A recent meta-analysis investigating 4351 diabetes patients reports that compared with placebo, standard dose statin treatment resulted in a significant RRR of 15% in the occurrence of any major CV or cerebrovascular event (RR, 0.85; 95% CI, 0.79–0.91). Compared with standard dose statin treatment (simvastatin 20 mg, pravastatin 40 mg, or atorvastatin 10 mg), intensive-dose statin (simvastatin 80 mg or atorvastatin 80 mg) treatment resulted in an additional 9% RRR [42].
- Moreover, statins were reported to produce similar results in various studies in India [43, 44]. Evidence advocates atorvastatin has negligible or no ability to increase HDL-C, which is the key feature in patients with diabetes. Thus, other statins should probably be preferred to atorvastatin in patients with diabetes/MS [45].
- In addition, ADA recommend that either high intensity or moderate intensity statin therapy should be used together with lifestyle intervention according to patient age and ASCVD risk factors [2]. The details have been given in Annexure XVI. The Lipid Association of India expert consensus statement 2016 revealed that statin therapy is highly effective in lowering NHDL‑C, LDL‑C, apolipoprotein B, and remnant cholesterol, besides being remarkably safe [46]. Recent evidence shows a clear CVD benefit of lowering LDL-C with ezetimibe on top of a statin in patients with T2DM [47].
- Furthermore, in CAD/CAD risk-equivalent patients, ezetimibe addition onto simvastatin, atorvastatin, or rosuvastatin provided greater LDL-C reductions and goal attainment than those who up-titrated these statin therapies [48]. The Fenofibrate Intervention and Event Lowering in Diabetes (FIELD) study assessed the effect of fenofibrate on CV events in T2DM patients. Fenofibrate reduced total CV events, mainly due to fewer non-fatal MI and revascularizations but did not significantly reduce the risk of coronary events such as CAD death or non-fatal MI [49].
- Furthermore, USFDA states that the current evidence base is insufficient to support fibrates for CVD protection and that more trial evidence is needed [50]. Nonetheless, prescribing lipid-lowering agents in older people with T2DM (> 85 years) requires special consideration because exposure to higher doses (or higher potency) might increase the risk of adverse effects instead of improving life expectancy.

##### *Glucose-lowering drugs*

- Intensive glycemic control with antidiabetic drugs reduces CV risk and complications in patients with T2DM. A meta-analysis including large, long-term prospective RCTs (such as the UKPDS, the prospective pioglitazone clinical trial in macrovascular events [PROactive], the Action in Diabetes and Vascular Disease: Preterax and Diamicron MR Controlled Evaluation [ADVANCE] trial, the Veterans Affairs Diabetes Trial [VADT], and the Action to Control Cardiovascular Risk in Diabetes [ACCORD] trial) reports that intensive glycemic control was associated with 17% reduction in events of non-fatal MI (OR, 0.83; 95% CI, 0.75–0.93) and a 15% reduction in events of CAD (OR, 0.85; 0.77–0.93); however, the study did not find any significant effect on events of stroke (0.93, 0.81–1.06) or all-cause mortality (1.02, 0.87–1.19) [51].
- In a meta-analysis of 301 clinical trials, the CVD risk of all glucose-lowering drugs including metformin, sulfonylurea, thiazolidinedione, DPP4 inhibitor, AGI, SGLT2 inhibitors, GLP-1 analogue, meglitinides, and insulins was evaluated. The results indicated that there were no significant differences in the association between any of the nine glucose-lowering drugs alone or in combination and risk of CV mortality [52].
- Two SGLT2 inhibitors, empagliflozin and canagliflozin, were recently shown to provide CV benefits in patients with T2DM. Empagliflozin was reported to produce substantial reductions in CVD death (by 38%) and all-cause mortality (by 32%), as well as in hospitalization for HF (by 35%), as compared with standard care in EMPA-REG OUTCOME trial [53]. In the recently published CANVAS trial, canagliflozin significantly reduced the composite of death from CV causes, non-fatal MI, or nonfatal stroke (HR, 0.86; 95% CI, 0.75 to 0.97; *p* < 0.001 for non-inferiority; *p* = 0.02 for superiority) in T2DM patients with established CVD or at high risk for CV events [54].
- Similarly in LEADER trial, liraglutide 1.8 mg daily was associated with lower rates (patients) of death from CV causes (4.7 vs 6.0%; HR, 0.78; 95% CI, 0.66 to 0.93; *p* = 0.007) or any causes (8.2 vs 9.6%; HR, 0.85; 0.74 to 0.97; *p* = 0.02) compared to placebo in patients with T2DM [55]. Therefore, using these medications early in the course of management in high-risk T2DM patients could provide potential benefits from looming CVDs.
- Furthermore, in the future, CAROLENA trial (NCT01243424) will provide CV outcomes of linagliptin and glimepiride in patients with T2DM.

##### *BP-lowering agents*

- A tight control of BP with pharmacological therapy like ARBs, ACE inhibitors, or β-blockers, diuretics, and calcium channel blockers helps in minimizing CVD risks in patients with T2DM [2]. Tight control of blood glucose decreases the risk of microvascular complications, whereas tight control of BP reduces both micro- and macrovascular complications.
- ADA, IDF, and other organizations recommend a target BP of 130/80 mmHg in diabetes patients [2, 56]. Furthermore, patients with confirmed office-based BP > 140/90 mmHg in addition to lifestyle therapy should be initiated with pharmacological therapy to achieve BP goals [2].
- A meta-analysis including 147 RCTs involving 464,164 people reports a significant reduction in risk of coronary events (20–25%) and stroke (30–45%) with all five BP-lowering agents. However, calcium channel blockers had a greater preventive effect on stroke (RR, 0.92; 95% CI, 0.85 to 0.98) [57].
- Two meta-analyses and ACCORD study report that intensive BP control associated with a reduction of stroke event; however, these studies report more adverse effects [58–60]. In addition, in the ADVANCE trial, a fixed combination of perindopril and indapamide was associated with mean reduction in SBP of 5.6 mmHg and DBP of 2.2 mmHg after a mean of 4.3 years of follow-up in patients with T2DM. The relative risk of a major macrovascular or microvascular event was also reduced by 9% [61].
- Furthermore, some patients require a combination of two drugs in order to achieve a recommended BP target. Several Indian studies evaluated the efficacy of some FDCs, losartan 50 mg plus ramipril 2.5 mg vs each alone [62], metoprolol extended release (XL) plus amlodipine vs losartan plus amlodipine [63], and metoprolol and amlodipine [64], and reported that the FDCs were effective, safe, and well tolerated in patients with hypertension.

##### *Cardiovascular karma (metabolic memory)*

- Karma, an ancient term, suggests that right action with right intention consequently provides a right outcome in a persons’ life. This is also proved in the field of diabetes through STENO-2, EMPA-REG, and LEADER trials. Recent results of STENO-2 trial after 21 years follow-up report that an intensive, multifactorial intervention including ACE inhibitors/ARBs demonstrated a median of 7.9 years of gain of life in patients with T2DM [65]. The choice of individual agent for a person with diabetes may be influenced by a number of factors including their risk profile (CV, renal, end-organ damage), preferences, and previous experience of therapy, as well as costs. Moreover, a good karma may pass on to the unborn offspring of a pregnant mother with diabetes and also benefits the physician [66].

#### **Implementation**

Patients with diabetes and CVD risk should be assessed for complete lipid profile and BP measurement during their medical visits. Antiplatelet agents, lipid-lowering therapies, and antihypertensive medications along with lifestyle interventions should be provided with individualization and preference of each patient. Structured annual assessment and record-keeping should be instituted.

**References**

1. Martín-Timón I, Sevillano-Collantes C, Segura-Galindo A, del Cañizo-Gómez FJ. Type 2 diabetes and cardiovascular disease: have all risk factors the same strength. World J Diabetes. 2014;5(4):444–70.
2. American Diabetes Association. 8. Cardiovascular disease and risk management. Diabetes Care. 2016;39 (Supplement 1):S60–71.
3. Prabhakaran D, Jeemon P, Roy A. Cardiovascular diseases in India. Circulation. 2016;133(16):1605–20.
4. Gupta A, Gupta R, Sharma KK, Lodha S, Achari V, Asirvatham AJ, Bhansali A, Gupta B, Gupta S, Jali MV, Mahanta TG. Prevalence of diabetes and cardiovascular risk factors in middle-class urban participants in India. BMJ Open Diabetes Research and Care. 2014;2(1):e000048.
5. Sosale A, Kumar KP, Sadikot SM, Nigam A, Bajaj S, Zargar AH, Singh SK. Chronic complications in newly diagnosed patients with type 2 diabetes mellitus in India. Indian Journal of Endocrinology and Metabolism. 2014;18(3):355.
6. Sosale B, Sosale AR, Mohan AR, Kumar PM, Saboo B, Kandula S. Cardiovascular risk factors, micro and macrovascular complications at diagnosis in patients with young onset type 2 diabetes in India: CINDI 2. Indian Journal of Endocrinology and Metabolism. 2016;20(1):114.
7. Yusuf S, Hawken S, Ôunpuu S, Dans T, Avezum A, Lanas F, McQueen M, Budaj A, Pais P, Varigos J, Lisheng L. INTERHEART Study Investigators. Effect of potentially modifiable risk factors associated with myocardial infarction in 52 countries (the INTERHEART study): case-control study. Lancet. 2004;364(9438):937–52.
8. O’Donnell MJ, Chin SL, Rangarajan S, Xavier D, Liu L, Zhang H, Rao-Melacini P, Zhang X, Pais P, Agapay S, Lopez-Jaramillo P. Global and regional effects of potentially modifiable risk factors associated with acute stroke in 32 countries (INTERSTROKE): a case-control study. The Lancet. 2016;388(10046):761–75.
9. Institute of Health Metrics and Evaluation. GBD compare 2010. http://vizhub.healthdata.org/gbd-compare/. Accessed May 1, 2017.
10. Nag T and Ghosh A. Framingham risk score in estimating cardiovascular disease risk factors in people of Asian Indian origin: a study on rural adult population in West Bengal, India. ejbps, 2016; 3 (4): 415–421
11. Robson J, Hippisley‐Cox J, Coupland C. QRISK or Framingham? British Journal of Clinical Pharmacology. 2012;74(3):545–6.
12. Bhagat M, Ghosh A. Comparison of Framingham Risk Score and Indian diabetes risk score by obesity status and lipids abnormality in women of Asian Indian origin: Santiniketan women study. International Journal of Diabetes in Developing Countries. 2011;31(2):123–4.
13. Stevens RJ, Kothari V, Adler AI, Stratton IM, Holman RR. The UKPDS risk engine: a model for the risk of coronary heart disease in type II diabetes (UKPDS 56). Clinical Science. 2001;101(6):671–9.
14. Piepoli, Massimo F., Arno W. Hoes, Stefan Agewall, Christian Albus, Carlos Brotons, Alberico L. Catapano, Marie-Therese Cooney et al. “2016 European guidelines on cardiovascular disease prevention in clinical practice. The Sixth Joint Task Force of the European Society of Cardiology and Other Societies on Cardiovascular Disease Prevention in Clinical Practice (constituted by representatives of 10 societies and by invited experts) Developed with the special contribution of the European Association for Cardiovascular Prevention & Rehabilitation (EACPR).” European Journal of Preventive Cardiology (2016): 2047487316653709.
15. WHO/ISH Risk prediction charts for 14 WHO epidemiological sub-regions; Available from http://ish-world.com/downloads/activities/colour_charts_24_Aug_07.pdf
16. Goff DC, Lloyd-Jones DM, Bennett G, Coady S, D’Agostino RB, Gibbons R, Greenland P, Lackland DT, Levy D, O’Donnell CJ, Robinson J. 2013 ACC/AHA guideline on the assessment of cardiovascular risk. Circulation. 2013:01-cir.
17. Joint British Societies for prevention of Cardiovascular Disease. Available from http://www.jbs3risk.com/
18. Savitharani BB, Madhu B, Renuka M, Ashok NC. Utilization of WHO-ISH 10-year CVD risk prediction chart as a screening tool among supporting staff of a tertiary care hospital, Mysuru, India. Heart India. 2016;4(1):13.
19. Bansal M, Kasliwal RR, Trehan N. Comparative accuracy of different risk scores in assessing cardiovascular risk in Indians: a study in patients with first myocardial infarction. Indian Heart Journal. 2014;66(6):580–6.
20. Malik PK, Dwivedi S. Diabetes and cardiovascular diseases. JIMSA. 2015; 28(1): 61–63
21. Chandra KS, Bansal M, Nair T, Iyengar SS, Gupta R, Manchanda SC, Mohanan PP, Rao VD, Manjunath CN, Sawhney JP, Sinha N. Consensus statement on management of dyslipidemia in Indian subjects. Indian Heart Journal. 2014;66:S1–51.
22. Varghese T, Schultz WM, McCue AA, Lambert CT, Sandesara PB, Eapen DJ, Gordon NF, Franklin BA, Sperling LS. Physical activity in the prevention of coronary heart disease: implications for the clinician. Heart. 2016:heartjnl-2015.
23. Buttar HS, Li T, Ravi N. Prevention of cardiovascular diseases: role of exercise, dietary interventions, obesity and smoking cessation. Experimental & Clinical Cardiology. 2005;10(4):229.
24. Lanier JB, Bury DC, Richardson SW. Diet and physical activity for cardiovascular disease prevention. American Family Physician. 2016;93(11).
25. Shantakumari N, Sequeira S. Effects of a yoga intervention on lipid profiles of diabetes patients with dyslipidemia. Indian Heart Journal. 2013;65(2):127–31.
26. Cui J, Yan JH, Yan LM, Pan L, Le JJ, Guo YZ. Effects of yoga in adults with type 2 diabetes mellitus: a meta‐analysis. Journal of Diabetes Investigation. 2017;8(2):201–9.
27. Innes KE, Selfe TK. Yoga for adults with type 2 diabetes: a systematic review of controlled trials. Journal of Diabetes Research. 2015;2016.
28. Chimkode SM, Kumaran SD, Kanhere VV, Shivanna R. Effect of yoga on blood glucose levels in patients with type 2 diabetes mellitus. Journal of Clinical and Diagnostic Research: JCDR. 2015;9(4):CC01.
29. Steptoe A, Kivimäki M. Stress and cardiovascular disease. Nature Reviews Cardiology. 2012;9(6):360–70.
30. Gulliksson M, Burell G, Vessby B, Lundin L, Toss H, Svärdsudd K. Randomized controlled trial of cognitive behavioral therapy vs standard treatment to prevent recurrent cardiovascular events in patients with coronary heart disease: Secondary Prevention in Uppsala Primary Health Care project (SUPRIM). Archives of Internal Medicine. 2011;171(2):134–40.
31. Richards SH, Anderson L, Jenkinson CE, Whalley B, Rees K, Davies P, Bennett P, Liu Z, West R, Thompson DR, Taylor RS. Psychological interventions for coronary heart disease. Cochrane Database Syst Rev. 2017;4:CD002902.
32. Xie M, Shan Z, Zhang Y, Chen S, Yang W, Bao W, Rong Y, Yu X, Hu FB, Liu L. Aspirin for primary prevention of cardiovascular events: meta-analysis of randomized controlled trials and subgroup analysis by sex and diabetes status. PLOS One. 2014;9(10):e90286.
33. Guirguis-Blake JM, Evans CV, Senger CA, Rowland MG, O’Connor EA, Whitlock EP. Aspirin for the primary prevention of cardiovascular events: a systematic evidence review for the U.S. Preventive Services Task Force [Internet]. Rockville (MD): Agency for Healthcare Research and Quality (US); 2015 Sep. Available from http://www.ncbi.nlm.nih.gov/books/NBK321623/ PubMed PMID: 26491760.
34. Kokoska LA, Wilhelm SM, Garwood CL, Berlie HD. Aspirin for primary prevention of cardiovascular disease in patients with diabetes: a meta-analysis. Diabetes Research and Clinical Practice. 2016;120:31–9.
35. Baigent C, Blackwell L, Collins R, Emberson J, Godwin J, Peto R, Buring J, Hennekens C, Kearney P, Meade T, Patrono C. Aspirin in the primary and secondary prevention of vascular disease: collaborative meta-analysis of individual participant data from randomised trials. Lancet. 2009;373(9678):1849–60.
36. Tufano A, Cimino E, Di Minno MN, Ierano P, Marrone E, Strazzullo A, Di Minno G, Cerbone AM. Diabetes mellitus and cardiovascular prevention: the role and the limitations of currently available antiplatelet drugs. International Journal of Vascular Medicine. 2011.
37. Squizzato A, Keller T, Romualdi E, Middeldorp S. Clopidogrel plus aspirin versus aspirin alone for preventing cardiovascular disease. The Cochrane Library. 2011.
38. Joshi P, Islam S, Pais P, Reddy S, Dorairaj P, Kazmi K, Pandey MR, Haque S, Mendis S, Rangarajan S, Yusuf S. Risk factors for early myocardial infarction in South Asians compared with individuals in other countries. JAMA. 2007;297(3):286–94.
39. Macchia A, Laffaye N, Comignani PD, Pucci EC, Igarzabal C, Scazziota AS, Herrera L, Mariani JA, Bragagnolo JC, Catalano H, Tognoni G. Statins but not aspirin reduce thrombotic risk assessed by thrombin generation in diabetic patients without cardiovascular events: the RATIONAL trial. PLOS One. 2012;7(3):e32894.
40. Shepherd J, Barter P, Carmena R, Deedwania P, Fruchart JC, Haffner S, Hsia J, Breazna A, LaRosa J, Grundy S, Waters D. Effect of lowering LDL cholesterol substantially below currently recommended levels in patients with coronary heart disease and diabetes. Diabetes Care. 2006;29(6):1220–6.
41. Knopp RH. Efficacy and safety of atorvastatin in the prevention of cardiovascular end points in subjects with type 2 diabetes: the atorvastatin study for prevention of coronary heart disease endpoints in non-insulin-dependent diabetes mellitus (ASPEN): response to Gazi and Mikhailidis. Diabetes Care. 2006;29(11):2561–3.
42. de Vries FM, Kolthof J, Postma MJ, Denig P, Hak E. Efficacy of standard and intensive statin treatment for the secondary prevention of cardiovascular and cerebrovascular events in diabetes patients: a meta-analysis. PLOS One. 2014;9(11):e111247.
43. Gupta R, Lodha S, Sharma KK, Sharma SK, Gupta S, Asirvatham AJ, Mahanta BN, Maheshwari A, Sharma DC, Meenawat AS, Khedar RS. Evaluation of statin prescriptions in type 2 diabetes: India Heart Watch-2. BMJ Open Diabetes Research and Care. 2016;4(1):e000275.
44. Enas EA, Kuruvila A, Khanna P, Pitchumoni CS, Mohan V. Benefits & risks of statin therapy for primary prevention of cardiovascular disease in Asian Indians—a population with the highest risk of premature coronary artery disease & diabetes. Indian Journal of Medical Research. 2013;138(4):461.
45. Doggrell SA. Is atorvastatin superior to other statins? Analysis of the clinical trials with atorvastatin having cardiovascular endpoints. Reviews on Recent Clinical Trials. 2006;1(2):143–53.
46. Enas EA, Dharmarajan TS. The Lipid Association of India Expert Consensus Statement 2016: a sea change for management of dyslipidemia in Indians. Journal of Clinical and Preventive Cardiology. 2016;5(2):62.
47. Cannon CP, Blazing MA, Giugliano RP, McCagg A, White JA, Theroux P, Darius H, Lewis BS, Ophuis TO, Jukema JW, De Ferrari GM. Ezetimibe added to statin therapy after acute coronary syndromes. New England Journal of Medicine. 2015;372(25):2387–97.
48. Foody JM, Toth PP, Tomassini JE, Sajjan S, Ramey DR, Neff D, Tershakovec AM, Hu H, Tunceli K. Changes in LDL-C levels and goal attainment associated with addition of ezetimibe to simvastatin, atorvastatin, or rosuvastatin compared with titrating statin monotherapy. Vascular Health and Risk Management. 2013;9:719.
49. FIELD Study Investigators. Effects of long-term fenofibrate therapy on cardiovascular events in 9795 people with type 2 diabetes mellitus (the FIELD study): randomised controlled trial. The Lancet. 2005;366(9500):1849–61.
50. Goldfine AB, Kaul S, Hiatt WR. Fibrates in the treatment of dyslipidemias—time for a reassessment. The New England Journal of Medicine. 2011;365(6):481.
51. Ray KK, Seshasai SR, Wijesuriya S, Sivakumaran R, Nethercott S, Preiss D, Erqou S, Sattar N. Effect of intensive control of glucose on cardiovascular outcomes and death in patients with diabetes mellitus: a meta-analysis of randomised controlled trials. The Lancet. 2009;373(9677):1765–72.
52. Palmer SC, Mavridis D, Nicolucci A, Johnson DW, Tonelli M, Craig JC, Maggo J, Gray V, De Berardis G, Ruospo M, Natale P. Comparison of clinical outcomes and adverse events associated with glucose-lowering drugs in patients with type 2 diabetes: a meta-analysis. JAMA. 2016;316(3):313–24.
53. Zinman B, Wanner C, Lachin JM, Fitchett D, Bluhmki E, Hantel S, Mattheus M, Devins T, Johansen OE, Woerle HJ, Broedl UC. Empagliflozin, cardiovascular outcomes, and mortality in type 2 diabetes. New England Journal of Medicine. 2015;373(22):2117–28.
54. Neal B, Perkovic V, Mahaffey KW, de Zeeuw D, Fulcher G, Erondu N, Shaw W, Law G, Desai M, Matthews DR. Canagliflozin and cardiovascular and renal events in type 2 diabetes. New England Journal of Medicine. 2017.
55. Marso SP, Daniels GH, Brown-Frandsen K, Kristensen P, Mann JF, Nauck MA, Nissen SE, Pocock S, Poulter NR, Ravn LS, Steinberg WM. Liraglutide and cardiovascular outcomes in type 2 diabetes. New England Journal of Medicine. 2016;375(4):311–22.
56. Global guideline for type 2 diabetes. Res Clin Pract. 2014;104:1–52. http://www.idf.org/sites/default/files/IDF-Guideline-for-Type-2-Diabetes.pdf
57. Law M, Morris JK, Wald NJ. Use of blood pressure lowering drugs in the prevention of cardiovascular disease: meta-analysis of 147 randomised trials in the context of expectations from prospective epidemiological studies. BMJ. 2009;338:b1665.
58. Bangalore S, Kumar S, Volodarskiy A, Messerli FH. Blood pressure targets in patients with coronary artery disease: observations from traditional and Bayesian random effects meta-analysis of randomised trials. Heart. 2013;99(9):601–13.
59. Thomopoulos C, Parati G, Zanchetti A. Effects of blood pressure lowering on outcome incidence in hypertension: 7. Effects of more vs. less intensive blood pressure lowering and different achieved blood pressure levels—updated overview and meta-analyses of randomized trials. Journal of Hypertension. 2016;34(4):613–22.
60. ACCORD Study Group. Effects of intensive blood-pressure control in type 2 diabetes mellitus. N Engl j Med. 2010;2010(362):1575–85.
61. Patel A, ADVANCE Collaborative Group. Effects of a fixed combination of perindopril and indapamide on macrovascular and microvascular outcomes in patients with type 2 diabetes mellitus (the ADVANCE trial): a randomised controlled trial. The Lancet. 2007;370(9590):829–40.
62. Joshi SR, Yeolekar ME, Tripathi KK, Giri J, Maity AK, Chopda M, Gujarathi S, Maroli S, Maity A. Evaluation of efficacy and tolerability of Losartan and Ramipril combination in the management of hypertensive patients with associated diabetes mellitus in India (LORD Trial). The Journal of the Association of Physicians of India. 2004;52:189–95.
63. Pareek A, Chandurkar NB, Sharma R, Tiwari D, Gupta BS. Efficacy and tolerability of a fixed-dose combination of metoprolol extended release/amlodipine in patients with mild-to-moderate hypertension. Clinical Drug Investigation. 2010;30(2):123–31.
64. Rao NS, Oomman A, Bindumathi PL, Sharma V, Rao S, Moodahadu LS, Patnaik A, Kumar BN. Efficacy and tolerability of fixed dose combination of metoprolol and amlodipine in Indian patients with essential hypertension. Journal of Mid-Life Health. 2013;4(3):160.
65. Gæde P, Oellgaard J, Carstensen B, Rossing P, Lund-Andersen H, Parving HH, Pedersen O. Years of life gained by multifactorial intervention in patients with type 2 diabetes mellitus and microalbuminuria: 21 years follow-up on the Steno-2 randomised trial. Diabetologia. 2016;59(11):2298–307.
66. Kalra S, Ved J, Baruah MP. Diabetes destiny in our hands: achieving metabolic karma. Indian Journal of Endocrinology and Metabolism. 2017;21(3):482.

## **Hypoglycemia**

### **RSSDI 2017 recommendations**

#### **Recommended care**

- Risk of hypoglycemia should be assessed in every visit in patients with T2DM by using questionnaires.
- Patient should be well educated and informed regarding:
  - The symptoms, causes, and risks associated with hypoglycemia
  - Usage of SMBG tools with frequent monitoring especially patients taking insulin
  - Insulin dose adjustment considering blood glucose values
- A strict monitoring of hypoglycemic episodes is recommended for patients taking insulin, sulfonylureas, or meglitinides alone or in combination.
- Modern insulins or modern sulfonylureas should be used instead of respective traditional drugs in patients with high risk of hypoglycemia.
- Oral glucose (15–20 g) is preferred in conscious hypoglycemic patients (glucose alert value of < 70 mg/dL). Repeat the treatment, if SMBG shows continued hypoglycemia after 15 min. Patient should consume a meal or snack once SMBG returns to normal, to prevent recurrence of hypoglycemia
- Intramuscular glucagon or intravenous glucose is preferred for unconscious patients or patients with clinically significant hypoglycemia (glucose alert value of < 54 mg/dL). Repeat intramuscular or subcutaneous glucagon dose of 0.5 mg if there is no symptomatic improvement.
- Treatment should be modified in the event of hypoglycemia occurring repeatedly at a particular time of the day or in the event of hypoglycemia unawareness.

#### **Limited care**

- All patients with risk of hypoglycemia should be enquired about symptomatic and asymptomatic hypoglycemia at each visit.
- Patients along with their family members should be well educated about identification and management of hypoglycemia.
- Hypoglycemia should be strictly managed and monitored in special situations such as elderly, pregnancy, fasting, and metabolic disorders.

#### **Preamble**

Hypoglycemia is a major cause of concern with some antidiabetic drugs during the course of glycemic management in patients with T2DM [1]. However, the extent of hypoglycemia varies with different antidiabetic drugs pertaining to their pharmacokinetic and pharmacodynamic properties. The International Hypoglycemia Study Group categorizes hypoglycemia into three categories basing upon the glycemic criteria [2].

- Glucose alert value (level 1): < 70 mg/dL (3.9 mmol/L)
- Clinically significant hypoglycemia (level 2): < 54 mg/dL (3.0 mmol/L)
- Severe hypoglycemia (level 3): no specific glucose threshold

The prevalence of hypoglycemia in patients with T2DM in India is quite high. A recent cross-sectional study reports that nearly 96% of patients (out of 366 patients) were associated with at least one or other symptoms of hypoglycemia (dizziness, weakness). Furthermore, patients taking insulin in addition to OADs were at higher risk than patient taking OADs alone (OR, 2.3; *p* < 0.01) [3]. Meanwhile, another cross-sectional study including 1650 subjects from South India revealed that the cumulative incidence of institutional hypoglycemia was 12.36%; among which, 26.96% had asymptomatic episodes [4]. Severe hypoglycemia can lead to several diabetes-related short- and long-term complications such as precipitation of acute cerebrovascular disease, MI, neurocognitive dysfunction, retinal cell death, and loss of vision [5] and may lead to coma or death if not reversed [1]. The ACCORD and ADVANCE trial and other evidences report that severe hypoglycemia was directly associated with mortality in patients with T2DM [6–8]. Furthermore, *Kalra et al*. stated that diabetes patients with severe hypoglycemia are associated with sixfold increase in deaths over those not experiencing it [5]. Therefore, urgent steps need to be taken with some corrective measures against hypoglycemia in T2DM patients to minimize the burden. Following are some of the causes and risk factors of hypoglycemia [5].

**Table:** 

Causes and risk factors for hypoglycemia	
Causes	**Risk factors**
• Metabolic defects • Autoimmune conditions • Inborn errors of metabolism • Dietary toxins • Alcohol consumption • Stress • Infections • Starvation • Severe excessive exercise	**•** Glucose-lowering drugs (especially sulfonylureas/insulin) **•** Increased glucose utilization **•** Decreased glucose production **•** Female gender **•** Sleep **•** Duration of diabetes **•** Age **•** Progressive insulin deficiency **•** Intensive diabetes treatment with OADs and insulin combination

#### **Considerations**

Several factors such as the intensity of hypoglycemic risk, patient characteristics, drug usage, fasting, and patient education should be considered during framing the recommendations for hypoglycemia management in patients with T2DM.

#### **Rationale and evidence**

##### *Identification*

- Symptoms of hypoglycemia include, but not limited to, excess sweating and hunger, dizziness, blackout, fainting, fatigue, light-headedness or shakiness, nausea or vomiting, mental confusion or unresponsiveness, and dryness or tingling lips [1].
- Some endocrinologists or diabetologists use a three-step approach (Whipple’s Triad) for diagnosis of hypoglycemia. It includes:
  - Low blood glucose level
  - Symptoms of hypoglycemia at the time of the low glucose level
  - Symptom relief with treatment of hypoglycemia
    - *Management*
- Management of hypoglycemia can be subdivided into three aspects:

- Prevention of hypoglycemia
- Adjustment or withdrawal or modification of current antidiabetic regimen
- Treatment of hypoglycemia

##### *Prevention of hypoglycemia*

- Prevention of hypoglycemia is preferable than treatment, as it is much more likely to avoid severe events and economic burden [9]. Hypoglycemia prevention requires a combined effort from physician as well as patient. Patient education, patient counselling, and continuous blood glucose monitoring are the critical factors that need to be considered for the prevention of hypoglycemia in patients with diabetes. Evidence suggests that a proper and structured diabetes education helps in reducing diabetic complications including hypoglycemia [10–13].
- Furthermore, interventions targeting health beliefs and attitudes about hypoglycemia and diabetes self-management can be more effective than knowledge-centered patient education, which focuses on “symptom perception” in reducing hypoglycemia unawareness [5]. Patients receiving insulin for the treatment of T2DM can be benefitted by adjusting insulin doses following SMBG procedure [5, 14]. In addition, a cross-sectional study from India reports that 85% of patients were taking timely meals to prevent hypoglycemia [3]. Stratifying patients according to age and avoiding very tight glucose control in elderly patients (> 70 years) and very young children < 5 years of age will help to prevent hypoglycemia in these high-risk people.

##### *Adjustment or withdrawal or modification of ongoing antidiabetic regimen*

- Majority of the antidiabetic agents can produce hypoglycemia; however, the intensity depends upon their mechanism of action. Insulin, sulfonylureas, and meglitinides due to their glucose-independent mechanism of action cause a high risk of hypoglycemia [5].
- The UK Hypoglycemia Study Group report that the incidence of severe hypoglycemia increased from 7 to 25% in patients treated with insulin for < 2 years with those treated for > 5 years [15]. However, modern insulin analogues report lower incidence of hypoglycemia than traditional human insulins [16–18].
- Among all sulfonylureas, modern sulfonylureas like gliclazide MR and glimepiride are associated with lesser hypoglycemic episodes [19, 20]. Meglitinides were reported to inflict high rates of hypoglycemia [21]. In special situations like elderly, fasting, metabolic disorders, and pregnancy, the dose of these drugs should be adjusted or modified to avoid further complications. Furthermore, avoid/reduce the dose of insulin in people with CKD who have a tendency to develop hypoglycemia.

##### *Treatment of hypoglycemia*

- Fifteen to 20 g of carbohydrate (four teaspoons of sugar or glucose) can be given orally to a conscious patient with hypoglycemia; if unconscious, glucagon injection intramuscularly or glucose injection intravenously can be preferred [1, 5].
- Care takers of hypoglycemia-prone diabetes patients (family members, roommates, school personnel, child care providers, correctional institution staff, or coworkers) should be well instructed on the use of glucagon kits including where the kit is located and when and how to administer glucagon [1].
- Acute glycemic response correlates better with the glucose content than with the carbohydrate content of food. Therefore, pure glucose is the preferred treatment [1]. Fifteen minutes after glucose administration, an SMBG should be done and the treatment should be repeated if hypoglycemia persists. Patient should be advised to eat a regular meal or have a snack to prevent recurrence of hypoglycemia [22].

#### **Implementation**

Patient empowerment with hypoglycemia monitoring tools, hypoglycemia risk awareness, and the available preventive strategies together with physician-patient collaboration plan of treatment can reduce the frequency and intensity of hypoglycemia.

**References**

1. American Diabetes Association. Standards of medical care in diabetes. 2017. Available at: http://professional.diabetes.org/sites/professional.diabetes.org/files/media/dc_40_s1_final.pdf
2. International Hypoglycaemia Study Group. Glucose concentrations of less than 3.0 mmol/L (54 mg/dL) should be reported in clinical trials: a joint position statement of the American Diabetes Association and the European Association for the Study of Diabetes. Diabetes Care. 2017;40(1):155–7.
3. Shriraam V, Mahadevan S, Anitharani M, Jagadeesh NS, Kurup SB, Vidya TA, Seshadri KG. Reported hypoglycemia in type 2 diabetes mellitus patients: prevalence and practices—a hospital-based study. Indian Journal of Endocrinology and Metabolism. 2017;21(1):148.
4. Vikas PV, Chandrakumar A, Dilip C, Suriyaprakash TN, Thomas L, Surendran R. Incidence and risk factors of hypoglycemia among type 2 diabetic patients in a South Indian hospital. Diabetes & Metabolic Syndrome: Clinical Research & Reviews. 2016;10(2):S22–5.
5. Kalra S, Mukherjee JJ, Venkataraman S, Bantwal G, Shaikh S, Saboo B, Das AK, Ramachandran A. Hypoglycemia: The neglected complication. Indian Journal of Endocrinology and Metabolism. 2013;17(5):819.
6. ACCORD Study Group. Effects of intensive blood-pressure control in type 2 diabetes mellitus. N Engl J Med. 2010;2010(362):1575–85.
7. Zoungas S, Patel A, Chalmers J, de Galan BE, Li Q, Billot L, Woodward M, Ninomiya T, Neal B, MacMahon S, Grobbee DE. Severe hypoglycemia and risks of vascular events and death. New England Journal of Medicine. 2010;363(15):1410–8.
8. McCoy RG, Van Houten HK, Ziegenfuss JY, Shah ND, Wermers RA, Smith SA. Increased mortality of patients with diabetes reporting severe hypoglycemia. Diabetes Care. 2012;35(9):1897–901.
9. Shafiee G, Mohajeri-Tehrani M, Pajouhi M, Larijani B. The importance of hypoglycemia in diabetic patients. Journal of Diabetes & Metabolic Disorders. 2012;11(1):17.
10. Bravis V, Hui E, Salih S, Mehar S, Hassanein M, Devendra D. Ramadan Education and Awareness in Diabetes (READ) programme for Muslims with type 2 diabetes who fast during Ramadan. Diabet Med. 2010;27(3):327–31.
11. Norris SL, Lau J, Smith SJ, Schmid CH, Engelgau MM. Self-management education for adults with type 2 diabetes. Diabetes Care. 2002;25(7):1159–71.
12. Ahmedani MY, Haque MS, Basit A, Fawwad A, Alvi SF. Ramadan Prospective Diabetes Study: the role of drug dosage and timing alteration, active glucose monitoring and patient education. Diabet Med. 2012 Jun;29(6):709–15
13. Tenzer-Iglesias P, Shannon MH. Managing hypoglycemia in primary care. J Fam Pract. 2012;61(Suppl 10):S1–8.
14. Noh RM, Graveling AJ, Frier BM. Medically minimising the impact of hypoglycaemia in type 2 diabetes: a review. Expert opinion on pharmacotherapy. 2011;12(14):2161–75.
15. UK Hypoglycaemia Study Group. Risk of hypoglycaemia in types 1 and 2 diabetes: effects of treatment modalities and their duration. Diabetologia. 2007;50(6):1140–7.
16. Garber AJ, Clauson P, Pedersen CB, Kølendorf K. Lower risk of hypoglycemia with insulin detemir than with neutral protamine hagedorn insulin in older persons with type 2 diabetes: a pooled analysis of phase III trials. Journal of the American Geriatrics Society. 2007;55(11):1735–40.
17. BRUNTON S. Safety and effectiveness of modern insulin therapy. Current Issues in the Management of Type 2 Diabetes. 2009:13.
18. Fakhoury W, Lockhart I, Kotchie RW, Aagren M, LeReun C. Indirect comparison of once daily insulin detemir and glargine in reducing weight gain and hypoglycaemic episodes when administered in addition to conventional oral anti-diabetic therapy in patients with type-2 diabetes. Pharmacology. 2008;82(2):156–63.
19. Chan SP, Colagiuri S. Systematic review and meta-analysis of the efficacy and hypoglycemic safety of gliclazide versus other insulinotropic agents. Diabetes Research and Clinical Practice. 2015;110(1):75–81.
20. Draeger KE, Wernicke-Panten K, Lomp HJ, et al. Long-term treatment of type 2 diabetic patients with the new oral antidiabetic agent glimepiride (Amaryl): a double-blind comparison with glibenclamide. Horm Metab Res. 1996;28(9):419,425
21. Phung OJ, Scholle JM, Talwar M, Coleman CI. Effect of noninsulin antidiabetic drugs added to metformin therapy on glycemic control, weight gain, and hypoglycemia in type 2 diabetes. JAMA. 2010;303(14):1410–8.
22. Viswanathan M, Joshi SR, Bhansali A. Hypoglycemia in type 2 diabetes: Standpoint of an experts’ committee (India hypoglycemia study group). Indian Journal of Endocrinology and Metabolism. 2012;16(6):894.

## **Technologies**

### **RSSDI 2017 recommendations**

#### **Recommended care**

- Technology in diabetes management may be used in all patients for better outcome and to minimize complications.
- Diabetes technology should be used for prevention, screening, and management of patients with T2DM and to improve their QoL.
- Technology-assisted tools, including telemedicine, dedicated electronic health records, m-Health, e-Health, and mobile applications, may be advantageous for effective lifestyle modification to prevent diabetes.
- Tools including telemedicine, m-Health, e-Health, and mobile applications may be useful for improving medication adherence with reducing overall complications and modifying risk factors in patients with T2DM.

#### **Limited care**

- Barriers for diabetes technology like cost, regulatory permissions, patient literacy, data protection, and data security should be considered, and therefore, use of technology should be individualized.

#### **Preamble**

Technology in diabetes imparts both educational and motivational assistance and improves the healthcare system for diabetes patients. Technology involving mobile applications, internet portals, and websites helps in daily diabetes self-management activities including blood glucose monitoring, online diabetes education, calculation of insulin dose, exercise regimes, healthy eating, taking medication, monitoring for complications, and problem-solving [1, 2]. Furthermore, diabetes education through technology enables the patients to gain knowledge on current practices in the field of diabetes management as well as enables them to guide other patients on management of diabetes. A current statistics report states that 57% (731 million) Indians have mobile phones, out of which, 33.4% (244 million) use a smartphone [3].

Diabetes technology includes the development of markers for diabetes control, advanced monitoring techniques, mathematical models, assessment procedures, and control algorithms [4]. Nonetheless, certain barriers like cost, patient literacy, regulatory permission, data protection, and data security limit the use of the advanced technology in the field of diabetes [2]. Some new technologies and novel therapies are given below [5]:

1. New insulin delivery systems
2. Usage of informatics in the medicinal field
3. Usage of telemedicine
4. Glucose content sensors
5. Closed loop system and algorithms
6. Advanced multidisciplinary approaches for controlling diabetic eye diseases
7. Novel pharmacological approaches to the treatment of T2DM
8. Nanomedicines set to revolutionize the treatment of diabetes

**Table:** 

Mobile applications used for digital management of diabetes (adapted from [2])	
Diet therapy	HealthyOut, Foodily, CarbControl, Lose It, Weight Watchers, Daily Burn, Calorie Counter PRO, iCookbook Diabetic, Fooducate, EatLocal, CalorieKing, HEALTHeDiabetes
Physical exercise	Track 3, Strava, MyFitnessPal, Moves, Pacer, Steps Pedometer and Step Counter Activity Tracker, Map My Walk, Stepz, Walker-Pedometer Lite, Footsteps, iRunner and Runtastic Pedometer, Fitbit, the Jawbone Up24, the Nike Fuelband
Blood glucose controlling	Diabetic, Diabetes in Check, Diabetes Companion, My Sugar Junior, Go Meal, Glooko, Glucose Buddy, DiabetesApp Lite, CareLink, LibreView, Accu-Chek Connect
Online diabetes education	Diabetes EDC, Point of Care Diabetes, Diabetes Journal, Prognosis Diabetes, Diabetes Forecast, Diabetes Forum, and Diabetes FAQ
Insulin dose calculators	Insulin Calculator, Bolus Calc, Insulin Dose Calculator Pro, Diabetes Personal Calculator
Medication adherence	MyMedSchedule, My Meds, MedSimple, Medagenda, Pillmanager, Pill Reminder, RxmindMe Prescription
Digital healthcare information	Kaiser Permanente (My Health manager), EPIC (My Chart), and VistA (U.S. Department of Veterans Affairs)

#### **Considerations**

During framing the recommendations, the applicability, usefulness, and barriers of novel technologies and newer therapies for diabetes management especially in Indian patients should be considered.

#### **Rationale and evidence**

##### *Technology for glucose monitoring (skin patches)*

- Flash glucose monitoring (FGM) or sensing technology is a new tool used over CGM or SMBG for glucose monitoring in insulin-treated T2DM patients (FGM is a new tool for CGM in patients with diabetes). It is made with a small, round sensor with microfilament that measures glucose levels in the interstitial fluids.
- A recent multicenter, open-label RCT reports that time spent by participants in hypoglycemia < 70 and <55 mg/dL reduced by 43 and 53%, respectively, with FGM compared to SMBG (*p* = 0.0014) [6]. However, no change in A1C was detected.
- An Indian study including 388 T2DM patients also reported statistically significant reductions from baseline in A1C (*p* < 0.0001), FPG (*p* < 0.0001), and BMI (*p* = 0.0226) levels after 6 months of undergoing FGM [7].
- Furthermore, evidence suggests that professional (masked) continuous glucose monitoring (P-CGM) system and factory-calibrated glucose monitoring (F-CGM) system can influence patients for diabetes self-care practices, which in turn results in glycemic control enhancement over a wide range of baseline therapies [8, 9].

##### *Telemedicine*

- Telemedicine is used for distant management of the disease such as screening, prevention, and treatment by means of telecommunications technology. Diabetes Tele Management System (DTMS®) is a telemedicine-based system which provides individualized therapy advice for diabetes management by considering each individual’s A1C, BP, and LDL levels and presence of any comorbid conditions [10].
- A retrospective cohort study comprising 1000 T2DM patients reports that DTMS® was safe and cost-effective in the intensive treatment of T2DM without serious comorbidities and avoided limitations of a traditional healthcare such as very frequent physical visits for each and every drug dose adjustment, diet, and exercise advice [11].
- *Mohan et al*. used telemedicine intervention for the prevention of diabetes through The Chunampet Rural Diabetes Prevention Project (CRDPP) [12].
- Furthermore, evidence report that tele-screening for diabetic retinopathy in Indians was cost-effective compared with no screening [13, 14].

##### *Mobile health (m-Health)*

- A systematic review revealed that mobile phone technology improved health outcomes for chronic disease conditions such as diabetes in Asian Indian patients [15]. Furthermore, a review by *Muralidharan et al*. reports that m-Health including short message service (SMS) and applications for medication reminders and insulin optimization had a great role in prevention and management of T2DM [16].
- In a randomized trial which evaluated the outcomes of mobile reminder in opportunistic screening for T2DM in a primary healthcare setting, more participants in the intervention arm (85.7%) returned for definitive test compared to control arm (53.3%) (RR, 1.61; 95% CI, 1.35–1.91) [17]. A multisite RCT compared the outcome of m-Health among people with T2DM in India. The study reports that significantly more participants in m-Health than usual care had improved medication adherence (39.0 vs 12.8%; *p* = 0.03) and increased the frequency of blood glucose self-testing (39.0 vs 10.3%; *p* = 0.01) at 6 months [18].
- Furthermore, diabetes prevention trial revealed that a pragmatic and scalable strategy using mobile technology promotes sustained lifestyle changes and prevents people from developing T2DM [19]. Mobile phone messaging is an economical way to provide educational and motivational advice about lifestyle modification. A prospective, parallel group RCT reports that fewer participants in the mobile messaging group had developed T2DM than standard care group (18 vs 27%; HR, 0.64; 95% CI, 0.45–0.92; *p* = 0.015) in a period of 3 years [20]. An observational study compared the outcomes of M-health/E-health with conventional care in 109 diabetes patients. Diabetes knowledge scores (19.9 ± 2.5 vs 17.9 ± 3.98, *p* = 0.005) and QoL indices (88.5 ± 7.8 vs 83.5 ± 10.7, *p* = 0.015) showed a statistically significant improvement in the intervention arm; however, no significant difference was observed in glycemic control parameters between arms [21].
- Furthermore, Lifestyle Modification in Information Technology (LIMIT) reduced various risk factors for T2DM like overweight/obesity, hypertriglyceridemia, high LDL-C, and low HDL-C with less cost compared to control in young employees [22]. In addition, Welltang—a smartphone-based diabetes management application—resulted in statistically significant improvements in A1C, blood glucose, and satisfaction in Chinese people with diabetes [23].

#### **Implementation**

With more and more patients, as well as the healthcare practitioners, being comfortable with, and are having expertise in using the novel and advanced technologies, diabetes technology has become an attractive and beneficial option for diabetes management. However, large RCTs are needed to establish the effectiveness, safety, and cost-benefits in improving diabetes-related outcomes. Nonetheless, the future of the digital health industry is encouraging despite many challenges to overcome.

**References**

1. Hunt CW. Technology and diabetes self-management: an integrative review. World Journal of Diabetes. 2015;6(2):225.
2. Shah VN, Garg SK. Managing diabetes in the digital age. Clinical Diabetes and Endocrinology. 2015;1(1):16.
3. Mobile and smartphone usage statistics for India. Available from: https://apsalar.com/2016/03/mobile-and-smartphone-usage-statistics-for-india/
4. Kovatchev BP. Diabetes technology: markers, monitoring, assessment, and control of blood glucose fluctuations in diabetes. Scientifica. 2012.
5. Novel Technologies for the treatment of diabetes. 11th Asia Pacific Diabetes Conference and Expo. July 11–12, 2016 Brisbane, Australia.
6. Haak T, Hanaire H, Ajjan R, Hermanns N, Riveline JP, Rayman G. Flash glucose-sensing technology as a replacement for blood glucose monitoring for the management of insulin-treated type 2 diabetes: a multicenter, open-label randomized controlled trial. Diabetes Therapy. 2016:1–9.
7. Kesavadev J, Krishnan G, Saboo B, Shankar A, Ashok AD, Sanal G, Ramachandran L, Jothydev S. Better outcomes in type 2 diabetes management with a user friendly flash glucose monitoring system: freestyle Libre pro. Advanced Technologies and Treatments for Diabetes. 2017; poster no 031
8. Kesavadev J, Vigersky R, Shin J, Pillai PB, Shankar A, Sanal G, Krishnan G, Jothydev S. Assessing the therapeutic utility of professional continuous glucose monitoring in type 2 diabetes across various therapies: a retrospective evaluation. Advances in Therapy. 2017:1–0.
9. Kesavadev J, Shankar A, Ashok AD, Srinivas S, Ajai NA, Sanal G, Krishnan G, Ramachandran L, Jothydev S. Our first 825 T2DM patients on 14-day factory-calibrated glucose monitoring system: clinical utility and challenges. Journal of Diabetes Science and Technology. 2017:1932296817717504.
10. Kesavadev J, Saboo B, Shankar A, Krishnan G, Jothydev S. Telemedicine for diabetes care: an Indian perspective-feasibility and efficacy. Indian Journal of Endocrinology and Metabolism. 2015 Nov;19(6):764.
11. Kesavadev J, Shankar A, Pillai PB, Krishnan G, Jothydev S. Cost-effective use of telemedicine and self-monitoring of blood glucose via Diabetes Tele Management System (DTMS) to achieve target glycosylated hemoglobin values without serious symptomatic hypoglycemia in 1,000 subjects with type 2 diabetes mellitus—a retrospective study. Diabetes Technology & Therapeutics. 2012;14(9):772–6.
12. Mohan V, Deepa M, Pradeepa R, Prathiba V, Datta M, Ravikumar S, Rakesh H, Sucharita Y, Webster P, Allender S, Kapur A. Prevention of diabetes in rural India with a telemedicine intervention. Journal of Diabetes Science and Technology. 2012;6(6):1355–64.
13. Rachapelle S, Legood R, Alavi Y, Lindfield R, Sharma T, Kuper H, Polack S. The cost–utility of telemedicine to screen for diabetic retinopathy in India. Ophthalmology. 2013;120(3):566–73.
14. Raman R, Bhojwani DN, Sharma T. How accurate is the diagnosis of diabetic retinopathy on telescreening? The Indian scenario. Rural Remote Health. 2014;14:2809.
15. Sahu M, Grover A, Joshi A. Role of mobile phone technology in health education in Asian and African countries: a systematic review. International Journal of Electronic Healthcare. 2014;7(4):269–86.
16. Muralidharan S, Ranjani H, Anjana RM, Allender S, Mohan V. Mobile health technology in the prevention and management of type 2 diabetes. Indian Journal of Endocrinology and Metabolism. 2017;21(2):334.
17. Kumar S, Shewade HD, Vasudevan K, Durairaju K, Santhi VS, Sunderamurthy B, Krishnakumari V, Panigrahi KC. Effect of mobile reminders on screening yield during opportunistic screening for type 2 diabetes mellitus in a primary health care setting: a randomized trial. Preventive Medicine Reports. 2015;2:640–4.
18. Kleinman NJ, Shah A, Shah S, Phatak S, Viswanathan V. Improved medication adherence and frequency of blood glucose self-testing using an m-Health platform versus usual care in a multisite randomized clinical trial among people with type 2 diabetes in India. Telemedicine and e-Health. 2017.
19. Priscilla S, Nanditha A, Simon M, Satheesh K, Kumar S, Shetty AS, Snehalatha C, Johnston DG, Godsland IF, Wareham NJ, Ramachandran A. A pragmatic and scalable strategy using mobile technology to promote sustained lifestyle changes to prevent type 2 diabetes in India—outcome of screening. Diabetes Research and Clinical Practice. 2015;110(3):335–40.
20. Ramachandran A, Snehalatha C, Ram J, Selvam S, Simon M, Nanditha A, Shetty AS, Godsland IF, Chaturvedi N, Majeed A, Oliver N. Effectiveness of mobile phone messaging in prevention of type 2 diabetes by lifestyle modification in men in India: a prospective, parallel-group, randomised controlled trial. The Lancet Diabetes & Endocrinology. 2013;1(3):191–8.
21. Jha S, Dogra S, Yadav A, Siddiqui S, Panda M, Srivastava K, Raghuvanshi L, Kaur S, Bhargava A, Mathur R, Gupta SK. A prospective observational study to assess the effectiveness of an electronic health (E-health) and mobile health (M-health) platform versus conventional care for the management of diabetes mellitus. International Journal of Diabetes in Developing Countries. 2016;36(4):529–34.
22. Limaye T, Kumaran K, Joglekar C, Bhat D, Kulkarni R, Nanivadekar A, Yajnik C. Efficacy of a virtual assistance‐based lifestyle intervention in reducing risk factors for type 2 diabetes in young employees in the information technology industry in India: LIMIT, a randomized controlled trial. Diabetic Medicine. 2016.
23. Zhou W, Chen M, Yuan J, Sun Y. Welltang—a smart phone-based diabetes management application—improves blood glucose control in Chinese people with diabetes. Diabetes Research and Clinical Practice. 2016; 116:105–10.

**Acknowledgements** The authors thank Rabi Narayan Panigrahy and Syam Kumar Yelamanchi from Jeevan Scientific Technology Limited (Hyderabad, India) for providing writing and editorial support in the development of this guideline.

## **Annexures**

### **Screening/early detection of diabetes/prediabetes**

#### **Annexure I**

##### **The Indian Diabetes Risk Score**

- The tool encompasses four parameters, age, abdominal obesity, family history of diabetes, and physical activity to detect T2DM, and also helps to distinguish T2DM from non-T2DM.
- A maximum score of 100 is given for these categories combined as shown in the figure.
  - Subjects with an IDRS of < 30 are categorized under low risk, 30–50 as medium risk, and those with > 60 as high risk for diabetes.
  - Similarly WC ≥ 90 cm, sedentary lifestyle, and family history of diabetes are indicators for high risk of diabetes.
  - Limiting the blood sugar testing to those with an IDRS score of 50 and above could identify more than 90% of Indians with diabetes and prediabetes.

**Table:** 

Parameter	Score
*Age*	
< 35 years	0
35–49 years	20
≥ 50 years	30
*Waist circumference*	
Waist < 80 cm (female), < 90 cm (male)	0
Waist ≥ 80–89 cm (female), ≥ 90–99 cm (male)	10
Waist ≥ 90 cm (female), ≥ 100 cm (male)	20
*Physical activity*	
Regular vigorous exercise or strenuous (manual) activity at home/work	0
Regular moderate exercise or moderate physical activity at home/work	10
Regular mild exercise or mild physical activity at home/work	20
No exercise and/or sedentary activities at home/work	30
*Family history of diabetes*	
No diabetes in parents	0
One parent is diabetic	10
Both parents are diabetic	20

Minimum score = 0; maximum score = 100; positive score ≥ 60/100

**References**

1. Mohan V, Sandeep S, Deepa M, Gokulakrishnan K, Datta M, Deepa R. A diabetes risk score helps identify metabolic syndrome and cardiovascular risk in Indians—the Chennai Urban Rural Epidemiology Study (CURES-38). Diabetes, Obesity & Metabolism. 2007;9(3):337–343.
2. Mohan V, Deepa M, Farooq S, Narayan KM, Datta M, Deepa R. Anthropometric cut points for identification of cardiometabolic risk factors in an urban Asian Indian population. Metabolism. 2007;56(7):961–968.
3. Mohan V, Deepa M, Farooq S, Prabhakaran D, Reddy KS. Surveillance for risk factors of cardiovascular disease among an industrial population in southern India. Natl Med J India. 2008;21(1):8–13.
4. Mohan V, Deepa M, Anjana RM, Lanthorn H, Deepa R. Incidence of diabetes and pre-diabetes in a selected urban south Indian population (CUPS-19). J Assoc Physicians India. 2008;56:152–157.
5. Mohan V, Sandeep S, Deepa M, Gokulakrishnan K, Datta M, Deepa R. A diabetes risk score helps identify metabolic syndrome and cardiovascular risk in Indians—the Chennai Urban Rural Epidemiology Study (CURES-38). Diabetes, Obesity & Metabolism. 2007;9(3):337–343.
6. Mohan V, Deepa R, Deepa M, Somannavar S, Datta M. A simplified Indian Diabetes Risk Score for screening for undiagnosed diabetic subjects. J Assoc Physicians India. 2005;53:759–763.
7. Sharma KM, Ranjani H, Nguyen H, et al. Indian Diabetes Risk Score helps to distinguish type 2 from non-type 2 diabetes mellitus (GDRC-3). J Diabetes Sci Technol. 2011;5(2):419–425

#### **Annexure II**

Dr. A. Ramachandran’s Diabetes Risk Score for Indians can be used to identify people having undetected diabetes. The likelihood of detecting diabetes in people with a score of 21 or more is high and requires testing with an oral glucose tolerance test.

**Table:** 

Variables	Risk score
Age (30–44) years	10
Age (45–59) years	18
Age (> 59) years	19
Family history of diabetes	7
Body mass index (≥ 25) kg/m^2^	7
Waist (M ≥ 85, W ≥ 80 cm)	5
Sedentary physical activity	4
Maximum score	42

**Reference**

Ramachandran A, Snehalatha C, Vijay V, Wareham NJ, Colagiuri S., Derivation and validation of diabetes risk score for urban Asian Indians. Diabetes Res Clin Pract. 2005;70(1):63–70.

### **Diet therapy**

#### **Annexure III**

**Table:** 

General recommendations for diet in diabetes patients
• When blood glucose is under control, 100 g of fruit (e.g., papaya, sweet lime, orange, guava) should be allowed daily
• Whole fruits are recommended rather than fruit juices
• At least one vegetable dish has to be included in the daily menu
• Roots and tubers can be consumed once a week by patients with diabetes but should be included as calorie suppliers
• Low-calorie foods like tea, coffee, skimmed milk (without sugar), buttermilk, and salads are allowed for patients with diabetes • Cream from milk should be removed before consuming

**Reference**

1. Shobana et al. Awareness about dietary factors in patients before exposure to diabetes education. IJDDC. 2000; 20;85–87.

#### **Annexure IV**

**Table:** 

Diet in diabetes patients with established CVD		
**Highly recommended**	**Moderately recommended**	**Not recommended**
Leafy vegetables, vegetable salads, fruits, whole grain, coarse grains, nuts, sprouted grams, spices, and all other foods which are rich in fiber and antioxidants	Low fat milk and milk products, vegetable oils with MUFA and PUFA, flesh foods (fish, chicken without skin, red meats), white of the egg, and artificial sweeteners	Alcohol, refined sugar, beverages with HFCS, industrial *trans* fat, excess fats and foods that are refined, processed, salt-rich, and deep-fried

*CVD* cardiovascular disease, *MUFA* mono-unsaturated fatty acids, *PUFA* polyunsaturated fatty acids, *HFCS* high fructose corn syrup

**Reference**

1. Raghuram TC. Diet in diabetes with heart disease. IJDDC. 1995;14;123–6.
2. Mozaffarian D. Dietary and policy priorities for cardiovascular disease, diabetes, and obesity. Circulation. 2016;133(2):187–225.

### **Lifestyle management**

#### **Annexure V**

**Table:** 

General recommendations: exercise therapy in diabetes
• Patients with diabetes should exercise as part of their medical management
• Exercise used to reduce weight should be combined with dietary measures
• Moderate intensity aerobic activity and resistance training should be part of the exercise regimen
• Multiple short exercise sessions lasting at least 10 min each in course of a day are useful
• Exercise should be appropriate to the persons general physical condition and lifestyle
• Use proper foot wear and if appropriate, use protective equipment
• Avoid exercise in extreme hot or cold conditions
• Inspect feet before and after exercise

## **Injectables**

### **Insulin therapy**

#### **Annexure VI**

##### **Approaches for initiating insulin**


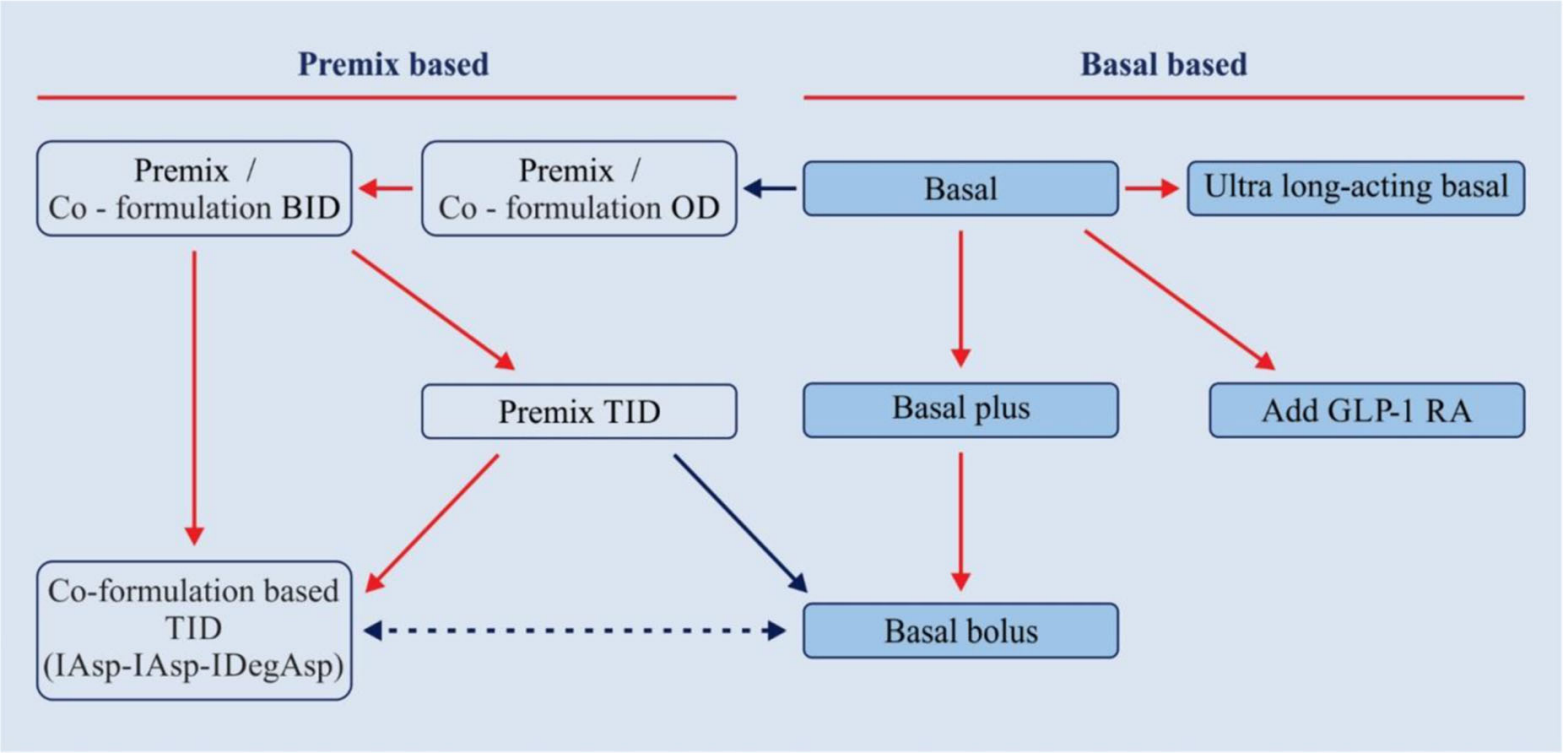


*OD* once daily, *BID* twice daily, *TID* three times a day, *GLP-1 RA* glucagon like peptide-1 receptor agonist, *IAsp* Insulin aspart, *IDegAsp* mix of insulin degludec and insulin aspart
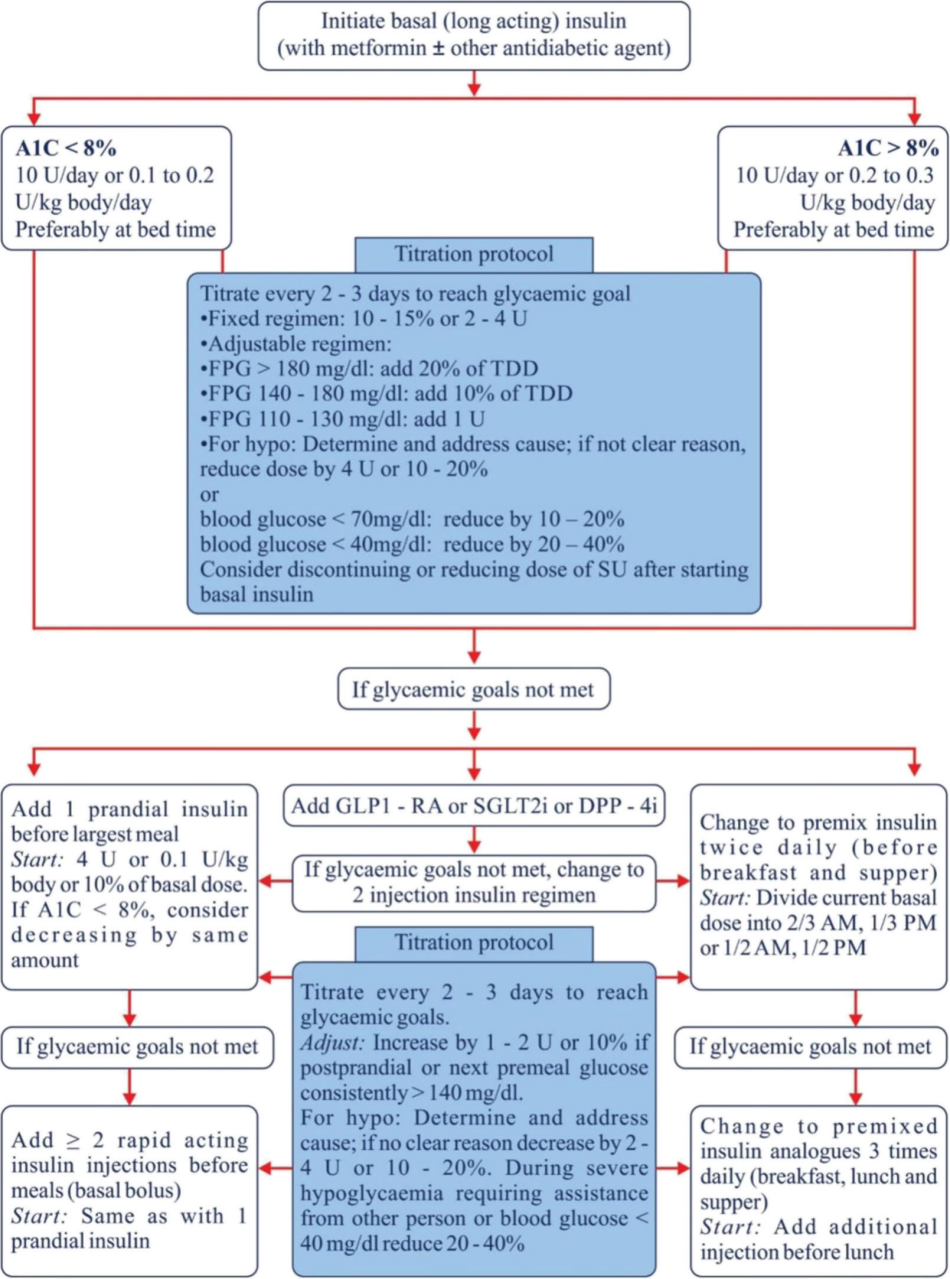


*TDD* total daily dose, *GLP-1 RA* glucagon like peptide-1 receptor agonist, *SGLT2i* sodium glucose co-transporter 2 inhibitors, *DPP-4i* dipeptidyl peptidase-4 inhibitors

**References**

1. American Diabetes Association. Standards of medical care in diabetes. 2017. Available at: http://professional.diabetes.org/sites/professional.diabetes.org/files/media/dc_40_s1_final.pdf
2. Garber AJ, Abrahamson MJ, Barzilay JI, Blonde L, Bloomgarden ZT, Bush MA, Dagogo-Jack S, DeFronzo RA, Einhorn D, Fonseca VA, Garber JR. Consensus statement by the American Association of Clinical Endocrinologists and American College of Endocrinology on the comprehensive type 2 diabetes management algorithm—2017 executive summary. Endocrine Practice. 2017;23(2):207–38.

#### **Annexure VII**

**Table:** 

**Steps for initiation of basal insulin therapy**		
	**Glucose value**	**Total daily dose**
**Step 1. Initiation with basal insulin** ^**a**^	A1C < 8%	0.1–0.2 units/kg
	A1C > 8%	0.2–0.3 units/kg
**Step 2. Titration**^**b**^ (every 2–3 days to reach glycemic goals)	Fixed regimen	Increase by 2 units/day
	Adjustable regimen	
	FPG > 180 mg/dL	Add 4 units
	FPG 140–180 mg/dL	Add 2 units
	FPG 110–139 mg/dL	Add 1 unit
**Step 3. Monitor for hypoglycemia**	BG < 70 mg/dL	Reduce by 10 to 20%
	BG < 40 mg/dL	Reduce by 20 to 40%

*A1C* glycated hemoglobin, *BG* blood glucose, *FPG* fasting plasma glucose, *NPH* neutral protamine Hagedorn, *SU* sulfonylureas

^a^Consider discontinuing SU therapy and basal analogues should be preferred over NPH insulin

^b^For most patients with T2D taking insulin, glucose goals are A1C < 7% and fasting and premeal blood glucose < 110 mg/dL in the absence of hypoglycemia. A1C and FPG targets may be adjusted based on patients age, duration of diabetes, presence of comorbidities, diabetic complications, and hypoglycemia risk

**Reference**

1. Handelsman Y, Bloomgarden ZT, Grunberger G, Umpierrez G, Zimmerman RS, Bailey TS, et al. American Association of Clinical Endocrinologists and American College of Endocrinology—clinical practice guidelines for developing a diabetes mellitus comprehensive care plan—2015. Endocr Pract. 2015;21 Suppl 1:1–87.

#### **Annexure VIII**

##### **Steps for initiating premixed insulin**


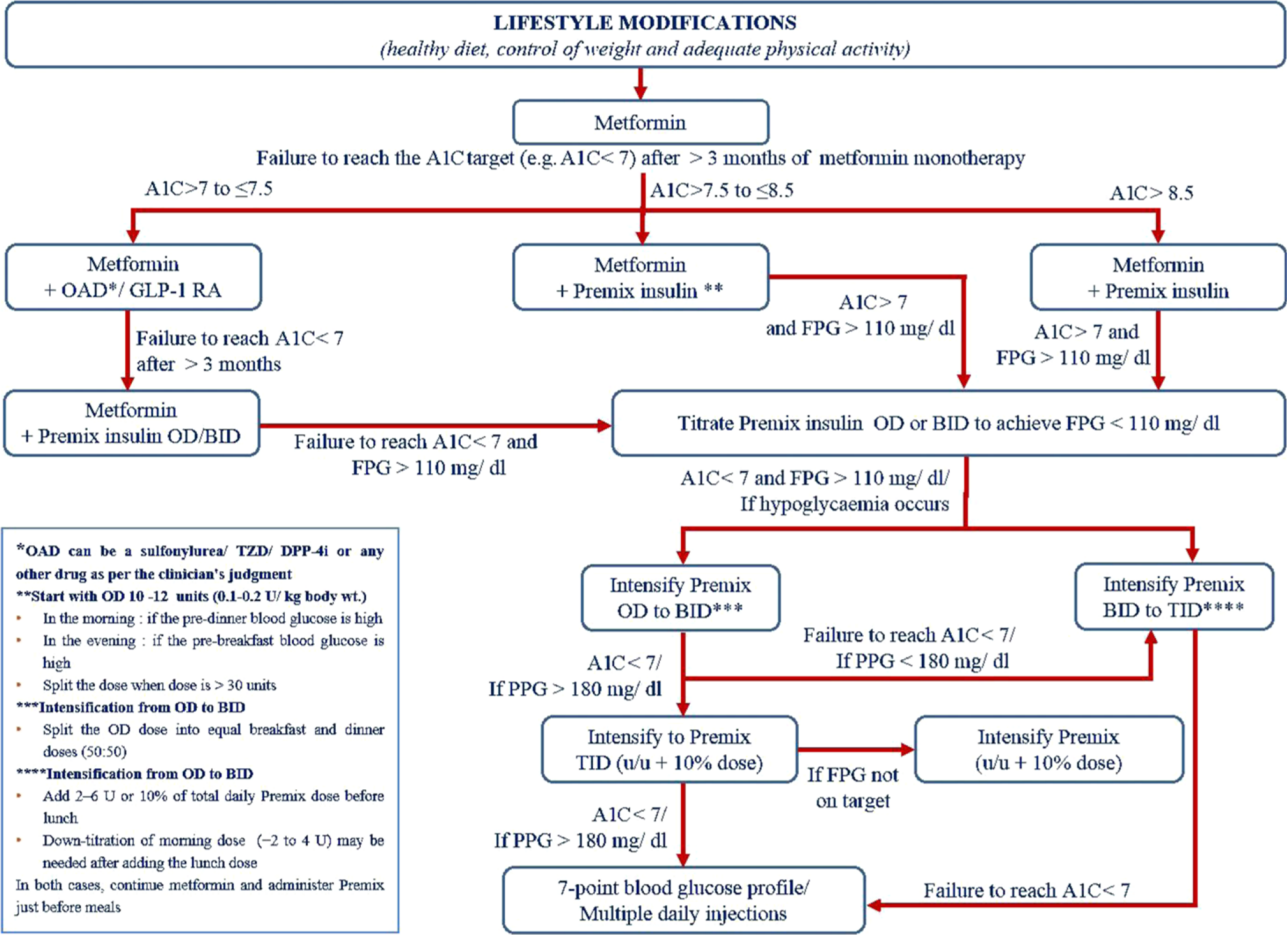


*OAD* oral antidiabetic agents, *GLP-1 RA* glucagon like peptide-1 receptor agonist, *OD* once daily, *BID* twice daily, *TID* three times in a day, *TZD* thiazolidinedione, *DPP-4I* dipeptidyl peptidase-4 inhibitors

#### **Annexure IX**

**Table:** 

**Steps for intensification of insulin therapy**		
	**Therapeutic option**	**Total daily dose**
**Step 1. Add prandial insulin**	When glycemic targets are unmet	TDD 0.3–0.5 units/kg (40–50% basal: 50–60% prandial)^a^
**Step 2. Titration**^**b**^ (every 2–3 days to reach glycemic goals)	Fixed regimen (prandial insulin)	Increase TDD by 2 units/day
	Adjustable regimen (prandial insulin)	
	FPG > 180 mg/dL	Increase TDD by 4 units
	FPG 140–180 mg/dL	Increase TDD by 2 units
	FPG 110–139 mg/dL	Increase TDD by 1 unit
	2-h PPG or next premeal glucose > 180 mg/dL	Increase prandial dose for the next meal by 10%
	Premixed insulin	
	FPG/premeal BG > 180 mg/dL	Increase TDD by 10%
**Step 3. Monitor for hypoglycemia**	Fasting hypoglycemia	Reduce basal insulin dose
	Night time hypoglycemia	Reduce basal insulin or reduce short/rapid-acting insulin taken before supper or evening snack
	Between meal hypoglycemia	Reduce previous premeal short/rapid-acting insulin

*BG* blood glucose, *DPP-4* dipeptidyl peptidase 4 inhibitors, *FPG* fasting plasma glucose, *GLP-1* glucagon-like peptide 1 receptor agonists, *NPH* neutral protamine Hagedorn, *PPG* postprandial glucose, *SGLT2* sodium glucose cotransporter 2, *TDD* total daily dose

^a^Basal + prandial insulin analogues preferred over NPH + regular insulin or premixed insulin

^b^For most patients with T2D taking insulin, glucose goals are A1C < 7% and fasting and premeal blood glucose < 110 mg/dL in the absence of hypoglycemia. A1C and FPG targets may be adjusted based on patient’s age, duration of diabetes, presence of comorbidities, diabetic complications, and hypoglycemia risk

**Reference**

Handelsman Y, Bloomgarden ZT, Grunberger G, Umpierrez G, Zimmerman RS, Bailey TS, et al. American Association of Clinical Endocrinologists and American College of Endocrinology—clinical practice guidelines for developing a diabetes mellitus comprehensive care plan—2015. Endocr Pract. 2015;21 Suppl 1:1–87.

## **Chronic complications**

### **Diabetic neuropathy**

#### **Annexure X**

##### Diabetic neuropathy symptom score (DNS) (Meijer JWG et al. 2002)

**Table:** 

DNS Questionnaire
1. Are you suffering of unsteadiness in walking? *Need for visual control, increase in dark, walk like a drunken man, and lack of contact with floor* 2. Do you have a burning, aching pain or tenderness at your legs or feet? *Occurring at rest or at night, not related to exercise, exclude intermittent claudication* 3. Do you have prickling sensations at your legs and feet *Occurring at rest or at night, distal > proximal, stocking glove distribution* 4. Do you have places of numbness on your legs or feet? *Distal > proximal, stocking glove distribution*
The questions were answered either “Yes” (positive = 1 point) if symptom has occurred during the last 2 weeks or “No” (negative = 0 point) if it did not. Maximum score is 4 and minimum 0

#### **Annexure XI**

##### **Modified neuropathy disability score (NDS) (Boulton 2005)**

**Table:** 

**Neuropathy Disability Score (NDS)**	
**Vibration perception threshold** 128-Hz tuning fork; apex of big toe: normal = can distinguish vibrating/not vibrating	**Normal = 0** **Abnormal = 1**
**Temperature perception on dorsum of the foot** Use tuning fork with beaker of ice/warm water	
**Pinprick** Apply pin proximal to big toe nail just enough to deform the skin: trial pair = sharp blunt: normal = can distinguish sharp/not sharp	
**Achilles reflex**	Present = 0 Present with reinforcement = 1 Absent = 2
	NDS total out of 10

### **Diabetic foot care**

#### **Annexure XII**

##### *Scoring systems to screen for diabetic peripheral neuropathy (Yang Z et al. 2014)*

**Table:** 

Diabetic neuropathy examination
Muscle strength 1. Quadriceps femoris: extension of the knee 2. Tibialis anterior: dorsiflexion of the foot Reflex 1. Triceps surae Sensation: index finger 1. Sensitivity to pinpricks 2. Monofilament and vibration perception threshold Sensation: big toe 1. Sensitivity to pinpricks 2. Sensitivity to touch 1. Vibration perception 2. Sensitivity to joint position
Only the right leg and foot are tested. Scoring from 0 to 2: 0 = Normal 1 = Mild/moderate deficit • Muscle strength: Medical Research Council scale 3–4 • Reflex: decreased but present • Sensation: decreased but present 2 = Severely disturbed/absent • Muscle strength: Medical Research Council scale 0–2 • Reflex: absent • Sensation: absent Maximum score = 16 points

**References**

1. Meijer JWG, Smit AJ, Sonderen EV, Groothoff JW, Eisma WH, Links TP. Symptom scoring systems to diagnose distal polyneuropathy in diabetes: the Diabetic Neuropathy Symptom score. Diabet Med 2002;19:962–5.
2. Boulton AJ. Management of diabetic peripheral neuropathy. Clinical Diabetes. 2005;23(1):9–15.
3. Yang Z, Chen R, Zhang Y, Huang Y, Hong T, Sun F, Ji L, Zhan S. Scoring systems to screen for diabetic peripheral neuropathy. The Cochrane Library. 2014.

### **Infections and vaccinations**

#### **Annexure XIII**

##### Recommended vaccines for diabetic patients

**Table:** 

Vaccines	Dosage
Pneumococcal (polysaccharide)	1 or 2 doses
Influenza	1 dose TIV annually
Tetanus, diphtheria, pertussis (Td/Tdap)	• Substitute one time dose of Tdap; then boost with Td every 10 years
Measles, mumps, rubella (MMR)	• 1 or 2 doses, 4-week interval
Varicella	• 2 doses, at least 4 weeks apart
Zoster	• 1 dose, 60 years
Hepatitis A	• 2 doses at least 6 months apart
Hepatitis B	• 3 doses
Human papillomavirus (HPV) Female	• 3 doses through age 26 years • The second dose should be administered 1–2 months after the first dose • Third dose should be administered 6 months after the first dose (at least 24 weeks after the first dose)
Human papillomavirus (HPV) Male	• 3 doses through age 21 years

**Reference**

1. Kesavadev J, Misra A, Das AK, Saboo B, Basu D, Thomas N, Joshi SR, Unnikrishnan AG, Shankar A, Krishnan G, Unnikrishnan R, Mohan V. Suggested use of vaccines in diabetes. Indian J Endocrinol Metab. 2012;16(6):886–93.

### **Fasting and diabetes**

#### **Annexure XIV**

##### **Categories of risk in patients with T1DM or T2DM who fast during Ramadan**

**Table:** 

Category	Parameter	I [very high risk]	II [high risk]	III [low/moderate risk]
Personal characteristics	Life stage	Childhood/adolescence/pregnancy/lactation/elderly	Late mid age	Healthy adulthood
	Lifestyle	Intense physical labor; potential public health impact of hypoglycemia, e.g., in commercial drivers	Variable duties, e.g., shift workers	Routine lifestyle
	Overall health	Infirm; cognitive dysfunction; severe acute illness	Risk of dehydration; on concomitant steroid therapy	Stable
Diabetes-related characteristics	Type of diabetes	Brittle diabetes; T1DM, poorly controlled	T2DM, poorly controlled T1DM, well controlled	T2DM, well controlled
	Acute complications	h/o severe hypoglycemia/DKA/HHNKC within 3 months prior to Ramadan; h/o recurrent hypoglycemia	None	None
	Chronic complications	h/o hypoglycemia unawareness; CKD stage 4/5; Advanced macrovascular	CKD stage 3, stable macrovascular complications	No complication
Therapeutic characteristics	Non-insulin therapy	Conventional sulfonylurea	Thrice daily regimes	All other therapy
	Insulin therapy	Basal bolus regimes	Thrice daily regimes: basal-plus; premixed tds; rapid-rapid-premix; premix-rapid-premix	Once or twice daily regimes: basal; premixed analogues
Medico-religious advice	Religious suggestion	Listen to medical advice. Do not fast in health is endangered. Be prepared to break the fast if ill health occurs		
	Medical management	Structured education; SMBG; dose titration. Watch for complications, and manage appropriately		

*T1DM* type 1 diabetes, *T2DM* type 2 diabetes, *DKA* diabetes ketoacidosis, *HHNKC* hyperosmolar hyperglycemic non-ketotic coma, *CKD* chronic kidney disease

ᅟ

#### **Annexure XV**

##### **Dose adjustment/modifications for the management of T2DM during Ramadan fast**

**Table:** 

Antidiabetic agents	Current regimen	Recommended dose modification during Ramadan
Metformin	Once daily	• Take at iftar
	Twice daily	• Take at iftar and suhur
	Thrice daily	• Take 2/3 of the total daily dose at the iftar and the other 1/3 at the suhur
Sulfonylureas^a^	Once daily	• Take at iftar
	Twice daily	• Take 1/2 of usual evening dose with the suhur and the usual morning dose with the Iftar
Glinides	• The daily dose may be ↓ or divided to 2 doses according to meal size and should be taken at iftar and suhur	
TZD	• No dose adjustments is required	
DPP-4 inhibitors	• No dose adjustments required	
Acarbose	• No dose adjustments is required	
SGLT-2 inhibitors^b^	• No dose adjustment is required and the dose be taken with iftar	
GLP-1 receptor agonists	• The dose should be titrated 6 weeks prior to Ramadan and no dose adjustment is required	
AGIs	• No dose modification is required	
Long-acting insulin	Once daily	• ↓ dose by 15–30% and take at iftar
	Twice daily	• Take usual morning dose at iftar • ↓ evening dose by 50% and take at suhur
Short-acting insulin		• Take normal dose at iftar and lunch-time dose at dinner • ↓ suhur dose by 50%
Premixed insulin	Once daily	• Take normal dose at iftar
	Twice daily	• Take 1/2 of usual evening dose with the suhur and the usual morning dose with the iftar
	Thrice Daily	• Omit afternoon dose and adjust iftar and suhur doses • Carry out dose titration every 3 days
Insulin pump	Basal rate	• ↓ dose by 20–40% in the last 3–4 h of fasting • ↑ dose by 0–30% early after iftar
	Bolus rate	• Normal carbohydrate counting and insulin sensitivity principles apply

*AGIs* alpha glucosidase inhibitors, *DPP-4* dipeptidyl peptidase-4, *SGLT-2* sodium-glucose co-transporter-2, *TZD* thiazolidinedione

^a^Gliclazide and glimepiride should be preferred among all other sulfonylureas

^b^Elderly patients, patients with renal impairment, hypotensive individuals, those at risk of dehydration, or those taking diuretics should not be treated with SGLT2 inhibitors

**References**

1. Kalra S, Aamir AH, Raza A, Das AK, Khan AA, Shrestha D, Qureshi MF, Fariduddin M, Pathan MF, Jawad F, Bhattarai J. Place of sulfonylureas in the management of type 2 diabetes mellitus in South Asia: a consensus statement. Indian Journal of Endocrinology and Metabolism. 2015;19(5):577.
2. Ibrahim M, Al Magd MA, Annabi FA, Assaad-Khalil S, Ba-Essa EM, Fahdil I, Karadeniz S, Meriden T, Misha AA, Pozzilli P, Shera S. Recommendations for management of diabetes during Ramadan: update 2015. BMJ Open Diabetes Research and Care. 2015;3(1):e000108.
3. Hassanein M, Al-Arouj M, Hamdy O, Bebakar WM, Jabbar A, Al-Madani A, Hanif W, Lessan N, Basit A, Tayeb K, Omar MA. Diabetes and Ramadan: practical guidelines. Diabetes Research and Clinical Practice. 2017.
4. Al-Arouj M, Bouguerra R, Buse J, Hafez S, Hassanein M, Ibrahim MA, Ismail-Beigi F, El-Kebbi I, Khatib O, Kishawi S, Al-Madani A. Recommendations for management of diabetes during Ramadan. Diabetes Care. 2005;28(9):2305–11.

### **Diabetes and CV risk**

#### **Annexure XVI**

##### **Statin treatment in patients with diabetes**

**Table:** 

Risk	Age	Statins
ASCVD risk factors^a^	< 40 years	Moderate^b^/high intensity statins^c^
	40–75 years	High intensity statins
	> 75 years	Moderate/high intensity statins
ASCVD	< 40 years	High intensity statins
	40–75 years	High intensity statins
	> 75 years	High intensity statins

^a^ASCVD risk factors include LDL cholesterol ≥ 100 mg/dL (2.6 mmol/L), high blood pressure, smoking, overweight and obesity, and family history of premature ASCVD

^b^Moderate intensity statins (once daily): Atorvastatin 10–20 mg, Rosuvastatin 5–10 mg, Simvastatin 20–40 mg, Pravastatin 40–80 mg, Lovastatin 40 mg, Fluvastatin XL 80 mg, Pitavastatin 2–4 mg

^c^High intensity statins (once daily): Atorvastatin 40–80 mg, Rosuvastatin 20–40 mg atherosclerotic cardiovascular disease (ASCVD)

**Reference**

1. American Diabetes Association. 8. Cardiovascular disease and risk management. Diabetes Care. 2016;39 (Supplement 1):S60–71.

